# Acute lymphoblastic leukemia in patients treated with lenalidomide for multiple myeloma: a safety meta-analysis of randomized controlled trials combined with a retrospective study of the WHO’s pharmacovigilance database

**DOI:** 10.1038/s41408-024-01154-z

**Published:** 2024-10-14

**Authors:** Pierre-Marie Morice, Sabine Khalife-Hachem, Marion Sassier, Véronique Lelong-Boulouard, Alina Danu, Florence Pasquier, Aline Renneville, Charles Dolladille, Jean-Baptiste Micol

**Affiliations:** 1 Department of Pharmacology, Caen-Normandy University Hospital, Caen, France; 2 Normandie Univ, UNICAEN, INSERM U1086 ‘Interdisciplinary Research Unit for Cancers Prevention and Treatment’ (ANTICIPE), Caen, France; 3 Department of Hematology, Gustave Roussy, Université Paris-Saclay, Villejuif, France; 4 Normandie University, UNICAEN, INSERM COMETE, Caen, France; 5 INSERM U1287, Gustave Roussy, Université Paris-Saclay, Villejuif, France; 6 Interception Program, Personalized Cancer Prevention Center, Gustave Roussy, Université Paris-Saclay, Villejuif, France; 7 PICARO Cardio-Oncology Program, Department of Pharmacology, Caen-Normandy University Hospital, Caen, France

**Keywords:** Acute lymphocytic leukaemia, Risk factors

Dear Editor,

Since the first Food and Drug Administration (FDA) approval of lenalidomide in treating patients with myelodysplastic syndromes (MDS) in 2005, its indications have been extended to lymphomas and multiple myeloma (MM). While the risk of lenalidomide-related acute myeloid leukemia (AML) and MDS is well known, long-term follow-up of RCTs also reported few cases of acute lymphoblastic leukemia (ALL) in patients with newly diagnosed MM after lenalidomide exposure [1, 2]. To date, neither the risk nor incidence of this unexpected adverse event has been yet studied in details.

In the first part of this study, we assessed both the risk and incidence of lenalidomide-associated ALL in the MM setting throughout a systematic review and safety meta-analysis of randomized controlled trials (RCTs) according to PRISMA harms checklist [3] (Supplementary Table 1; PROSPERO, CRD42024495677). RCTs were identified by reviewing the literature (Supplementary Table 2) in MEDLINE, Cochrane Central Register of Controlled Trials (until November 15, 2023), followed by the ClinicalTrials.gov and ClinicalTrialsRegister.eu registry websites (until November 17, 2023). Eligibility criteria for study inclusion were RCTs comparing lenalidomide (also known as CC-5013 or L04AX042) versus control (placebo or open-label) in adult patients (age ≥18 years) with MM. Only RCTs with available safety data of interest were included in analysis. The primary outcome was the summary risk of ALL associated to lenalidomide versus placebo in RCTs in patients with MM. The secondary outcomes were the summary risk and incidence of ALL associated to lenalidomide versus control treatment in RCTs. To address it, we performed a fixed-effect meta-analysis to compute Peto odds ratios (ORs) with 95% CIs, a dedicated method for binary studies with rare events (Supplementary Methods) [4]. The summary incidences were computed with the logit transformation and inverse variance weighting. Subgroup or sensitivity analyses were also conducted to explore possible sources of heterogeneity or inconsistency as well as robustness of analysis. Estimates were computed with R (version 4.3.1, including package meta) and presented in forest plots. A two-sided *P*-value < 0.05 in Z-tests (for overall effect) or χ² tests (for overall subgroup comparison) in all estimates was considered statistically significant. The risk of bias was assessed using the Pharmacoepidemiological Research on Outcomes of Therapeutics by a European Consortium checklist tool [5]. The publication bias was assessed graphically by constructing a funnel plot. The quality of evidence was assessed with the Grading of Recommendations Assessment, Development and Evaluation (GRADE) system. In the second part of this study, we described clinical features of lenalidomide-associated ALL reported in VigiBase -the World Health Organization’s pharmacovigilance database- until March 1, 2024 (NCT06251648).

Based on the search strategy of our systematic review, we identified 2982 citations (Supplementary Fig. 1A). After screening, 18 RCTs met the predefined criteria and were included in our safety meta-analysis (Supplementary Table 3). Among them, eight studies were placebo RCTs, ten were open-label RCTs (6 chemotherapy ± SCT-based RCTs and 4 observation RCTs). These 18 RCTs enrolled 5980 patients with MM, of whom 3265 (55%) were included in lenalidomide-based treatment groups and 2715 (45%) were included in control groups. Overall, 20 ALL cases were collected from publications (*n* = 9), clinical trial registry websites (*n* = 8) and principal investigator (*n* = 1; Supplementary Table 4). Median follow-up duration was available in 16 out of 18 RCTs and ranged from 12.9 months to 12.5 years. Based on the 8 placebo RCTs (*n* = 2362 patients), lenalidomide significantly increased the risk of ALL compared to placebo treatment (Peto OR 3.82 [95% CI 1.22–11.92], *p* = 0.02), with no heterogeneity across the studies (*I*² = 0%, χ² *p* = 0.72; Fig. 1A; Supplementary Fig. 2). The incidence of lenalidomide-associated ALL was 1.41% (95% CI 0.81–2.44; *I*² = 0%, χ² *p* = 0.47) across placebo RCTs, 1.68% (0.73–3.84; *I*² = 9%, χ² *p* = 0.36) across chemotherapy ± SCT-based RCTs, 0.46% (0.21–0.98; *I*² = 0%, χ² *p* = 0.68) across observation RCTs, and 1.09% (0.73–1.61; *I*² = 17%, *χ² p* = 0.25; Fig. 1B) across all RCTs (placebo, chemotherapy ± SCT-based, observation). Lenalidomide therapy significantly increased the risk of ALL compared to all control treatments (Peto OR 4.48 [1.85–10.86], *p* < 0.01), with no heterogeneity across the different studies (*I*² = 0%, χ² *p* = 0.93).Details of 7 out of 20 ALL cases were reported in RCTs (Supplementary Table 5) with a median age at ALL onset of 62.6 [IQR 61.4–66.2] years. Median lenalidomide exposure of 4.4 [3.6–8.0] years (range: 2.1–9.3). Median latency period was 5.1 [4.0–8.1] years ranging from 2.1 to 9.4 years, and 5 out of 7 (71%) were alive. Median time to death following ALL onset was 9.6 [7.7–11.5] months (*n* = 2). The inverted funnel plot for the primary outcome did not suggest publication bias (Supplementary Fig. 3). Risk of bias and quality of evidence are summarized in the Supplementary Tables 6 and 7. Subgroup and sensitivity analyses did not show any significant differences (Supplementary Table 8 and Supplementary Fig. 4).

**Fig. 1:**
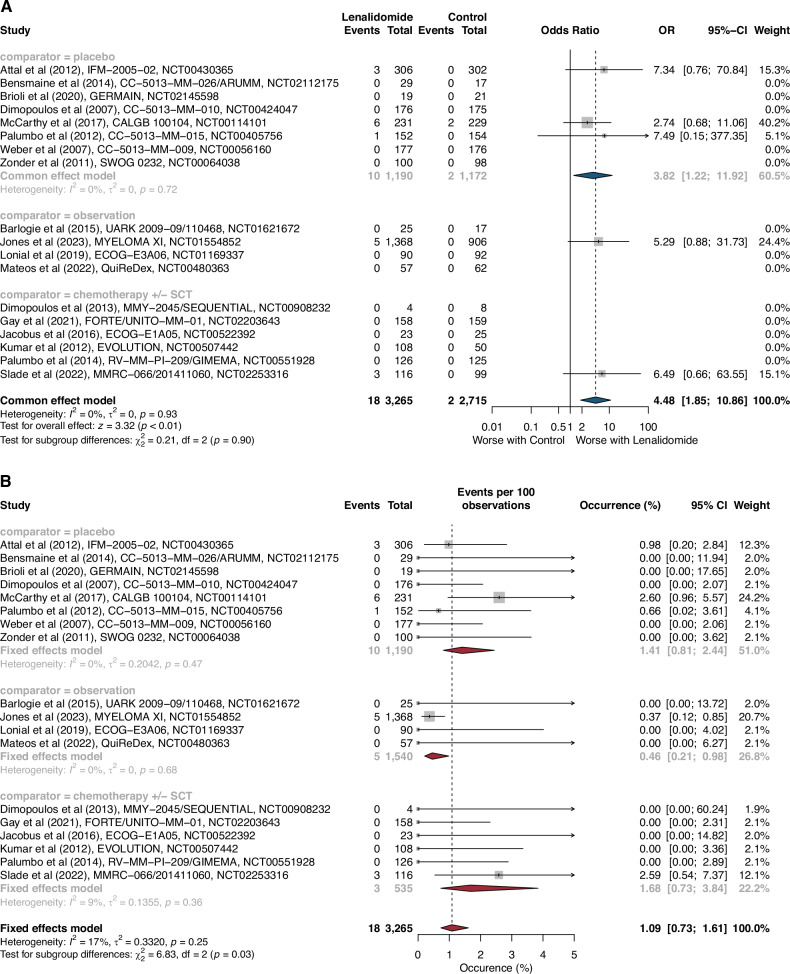
Risk and incidence of therapy-associated acute lymphoblastic leukemias in randomized controlled trials. Risk of t-ALL (**A**) with lenalidomide versus control in 18 RCTs. Events refers to the number of patients with acute lymphoblastic leukemia in regard to the total number of patients (intent-to-treat). Incidence of ALL (**B**) with lenalidomide treatment per 100 patients in 18 RCTs. CI Confidence Intervals, RCTs Randomized Controlled Trials, SCT Stem-Cell Transplantation, OR Odds Ratio.

On March 1, 2024, our search in VigiBase reported 2,155 cases of ALL associated to drugs. Among them, 269 (12%) cases were associated to lenalidomide therapy, including ALL with no other specification (*n* = 187), B-ALL (*n* = 81), and *BCR::ABL1* positive ALL (*n* = 1) (Supplementary Fig. 1B). Patient characteristics are summarized in Supplementary Table 9. Patients were male in 151 [60%] of 251 cases with available data. MM was the most common indication for lenalidomide therapy (241 [93%] of 260 patients with available data) followed by leukemia (seven [3%]), and lymphomas (three [1%]). Based on available data (*n* = 51 of 269 cases) the lenalidomide duration before ALL onset was 2.12 years (IQR 0.99–3.89), ranging from 0.01 to 11.17 years. The latency period of ALL from first exposure (*n* = 15 of 269 cases) was 3.08 years (IQR 2.69–3.23), ranging from 0·.1 to 5.84 years. After ALL onset, lenalidomide was withdrawn in 83 [93%] of the 89 cases with available data. Clinical outcomes were available in 100 cases. Forty-two patients (42%) did not recover from ALL, 34 patients (34%) were reported as recovered or recovering, and 24 patients (24%) resulted in death.

To our knowledge, this is the first study that highlights an increased risk and incidence of ALL after lenalidomide exposure in RCTs. Our findings are of clinical importance given that therapy-associated ALL is not a well-recognized entity, unlike therapy-related myeloid neoplasm (t-MN) [6]. At least three criteria are required to qualify a disease as therapy-related. The current definition is based on the patient’s medical history such as the occurrence of a secondary MN after exposure to a cytotoxic treatment for a previous neoplastic disease. The term “cytotoxic” is not limited to chemotherapy as the 2022 WHO classification includes PARP inhibitors, known to be associated with *TP53* mutated t-MN, as a qualifying criterion for myeloid neoplasm post cytotoxic treatment [7, 8]. Moreover, the imputability of a potential treatment has to be confirmed by epidemiological studies and scientifically explained. For example, t-MN is mainly driven by the selection of a preexisting clone related to a clonal haematopoiesis of indeterminate potential (CHIP), in the blood and bone marrow of a patient previously exposed to cytotoxic drugs, leading potentially to a t-MN [9]. The lack of clear pathophysiological evidence and the scarcity of the disease may explain the difficulty to consider ALL occurring after a prior malignancy as therapy related, but recent discoveries worked to decipher ALL leukemogenesis [6, 10]. Experimental in vivo and in vitro studies described the expansion of preleukemic TrP53 mutant haematopoietic stem cells (HSC) under lenalidomide exposure but not pomalidomide exposure [11]. This could explain the differential toxicity profile between these two drugs. The clinical efficacy of both lenalidomide and pomalidomide in MM relies on the binding to CRBN and induction of the proteasomal degradation of IKZF1 and IKZF3. However, only lenalidomide induces efficient degradation of CK1α [12]. Additionally, recent case series suggested a distinct genetic profile of lenalidomide-related ALL, as showed by literature review on Supplementary Table 10. Analysis of paired MM and ALL samples revealed that the *TP53* mutations were unrelated to the MM founding clone but could be present in HSC at the MM stage [13]. To close the loop, a study using single-cell analyses showed that low hypodiploid ALL can arise from a *TP53-*CHIP, a mechanism which was deemed to be restricted to myeloid neoplasms until very recently [14]. As *TP53*-CHIP is known to increase with age and exposure to anticancer therapies, these observations suggest that lenalidomide may contribute to the early phases of therapy-related ALL development [15]. This work also provides the largest description of clinical features of 269 lenalidomide-associated ALL reported to the WHO’s pharmacovigilance database. Interestingly, 7% of cases were reported in patients with non-MM diseases. This underlines that not only the drug exposure but also the genetic background and patient’s history could be involved in ALL development and need further investigations.

The main first limitation of our systematic review is related to the follow-up duration, which varies across trials and are not designed to capture ALL events (Supplementary Limitations). These differences might affect the estimation of risk and incidence of lenalidomide-associated ALL. Secondly, and inherently to pharmacovigilance studies conducted in VigiBase, we do not have access to the initial workup that led to the diagnosis of lenalidomide-associated ALL, such as clinical evaluation and laboratory test. Thus, we acknowledge that a definitive causal relationship between lenalidomide use and ALL onset cannot be formally ascertained.

In conclusion, we reported an increased risk and incidence of ALL in patients with MM who received lenalidomide. These findings could lead to consider lenalidomide-associated ALL as a specific entity and may help the physicians to adequately inform patients and better monitor this rare but serious long-term AE. Further studies are needed to refine the risk and incidence of lenalidomide-associated ALL in non-MM diseases, as well as to fix the duration of lenalidomide exposure and identify potential drug combinations that could increase this risk.

## Supplementary information

supplemental data

## Data Availability

Data from the safety meta-analysis are freely and publicly available on publications and clinical trial registries websites. At this time, data from VigiBase (the WHO global pharmacovigilance database of individual case safety reports) are only available for national pharmacovigilance centers and the Uppsala Monitoring Centre. Public access to overview statistics from VigiBase can be gained via the VigiAccess website.

## References

[1] Jones JR, Cairns DA, Menzies T, Pawlyn C, Davies FE, Sigsworth R, et al. Maintenance lenalidomide in newly diagnosed transplant eligible and non-eligible myeloma patients; profiling second primary malignancies in 4358 patients treated in the Myeloma XI Trial. EClinicalMedicine. 2023;62:102099. 37554123 10.1016/j.eclinm.2023.102099 PMC10404862

[2] Holstein SA, Jung S-H, Richardson PG, Hofmeister CC, Hurd DD, Hassoun H, et al. Updated analysis of CALGB (Alliance) 100104 assessing lenalidomide versus placebo maintenance after single autologous stem-cell transplantation for multiple myeloma: a randomised, double-blind, phase 3 trial. Lancet Haematol. 2017;4:e431–42. 28826616 10.1016/S2352-3026(17)30140-0 PMC5718627

[3] Zorzela L, Loke YK, Ioannidis JP, Golder S, Santaguida P, Altman DG, et al. PRISMA harms checklist: improving harms reporting in systematic reviews. BMJ. 2016;352:i157. 26830668 10.1136/bmj.i157

[4] Morton S, Murad M, O’Connor E, Lee C, Booth M, Vandermeer B et al. Quantitative Synthesis—An Update - Methods Guide for Effectiveness and Comparative Effectiveness Reviews - NCBI Bookshelf. 2018. https://www.ncbi.nlm.nih.gov/books/NBK519365/ (accessed 18 Mar2020).

[5] Faillie J-L, Ferrer P, Gouverneur A, Driot D, Berkemeyer S, Vidal X, et al. A new risk of bias checklist applicable to randomized trials, observational studies, and systematic reviews was developed and validated to be used for systematic reviews focusing on drug adverse events. J Clin Epidemiol. 2017;86:168–75. 28487158 10.1016/j.jclinepi.2017.04.023

[6] Riazat-Kesh YJRA, Mascarenhas J, Bar-Natan M. ‘Secondary’ acute lymphoblastic/lymphocytic leukemia - done playing second fiddle? Blood Rev. 2023;60:101070. 36894417 10.1016/j.blre.2023.101070

[7] Khoury JD, Solary E, Abla O, Akkari Y, Alaggio R, Apperley JF, et al. The 5th edition of the World Health Organization Classification of Haematolymphoid Tumours: Myeloid and Histiocytic/Dendritic Neoplasms. Leukemia. 2022;36:1703–19. 35732831 10.1038/s41375-022-01613-1 PMC9252913

[8] Marmouset V, Decroocq J, Garciaz S, Etienne G, Belhabri A, Bertoli S, et al. Therapy-related Myeloid Neoplasms Following PARP Inhibitors: Real-life Experience. Clin Cancer Res J Am Assoc Cancer Res. 2022;28:5211–20. 10.1158/1078-0432.CCR-22-1622 36201165

[9] Renneville A, Bernard E, Micol J-B. Therapy-related myelodysplastic syndromes in the genomics era. Bull Cancer (Paris). 2023;110:1129–40. 37391357 10.1016/j.bulcan.2023.02.022

[10] Saygin C, Zhang P, Stauber J, Aldoss I, Sperling AS, Weeks LD, et al. Acute lymphoblastic leukemia with myeloid mutations is a high-risk disease associated with clonal Hematopoiesis. Blood Cancer Discov. 2024;5:164–79. 38150184 10.1158/2643-3230.BCD-23-0106 PMC11061587

[11] Sperling AS, Guerra VA, Kennedy JA, Yan Y, Hsu JI, Wang F, et al. Lenalidomide promotes the development of TP53-mutated therapy-related myeloid neoplasms. Blood. 2022;140:1753–63. 35512188 10.1182/blood.2021014956 PMC9837415

[12] Krönke J, Fink EC, Hollenbach PW, MacBeth KJ, Hurst SN, Udeshi ND, et al. Lenalidomide induces ubiquitination and degradation of CK1α in del(5q) MDS. Nature. 2015;523:183–8. 26131937 10.1038/nature14610 PMC4853910

[13] Barnell EK, Skidmore ZL, Newcomer KF, Chavez M, Campbell KM, Cotto KC, et al. Distinct clonal identities of B-ALLs arising after lenolidomide therapy for multiple myeloma. Blood Adv. 2023;7:236–45. 36251745 10.1182/bloodadvances.2022007496 PMC9860439

[14] Kim R, Bergugnat H, Larcher L, Duchmann M, Passet M, Gachet S, et al. Adult low-hypodiploid acute lymphoblastic leukemia emerges from preleukemic TP53-mutant clonal Hematopoiesis. Blood Cancer Discov. 2023;4:134–49. 36630200 10.1158/2643-3230.BCD-22-0154 PMC9975768

[15] Bolton KL, Ptashkin RN, Gao T, Braunstein L, Devlin SM, Kelly D, et al. Cancer therapy shapes the fitness landscape of clonal hematopoiesis. Nat Genet. 2020;52:1219–26. 33106634 10.1038/s41588-020-00710-0 PMC7891089

