# Supplementary material for: Acute lymphoblastic leukemia in patients treated with lenalidomide for multiple myeloma: a safety meta-analysis of randomized controlled trials combined with a retrospective study of the WHO’s pharmacovigilance database
Source: Blood Cancer J. 2024 Oct 14;14(1):177. doi: 10.1038/s41408-024-01154-z (PMC11473840; doi:10.1038/s41408-024-01154-z)
Supplement: Supplementary file 1 — supplemental data [file 41408_2024_1154_MOESM1_ESM.docx]

**Supplemental Data**

[Supplementary Table 1. PRISMA Harms Checklist.. 2](#_Toc176259246)

[Supplementary Table 2. Search strategies for systematic review and pharmacovigilance study. 4](#_Toc176259247)

[Supplementary Methods. Data analysis related to systematic review (A) and pharmacovigilance study (B). 7](#_Toc176259248)

[Supplementary Figure 1. Study flow diagrams. 9](#_Toc176259249)

[Supplementary Table 3. Characteristics of randomized controlled trials. 10](#_Toc176259250)

[Supplementary Table 4. Selected sources provided the final safety dataset in 18 randomized controlled trials (last check: March 18, 2024). 14](#_Toc176259251)

[Supplementary Figure 2. Summary risk (A) and incidence (B) analysis of acute lymphoblastic leukaemia in randomized controlled trials. 17](#_Toc176259252)

[Supplementary Table 5. Characteristics of acute lymphoblastic leukaemia (ALL) cases reported in randomized controlled trials. 18](#_Toc176259253)

[Supplementary Figure 3. Funnel plot for publication bias of the primary and secondary outcomes. 20](#_Toc176259254)

[Supplementary Table 6. Summary (A) and individual (B) risk of bias of all randomized controlled trials (n=18). 21](#_Toc176259255)

[Supplementary Table 7. Quality of evidence with Grading of Recommendations Assessment, Development and Evaluation system (GRADE). 187](#_Toc176259256)

[Supplementary Table 8. Subgroup analyses on the risk of therapy-associated acute lymphoblastic leukaemia with lenalidomide versus placebo (A) or all controls (B). 189](#_Toc176259257)

[Supplementary Figure 4. Sensitivity analysis on the risk of therapy-associated acute lymphoblastic leukaemia with lenalidomide versus placebo in RCTs (n=8). 192](#_Toc176259258)

[Supplementary Table 9. Characteristics of patients diagnosed with acute lymphoblastic leukaemia following lenalidomide use from the WHO’s pharmacovigilance database.. 193](#_Toc176259259)

[Supplementary Table 10. Review of litterature associated to ALL post-lenalidomide in patients with multiple myeloma.. 195](#_Toc176259260)

[Supplementary Limitations of systematic review and pharmacovigilance study. 196](#_Toc176259261)

[Supplementary references 197](#_Toc176259262)

# Supplementary Table 1. PRISMA Harms Checklist. The PRISMA harms checklist contains additional items that must be used in any systematic review addressing harms, irrespective of whether harms are analysed alone or in association with benefits.

| Section/topic | Item | PRISMA checklist item | Recommendations for reporting PRISMA Harms in systematic reviews | Check if done |
| --- | --- | --- | --- | --- |
| **Title** |  |  |  |  |
| Title | 1 | Identify the report as a systematic review, meta-analysis, or both. | Specifically mention “harms” or other related terms, or the harm of interest in the review | **Check** |
| **Abstract** |  |  |  |  |
| Structured summary | 2 | Provide a structured summary including, as applicable: background; objectives; data sources; study eligibility criteria, participants, and interventions; study appraisal and synthesis methods; results; limitations; conclusions and implications of key findings; systematic review registration number. | Abstracts should report any analysis of harms undertaken in the review, if harms are a primary or secondary outcome. | **Check** |
| **Introduction** |  |  |  |  |
| Rationale | 3 | Describe the rationale for the review in the context of what is already known. | It should clearly describe in introduction or in methods section which events are considered harms and provide a clear rationale for the specific harm(s), condition(s), and patient group(s) included in the review. | **Check** |
| Objectives | 4 | Provide an explicit statement of questions being addressed with reference to participants, interventions, comparisons, outcomes, and study design (PICOS). | PICOS format should be specified, although in systematic reviews of harms the selection criteria for P, C, and O may be very broad (same intervention may have been used for heterogeneous indications in a diverse range of patients) | **Check** |
| **Methods** |  |  |  |  |
| Protocol and registration | 5 | Indicate if a review protocol exists, if and where it can be accessed (e.g., web address), and, if available, provide registration information including registration number. | No specific additional information is required for systematic reviews of harms. | **Check** |
| Eligibility criteria | 6 | Specify study characteristics (e.g., PICOS, length of follow-up) and report characteristics (e.g., years considered, language, publication status) used as criteria for eligibility, giving rationale. | Report how handled relevant studies (based on population and intervention) when the outcomes of interest were not reported.  Report choices for specific study designs and length of follow-up. | **Check** |
| Information sources | 7 | Describe all information sources (e.g., databases with dates of coverage, contact with study authors to identify additional studies) in the search and date last searched. | Report if only searched for published data, or also sought data from unpublished sources, from authors, drug manufacturers and regulatory agencies. If includes unpublished data, provide the source and the process of obtaining it. | **Check** |
| Search | 8 | Present full electronic search strategy for at least one database, including any limits used, such that it could be repeated. | If additional searches were used specifically to identify adverse events, authors should present the full search process so it can be replicated. | **Check** |
| Study selection | 9 | State the process for selecting studies (i.e., screening, eligibility, included in systematic review, and, if applicable, included in the meta-analysis). | If only included studies reporting on adverse events of interest, defined if screening was based on adverse event reporting in title/abstract or full text. If no harms reported in the text, report if any attempt was made to retrieve relevant data from authors. | **Check** |
| Data collection process | 10 | Describe method of data extraction from reports (e.g., piloted forms, independently, in duplicate) and any processes for obtaining and confirming data from investigators. | No specific additional information is required for systematic reviews of harms. | **Check** |
| Data items | 11 | List and define all variables for which data were sought (e.g., PICOS, funding sources) and any assumptions and simplifications made. | Report the definition of the harm and seriousness used by each included study (if applicable). Report if multiple events occurred in the same individuals, if this information is available. Consider if the harm may be related to factors associated with participants (e.g., age, sex, use of medications) or provider (e.g., years of practice, level of training). Specify if information was extracted and how it was used in subsequent results. Specify if extracted details regarding the specific methods used to capture harms (active/passive and timing of adverse event). | **Check** |
| Risk of bias in individual studies | 12 | Describe methods used for assessing risk of bias of individual studies (including specification of whether this was done at the study or outcome level), and how this information is to be used in any data synthesis. | The risk of bias assessment should be considered separately for outcomes of benefit and harms. | **Check** |
| Summary measures | 13 | State the principal summary measures (e.g., risk ratio, difference in means). | No specific additional information is required for systematic reviews of harms. | **Check** |

| Synthesis of results | 14 | Describe the methods of handling data and combining results of studies, if done, including measures of consistency (e.g., I^2^) for each meta-analysis. | Specify how zero events were handled, if relevant. | **Check** |
| --- | --- | --- | --- | --- |
| Risk of bias across studies | 15 | Specify any assessment of risk of bias that may affect the cumulative evidence (e.g., publication bias, selective reporting within studies). | Present the extent of missing information (studies without harms outcomes), any factors that may account for their absence, and whether these reasons may be related to the results. | **Check** |
| Additional analyses | 16 | Describe methods of additional analyses (e.g., sensitivity or subgroup analyses, meta-regression), if done, indicating which were prespecified. | Sensitivity analyses may be affected by different definitions, grading, and attribution of adverse events, as adverse events are typically infrequent or reported using heterogeneous classifications. Report the number of participants and studies included in each subgroup. | **Check** |
| **Results** |  |  |  |  |
| Study selection | 17 | Give numbers of studies screened, assessed for eligibility, and included in the review, with reasons for exclusions at each stage, ideally with a flow diagram. | If a review addresses both efficacy and harms, display a flow diagram specific for each (efficacy and harm). | **Check** |
| Study characteristics | 18 | For each study, present characteristics for which data were extracted (e.g., study size, PICOS, follow-up period) and provide the citations. | Define each harm addressed, how it was ascertained (e.g., patient report, active search), and over what time period.  Add additional characteristics to: “P” (population) patient risk factors that were considered as possibly affecting the risk of the harm outcome. “I” (intervention) professional expertise/skills if relevant (for example if the intervention is a procedure). “T” (time) timing of all harms assessments and the length of follow-up. | **Check** |
| Risk of bias within studies | 19 | Present data on risk of bias of each study and, if available, any outcome level assessment (see item 12). | Consider the possible sources of biases that could affect the specific harm under consideration within the review. Sample selection, dropouts and measurement of adverse events should be evaluated separately from the outcomes of benefit as described in item 12, above. | **Check** |
| Results of individual studies | 20 | For all outcomes considered (benefits or harms), present, for each study: (a) simple summary data for each intervention group (b) effect estimates and confidence intervals, ideally with a forest plot. | Report the actual numbers of adverse events in each study, separately for each intervention. | **Check** |
| Synthesis of results | 21 | Present results of each meta-analysis done, including confidence intervals and measures of consistency. | Describe any assessment of possible causality.  If included data from unpublished sources, report clearly the data source and the impact of these studies to the final systematic review. | **Check** |
| Risk of bias across studies | 22 | Present results of any assessment of risk of bias across studies (see item 15). | No specific additional information is required for systematic reviews of harms. See item 15 above. | **Check** |
| Additional analysis | 23 | Give results of additional analyses, if done (e.g., sensitivity or subgroup analyses, meta-regression (see item 16)). | No specific additional information is required for systematic reviews of harms. | **Check** |
| **Discussion** |  |  |  |  |
| Summary of evidence | 24 | Summarise the main findings including the strength of evidence for each main outcome; consider their relevance to key groups (e.g., healthcare providers, users, and policy makers). | No specific additional information is required for systematic reviews of harms. | **Check** |
| Limitations | 25 | Discuss limitations at study and outcome level (e.g., risk of bias), and at review level (e.g., incomplete retrieval of identified research, reporting bias). | Recognise possible limitations of meta-analysis for rare adverse events (i.e., quality and quantity of data), issues noted previously related to collection and reporting. | **Check** |
| Conclusions | 26 | Provide a general interpretation of the results in the context of other evidence, and implications for future research. | State conclusions in coherence with the review findings. When adverse events were not identified we caution against the conclusion that the intervention is “safe,” when, in reality, its safety remains unknown. | **Check** |
| **Funding** |  |  |  |  |
| Funding | 27 | Describe sources of funding for the systematic review and other support (e.g., supply of data); role of funders for the systematic review. | No specific additional information is required for systematic reviews of harms. | **Check** |

# Supplementary Table 2. Search strategies for systematic review and pharmacovigilance study. Researches for systematic review were conducted through MEDLINE (A), Cochrane CENTRAL (B) both in November 15, 2023 followed by https://www.ClinicalTrials.gov and https://www.ClinicalTrialsRegister.eu/ registry websites both in November 17, 2023 (C and D). Researches for pharmacovigilance study were conducted up to March 1, 2024 (E).

In the first part of this study, we conducted a systematic review and meta-analysis of RCTs according to the PRISMA harms checklist^1^. Eligibility criteria for study inclusion were RCTs comparing lenalidomide (also known as CC-5013 or L04AX04^2^) versus control (placebo or open-label) in adult patients (age ≥18 years) with MM. Based on a previously published stepwise method^3^, we first extracted all ALL cases in RCTs that studied lenalidomide from ClinicalTrials.gov and ClinicalTrialsRegister.eu registry websites. If ALL were not available from ClinicalTrials.gov or ClinicalTrialsRegister.eu registry websites, events were extracted from published RCTs. When ALL cases were available both from clinical trial registry websites and publications, the most informative source was selected. Lastly, if safety data were not available, the corresponding author or sponsor of the RCTs was contacted by e-mail (P-MM, J-BM). RCTs without safety data of interest were excluded from summary estimates. RCTs were identified by reviewing the literature in MEDLINE and the Cochrane Central Register of Controlled Trials (CENTRAL) both until November 15, 2023, followed by the ClinicalTrials.gov and ClinicalTrialsRegister.eu registry websites both until November 17, 2023.

The search strategy included specific medical subject heading terms, as well as and free-text words, such as “randomized controlled trial”, “placebo”, “lenalidomide and MM” (Supplementary Table 2)**.** To identify newly published studies or available safety data posted on clinical trials registry websites that might affect the findings of the review, a last check was performed on March 18, 2024. We excluded systematic reviews, preclinical studies, case reports, retrospective studies, observational studies, single-arm studies, RCTs with lenalidomide in all arms, RCTs with thalidomide or pomalidomide as comparator group, non-randomised trials and non-English citations. Two authors (P-MM and SKH) independently screened citations for eligibility of data extraction and consulted a third author (J-BM) to resolve potential disagreements. The study protocol was registered online in the International Prospective Register of Systematic Reviews (PROSPERO, CRD42024495677).

**A**

| Search | Query | Hits |
| --- | --- | --- |
| 15 | #13 AND #14 | 2,182 |
| 14 | multiple myeloma [MeSH Terms] | 48,152 |
| 13 | #11 AND #12 | 3,889 |
| 12 | lenalidomide [Title/Abstract] | 5,649 |
| 11 | #9 NOT #10 | 5,144,594 |
| 10 | (animals [MeSH Terms]) NOT (humans [MeSH Terms]) | 5,168,845 |
| 9 | #1 OR #2 OR #3 OR #4 OR #5 OR #6 OR #7 OR #8 | 5,882,404 |
| 8 | groups [Title/Abstract] | 2,627,586 |
| 7 | trial [Title/Abstract] | 790,556 |
| 6 | randomly [Title/Abstract] | 421,399 |
| 5 | Drug Therapy [MeSH Subheading] | 2,637,974 |
| 4 | placebo [Title/Abstract] | 250,028 |
| 3 | randomized [Title/Abstract] | 681,545 |
| 2 | controlled clinical trial [Publication Type] | 694,933 |
| 1 | randomized controlled trial [Publication Type] | 604,395 |

**B**

| Search | Query | Hits |
| --- | --- | --- |
| 17 | (#15) AND #16 | 701 |
| 16 | MeSH descriptor: [Multiple Myeloma] explode all trees | 2,794 |
| 15 | (#13) AND #14 | 2,295 |
| 14 | (lenalidomide):ti,ab,kw | 2,575 |
| 13 | (#9) NOT #12 | 1,534,426 |
| 12 | (#10) NOT #11 | 2,951 |
| 11 | MeSH descriptor: [Humans] explode all trees | 774,264 |
| 10 | MeSH descriptor: [Animals] explode all trees | 777,215 |
| 9 | (((((((#1) OR #2) OR #3) OR #4) OR #5) OR #6) OR #7) OR #8 | 1,537,335 |
| 8 | ("groups"):ti,ab,kw | 589,288 |
| 7 | ("trial"):ti,ab,kw | 1,029,309 |
| 6 | ("randomly"):ti,ab,kw | 312,339 |
| 5 | MeSH descriptor: [Drug Therapy] explode all trees | 180,963 |
| 4 | ("placebo"):ti,ab,kw | 364,265 |
| 3 | ("randomized"):ti,ab,kw | 1,111,235 |
| 2 | ("controlled clinical trial"):ti,ab,kw | 174,837 |
| 1 | ("randomized controlled trial"):ti,ab,kw | 626,223 |

**C**

| Step 1 | Connect to: <https://clinicaltrials.gov/> |
| --- | --- |
| Step 2 | Fill the field ‘Other terms’ with following key words: ‘lenalidomide AND randomized’ |
| Step 3 | Select ‘Phase 2’ and ‘Phase 3’ in ‘Study Phase’ section |
| Step 4 | Select ‘Interventional’ in ‘Study type’ section |
| Step 5 | Select ‘All results’ in ‘Study Results’ section |
| Step 6 | Select ‘Search’ and screen each study found |

**D**

| Step 1 | Connect to: <https://www.clinicaltrialsregister.eu/ctr-search/search> |
| --- | --- |
| Step 2 | Fill the field ‘Please enter search term’ with following key words: ‘lenalidomide AND randomized’ |
| Step 3 | Select ‘Search’ and screen each study found |

**E**

In the second part of the study, we described ALL cases reported in VigiBase, the WHO’s pharmacovigilance database managed by the Uppsala Monitoring Centre (Uppsala, Sweden). To identify them, we used the Medical Dictionary for Regulatory Activities (version 26.1) set of terms gathered by the High-Level Term (HLT) “Leukemias acute lymphocytic”. Among this HLT, a preplanned selection of the following preferred terms (PT) was conducted (J-BM): “acute lymphocytic leukemia, B precursor type acute leukemia, B-cell type acute leukemia, Mature B-cell type acute leukemia, Philadelphia positive acute lymphocytic leukemia, T-cell type acute leukemia”. Only ALL cases notified as suspected to be caused by lenalidomide were analysed with the Extract Case Level function provided by VigiBase. The study protocol of our pharmacovigilance study entitled ‘Acute Lymphoblastic Leukemia Related to Lenalidomide (LenALL)’ was registered on ClinicalTrials.gov, NCT06251648. The use of confidential, electronically processed de-identified patient data was approved by the Caen University Hospital Research Ethics Committee (#2646).

# Supplementary Methods. Data analysis related to systematic review (A) and pharmacovigilance study (B).

**A**

For the studies included in our systematic review, the following available data were extracted and recorded in Microsoft Excel: lenalidomide or control regimens, previous line of chemotherapy and stem-cell transplantation (SCT) before randomisation, previous exposure to cytotoxic treatment known to increase the risk of ALL, allocation, intervention model, masking, age (at baseline and at ALL onset), median follow-up, duration of lenalidomide treatment, lenalidomide setting use, MM disease, early stopped status of study, intent-to-treat patients, (online) publication dates, ALL events reported in lenalidomide and control arms, and time to death following ALL onset. All these data were carefully double-checked (P-MM, SKH) to avoid overlapping.

The **primary outcome** was the summary risk of ALL associated to lenalidomide versus placebo in RCTs in patients with MM. The **secondary outcomes** were: (i) the summary risk of ALL associated to lenalidomide versus control treatment (placebo and open-label) in RCTs, (ii) the summary incidence of ALL cases associated to lenalidomide or placebo treatment in RCTs and, (iii) the summary incidence of ALL cases associated to lenalidomide or treatment control (placebo and open-label) in RCTs. In case of multi-arm RCTs, the lenalidomide groups or the control groups were pooled.

To explore possible sources of heterogeneity or inconsistency in placebo RCTs in the primary analysis, we did prespecified subgroup analyses according to patient’s age at baseline, MM diagnosis previous exposure to lenalidomide before randomisation, previous exposure to cytotoxic drugs known to increase the risk of ALL (based on Anatomical Therapeutic Chemical (ATC) classification system: alkylating agents L01A^4^, anthracyclines and related substances L01DB^5^), previous SCT before randomisation, lenalidomide starting dose, lenalidomide regimen, lenalidomide setting use (eg, induction), duration of lenalidomide administration, follow-up length, study stopped early. As previously reported by Saleem *et al*., median cumulative lenalidomide dose was not available in most studies and was not included in the preplanned subgroup analyses.^6^ Secondly, subgroup analysis were repeated including all RCTs. During the data extraction process, we identified a conflicting number of ALL reported between the main publication (selected to assess primary outcome) and the supplementary appendix for the lenalidomide arm of the CALGB 100104 trial.^7^ To assess the robustness of primary outcome, we did a post-hoc sensitivity analysis by recalculating the combined Peto OR with data reported in supplementary data.

One author (P-MM) evaluated the risk of bias in individual studies using the Pharmacoepidemiological Research on Outcomes of Therapeutics by a European Consortium checklist tool^8^. In case of disagreements, a second author (J-BM) was consulted. Publication bias was assessed graphically by constructing a funnel plot (P-MM). Quality of evidence was assessed with the Grading of Recommendations Assessment, Development and Evaluation (GRADE) system (P-MM).

We performed a fixed-effect meta-analysis to compute Peto odds ratios (ORs) with 95% CIs, a dedicated method for binary studies with rare events (<1%).^9^ As ALL were rare events, we assumed OR as a measure of the risk.^10,11^ The summary incidences of ALL associated to lenalidomide or control (placebo, open-label) were computed with the logit transformation and inverse variance weighting. We assessed between-study heterogeneity using the inconsistency index *I*² statistic and the χ² test with its P-value. An *I*² value of greater than 50% denoted a substantial between-study heterogeneity. A χ² P-value of less than 0·10 denoted a significant heterogeneity^12^. Estimates were computed with R (version 4.3.1, including package meta) and presented in forest plots. A two-sided P-value < 0·05 in Z-tests (for overall effect) or χ² tests (for overall subgroup comparison) in all estimates was considered statistically significant. Median latency period, defined as the interval between randomization and diagnosis of ALL, was calculated with data from RCTs and expressed in years with interquartile range (IQR) and range.

**B**

In the second part of the study, the following pharmacovigilance data were extracted and recorded in R (CD): reporting year, type of report, sex, age at onset, geographical location, reporter, lenalidomide characteristics (indication, start and end date, treatment modifications), ALL characteristics (type, onset date, latency period, seriousness), outcome, co-suspected anticancer drugs involved in ALL onset, and co-reported adverse events (AEs) ≥5%. Median lenalidomide duration, defined as the sum of all lenalidomide exposure durations for each case, was computed with available data and expressed in years with interquartile range (IQR) and range. Median latency period, defined as the interval between lenalidomide initiation and diagnosis of ALL, was computed with available data and expressed in years with IQR and range. Seriousness was defined by Cancer Therapy Evaluation Program Adverse Event Reporting System (CTEP-AERS) as any adverse drug event (experience) occurring at any dose that results in any of the following outcomes: death, a life-threatening adverse drug experience, an inpatient (prolonged) hospitalization, a persistent or significant incapacity or substantial disruption of the ability to conduct normal life functions, a congenital anomaly or birth defect, or important medical event based on medical judgment.^13^

# Supplementary Figure 1. Study flow diagrams.

(A) PRISMA diagram of our systematic review and safety meta-analysis of RCTs on lenalidomide in adult patients with multiple myeloma, available in MEDLINE, Cochrane CENTRAL, the ClinicalTrials.gov and the ClinicalTrialsRegister.eu registries on November 17, 2023, with ongoing surveillance until March 18, 2024. (B) Flow diagram of our observational, retrospective, pharmacovigilance study of cases of acute lymphoblastic leukaemias associated to lenalidomide treatment reported in VigiBase up to March 1, 2024. RCTs=randomized controlled trials. CENTRAL=Central Register of Controlled Trials. *Studies were searched in MEDLINE (n=2182) followed by Cochrane CENTRAL (n=701), ClinicalTrials.gov (n=43) and ClinicalTrialsRegister.eu (n=56). † One RCT (2010-021557-40) without safety data associated to acute lymphoblastic leukaemia (ALL) from publications, corresponding authors or sponsors, and clinical trial registries.


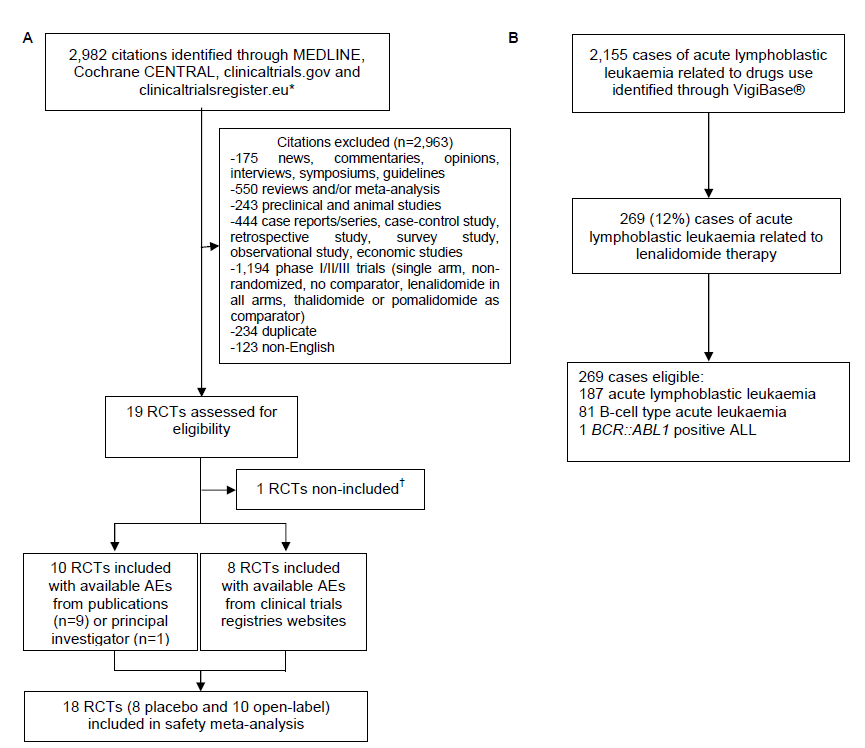


# **Supplementary Table 3. Characteristics of randomized controlled trials.** NA: Not available. SCT: stem-cell transplantation. VD(C)R: bortezomib, dexamethasone, (cyclophosphamide), lenalidomide. *Phase of interest for the meta-analysis. ^†^Changed to 10mg/day following protocol amendment (Sept 14, 2011) ^‡^pooled VDCR+VDR vs VDC+VDC-modified ^¶^

Among 18 RCTs, 8 were placebo RCTs^7,14–20^, 10 were open-label RCTs (6 were chemotherapy ± SCT-based RCTs^21–26^ and 4 were observation RCTs^27–30^).

| **Study** | **Patients groups (n= 5,980), intention-to-treat** | **Comparator** | **Multiple Myeloma** | **Previous lines of**  **chemotherapy** | **Interventional model** | **masking** | **Median or mean age, years** | **Median follow-up, months or years** |
| --- | --- | --- | --- | --- | --- | --- | --- | --- |
| Attal et al (2012), IFM-2005-02, NCT00430365 | Lenalidomide 25mg/day (n=306) consolidation followed by lenalidomide 10 mg/day maintenance versus lenalidomide 25mg/day (n=302) consolidation followed by placebo maintenance | placebo | newly diagnosed | One (vincristine, doxorubicin, dexamethasone or bortezomib, dexamethasone induction regimens) followed by hematopoietic SCT | parallel assignment | Double | 55 years (lenalidomide group) versus 55 years (placebo group) | 45 months |
| Barlogie et al (2015), UARK 2009-09/110468, NCT01621672 | Lenalidomide 10mg/day (n=25) versus observation (n=17) | observation | newly diagnosed | NA (bortezomib-, thalidomide- or lenalidomide-, and dexamethasone-based regimens) | parallel assignment | Open label | NA | NA |
| Bensmaine et al (2014), CC-5013-MM-026/ARUMM, NCT02112175 | Lenalidomide 10 mg/day (n=29) versus placebo (n=17) | placebo | newly diagnosed | One (melphalan plus prednisone plus bortezomib regimen) | parallel assignment | Double | 73·1 years (lenalidomide group) versus 72·9 years (placebo group) | NA |
| Brioli et al (2020), GERMAIN, NCT02145598 | Lenalidomide 10 mg/day (n=19) versus placebo (n=21) | placebo | newly diagnosed | One (melphalan plus prednisone plus bortezomib regimen) | parallel assignment | Quadruple | NA | 12·9 months |
| Dimopoulos et al (2007), CC-5013-MM-010, NCT00424047 | Lenalidomide 25 mg/day plus dexamethasone (n=176) versus placebo plus dexamethasone (n=175) | placebo | relapsed or refractory | One to two (bortezomib-, thalidomide-, glucocorticoids- and SCT-based regimens) | parallel assignment | Quadruple | 63 years (lenalidomide group) versus 64 years (placebo group) | 16·4 months |
| Dimopoulos et al (2013), MMY-2045/SEQUENTIAL, NCT00908232 | Lenalidomide 10mg/day plus bortezomib plus dexamethasone (n=4) versus cyclophosphamide plus bortezomib plus dexamethasone (n=8) | chemotherapy | relapsed or refractory | NA (bortezomib-, thalidomide- or lenalidomide-, melphalan-, cyclophosphamide-, vincristine-, doxorubicine-, and glucocorticoids-based regimens) followed (or not) by SCT | parallel assignment | Open label | 63 years | 16·9 months |
| Gay et al (2021), FORTE/UNITO-MM-01, NCT02203643 | **First randomization* (induction, intensification, consolidation)**: lenalidomide 25mg/day plus carfilzomib plus dexamethasone followed by ASCT (n=158) versus lenalidomide 25mg/day plus carfilzomib plus dexamethasone (n=156) versus cyclophosphamide plus carfilzomib plus dexamethasone followed by ASCT (n=159)  **Second randomization (maintenance)**: lenalidomide 10mg/day plus carfilzomib (n=178) versus lenalidomide 10mg/day (n=178) | chemotherapy | newly diagnosed | Naive | parallel assignment | Open label | 57 years in three arms (first randomization) | 50·9 months (following first randomization) 37·3 months (following second randomization) |
| Jones et al (2023), MYELOMA XI, NCT01554852 | Lenalidomide^†^ 25* mg/day (n=1368) versus observation (n=906) | observation | newly diagnosed | One (cyclophosphamide, thalidomide or lenalidomide, and dexamethasone or carfilzomib, cyclophosphamide, lenalidomide, and dexamethasone regimens) followed (or not) by SCT | parallel assignment | Open label | 66 years (lenalidomide group) versus 66 years (observation group) | 46 and 55 months, for transplant eligible and transplant non-eligible patients, respectively |
| Jacobus et al (2016), ECOG-E1A05, NCT00522392 | Lenalidomide 15mg/day plus bortezomib plus dexamethasone (n=23) versus bortezomib plus dexamethasone (n=25) | chemotherapy | newly diagnosed | One (lenalidomide-, dexamethasone-based regimens) | parallel assignment | Open label | 64 years (lenalidomide group) versus 65 years (chemotherapy group) | 72 months |
| Kumar et al (2012), EVOLUTION, NCT00507442 | Lenalidomide 25 mg/day plus cyclophosphamide plus bortezomib plus dexamethasone induction (n=66) or lenalidomide 15 mg/day plus bortezomib plus dexamethasone induction (n=42) versus cyclophosphamide plus bortezomib plus dexamethasone induction (n=33) or cyclophosphamide-modified plus bortezomib plus dexamethasone induction (n=17)· Induction therapies were followed by bortezomib maintenance regimen^‡^ | chemotherapy | newly diagnosed | Naive | parallel assignment | Open label | 61·5 years (VDCR group) or 60 years (VDR group) versus 62 years (VDC group) or 63 years (VDC-modified group) | 20 months (VDCR group) or 20 months (VDR group) versus 22 months (VDC group) or 15 months (VDC-modified group) |
| Lonial et al (2019), ECOG-E3A06, NCT01169337 | Lenalidomide 10 mg/day (n=90) versus observation (n=92) | observation | smoldering | Naive | parallel assignment | Open label | 63 years (lenalidomide group) versus 64 years (observation group) | 35 months |
| Mateos et al (2022), QuiReDex, NCT00480363 | Lenalidomide 10mg/day plus dexamethasone (n=57) induction followed by lenalidomide maintenance versus observation (n=62) | observation | smoldering | Naive | parallel assignment | Open label | 63 years (lenalidomide group) versus 69 years (observation group) | 12,5 years |
| McCarthy et al (2017), CALGB 100104, NCT00114101 | Lenalidomide 10 mg/day (n=231) versus placebo (n=229) | placebo | newly diagnosed | One (bortezomib-, lenalidomide-, thalidomide-, glucocorticoids-based regimens) followed by single autologous hematopoietic SCT | parallel assignment | Double | 59 years (lenalidomide group) versus 58 years (placebo group) | 91 months |
| Palumbo et al (2012), CC-5013-MM-015, NCT00405756 | Lenalidomide plus melphalan-prednisone (n=152) induction followed by lenalidomide maintenance versus placebo plus melphalan-prednisone (n=154) followed by placebo maintenance | placebo | newly diagnosed | Naive | parallel assignment | Quadruple | 71 years (lenalidomide group) versus 71 years (placebo group) | 30 months |
| Palumbo et al (2014), RV-MM-PI-209/GIMEMA, NCT00551928 | **First randomization (consolidation)**: lenalidomide 10mg/day plus melphalan plus prednisone (n=132) versus high-dose melphalan plus autologous stem-cell transplantation (n=141) **Second randomization (maintenance)**: lenalidomide 10mg/day (n=126) versus no maintenance (n=125) | chemotherapy | newly diagnosed | One induction line (lenalidomide and dexamethasone regimen) followed by mobilisation (cyclophosphamide regimen) | parallel assignment | Open label | 58 years | 51·2 months |
| Slade et al (2022), MMRC-066/201411060, NCT02253316 | Lenalidomide 10mg/day (n=116) versus ixazomib (n=99) | chemotherapy | newly diagnosed | One induction line (proteasome inhibitors-, lenalidomide-, cyclophosphamide-, daratumumab-based regimens followed by SCT) One consolidation line (ixazomib, lenalidomide, and dexamethasone regimen) | parallel assignment | Open label | 58 years (lenalidomide group) versus 56·5 years (chemotherapy group) | 26·5 months |
| Weber et al (2007), CC-5013-MM-009, NCT00056160 | Lenalidomide 25 mg/day plus dexamethasone (n=177) versus placebo plus dexamethasone (n=176) | placebo | relapsed or refractory | One to two (bortezomib-, thalidomide-, glucocorticoids- and SCT-based regimens) | parallel assignment | Quadruple | 64 years (lenalidomide group) versus 62 years (placebo group) | 26·2 months (lenalidomide group) versus 12·9 months (placebo group) |
| Zonder et al (2011), SWOG 0232, NCT00064038 | Lenalidomide 25 mg/day plus dexamethasone (n=100) versus placebo plus dexamethasone (n=98) | placebo | newly diagnosed | Naive | parallel assignment | Triple | 64·9 years (lenalidomide group) versus 63·1 years (placebo group) | 45·4 months |

# Supplementary Table 4. Selected sources provided the final safety dataset in 18 randomized controlled trials (last check: March 18, 2024). Green cells denote final source selection. ALL=Acute lymphoblastic leukaemia.

| **study** | **publication year** | **publication, ALL event** | **Online clinicaltrials.gov registry** | **Last safety posted** | **ALL event** | **Online clinicaltrialsregister.eu trial registry** | **Last safety posted** | **ALL event** | **Principal investigator** |
| --- | --- | --- | --- | --- | --- | --- | --- | --- | --- |
| Attal et al (2012), IFM-2005-02, NCT00430365 | May 10, 2012 | 3 vs 0 | NCT00430365 | **no** | **NA** | **NA** | **NA** | **NA** | - |
| Barlogie et al (2015), UARK 2009-09/110468, NCT01621672 | **no publication** | **NA** | NCT01621672 | 2015-08-12 | 0 vs 0 | **no** | **no** | **NA** | - |
| Bensmaine et al (2014), CC-5013-MM-026/ARUMM, NCT02112175 | **no publication** | **NA** | NCT02112175 | **no** | **NA** | 2013-001729-26 | 2021-04-29 | 0 vs 0 | - |
| Brioli et al (2020), GERMAIN, NCT02145598 | December 2, 2019 | 0 vs 0 | NCT02145598 | **no** | **NA** | 2012-003023-38 | **no** | **NA** | - |
| Dimopoulos et al (2007), CC-5013-MM-010, NCT00424047 | November 22, 2007 | not reported | NCT00424047 | 2015-3-3 | 0 vs 0 | **NA** | **NA** | **NA** | - |
| Dimopoulos et al (2013), MMY-2045/SEQUENTIAL, NCT00908232 | May 14, 2013 | not reported | NCT00908232 | 2015-01-14 | 0 vs 0 | 2007-001462-33 | **no** | **NA** | - |
| Gay et al (2021), FORTE/UNITO-MM-01, NCT02203643 | November 11, 2021 | 0 vs 0 | NCT02203643 | **no** | **NA** | 2014-000782-53 | **no** | **NA** | - |
| Jones et al (2023), MYELOMA XI, NCT01554852 | July 27, 2023 | 5 vs 0 | NCT01554852 | **no** | **NA** | 2009-010956-93 | **no** | **NA** | - |
| Jacobus et al (2016), ECOG-E1A05, NCT00522392 | July 29, 2016 | not reported | NCT00522392 | 2015-6-23 | not reported | **no** | **no** | **NA** | principal investigator  0 vs 0 |
| Kumar et al (2012), EVOLUTION, NCT00507442 | March 15, 2012 | not reported | NCT00507442 | 2012-4-12 | 0 vs 0 | **no** | **no** | **NA** | - |
| Lonial et al (2019), ECOG-E3A06, NCT01169337 | yes, 25-10-2020 | 0 vs 0 | NCT01169337 | 2021-4-29 | not reported | 2012-000750-66 | **no** | **NA** | - |
| Mateos et al (2022), QuiReDex, NCT00480363 | September 5, 2022 | 0 vs 0 | NCT00480363 | **no** | **NA** | 2007-000649-36 | **no** | **NA** | - |
| McCarthy et al (2012), CALGB 100104, NCT00114101 | August 17, 2017 | 6 vs 2 (sup data : 5 vs 2) | NCT00114101 | 2013-05-28 | not reported | **NA** | **NA** | **NA** | - |
| Palumbo et al (2012), CC-5013-MM-015, NCT00405756 | May 10, 2012 | 1 vs 0 | NCT00405756 | 2012-4-16 | not reported | 2006-001865-41 | 2017-4-29 | 1 vs 0 | - |
| Palumbo et al (2014), RV-MM-PI-209/GIMEMA, NCT00551928 | September 4, 2014 | 0 vs 0 | NCT00551928 | **no** | **NA** | 2007-001610-16 | **no** | **NA** | - |
| Slade et al (2022), MMRC-066/201411060, NCT02253316 | September 16, 2022 | not reported | NCT02253316 | 2022-09-03 | 3 vs 0 | **no** | **no** | **NA** | - |
| Weber et al (2007), CC-5013-MM-009, NCT00056160 | November 22, 2007 | not reported | NCT00056160 | 2010-3-10 | 0 vs 0 | **NA** | **NA** | **NA** | - |
| Zonder et al (2011), SWOG 0232, NCT00064038 | [Publication](https://haematologica.org/article/view/5980)  [(P-167 - 13th International Myeloma Workshop, Paris, France, May 3–6, 2011)](https://haematologica.org/article/view/5980) | 0 vs 0 | NCT00064038 | 2013-6-12 | not reported | **NA** | **NA** | **NA** | - |

# Supplementary Figure 2. Summary risk (A) and incidence (B) analysis of acute lymphoblastic leukaemia in randomized controlled trials.

Events refers to the number of patients with acute lymphoblastic leukaemia in regard to the total number of patients (intent-to-treat). CI: Confidence Intervals. SCT: Stem-cell transplantation.

**A risk in lenalidomide versus placebo RCTs**


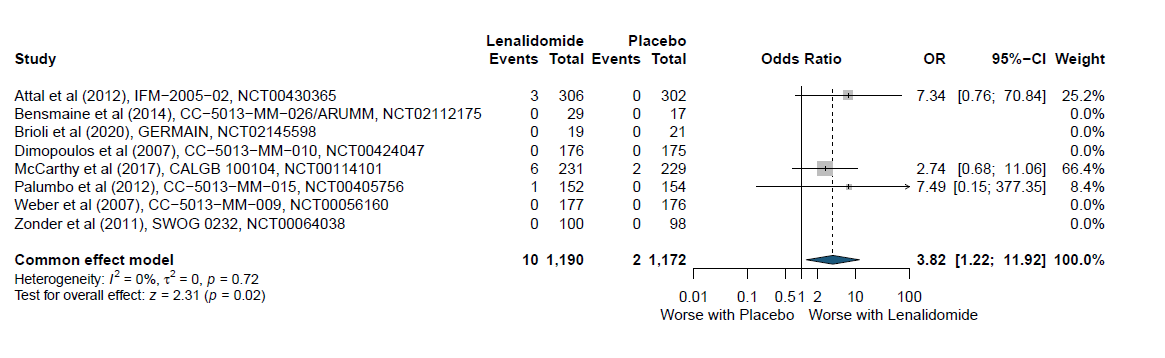


**B incidence with control groups per 100 patients**


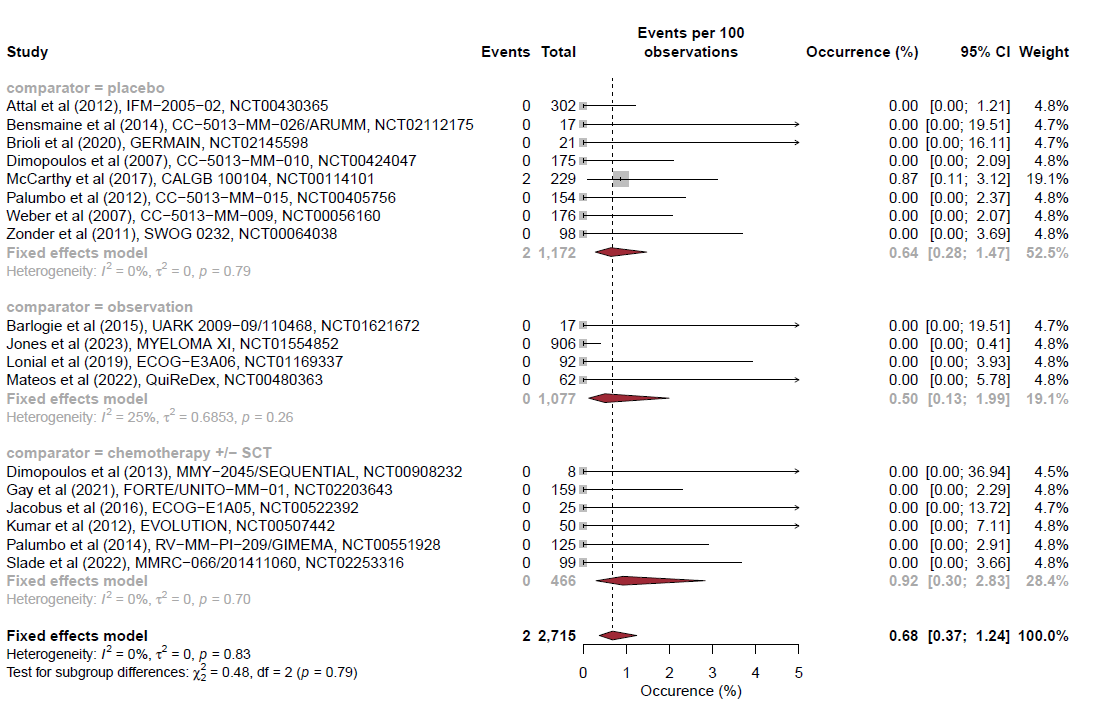


# Supplementary Table 5. Characteristics of acute lymphoblastic leukaemia (ALL) cases reported in randomized controlled trials.

ASCT: autologous stem-cell transplantation. B- (or T-) ALL: B (or T) lymphocyte acute lymphoblastic leukaemia. CO: crossover from placebo to LEN. LEN: lenalidomide. MPR-R: melphalan-prednisone-lenalidomide induction followed by lenalidomide maintenance. NA: not available. T[N]E: transplant [non] eligible.

| **Study** | **Cases** | **ALL type** | **Arm** | **Multiple myeloma induction therapy** | **Sex** | **Age at ALL, years** | **Cause of death** | **Time on LEN (y)** | **Time from randomisation to ALL onset (years)** | **Time from ALL to death (months)** | **ALL cytogenetics** | **Comment** |
| --- | --- | --- | --- | --- | --- | --- | --- | --- | --- | --- | --- | --- |
| McCarthy et al (2017), CALGB 100104, NCT00114101 | 1 | B-ALL | **CO** | thalidomide, dexamethasone | M | 64.5 | ALL | 4.4 | 5.3 | 11.5 | 20q- | induction + ASCT before randomization |
|  | 2 | B-ALL | **CO** | lenalidomide, dexamethasone | M | 66.2 | Alive | 4.5 | 4.8 | - | NA |  |
|  | 3 | B-ALL | LEN | thalidomide, dexamethasone | M | 50.4 | ALL | 2.1 | 2.1 | 7.7 | Normal |  |
|  | 4 | B-ALL | LEN | bortezomib, dexamethasone | M | 62.6 | Alive | 8.0 | 8.1 | - | *+8, +10, +21, del20, gain RUNX1 |  |
|  | 5 | B-ALL | LEN | thalidomide, dexamethasone | M | 61.4 | Alive | 3.6 | 4.0 | - | NA |  |
|  | 6 | B-ALL | LEN | thalidomide, dexamethasone, liposomal doxorubicin | M | 68.3 | Alive | 4.2 | 5.1 | - | NA |  |
|  | 7 | B-ALL | LEN | vincristine, liposomal doxorubicin, dexamethasone, thalidomide | F | 61.4 | Alive | 9.3 | 9.4 | - | Hyperdiploid with 20q- |  |
|  | 8 | NA | LEN | NA | NA | NA | NA | NA | NA | NA | NA |  |
| Palumbo et al (2012), CC-5013-MM-015, NCT00405756 | 9 | T-ALL | LEN | melphalan-prednisone | NA | NA | NA | NA | NA | NA | NA | MPR-R arm |
| Attal et al (2012), IFM-2005-02, NCT00430365 | 10 | ALL | LEN | NA | NA | NA | NA | NA | NA | NA | NA |  |
|  | 11 | ALL | LEN | NA | NA | NA | NA | NA | NA | NA | NA |  |
|  | 12 | ALL | LEN | NA | NA | NA | NA | NA | NA | NA | NA |  |
| Jones et al (2023), MYELOMA XI, NCT01554852 | 13 | B-ALL | LEN | TE Pathway: cyclophosphamide, thalidomide, and dexamethasone induction | NA | NA | NA | NA | NA | NA | NA |  |
|  | 14 | B-ALL | LEN | TE Pathway: CTD induction | NA | NA | NA | NA | NA | NA | NA |  |
|  | 15 | T-ALL | LEN | TE Pathway: cyclophosphamide, thalidomide, and dexamethasone induction | NA | NA | NA | NA | NA | NA | NA |  |
|  | 16 | B-ALL | LEN | TNE Pathway: cyclophosphamide, lenalidomide, dexamethasone attenuated induction | NA | NA | NA | NA | NA | NA | NA |  |
|  | 17 | B-ALL | LEN | TNE Pathway: cyclophosphamide, lenalidomide, dexamethasone attenuated induction | NA | NA | NA | NA | NA | NA | NA |  |
| Slade et al (2022), MMRC-066/201411060, NCT02253316 | 18 | ALL | LEN | NA | NA | NA | NA | NA | NA | NA | NA |  |
|  | 19 | ALL | LEN | NA | NA | NA | NA | NA | NA | NA | NA |  |
|  | 20 | ALL | LEN | NA | NA | NA | NA | NA | NA | NA | NA |  |

# Supplementary Figure 3. Funnel plot for publication bias of the primary and secondary outcomes.

Standard error (log Peto Odds Ratio [OR]) by Peto Odds Ratio to evaluate publication bias for effect of lenalidomide in acute lymphoblastic leukaemia onset versus placebo (black circle, primary outcome), chemotherapy +/- SCT (red diamond, secondary outcome) or observation (green square, secondary outcome) in randomized controlled trials.


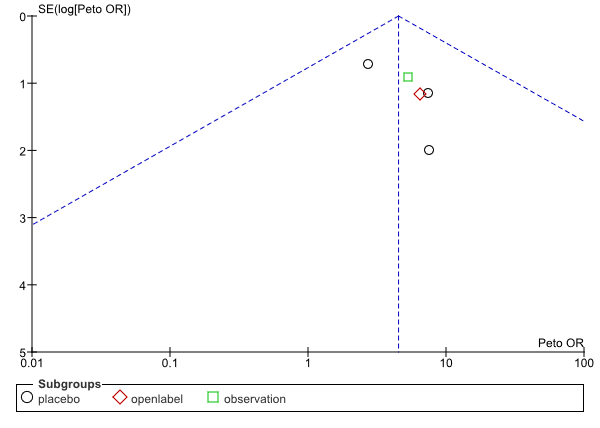


# Supplementary Table 6. Summary (A) and individual (B) risk of bias of all randomized controlled trials (n=18).

**A**

| **Study** | **Control** | **Study design and objectives** | **Bias in selection of participants and constitution of study groups** | **Bias due to withdrawal or loss to follow up (attrition)** | **Information bias regarding the drug safety outcome** | **Other information bias** | **Statistical methods to control confounding** | **Statistical methods excluding methods to control confounding** | **Conflict of interest** | **SUMMARY RISK OF BIAS** |
| --- | --- | --- | --- | --- | --- | --- | --- | --- | --- | --- |
| Attal et al (2012), IFM-2005-02, NCT00430365 | placebo | **Low** | **Unclear** | **Low** | **Unclear** | **Low** | **Low** | **Unclear** | **Low** | **Unclear** |
| Barlogie et al (2015), UARK 2009-09/110468, NCT01621672 | observation | **Low** | **Unclear** | **Unclear** | **Unclear** | **Unclear** | **Unclear** | **Unclear** | **Unclear** | **Unclear** |
| Bensmaine et al (2014), CC-5013-MM-026/ARUMM, NCT02112175 | placebo | **Low** | **Unclear** | **Unclear** | **Unclear** | **Unclear** | **Unclear** | **Unclear** | **Unclear** | **Unclear** |
| Brioli et al (2020), GERMAIN, NCT02145598 | placebo | **Low** | **Low** | **Low** | **Unclear** | **Low** | **Low** | **Unclear** | **Low** | **Unclear** |
| Dimopoulos et al (2007), CC-5013-MM-010, NCT00424047 | placebo | **Low** | **Low** | **Low** | **Unclear** | **Low** | **Low** | **Unclear** | **Low** | **Unclear** |
| Dimopoulos et al (2013), MMY-2045/SEQUENTIAL, NCT00908232 | chemo | **Low** | **Unclear** | **Unclear** | **Unclear** | **Unclear** | **Unclear** | **Unclear** | **Low** | **Unclear** |
| Gay et al (2021), FORTE/UNITO-MM-01, NCT02203643 | chemo | **Low** | **Low** | **Low** | **Unclear** | **Unclear** | **Low** | **Unclear** | **Low** | **Unclear** |
| Jones et al (2023), MYELOMA XI, NCT01554852 | observation | **Low** | **Low** | **Low** | **Unclear** | **Unclear** | **Low** | **Unclear** | **Low** | **Unclear** |
| Jacobus et al (2016), ECOG-E1A05, NCT00522392 | chemo | **Low** | **Unclear** | **Low** | **Unclear** | **Unclear** | **Low** | **Unclear** | **Low** | **Unclear** |
| Kumar et al (2012), EVOLUTION, NCT00507442 | chemo | **Low** | **Low** | **Low** | **Unclear** | **Unclear** | **Low** | **Unclear** | **Low** | **Unclear** |
| Lonial et al (2019), ECOG-E3A06, NCT01169337 | observation | **Low** | **Low** | **Low** | **Unclear** | **Unclear** | **Low** | **Unclear** | **Low** | **Unclear** |
| Mateos et al (2022), QuiReDex, NCT00480363 | observation | **Low** | **Unclear** | **Low** | **Unclear** | **Unclear** | **Low** | **Unclear** | **Low** | **Unclear** |
| McCarthy et al (2017), CALGB 100104, NCT00114101 | placebo | **Low** | **Low** | **Low** | **Unclear** | **Low** | **Low** | **Unclear** | **Low** | **Unclear** |
| Palumbo et al (2012), CC-5013-MM-015, NCT00405756 | placebo | **Low** | **Unclear** | **Low** | **Unclear** | **Low** | **Low** | **Unclear** | **Low** | **Unclear** |
| Palumbo et al (2014), RV-MM-PI-209/GIMEMA, NCT00551928 | chemo | **Low** | **Low** | **Low** | **Unclear** | **Unclear** | **Low** | **Unclear** | **Low** | **Unclear** |
| Slade et al (2022), MMRC-066/201411060, NCT02253316 | chemo | **Low** | **Unclear** | **Unclear** | **Unclear** | **Unclear** | **Low** | **Unclear** | **Low** | **Unclear** |
| Weber et al (2007), CC-5013-MM-009, NCT00056160 | placebo | **Low** | **Low** | **Low** | **Unclear** | **Low** | **Low** | **Unclear** | **Low** | **Unclear** |
| Zonder et al (2011), SWOG 0232, NCT00064038 | placebo | **Low** | **Unclear** | **Low** | **Unclear** | **Low** | **Low** | **Unclear** | **Low** | **Unclear** |

**B**

| **RISK OF BIAS ASSESSMENT CHECKLIST  FOR STUDIES INCLUDED IN SYSTEMATIC REVIEWS OF DRUG HARMS** | | | | |
| --- | --- | --- | --- | --- |
|  |  |  |  |  |
|  | **Study ID - Author** | Attal et al (2012), IFM-2005-02 |  |  |
|  |  |  |  |  |
| **A. STUDY DESIGN AND OBJECTIVES** | | | | |
| **A1. Are study objectives clearly specified and appropriate?** | | | | **Yes** |
|  | *Yes:* | *No:* |  |  |
|  | *Study objectives clearly specified and appropriate.* | *Study objectives are not clearly specified or not appropriate.* |  |  |
| **A2. Is study design clearly specified and appropriate?** | | | | **Yes** |
|  | *Yes:* | *No:* |  |  |
|  | *Study design clearly specified and appropriate.* | *Study design not clearly specified or not appropriate.* |  |  |
| **A3. Is the study design free of run-in/lead-in period before inclusion/randomization of participants?** | | | | **Yes** |
|  | *Yes:* | *Unclear:* | *No:* |  |
|  | *No run-in/lead-in period.* | *Not clear information.* | *Presence of a run-in/lead-in period.* |  |
| **A4. Cross-over designs: Is the study designed to adequately address carry-over effect?** | | | | **yes** |
| *N/A* | *Yes:* | *Unclear:* | *No:* |  |
|  | *Carry-over effect absent or adequately addressed (randomized order and sufficiently long wash-out period.* | *Not clear information.* | *Carry-over effect not adequately addressed and susceptible to bias the results.* |  |
| **A. RISK OF BIAS ASSESSMENT FOR STUDY DESIGN AND OBJECTIVES** | | | | **Low** |
|  | *Low:* | *Unclear:* | *High:* |  |
|  | *Plausible bias unlikely to seriously alter the results.* | *Plausible bias that raises some doubts about the results or when information on which to base risk of bias judgments is missing or poorly reported.* | *Plausible bias that seriously weakens confidence in the results.* |  |
| **Comments: Low risk of bias according to data provided by sources** | | | | |
|  |  |  |  |  |
| **B. BIAS IN SELECTION OF SUBJECTS AND CONSTITUTION OF STUDY GROUPS** | | | | |
| **B1. Was the method used to generate the allocation sequence adequate as to produce comparable groups?** | | | | **Yes** |
|  | *Yes:* | *Unclear:* | *No:* |  |
|  | *Allocation methods are adequate to produce comparable groups.* | *Allocation methods are not clearly reported.* | *Allocation methods are not adequate (e.g. assignment to treatment by birth date, week day, etc.), groups are not comparable.* |  |
| **B2. Was the method used to conceal the allocation sequence adequate as to produce comparable groups?** | | | | **unclear** |
|  | *Yes:* | *Unclear:* | *No:* |  |
|  | *Concealment is adequate.* | *Concealment methods are not clearly reported and groups may not be comparable.* | *Concealment methods are not adequate, groups are not comparable.* |  |
| **B3. Are all the subjects recruited from the same source population?** | | | | **Yes** |
|  | *Yes:* | *Unclear:* | *No:* |  |
|  | *All the subjects recruited from the same source population.* | *Unclear if all the subjects recruited from the same source population.* | *All the subjects are not recruited from the same source population.* |  |
| **B4. Were inclusion and exclusion criteria implemented uniformly across study groups?** | | | | **Yes** |
|  | *Yes:* | *Unclear:* | *No:* |  |
|  | *Selection criteria uniformly implemented.* | *Unclear if selection criteria are uniformly implemented.* | *Selection criteria not uniformly implemented.* |  |
| **B8. Are baseline characteristics and prognostic factors comparable between different groups?** | | | | **Yes** |
|  | *Yes:* | *Unclear:* | *No:* |  |
|  | *RCT: Groups are comparable at baseline.* | *No description of baseline characteristics or only significance tests.* | *The groups are unbalanced at baseline.* |  |
|  | *Cohort studies: Groups are comparable at baseline or matched for the main prognostic factors.* |  |  |  |
| **B. RISK OF BIAS ASSESSMENT FOR SELECTION OF PARTICIPANTS AND CONSTITUTION OF STUDY GROUPS** | | | | **Unclear** |
|  | *Low:* | *Unclear:* | *High:* |  |
|  | *Plausible bias unlikely to seriously alter the results.* | *Plausible bias that raises some doubts about the results or when information on which to base risk of bias judgments is missing or poorly reported.* | *Plausible bias that seriously weakens confidence in the results.* |  |
| **Comments: Low risk of bias according to data provided by sources** | | | | |
|  |  |  |  |  |
| **C. BIAS DUE TO WITHDRAWALS OR LOSS OF FOLLOW-UP (ATTRITION)** | | | | |
| **C1. Are the number of participants clearly reported throughout the study?** | | | | **Yes** |
|  | *Yes:* | *No:* |  |  |
|  | *Numbers of participants throughout the study are reported. Complete flow chart.* | *Numbers of patients at every stage is not clearly reported. Confusing information is reported regarding the number of participants. No or incomplete flow chart.* |  |  |
| **C2. Is the number of drop-outs/withdrawals due to harmful outcome clearly stated for each treatment arm?** | | | | **Yes** |
|  | *Yes:* | *No:* |  |  |
|  | *The number of drop-outs due to harmful outcome is specified.* | *The number of drop-outs due to harmful outcome is not specified, unclear or combined.* |  |  |
| **C3. Does the study adequately address biased loss to follow-up?** | | | | **Yes** |
|  | *Yes:* | *Unclear:* | *No:* |  |
|  | *Complete follow-up or drop-outs unlikely to introduce bias or adequately controlled.* | *Drop-outs/withdraws due to harmful outcome are not clearly reported.* | *Loss to follow-up affects the safety outcome and is not adequately controlled.* |  |
| **C4. Are the results based on an intention-to-treat analysis?** | | | | **Yes** |
|  | *Yes:* | *Unclear:* | *No:* |  |
|  | *Results are based on a strict intention-to-treat analysis.* | *Not clear if an intention-to-treat analysis is performed. No strict intention-to-treat analysis.* | *Results are not based on intention-to-treat analysis (not done or not possible).* |  |
| **C. RISK OF BIAS DUE TO WITHDRAWALS OR LOSS OF FOLLOW-UP (ATTRITION)** | | | | **Low** |
|  | *Low:* | *Unclear:* | *High:* |  |
|  | *Plausible bias unlikely to seriously alter the results.* | *Plausible bias that raises some doubts about the results or when information on which to base risk of bias judgments is missing or poorly reported.* | *Plausible bias that seriously weakens confidence in the results.* |  |
| **Comments: Low risk of bias according to data provided by sources** | | | | |
|  |  |  |  |  |
| **D. INFORMATION BIAS REGARDING THE HARMFULL OUTCOME** | | | | |
| **D1. Is the definition of the harmful outcome clearly stated?** | | | | **yes** |
|  | *Yes:* | *No:* |  |  |
|  | *RCT: clear / standardized definition of the harmful outcome (e.g. diagnostic codes, clinical and laboratory data). Cohort studies: clear definition of the outcome. Case-control studies: clear definition of cases.* | *Definition of the harmful outcome not reported or that leads to confusion. Terms not well-constructed, wrong definition.* |  |  |
| **D2. If applicable, is the severity of the harmful outcome clearly stated?** | | | | **yes** |
|  | *N/A:* | *Yes:* | *No:* |  |
|  | *Self evident severity (e.g. death).* | *Detailed degree of severity or reference to a known scale of severity or a new scale developed for the study.* | *Unclear degrees of severity or without clear boundaries between them.* |  |
| **D3. Was the blinding methods of participants regarding the intervention appropriate considering the nature of the harmful outcome?** | | | | **Unclear** |
|  | *Yes:* | *Unclear:* | *No:* |  |
|  | *Blinding ensured (and unlikely broken) or outcome not likely to be influenced by lack of blinding.* | *There is no sufficient information regarding the process of blinding or the outcome assessment.* | *No blinding (or incomplete blinding or risk of broken blinding) and outcome likely to be influenced by lack of blinding.* |  |
| **D4. Was the blinding methods of harmful outcome assessment appropriate considering the nature of the harmful outcome?** | | | | **Unclear** |
|  | *Yes:* | *Unclear:* | *No:* |  |
|  | *Blinding ensured (and unlikely broken) or outcome assessment not likely to be influenced by lack of blinding.* | *There is no sufficient information regarding the process of blinding of outcome assessment.* | *No blinding (or incomplete blinding or risk of broken blinding) and outcome likely to be influenced by lack of blinding.* |  |
| **D5. Was the duration of follow-up adequate to assess the harmful outcome?** | | | | **Unclear** |
|  | *Yes:* | *Unclear:* | *No:* |  |
|  | *Sufficient duration of follow-up to assess the outcome.* | *It is unclear whether the duration of follow-up is adequate.* | *Too short duration of follow-up.* |  |
| **D6. Was the methods for ascertaining the harmful outcome adequately constructed and equal for all participants?** | | | | **Unclear** |
|  | *Yes:* | *Unclear:* | *No:* |  |
|  | *Adequate or validated methods of outcome measurement for all participants. Clinical reactions medically confirmed by a physician. Minimized risk of misclassification or differential assessment, reporting or detection.  RCT : Active harmful outcome surveillance (prospective/retrospective case-record review, questionnaires, patient’s diary/checklist…) .* | *There is no or not sufficient information to clearly determine how information on harmful outcome is collected or the process of minimizing misclassification.* | *Substantial risk of misclassification of outcome or differential assessment, reporting or detection. Clinical reactions not medically confirmed. RCT : Passive harmful outcome surveillance (patient’s volunteer reporting).* |  |
| **D7. Are the number of harmful outcome and the number of patients with a harmful outcome reported in both treatment arms?** | | | | **Yes** |
|  | *Yes:* | *Unclear:* | *No:* |  |
|  | *Numbers are reported. It is possible to calculate the rates of harmful outcome.* | *Confusion between the number of harmful outcomes or the number of patients with a harmful outcome, or general statements such as “5% of patients developed a harmful outcome”.* | *Neither the number of harmful outcomes nor the number of patients with a harmful outcome is reported. Or numbers are combining both treatment arms.* |  |
| **D8. Is the time frequency of harmful outcome assessment during the follow-up period appropriate?** | | | | **Unclear** |
|  | *Yes:* | *Unclear:* | *No:* |  |
|  | *For all study groups, the time frequency at which the harmful outcome is assessed is appropriate.* | *General statements such as “patients were routinely assessed for harmful outcomes”.* | *There is no regular collection of data on harmful outcomes during the study.* |  |
| **D9. Was the time between the exposure to a drug and the onset of the harmful outcome reported?** | | | | **Yes** |
|  | *Yes:* | *Unclear:* | *No:* |  |
|  | *The time between the drug exposure to the onset of harmful outcome is specified.* | *The authors do not report a clear time frame between drug exposure and harmful outcome.* | *The authors do not report the time between the drug exposure to the onset of harmful outcome.* |  |
| **D10. Was the process of determining that the harmful outcome is linked to the drug appropriate? Was the process blinded to the assigned treatment?** | | | | **Unclear** |
|  | *Yes:* | *Unclear:* | *No:* |  |
|  | *Methods for causality assessment are appropriate and, if applicable, made by investigators blinded to the intervention.* | *Unclear how the causality attribution is made. It is not clear who make the assessment or whether it is blinded to the assigned treatment.* | *Causality assessment is made by investigators not blinded to the intervention, or by participants or sponsors, or unblinding of treatment assignment precedes the decision to withdraw.* |  |
| **D. RISK OF BIAS ASSESSMENT FOR INFORMATION BIAS REGARDING THE HARMFULL OUTCOME** | | | | **Unclear** |
|  | *Low:* | *Unclear:* | *High:* |  |
|  | *Plausible bias unlikely to seriously alter the results.* | *Plausible bias that raises some doubts about the results or when information on which to base risk of bias judgments is missing or poorly reported.* | *Plausible bias that seriously weakens confidence in the results.* |  |
| **Comments: Unclear risk of bias** | | | | |
|  |  |  |  |  |
| **E. OTHER INFORMATION BIAS** | | | | |
| **E1. Is blinding of care givers during follow-up adequately performed in order to avoid differential care between study groups (performance bias)?** | | | | **Yes** |
|  | *Yes:* | *Unclear:* | *No:* |  |
|  | *There is no risk of differential care or it is adequately addressed.* | *Unclear risk of bias due to differential care.* | *The bias due to differential care is not controlled.* |  |
| **E5. Does the study appear free of other information bias ?** | | | | **Yes** |
|  | *Yes:* | *Unclear:* | *No:* |  |
|  | *The study appears to be free of other information bias.* | *Unclear presence of other information bias.* | *Additional source of other information bias.* |  |
| **E. RISK OF BIAS ASSESSMENT FOR OTHER INFORMATION BIAS** | | | | **Low** |
|  | *Low:* | *Unclear:* | *High:* |  |
|  | *Plausible bias unlikely to seriously alter the results.* | *Plausible bias that raises some doubts about the results or when information on which to base risk of bias judgments is missing or poorly reported.* | *Plausible bias that seriously weakens confidence in the results.* |  |
| **Comments: low risk of bias** | | | | |
|  |  |  |  |  |
| **F. STATISTICAL METHODS TO CONTROL CONFOUNDING** | | | | |
| **F5. Does the study adequately address residual or unmeasured confounding?** | | | | **Yes** |
|  | *Yes:* | *Unclear:* | *No:* |  |
|  | *The study adequately addresses residual or unmeasured confounding.* | *Unclear presence of residual or unmeasured confounding.* | *Residual or unmeasured confounding is likely to be important.* |  |
| **F. RISK OF BIAS ASSESSMENT FOR STATISTICAL METHODS TO CONTROL CONFOUNDING:** | | | | **Low** |
|  | *Low:* | *Unclear:* | *High:* |  |
|  | *Plausible bias unlikely to seriously alter the results.* | *Plausible bias that raises some doubts about the results or when information on which to base risk of bias judgments is missing or poorly reported.* | *Plausible bias that seriously weakens confidence in the results.* |  |
| **Comments: Randomization was stratified according to baseline levels of serum β2-microglobulin (≤3 mg per liter or >3 mg per liter), the presence or absence of a 13q deletion on the basis of fluorescence in situ hybridization, and response after transplantation achieved at the time of randomization (a complete or very good partial response vs. a partial response or stable disease).** | | | | |
|  |  |  |  |  |
| **G. STATISTICAL METHODS EXCLUDING METHODS TO CONTROL CONFOUNDING** | | | | |
| **G1. Are the statistical methods used to analyze the harmful outcome appropriate?** | | | | **Unclear** |
|  | *Yes:* | *Unclear:* | *No:* |  |
|  | *Statistical techniques are appropriate to the data. If the distribution of the data (normal or not) is not described, it must be assumed that the estimates used were appropriate.* | *There is no description of the statistical techniques used, or the description is vague and not understandable.* | *The statistical techniques used are not appropriate.* |  |
| **G2. Is a survival analysis performed when there are individual differences in length of follow-up?** | | | | **Yes** |
|  | *Yes:* | *Unclear:* | *No:* |  |
|  | *Follow-up is the same for all study patients, if not survival analysis is performed.* | *Unclear whether there are different lengths of follow-up or whether they are taken into account.* | *Differences of follow up were ignored.* |  |
| **G3. If applicable, is composite outcome of harms adequately constructed?** | | | | **N/A** |
| *N/A* | *Yes:* | *Unclear:* | *No:* |  |
|  | *Composite outcome appropriate.* | *Unclear whether composite outcome is appropriate.* | *Construction of composite not described or not appropriate.* |  |
| **G6.** **Are the results consistent in primary and secondary analyses? Are confounding effects consistent with known associations?** | | | | **Yes** |
|  | *Yes:* | *Unclear:* | *No:* |  |
|  | *Consistency of primary, secondary analyses and consistency of confounding effects with known associations.* | *Not sufficient information to determine consistency.* | *No consistency of primary, secondary analyses or no consistency of confounding effects with known associations.* |  |
| **G. RISK OF BIAS ASSESSMENT FOR STATISTICAL METHODS EXCLUDING METHODS TO CONTROL CONFOUNDING:** | | | | **Unclear** |
|  | *Low:* | *Unclear:* | *High:* |  |
|  | *Plausible bias unlikely to seriously alter the results.* | *Plausible bias that raises some doubts about the results or when information on which to base risk of bias judgments is missing or poorly reported.* | *Plausible bias that seriously weakens confidence in the results.* |  |
| **Comments: Unclear risk of bias** | | | | |
|  | | | |  |
| **H. CONFLICT OF INTEREST** | | | | |
| **H1. Were the conflict of interest or sources of funding clearly acknowledged?** | | | | **Yes** |
|  | *Yes:* | *No:* |  |  |
|  | *Potential sources of support are acknowledged.* | *No sources of funding reported or not sufficient information.* |  |  |
| **H2. Does the study appear free of conflicts of interest susceptible to have influenced design, analysis or reporting (selective reporting of outcome or analysis)?** | | | | **Yes** |
|  | *Yes:* | *Unclear:* | *No:* |  |
|  | *No conflicts of interest or not susceptible to have influenced design, analysis or reporting.* | *It is unclear if there are conflicts of interest or if they are susceptible to have influenced design, analysis or reporting.* | *Conflicts of interest susceptible to have influenced design, analysis or reporting.* |  |
| **H. RISK OF BIAS ASSESSMENT FOR CONFLICT OF INTEREST** | | | | **Low** |
|  | *Low:* | *Unclear:* | *High:* |  |
|  | *Plausible bias unlikely to seriously alter the results.* | *Plausible bias that raises some doubts about the results or when information on which to base risk of bias judgments is missing or poorly reported.* | *Plausible bias that seriously weakens confidence in the results.* |  |
|  |  |  |  |  |
| **SUMMARY RISK-OF-BIAS ASSESSMENT FOR THE STUDY** | | | | |
| **RISK OF BIAS ASSESSMENT FOR THE STUDY** | | | | **Unclear** |
|  | *Low:* | *Unclear:* | *High:* |  |
|  | *Low risk of bias for all key domains.* | *Unclear risk of bias for one or more key domain.* | *High risk of bias for one or more key domains.* |  |

| **RISK OF BIAS ASSESSMENT CHECKLIST  FOR STUDIES INCLUDED IN SYSTEMATIC REVIEWS OF DRUG HARMS** | | | | |
| --- | --- | --- | --- | --- |
|  |  |  |  |  |
|  | **Study ID - Author** | Barlogie et al (2015), UARK 2009-09/110468, NCT01621672 |  |  |
|  |  |  |  |  |
| **A. STUDY DESIGN AND OBJECTIVES** | | | | |
| **A1. Are study objectives clearly specified and appropriate?** | | | | **Yes** |
|  | *Yes:* | *No:* |  |  |
|  | *Study objectives clearly specified and appropriate.* | *Study objectives are not clearly specified or not appropriate.* |  |  |
| **A2. Is study design clearly specified and appropriate?** | | | | **Yes** |
|  | *Yes:* | *No:* |  |  |
|  | *Study design clearly specified and appropriate.* | *Study design not clearly specified or not appropriate.* |  |  |
| **A3. Is the study design free of run-in/lead-in period before inclusion/randomization of participants?** | | | | **Yes** |
|  | *Yes:* | *Unclear:* | *No:* |  |
|  | *No run-in/lead-in period.* | *Not clear information.* | *Presence of a run-in/lead-in period.* |  |
| **A4. Cross-over designs: Is the study designed to adequately address carry-over effect?** | | | | **Yes** |
| *N/A* | *Yes:* | *Unclear:* | *No:* |  |
|  | *Carry-over effect absent or adequately addressed (randomized order and sufficiently long wash-out period.* | *Not clear information.* | *Carry-over effect not adequately addressed and susceptible to bias the results.* |  |
| **A. RISK OF BIAS ASSESSMENT FOR STUDY DESIGN AND OBJECTIVES** | | | | **Low** |
|  | *Low:* | *Unclear:* | *High:* |  |
|  | *Plausible bias unlikely to seriously alter the results.* | *Plausible bias that raises some doubts about the results or when information on which to base risk of bias judgments is missing or poorly reported.* | *Plausible bias that seriously weakens confidence in the results.* |  |
| **Comments: Low risk of bias according to data provided by sources** | | | | |
|  |  |  |  |  |
| **B. BIAS IN SELECTION OF SUBJECTS AND CONSTITUTION OF STUDY GROUPS** | | | | |
| **B1. Was the method used to generate the allocation sequence adequate as to produce comparable groups?** | | | | **Yes** |
|  | *Yes:* | *Unclear:* | *No:* |  |
|  | *Allocation methods are adequate to produce comparable groups.* | *Allocation methods are not clearly reported.* | *Allocation methods are not adequate (e.g. assignment to treatment by birth date, week day, etc.), groups are not comparable.* |  |
| **B2. Was the method used to conceal the allocation sequence adequate as to produce comparable groups?** | | | | **Unclear** |
|  | *Yes:* | *Unclear:* | *No:* |  |
|  | *Concealment is adequate.* | *Concealment methods are not clearly reported and groups may not be comparable.* | *Concealment methods are not adequate, groups are not comparable.* |  |
| **B3. Are all the subjects recruited from the same source population?** | | | | **Yes** |
|  | *Yes:* | *Unclear:* | *No:* |  |
|  | *All the subjects recruited from the same source population.* | *Unclear if all the subjects recruited from the same source population.* | *All the subjects are not recruited from the same source population.* |  |
| **B4. Were inclusion and exclusion criteria implemented uniformly across study groups?** | | | | **Yes** |
|  | *Yes:* | *Unclear:* | *No:* |  |
|  | *Selection criteria uniformly implemented.* | *Unclear if selection criteria are uniformly implemented.* | *Selection criteria not uniformly implemented.* |  |
| **B8. Are baseline characteristics and prognostic factors comparable between different groups?** | | | | **Unclear** |
|  | *Yes:* | *Unclear:* | *No:* |  |
|  | *RCT: Groups are comparable at baseline.* | *No description of baseline characteristics or only significance tests.* | *The groups are unbalanced at baseline.* |  |
|  | *Cohort studies: Groups are comparable at baseline or matched for the main prognostic factors.* |  |  |  |
| **B. RISK OF BIAS ASSESSMENT FOR SELECTION OF PARTICIPANTS AND CONSTITUTION OF STUDY GROUPS** | | | | **Unclear** |
|  | *Low:* | *Unclear:* | *High:* |  |
|  | *Plausible bias unlikely to seriously alter the results.* | *Plausible bias that raises some doubts about the results or when information on which to base risk of bias judgments is missing or poorly reported.* | *Plausible bias that seriously weakens confidence in the results.* |  |
| **Comments: unclear risk of bias according to data provided by sources** | | | | |
|  |  |  |  |  |
| **C. BIAS DUE TO WITHDRAWALS OR LOSS OF FOLLOW-UP (ATTRITION)** | | | | |
| **C1. Are the number of participants clearly reported throughout the study?** | | | | **Yes** |
|  | *Yes:* | *No:* |  |  |
|  | *Numbers of participants throughout the study are reported. Complete flow chart.* | *Numbers of patients at every stage is not clearly reported. Confusing information is reported regarding the number of participants. No or incomplete flow chart.* |  |  |
| **C2. Is the number of drop-outs/withdrawals due to harmful outcome clearly stated for each treatment arm?** | | | | **No** |
|  | *Yes:* | *No:* |  |  |
|  | *The number of drop-outs due to harmful outcome is specified.* | *The number of drop-outs due to harmful outcome is not specified, unclear or combined.* |  |  |
| **C3. Does the study adequately address biased loss to follow-up?** | | | | **Unclear** |
|  | *Yes:* | *Unclear:* | *No:* |  |
|  | *Complete follow-up or drop-outs unlikely to introduce bias or adequately controlled.* | *Drop-outs/withdraws due to harmful outcome are not clearly reported.* | *Loss to follow-up affects the safety outcome and is not adequately controlled.* |  |
| **C4. Are the results based on an intention-to-treat analysis?** | | | | **yes** |
|  | *Yes:* | *Unclear:* | *No:* |  |
|  | *Results are based on a strict intention-to-treat analysis.* | *Not clear if an intention-to-treat analysis is performed. No strict intention-to-treat analysis.* | *Results are not based on intention-to-treat analysis (not done or not possible).* |  |
| **C. RISK OF BIAS DUE TO WITHDRAWALS OR LOSS OF FOLLOW-UP (ATTRITION)** | | | | **Unclear** |
|  | *Low:* | *Unclear:* | *High:* |  |
|  | *Plausible bias unlikely to seriously alter the results.* | *Plausible bias that raises some doubts about the results or when information on which to base risk of bias judgments is missing or poorly reported.* | *Plausible bias that seriously weakens confidence in the results.* |  |
| **Comments: unclear risk of bias according to data provided by sources** | | | | |
|  |  |  |  |  |
| **D. INFORMATION BIAS REGARDING THE HARMFULL OUTCOME** | | | | |
| **D1. Is the definition of the harmful outcome clearly stated?** | | | | **No** |
|  | *Yes:* | *No:* |  |  |
|  | *RCT: clear / standardized definition of the harmful outcome (e.g. diagnostic codes, clinical and laboratory data). Cohort studies: clear definition of the outcome. Case-control studies: clear definition of cases.* | *Definition of the harmful outcome not reported or that leads to confusion. Terms not well-constructed, wrong definition.* |  |  |
| **D2. If applicable, is the severity of the harmful outcome clearly stated?** | | | | **N/A** |
|  | *N/A:* | *Yes:* | *No:* |  |
|  | *Self evident severity (e.g. death).* | *Detailed degree of severity or reference to a known scale of severity or a new scale developed for the study.* | *Unclear degrees of severity or without clear boundaries between them.* |  |
| **D3. Was the blinding methods of participants regarding the intervention appropriate considering the nature of the harmful outcome?** | | | | **Unclear** |
|  | *Yes:* | *Unclear:* | *No:* |  |
|  | *Blinding ensured (and unlikely broken) or outcome not likely to be influenced by lack of blinding.* | *There is no sufficient information regarding the process of blinding or the outcome assessment.* | *No blinding (or incomplete blinding or risk of broken blinding) and outcome likely to be influenced by lack of blinding.* |  |
| **D4. Was the blinding methods of harmful outcome assessment appropriate considering the nature of the harmful outcome?** | | | | **Unclear** |
|  | *Yes:* | *Unclear:* | *No:* |  |
|  | *Blinding ensured (and unlikely broken) or outcome assessment not likely to be influenced by lack of blinding.* | *There is no sufficient information regarding the process of blinding of outcome assessment.* | *No blinding (or incomplete blinding or risk of broken blinding) and outcome likely to be influenced by lack of blinding.* |  |
| **D5. Was the duration of follow-up adequate to assess the harmful outcome?** | | | | **Unclear** |
|  | *Yes:* | *Unclear:* | *No:* |  |
|  | *Sufficient duration of follow-up to assess the outcome.* | *It is unclear whether the duration of follow-up is adequate.* | *Too short duration of follow-up.* |  |
| **D6. Was the methods for ascertaining the harmful outcome adequately constructed and equal for all participants?** | | | | **Unclear** |
|  | *Yes:* | *Unclear:* | *No:* |  |
|  | *Adequate or validated methods of outcome measurement for all participants. Clinical reactions medically confirmed by a physician. Minimized risk of misclassification or differential assessment, reporting or detection.  RCT : Active harmful outcome surveillance (prospective/retrospective case-record review, questionnaires, patient’s diary/checklist…) .* | *There is no or not sufficient information to clearly determine how information on harmful outcome is collected or the process of minimizing misclassification.* | *Substantial risk of misclassification of outcome or differential assessment, reporting or detection. Clinical reactions not medically confirmed. RCT : Passive harmful outcome surveillance (patient’s volunteer reporting).* |  |
| **D7. Are the number of harmful outcome and the number of patients with a harmful outcome reported in both treatment arms?** | | | | **Unclear** |
|  | *Yes:* | *Unclear:* | *No:* |  |
|  | *Numbers are reported. It is possible to calculate the rates of harmful outcome.* | *Confusion between the number of harmful outcomes or the number of patients with a harmful outcome, or general statements such as “5% of patients developed a harmful outcome”.* | *Neither the number of harmful outcomes nor the number of patients with a harmful outcome is reported. Or numbers are combining both treatment arms.* |  |
| **D8. Is the time frequency of harmful outcome assessment during the follow-up period appropriate?** | | | | **Unclear** |
|  | *Yes:* | *Unclear:* | *No:* |  |
|  | *For all study groups, the time frequency at which the harmful outcome is assessed is appropriate.* | *General statements such as “patients were routinely assessed for harmful outcomes”.* | *There is no regular collection of data on harmful outcomes during the study.* |  |
| **D9. Was the time between the exposure to a drug and the onset of the harmful outcome reported?** | | | | **No** |
|  | *Yes:* | *Unclear:* | *No:* |  |
|  | *The time between the drug exposure to the onset of harmful outcome is specified.* | *The authors do not report a clear time frame between drug exposure and harmful outcome.* | *The authors do not report the time between the drug exposure to the onset of harmful outcome.* |  |
| **D10. Was the process of determining that the harmful outcome is linked to the drug appropriate? Was the process blinded to the assigned treatment?** | | | | **Unclear** |
|  | *Yes:* | *Unclear:* | *No:* |  |
|  | *Methods for causality assessment are appropriate and, if applicable, made by investigators blinded to the intervention.* | *Unclear how the causality attribution is made. It is not clear who make the assessment or whether it is blinded to the assigned treatment.* | *Causality assessment is made by investigators not blinded to the intervention, or by participants or sponsors, or unblinding of treatment assignment precedes the decision to withdraw.* |  |
| **D. RISK OF BIAS ASSESSMENT FOR INFORMATION BIAS REGARDING THE HARMFULL OUTCOME** | | | | **Unclear** |
|  | *Low:* | *Unclear:* | *High:* |  |
|  | *Plausible bias unlikely to seriously alter the results.* | *Plausible bias that raises some doubts about the results or when information on which to base risk of bias judgments is missing or poorly reported.* | *Plausible bias that seriously weakens confidence in the results.* |  |
| **Comments: Unclear risk of bias** | | | | |
| **E. OTHER INFORMATION BIAS** | | | | |
| **E1. Is blinding of care givers during follow-up adequately performed in order to avoid differential care between study groups (performance bias)?** | | | | **Unclear** |
|  | *Yes:* | *Unclear:* | *No:* |  |
|  | *There is no risk of differential care or it is adequately addressed.* | *Unclear risk of bias due to differential care.* | *The bias due to differential care is not controlled.* |  |
| **E5. Does the study appear free of other information bias ?** | | | | **Unclear** |
|  | *Yes:* | *Unclear:* | *No:* |  |
|  | *The study appears to be free of other information bias.* | *Unclear presence of other information bias.* | *Additional source of other information bias.* |  |
| **E. RISK OF BIAS ASSESSMENT FOR OTHER INFORMATION BIAS** | | | | **Unclear** |
|  | *Low:* | *Unclear:* | *High:* |  |
|  | *Plausible bias unlikely to seriously alter the results.* | *Plausible bias that raises some doubts about the results or when information on which to base risk of bias judgments is missing or poorly reported.* | *Plausible bias that seriously weakens confidence in the results.* |  |
| **Comments: unclear of bias -> open-label** | | | | |
|  |  |  |  |  |
| **F. STATISTICAL METHODS TO CONTROL CONFOUNDING** | | | | |
| **F5. Does the study adequately address residual or unmeasured confounding?** | | | | **Unclear** |
|  | *Yes:* | *Unclear:* | *No:* |  |
|  | *The study adequately addresses residual or unmeasured confounding.* | *Unclear presence of residual or unmeasured confounding.* | *Residual or unmeasured confounding is likely to be important.* |  |
| **F. RISK OF BIAS ASSESSMENT FOR STATISTICAL METHODS TO CONTROL CONFOUNDING:** | | | | **Unclear** |
|  | *Low:* | *Unclear:* | *High:* |  |
|  | *Plausible bias unlikely to seriously alter the results.* | *Plausible bias that raises some doubts about the results or when information on which to base risk of bias judgments is missing or poorly reported.* | *Plausible bias that seriously weakens confidence in the results.* |  |
| **Comments: Unclear risk of bias** | | | | |
|  |  |  |  |  |
| **G. STATISTICAL METHODS EXCLUDING METHODS TO CONTROL CONFOUNDING** | | | | |
| **G1. Are the statistical methods used to analyze the harmful outcome appropriate?** | | | | **Unclear** |
|  | *Yes:* | *Unclear:* | *No:* |  |
|  | *Statistical techniques are appropriate to the data. If the distribution of the data (normal or not) is not described, it must be assumed that the estimates used were appropriate.* | *There is no description of the statistical techniques used, or the description is vague and not understandable.* | *The statistical techniques used are not appropriate.* |  |
| **G2. Is a survival analysis performed when there are individual differences in length of follow-up?** | | | | **Unclear** |
|  | *Yes:* | *Unclear:* | *No:* |  |
|  | *Follow-up is the same for all study patients, if not survival analysis is performed.* | *Unclear whether there are different lengths of follow-up or whether they are taken into account.* | *Differences of follow up were ignored.* |  |
| **G3. If applicable, is composite outcome of harms adequately constructed?** | | | | **N/A** |
| *N/A* | *Yes:* | *Unclear:* | *No:* |  |
|  | *Composite outcome appropriate.* | *Unclear whether composite outcome is appropriate.* | *Construction of composite not described or not appropriate.* |  |
| **G6.** **Are the results consistent in primary and secondary analyses? Are confounding effects consistent with known associations?** | | | | **Unclear** |
|  | *Yes:* | *Unclear:* | *No:* |  |
|  | *Consistency of primary, secondary analyses and consistency of confounding effects with known associations.* | *Not sufficient information to determine consistency.* | *No consistency of primary, secondary analyses or no consistency of confounding effects with known associations.* |  |
| **G. RISK OF BIAS ASSESSMENT FOR STATISTICAL METHODS EXCLUDING METHODS TO CONTROL CONFOUNDING:** | | | | **Unclear** |
|  | *Low:* | *Unclear:* | *High:* |  |
|  | *Plausible bias unlikely to seriously alter the results.* | *Plausible bias that raises some doubts about the results or when information on which to base risk of bias judgments is missing or poorly reported.* | *Plausible bias that seriously weakens confidence in the results.* |  |
| **Comments: Unclear risk of bias** | | | | |
|  | | | |  |
| **H. CONFLICT OF INTEREST** | | | | |
| **H1. Were the conflict of interest or sources of funding clearly acknowledged?** | | | | **Unclear** |
|  | *Yes:* | *No:* |  |  |
|  | *Potential sources of support are acknowledged.* | *No sources of funding reported or not sufficient information.* |  |  |
| **H2. Does the study appear free of conflicts of interest susceptible to have influenced design, analysis or reporting (selective reporting of outcome or analysis)?** | | | | **Unclear** |
|  | *Yes:* | *Unclear:* | *No:* |  |
|  | *No conflicts of interest or not susceptible to have influenced design, analysis or reporting.* | *It is unclear if there are conflicts of interest or if they are susceptible to have influenced design, analysis or reporting.* | *Conflicts of interest susceptible to have influenced design, analysis or reporting.* |  |
| **H. RISK OF BIAS ASSESSMENT FOR CONFLICT OF INTEREST** | | | | **Unclear** |
|  | *Low:* | *Unclear:* | *High:* |  |
|  | *Plausible bias unlikely to seriously alter the results.* | *Plausible bias that raises some doubts about the results or when information on which to base risk of bias judgments is missing or poorly reported.* | *Plausible bias that seriously weakens confidence in the results.* |  |
|  |  |  |  |  |
| **SUMMARY RISK-OF-BIAS ASSESSMENT FOR THE STUDY** | | | | |
| **RISK OF BIAS ASSESSMENT FOR THE STUDY** | | | | **Unclear** |
|  | *Low:* | *Unclear:* | *High:* |  |
|  | *Low risk of bias for all key domains.* | *Unclear risk of bias for one or more key domain.* | *High risk of bias for one or more key domains.* |  |

| **RISK OF BIAS ASSESSMENT CHECKLIST  FOR STUDIES INCLUDED IN SYSTEMATIC REVIEWS OF DRUG HARMS** | | | | |
| --- | --- | --- | --- | --- |
|  |  |  |  |  |
|  | **Study ID - Author** | Bensmaine et al (2014), CC-5013-MM-026/ARUMM, NCT02112175 |  |  |
|  |  |  |  |  |
| **A. STUDY DESIGN AND OBJECTIVES** | | | | |
| **A1. Are study objectives clearly specified and appropriate?** | | | | **Yes** |
|  | *Yes:* | *No:* |  |  |
|  | *Study objectives clearly specified and appropriate.* | *Study objectives are not clearly specified or not appropriate.* |  |  |
| **A2. Is study design clearly specified and appropriate?** | | | | **Yes** |
|  | *Yes:* | *No:* |  |  |
|  | *Study design clearly specified and appropriate.* | *Study design not clearly specified or not appropriate.* |  |  |
| **A3. Is the study design free of run-in/lead-in period before inclusion/randomization of participants?** | | | | **Yes** |
|  | *Yes:* | *Unclear:* | *No:* |  |
|  | *No run-in/lead-in period.* | *Not clear information.* | *Presence of a run-in/lead-in period.* |  |
| **A4. Cross-over designs: Is the study designed to adequately address carry-over effect?** | | | | **Yes** |
| *N/A* | *Yes:* | *Unclear:* | *No:* |  |
|  | *Carry-over effect absent or adequately addressed (randomized order and sufficiently long wash-out period.* | *Not clear information.* | *Carry-over effect not adequately addressed and susceptible to bias the results.* |  |
| **A. RISK OF BIAS ASSESSMENT FOR STUDY DESIGN AND OBJECTIVES** | | | | **Low** |
|  | *Low:* | *Unclear:* | *High:* |  |
|  | *Plausible bias unlikely to seriously alter the results.* | *Plausible bias that raises some doubts about the results or when information on which to base risk of bias judgments is missing or poorly reported.* | *Plausible bias that seriously weakens confidence in the results.* |  |
| **Comments: Low risk of bias according to data provided by sources** | | | | |
|  |  |  |  |  |
| **B. BIAS IN SELECTION OF SUBJECTS AND CONSTITUTION OF STUDY GROUPS** | | | | |
| **B1. Was the method used to generate the allocation sequence adequate as to produce comparable groups?** | | | | **Yes** |
|  | *Yes:* | *Unclear:* | *No:* |  |
|  | *Allocation methods are adequate to produce comparable groups.* | *Allocation methods are not clearly reported.* | *Allocation methods are not adequate (e.g. assignment to treatment by birth date, week day, etc.), groups are not comparable.* |  |
| **B2. Was the method used to conceal the allocation sequence adequate as to produce comparable groups?** | | | | **Unclear** |
|  | *Yes:* | *Unclear:* | *No:* |  |
|  | *Concealment is adequate.* | *Concealment methods are not clearly reported and groups may not be comparable.* | *Concealment methods are not adequate, groups are not comparable.* |  |
| **B3. Are all the subjects recruited from the same source population?** | | | | **Yes** |
|  | *Yes:* | *Unclear:* | *No:* |  |
|  | *All the subjects recruited from the same source population.* | *Unclear if all the subjects recruited from the same source population.* | *All the subjects are not recruited from the same source population.* |  |
| **B4. Were inclusion and exclusion criteria implemented uniformly across study groups?** | | | | **Yes** |
|  | *Yes:* | *Unclear:* | *No:* |  |
|  | *Selection criteria uniformly implemented.* | *Unclear if selection criteria are uniformly implemented.* | *Selection criteria not uniformly implemented.* |  |
| **B8. Are baseline characteristics and prognostic factors comparable between different groups?** | | | | **Unclear** |
|  | *Yes:* | *Unclear:* | *No:* |  |
|  | *RCT: Groups are comparable at baseline.* | *No description of baseline characteristics or only significance tests.* | *The groups are unbalanced at baseline.* |  |
|  | *Cohort studies: Groups are comparable at baseline or matched for the main prognostic factors.* |  |  |  |
| **B. RISK OF BIAS ASSESSMENT FOR SELECTION OF PARTICIPANTS AND CONSTITUTION OF STUDY GROUPS** | | | | **Unclear** |
|  | *Low:* | *Unclear:* | *High:* |  |
|  | *Plausible bias unlikely to seriously alter the results.* | *Plausible bias that raises some doubts about the results or when information on which to base risk of bias judgments is missing or poorly reported.* | *Plausible bias that seriously weakens confidence in the results.* |  |
| **Comments: unclear risk of bias according to data provided by sources** | | | | |
|  |  |  |  |  |
| **C. BIAS DUE TO WITHDRAWALS OR LOSS OF FOLLOW-UP (ATTRITION)** | | | | |
| **C1. Are the number of participants clearly reported throughout the study?** | | | | **Yes** |
|  | *Yes:* | *No:* |  |  |
|  | *Numbers of participants throughout the study are reported. Complete flow chart.* | *Numbers of patients at every stage is not clearly reported. Confusing information is reported regarding the number of participants. No or incomplete flow chart.* |  |  |
| **C2. Is the number of drop-outs/withdrawals due to harmful outcome clearly stated for each treatment arm?** | | | | **No** |
|  | *Yes:* | *No:* |  |  |
|  | *The number of drop-outs due to harmful outcome is specified.* | *The number of drop-outs due to harmful outcome is not specified, unclear or combined.* |  |  |
| **C3. Does the study adequately address biased loss to follow-up?** | | | | **Unclear** |
|  | *Yes:* | *Unclear:* | *No:* |  |
|  | *Complete follow-up or drop-outs unlikely to introduce bias or adequately controlled.* | *Drop-outs/withdraws due to harmful outcome are not clearly reported.* | *Loss to follow-up affects the safety outcome and is not adequately controlled.* |  |
| **C4. Are the results based on an intention-to-treat analysis?** | | | | **yes** |
|  | *Yes:* | *Unclear:* | *No:* |  |
|  | *Results are based on a strict intention-to-treat analysis.* | *Not clear if an intention-to-treat analysis is performed. No strict intention-to-treat analysis.* | *Results are not based on intention-to-treat analysis (not done or not possible).* |  |
| **C. RISK OF BIAS DUE TO WITHDRAWALS OR LOSS OF FOLLOW-UP (ATTRITION)** | | | | **Unclear** |
|  | *Low:* | *Unclear:* | *High:* |  |
|  | *Plausible bias unlikely to seriously alter the results.* | *Plausible bias that raises some doubts about the results or when information on which to base risk of bias judgments is missing or poorly reported.* | *Plausible bias that seriously weakens confidence in the results.* |  |
| **Comments: unclear risk of bias according to data provided by sources** | | | | |
|  |  |  |  |  |
| **D. INFORMATION BIAS REGARDING THE HARMFULL OUTCOME** | | | | |
| **D1. Is the definition of the harmful outcome clearly stated?** | | | | **No** |
|  | *Yes:* | *No:* |  |  |
|  | *RCT: clear / standardized definition of the harmful outcome (e.g. diagnostic codes, clinical and laboratory data). Cohort studies: clear definition of the outcome. Case-control studies: clear definition of cases.* | *Definition of the harmful outcome not reported or that leads to confusion. Terms not well-constructed, wrong definition.* |  |  |
| **D2. If applicable, is the severity of the harmful outcome clearly stated?** | | | | **N/A** |
|  | *N/A:* | *Yes:* | *No:* |  |
|  | *Self evident severity (e.g. death).* | *Detailed degree of severity or reference to a known scale of severity or a new scale developed for the study.* | *Unclear degrees of severity or without clear boundaries between them.* |  |
| **D3. Was the blinding methods of participants regarding the intervention appropriate considering the nature of the harmful outcome?** | | | | **Unclear** |
|  | *Yes:* | *Unclear:* | *No:* |  |
|  | *Blinding ensured (and unlikely broken) or outcome not likely to be influenced by lack of blinding.* | *There is no sufficient information regarding the process of blinding or the outcome assessment.* | *No blinding (or incomplete blinding or risk of broken blinding) and outcome likely to be influenced by lack of blinding.* |  |
| **D4. Was the blinding methods of harmful outcome assessment appropriate considering the nature of the harmful outcome?** | | | | **Unclear** |
|  | *Yes:* | *Unclear:* | *No:* |  |
|  | *Blinding ensured (and unlikely broken) or outcome assessment not likely to be influenced by lack of blinding.* | *There is no sufficient information regarding the process of blinding of outcome assessment.* | *No blinding (or incomplete blinding or risk of broken blinding) and outcome likely to be influenced by lack of blinding.* |  |
| **D5. Was the duration of follow-up adequate to assess the harmful outcome?** | | | | **Unclear** |
|  | *Yes:* | *Unclear:* | *No:* |  |
|  | *Sufficient duration of follow-up to assess the outcome.* | *It is unclear whether the duration of follow-up is adequate.* | *Too short duration of follow-up.* |  |
| **D6. Was the methods for ascertaining the harmful outcome adequately constructed and equal for all participants?** | | | | **Unclear** |
|  | *Yes:* | *Unclear:* | *No:* |  |
|  | *Adequate or validated methods of outcome measurement for all participants. Clinical reactions medically confirmed by a physician. Minimized risk of misclassification or differential assessment, reporting or detection.  RCT : Active harmful outcome surveillance (prospective/retrospective case-record review, questionnaires, patient’s diary/checklist…) .* | *There is no or not sufficient information to clearly determine how information on harmful outcome is collected or the process of minimizing misclassification.* | *Substantial risk of misclassification of outcome or differential assessment, reporting or detection. Clinical reactions not medically confirmed. RCT : Passive harmful outcome surveillance (patient’s volunteer reporting).* |  |
| **D7. Are the number of harmful outcome and the number of patients with a harmful outcome reported in both treatment arms?** | | | | **Unclear** |
|  | *Yes:* | *Unclear:* | *No:* |  |
|  | *Numbers are reported. It is possible to calculate the rates of harmful outcome.* | *Confusion between the number of harmful outcomes or the number of patients with a harmful outcome, or general statements such as “5% of patients developed a harmful outcome”.* | *Neither the number of harmful outcomes nor the number of patients with a harmful outcome is reported. Or numbers are combining both treatment arms.* |  |
| **D8. Is the time frequency of harmful outcome assessment during the follow-up period appropriate?** | | | | **Unclear** |
|  | *Yes:* | *Unclear:* | *No:* |  |
|  | *For all study groups, the time frequency at which the harmful outcome is assessed is appropriate.* | *General statements such as “patients were routinely assessed for harmful outcomes”.* | *There is no regular collection of data on harmful outcomes during the study.* |  |
| **D9. Was the time between the exposure to a drug and the onset of the harmful outcome reported?** | | | | **No** |
|  | *Yes:* | *Unclear:* | *No:* |  |
|  | *The time between the drug exposure to the onset of harmful outcome is specified.* | *The authors do not report a clear time frame between drug exposure and harmful outcome.* | *The authors do not report the time between the drug exposure to the onset of harmful outcome.* |  |
| **D10. Was the process of determining that the harmful outcome is linked to the drug appropriate? Was the process blinded to the assigned treatment?** | | | | **Unclear** |
|  | *Yes:* | *Unclear:* | *No:* |  |
|  | *Methods for causality assessment are appropriate and, if applicable, made by investigators blinded to the intervention.* | *Unclear how the causality attribution is made. It is not clear who make the assessment or whether it is blinded to the assigned treatment.* | *Causality assessment is made by investigators not blinded to the intervention, or by participants or sponsors, or unblinding of treatment assignment precedes the decision to withdraw.* |  |
| **D. RISK OF BIAS ASSESSMENT FOR INFORMATION BIAS REGARDING THE HARMFULL OUTCOME** | | | | **Unclear** |
|  | *Low:* | *Unclear:* | *High:* |  |
|  | *Plausible bias unlikely to seriously alter the results.* | *Plausible bias that raises some doubts about the results or when information on which to base risk of bias judgments is missing or poorly reported.* | *Plausible bias that seriously weakens confidence in the results.* |  |
| **Comments: Unclear risk of bias** | | | | |
| **E. OTHER INFORMATION BIAS** | | | | |
| **E1. Is blinding of care givers during follow-up adequately performed in order to avoid differential care between study groups (performance bias)?** | | | | **Unclear** |
|  | *Yes:* | *Unclear:* | *No:* |  |
|  | *There is no risk of differential care or it is adequately addressed.* | *Unclear risk of bias due to differential care.* | *The bias due to differential care is not controlled.* |  |
| **E5. Does the study appear free of other information bias ?** | | | | **Unclear** |
|  | *Yes:* | *Unclear:* | *No:* |  |
|  | *The study appears to be free of other information bias.* | *Unclear presence of other information bias.* | *Additional source of other information bias.* |  |
| **E. RISK OF BIAS ASSESSMENT FOR OTHER INFORMATION BIAS** | | | | **Unclear** |
|  | *Low:* | *Unclear:* | *High:* |  |
|  | *Plausible bias unlikely to seriously alter the results.* | *Plausible bias that raises some doubts about the results or when information on which to base risk of bias judgments is missing or poorly reported.* | *Plausible bias that seriously weakens confidence in the results.* |  |
| **Comments: Unclear risk of bias** | | | | |
|  |  |  |  |  |
| **F. STATISTICAL METHODS TO CONTROL CONFOUNDING** | | | | |
| **F5. Does the study adequately address residual or unmeasured confounding?** | | | | **Unclear** |
|  | *Yes:* | *Unclear:* | *No:* |  |
|  | *The study adequately addresses residual or unmeasured confounding.* | *Unclear presence of residual or unmeasured confounding.* | *Residual or unmeasured confounding is likely to be important.* |  |
| **F. RISK OF BIAS ASSESSMENT FOR STATISTICAL METHODS TO CONTROL CONFOUNDING:** | | | | **Unclear** |
|  | *Low:* | *Unclear:* | *High:* |  |
|  | *Plausible bias unlikely to seriously alter the results.* | *Plausible bias that raises some doubts about the results or when information on which to base risk of bias judgments is missing or poorly reported.* | *Plausible bias that seriously weakens confidence in the results.* |  |
| **Comments: Unclear risk of bias** | | | | |
|  |  |  |  |  |
| **G. STATISTICAL METHODS EXCLUDING METHODS TO CONTROL CONFOUNDING** | | | | |
| **G1. Are the statistical methods used to analyze the harmful outcome appropriate?** | | | | **Unclear** |
|  | *Yes:* | *Unclear:* | *No:* |  |
|  | *Statistical techniques are appropriate to the data. If the distribution of the data (normal or not) is not described, it must be assumed that the estimates used were appropriate.* | *There is no description of the statistical techniques used, or the description is vague and not understandable.* | *The statistical techniques used are not appropriate.* |  |
| **G2. Is a survival analysis performed when there are individual differences in length of follow-up?** | | | | **Unclear** |
|  | *Yes:* | *Unclear:* | *No:* |  |
|  | *Follow-up is the same for all study patients, if not survival analysis is performed.* | *Unclear whether there are different lengths of follow-up or whether they are taken into account.* | *Differences of follow up were ignored.* |  |
| **G3. If applicable, is composite outcome of harms adequately constructed?** | | | | **N/A** |
| *N/A* | *Yes:* | *Unclear:* | *No:* |  |
|  | *Composite outcome appropriate.* | *Unclear whether composite outcome is appropriate.* | *Construction of composite not described or not appropriate.* |  |
| **G6.** **Are the results consistent in primary and secondary analyses? Are confounding effects consistent with known associations?** | | | | **Unclear** |
|  | *Yes:* | *Unclear:* | *No:* |  |
|  | *Consistency of primary, secondary analyses and consistency of confounding effects with known associations.* | *Not sufficient information to determine consistency.* | *No consistency of primary, secondary analyses or no consistency of confounding effects with known associations.* |  |
| **G. RISK OF BIAS ASSESSMENT FOR STATISTICAL METHODS EXCLUDING METHODS TO CONTROL CONFOUNDING:** | | | | **Unclear** |
|  | *Low:* | *Unclear:* | *High:* |  |
|  | *Plausible bias unlikely to seriously alter the results.* | *Plausible bias that raises some doubts about the results or when information on which to base risk of bias judgments is missing or poorly reported.* | *Plausible bias that seriously weakens confidence in the results.* |  |
| **Comments: Unclear risk of bias** | | | | |
|  | | | |  |
| **H. CONFLICT OF INTEREST** | | | | |
| **H1. Were the conflict of interest or sources of funding clearly acknowledged?** | | | | **Unclear** |
|  | *Yes:* | *No:* |  |  |
|  | *Potential sources of support are acknowledged.* | *No sources of funding reported or not sufficient information.* |  |  |
| **H2. Does the study appear free of conflicts of interest susceptible to have influenced design, analysis or reporting (selective reporting of outcome or analysis)?** | | | | **Unclear** |
|  | *Yes:* | *Unclear:* | *No:* |  |
|  | *No conflicts of interest or not susceptible to have influenced design, analysis or reporting.* | *It is unclear if there are conflicts of interest or if they are susceptible to have influenced design, analysis or reporting.* | *Conflicts of interest susceptible to have influenced design, analysis or reporting.* |  |
| **H. RISK OF BIAS ASSESSMENT FOR CONFLICT OF INTEREST** | | | | **Unclear** |
|  | *Low:* | *Unclear:* | *High:* |  |
|  | *Plausible bias unlikely to seriously alter the results.* | *Plausible bias that raises some doubts about the results or when information on which to base risk of bias judgments is missing or poorly reported.* | *Plausible bias that seriously weakens confidence in the results.* |  |
|  |  |  |  |  |
| **SUMMARY RISK-OF-BIAS ASSESSMENT FOR THE STUDY** | | | | |
| **RISK OF BIAS ASSESSMENT FOR THE STUDY** | | | | **Unclear** |
|  | *Low:* | *Unclear:* | *High:* |  |
|  | *Low risk of bias for all key domains.* | *Unclear risk of bias for one or more key domain.* | *High risk of bias for one or more key domains.* |  |

| **RISK OF BIAS ASSESSMENT CHECKLIST  FOR STUDIES INCLUDED IN SYSTEMATIC REVIEWS OF DRUG HARMS** | | | | |
| --- | --- | --- | --- | --- |
|  |  |  |  |  |
|  | **Study ID - Author** | Brioli et al (2020), GERMAIN, NCT02145598 |  |  |
|  |  |  |  |  |
| **A. STUDY DESIGN AND OBJECTIVES** | | | | |
| **A1. Are study objectives clearly specified and appropriate?** | | | | **Yes** |
|  | *Yes:* | *No:* |  |  |
|  | *Study objectives clearly specified and appropriate.* | *Study objectives are not clearly specified or not appropriate.* |  |  |
| **A2. Is study design clearly specified and appropriate?** | | | | **Yes** |
|  | *Yes:* | *No:* |  |  |
|  | *Study design clearly specified and appropriate.* | *Study design not clearly specified or not appropriate.* |  |  |
| **A3. Is the study design free of run-in/lead-in period before inclusion/randomization of participants?** | | | | **Yes** |
|  | *Yes:* | *Unclear:* | *No:* |  |
|  | *No run-in/lead-in period.* | *Not clear information.* | *Presence of a run-in/lead-in period.* |  |
| **A4. Cross-over designs: Is the study designed to adequately address carry-over effect?** | | | | **N/A** |
| *N/A* | *Yes:* | *Unclear:* | *No:* |  |
|  | *Carry-over effect absent or adequately addressed (randomized order and sufficiently long wash-out period.* | *Not clear information.* | *Carry-over effect not adequately addressed and susceptible to bias the results.* |  |
| **A. RISK OF BIAS ASSESSMENT FOR STUDY DESIGN AND OBJECTIVES** | | | | **Low** |
|  | *Low:* | *Unclear:* | *High:* |  |
|  | *Plausible bias unlikely to seriously alter the results.* | *Plausible bias that raises some doubts about the results or when information on which to base risk of bias judgments is missing or poorly reported.* | *Plausible bias that seriously weakens confidence in the results.* |  |
| **Comments: Low risk of bias according to data provided by sources** | | | | |
|  |  |  |  |  |
| **B. BIAS IN SELECTION OF SUBJECTS AND CONSTITUTION OF STUDY GROUPS** | | | | |
| **B1. Was the method used to generate the allocation sequence adequate as to produce comparable groups?** | | | | **Yes** |
|  | *Yes:* | *Unclear:* | *No:* |  |
|  | *Allocation methods are adequate to produce comparable groups.* | *Allocation methods are not clearly reported.* | *Allocation methods are not adequate (e.g. assignment to treatment by birth date, week day, etc.), groups are not comparable.* |  |
| **B2. Was the method used to conceal the allocation sequence adequate as to produce comparable groups?** | | | | **Yes** |
|  | *Yes:* | *Unclear:* | *No:* |  |
|  | *Concealment is adequate.* | *Concealment methods are not clearly reported and groups may not be comparable.* | *Concealment methods are not adequate, groups are not comparable.* |  |
| **B3. Are all the subjects recruited from the same source population?** | | | | **Yes** |
|  | *Yes:* | *Unclear:* | *No:* |  |
|  | *All the subjects recruited from the same source population.* | *Unclear if all the subjects recruited from the same source population.* | *All the subjects are not recruited from the same source population.* |  |
| **B4. Were inclusion and exclusion criteria implemented uniformly across study groups?** | | | | **Yes** |
|  | *Yes:* | *Unclear:* | *No:* |  |
|  | *Selection criteria uniformly implemented.* | *Unclear if selection criteria are uniformly implemented.* | *Selection criteria not uniformly implemented.* |  |
| **B8. Are baseline characteristics and prognostic factors comparable between different groups?** | | | | **Yes** |
|  | *Yes:* | *Unclear:* | *No:* |  |
|  | *RCT: Groups are comparable at baseline.* | *No description of baseline characteristics or only significance tests.* | *The groups are unbalanced at baseline.* |  |
|  | *Cohort studies: Groups are comparable at baseline or matched for the main prognostic factors.* |  |  |  |
| **B. RISK OF BIAS ASSESSMENT FOR SELECTION OF PARTICIPANTS AND CONSTITUTION OF STUDY GROUPS** | | | | **low** |
|  | *Low:* | *Unclear:* | *High:* |  |
|  | *Plausible bias unlikely to seriously alter the results.* | *Plausible bias that raises some doubts about the results or when information on which to base risk of bias judgments is missing or poorly reported.* | *Plausible bias that seriously weakens confidence in the results.* |  |
| **Comments: Low risk of bias according to data provided by sources** | | | | |
|  |  |  |  |  |
| **C. BIAS DUE TO WITHDRAWALS OR LOSS OF FOLLOW-UP (ATTRITION)** | | | | |
| **C1. Are the number of participants clearly reported throughout the study?** | | | | **Yes** |
|  | *Yes:* | *No:* |  |  |
|  | *Numbers of participants throughout the study are reported. Complete flow chart.* | *Numbers of patients at every stage is not clearly reported. Confusing information is reported regarding the number of participants. No or incomplete flow chart.* |  |  |
| **C2. Is the number of drop-outs/withdrawals due to harmful outcome clearly stated for each treatment arm?** | | | | **yes** |
|  | *Yes:* | *No:* |  |  |
|  | *The number of drop-outs due to harmful outcome is specified.* | *The number of drop-outs due to harmful outcome is not specified, unclear or combined.* |  |  |
| **C3. Does the study adequately address biased loss to follow-up?** | | | | **yes** |
|  | *Yes:* | *Unclear:* | *No:* |  |
|  | *Complete follow-up or drop-outs unlikely to introduce bias or adequately controlled.* | *Drop-outs/withdraws due to harmful outcome are not clearly reported.* | *Loss to follow-up affects the safety outcome and is not adequately controlled.* |  |
| **C4. Are the results based on an intention-to-treat analysis?** | | | | **yes** |
|  | *Yes:* | *Unclear:* | *No:* |  |
|  | *Results are based on a strict intention-to-treat analysis.* | *Not clear if an intention-to-treat analysis is performed. No strict intention-to-treat analysis.* | *Results are not based on intention-to-treat analysis (not done or not possible).* |  |
| **C. RISK OF BIAS DUE TO WITHDRAWALS OR LOSS OF FOLLOW-UP (ATTRITION)** | | | | **low** |
|  | *Low:* | *Unclear:* | *High:* |  |
|  | *Plausible bias unlikely to seriously alter the results.* | *Plausible bias that raises some doubts about the results or when information on which to base risk of bias judgments is missing or poorly reported.* | *Plausible bias that seriously weakens confidence in the results.* |  |
| **Comments: low risk of bias** | | | | |
|  |  |  |  |  |
| **D. INFORMATION BIAS REGARDING THE HARMFULL OUTCOME** | | | | |
| **D1. Is the definition of the harmful outcome clearly stated?** | | | | **No** |
|  | *Yes:* | *No:* |  |  |
|  | *RCT: clear / standardized definition of the harmful outcome (e.g. diagnostic codes, clinical and laboratory data). Cohort studies: clear definition of the outcome. Case-control studies: clear definition of cases.* | *Definition of the harmful outcome not reported or that leads to confusion. Terms not well-constructed, wrong definition.* |  |  |
| **D2. If applicable, is the severity of the harmful outcome clearly stated?** | | | | **No** |
|  | *N/A:* | *Yes:* | *No:* |  |
|  | *Self evident severity (e.g. death).* | *Detailed degree of severity or reference to a known scale of severity or a new scale developed for the study.* | *Unclear degrees of severity or without clear boundaries between them.* |  |
| **D3. Was the blinding methods of participants regarding the intervention appropriate considering the nature of the harmful outcome?** | | | | **Unclear** |
|  | *Yes:* | *Unclear:* | *No:* |  |
|  | *Blinding ensured (and unlikely broken) or outcome not likely to be influenced by lack of blinding.* | *There is no sufficient information regarding the process of blinding or the outcome assessment.* | *No blinding (or incomplete blinding or risk of broken blinding) and outcome likely to be influenced by lack of blinding.* |  |
| **D4. Was the blinding methods of harmful outcome assessment appropriate considering the nature of the harmful outcome?** | | | | **Unclear** |
|  | *Yes:* | *Unclear:* | *No:* |  |
|  | *Blinding ensured (and unlikely broken) or outcome assessment not likely to be influenced by lack of blinding.* | *There is no sufficient information regarding the process of blinding of outcome assessment.* | *No blinding (or incomplete blinding or risk of broken blinding) and outcome likely to be influenced by lack of blinding.* |  |
| **D5. Was the duration of follow-up adequate to assess the harmful outcome?** | | | | **Unclear** |
|  | *Yes:* | *Unclear:* | *No:* |  |
|  | *Sufficient duration of follow-up to assess the outcome.* | *It is unclear whether the duration of follow-up is adequate.* | *Too short duration of follow-up.* |  |
| **D6. Was the methods for ascertaining the harmful outcome adequately constructed and equal for all participants?** | | | | **Unclear** |
|  | *Yes:* | *Unclear:* | *No:* |  |
|  | *Adequate or validated methods of outcome measurement for all participants. Clinical reactions medically confirmed by a physician. Minimized risk of misclassification or differential assessment, reporting or detection.  RCT : Active harmful outcome surveillance (prospective/retrospective case-record review, questionnaires, patient’s diary/checklist…) .* | *There is no or not sufficient information to clearly determine how information on harmful outcome is collected or the process of minimizing misclassification.* | *Substantial risk of misclassification of outcome or differential assessment, reporting or detection. Clinical reactions not medically confirmed. RCT : Passive harmful outcome surveillance (patient’s volunteer reporting).* |  |
| **D7. Are the number of harmful outcome and the number of patients with a harmful outcome reported in both treatment arms?** | | | | **Yes** |
|  | *Yes:* | *Unclear:* | *No:* |  |
|  | *Numbers are reported. It is possible to calculate the rates of harmful outcome.* | *Confusion between the number of harmful outcomes or the number of patients with a harmful outcome, or general statements such as “5% of patients developed a harmful outcome”.* | *Neither the number of harmful outcomes nor the number of patients with a harmful outcome is reported. Or numbers are combining both treatment arms.* |  |
| **D8. Is the time frequency of harmful outcome assessment during the follow-up period appropriate?** | | | | **Unclear** |
|  | *Yes:* | *Unclear:* | *No:* |  |
|  | *For all study groups, the time frequency at which the harmful outcome is assessed is appropriate.* | *General statements such as “patients were routinely assessed for harmful outcomes”.* | *There is no regular collection of data on harmful outcomes during the study.* |  |
| **D9. Was the time between the exposure to a drug and the onset of the harmful outcome reported?** | | | | **Unclear** |
|  | *Yes:* | *Unclear:* | *No:* |  |
|  | *The time between the drug exposure to the onset of harmful outcome is specified.* | *The authors do not report a clear time frame between drug exposure and harmful outcome.* | *The authors do not report the time between the drug exposure to the onset of harmful outcome.* |  |
| **D10. Was the process of determining that the harmful outcome is linked to the drug appropriate? Was the process blinded to the assigned treatment?** | | | | **Unclear** |
|  | *Yes:* | *Unclear:* | *No:* |  |
|  | *Methods for causality assessment are appropriate and, if applicable, made by investigators blinded to the intervention.* | *Unclear how the causality attribution is made. It is not clear who make the assessment or whether it is blinded to the assigned treatment.* | *Causality assessment is made by investigators not blinded to the intervention, or by participants or sponsors, or unblinding of treatment assignment precedes the decision to withdraw.* |  |
| **D. RISK OF BIAS ASSESSMENT FOR INFORMATION BIAS REGARDING THE HARMFULL OUTCOME** | | | | **Unclear** |
|  | *Low:* | *Unclear:* | *High:* |  |
|  | *Plausible bias unlikely to seriously alter the results.* | *Plausible bias that raises some doubts about the results or when information on which to base risk of bias judgments is missing or poorly reported.* | *Plausible bias that seriously weakens confidence in the results.* |  |
| **Comments: Unclear risk of bias** | | | | |
|  |  |  |  |  |
| **E. OTHER INFORMATION BIAS** | | | | |
| **E1. Is blinding of care givers during follow-up adequately performed in order to avoid differential care between study groups (performance bias)?** | | | | **Yes** |
|  | *Yes:* | *Unclear:* | *No:* |  |
|  | *There is no risk of differential care or it is adequately addressed.* | *Unclear risk of bias due to differential care.* | *The bias due to differential care is not controlled.* |  |
| **E5. Does the study appear free of other information bias ?** | | | | **Yes** |
|  | *Yes:* | *Unclear:* | *No:* |  |
|  | *The study appears to be free of other information bias.* | *Unclear presence of other information bias.* | *Additional source of other information bias.* |  |
| **E. RISK OF BIAS ASSESSMENT FOR OTHER INFORMATION BIAS** | | | | **Low** |
|  | *Low:* | *Unclear:* | *High:* |  |
|  | *Plausible bias unlikely to seriously alter the results.* | *Plausible bias that raises some doubts about the results or when information on which to base risk of bias judgments is missing or poorly reported.* | *Plausible bias that seriously weakens confidence in the results.* |  |
| **Comments: Low of bias -> masking methods informations were well informative (pivotal +supp data).** | | | | |
|  |  |  |  |  |
| **F. STATISTICAL METHODS TO CONTROL CONFOUNDING** | | | | |
| **F5. Does the study adequately address residual or unmeasured confounding?** | | | | **Yes** |
|  | *Yes:* | *Unclear:* | *No:* |  |
|  | *The study adequately addresses residual or unmeasured confounding.* | *Unclear presence of residual or unmeasured confounding.* | *Residual or unmeasured confounding is likely to be important.* |  |
| **F. RISK OF BIAS ASSESSMENT FOR STATISTICAL METHODS TO CONTROL CONFOUNDING:** | | | | **Low** |
|  | *Low:* | *Unclear:* | *High:* |  |
|  | *Plausible bias unlikely to seriously alter the results.* | *Plausible bias that raises some doubts about the results or when information on which to base risk of bias judgments is missing or poorly reported.* | *Plausible bias that seriously weakens confidence in the results.* |  |
| **Comments: Low risk of bias->Statistical Analysis section is well informative ( stratification by quality of response after VMP induction (PR vs. at least very good partial response (VGPR)).** | | | | |
|  |  |  |  |  |
| **G. STATISTICAL METHODS EXCLUDING METHODS TO CONTROL CONFOUNDING** | | | | |
| **G1. Are the statistical methods used to analyze the harmful outcome appropriate?** | | | | **Unclear** |
|  | *Yes:* | *Unclear:* | *No:* |  |
|  | *Statistical techniques are appropriate to the data. If the distribution of the data (normal or not) is not described, it must be assumed that the estimates used were appropriate.* | *There is no description of the statistical techniques used, or the description is vague and not understandable.* | *The statistical techniques used are not appropriate.* |  |
| **G2. Is a survival analysis performed when there are individual differences in length of follow-up?** | | | | **Unclear** |
|  | *Yes:* | *Unclear:* | *No:* |  |
|  | *Follow-up is the same for all study patients, if not survival analysis is performed.* | *Unclear whether there are different lengths of follow-up or whether they are taken into account.* | *Differences of follow up were ignored.* |  |
| **G3. If applicable, is composite outcome of harms adequately constructed?** | | | | **N/A** |
| *N/A* | *Yes:* | *Unclear:* | *No:* |  |
|  | *Composite outcome appropriate.* | *Unclear whether composite outcome is appropriate.* | *Construction of composite not described or not appropriate.* |  |
| **G6.** **Are the results consistent in primary and secondary analyses? Are confounding effects consistent with known associations?** | | | | **unclear** |
|  | *Yes:* | *Unclear:* | *No:* |  |
|  | *Consistency of primary, secondary analyses and consistency of confounding effects with known associations.* | *Not sufficient information to determine consistency.* | *No consistency of primary, secondary analyses or no consistency of confounding effects with known associations.* |  |
| **G. RISK OF BIAS ASSESSMENT FOR STATISTICAL METHODS EXCLUDING METHODS TO CONTROL CONFOUNDING:** | | | | **Unclear** |
|  | *Low:* | *Unclear:* | *High:* |  |
|  | *Plausible bias unlikely to seriously alter the results.* | *Plausible bias that raises some doubts about the results or when information on which to base risk of bias judgments is missing or poorly reported.* | *Plausible bias that seriously weakens confidence in the results.* |  |
| **Comments: Unclear risk of bias** | | | | |
|  | | | |  |
| **H. CONFLICT OF INTEREST** | | | | |
| **H1. Were the conflict of interest or sources of funding clearly acknowledged?** | | | | **Yes** |
|  | *Yes:* | *No:* |  |  |
|  | *Potential sources of support are acknowledged.* | *No sources of funding reported or not sufficient information.* |  |  |
| **H2. Does the study appear free of conflicts of interest susceptible to have influenced design, analysis or reporting (selective reporting of outcome or analysis)?** | | | | **Yes** |
|  | *Yes:* | *Unclear:* | *No:* |  |
|  | *No conflicts of interest or not susceptible to have influenced design, analysis or reporting.* | *It is unclear if there are conflicts of interest or if they are susceptible to have influenced design, analysis or reporting.* | *Conflicts of interest susceptible to have influenced design, analysis or reporting.* |  |
| **H. RISK OF BIAS ASSESSMENT FOR CONFLICT OF INTEREST** | | | | **Yes** |
|  | *Low:* | *Unclear:* | *High:* |  |
|  | *Plausible bias unlikely to seriously alter the results.* | *Plausible bias that raises some doubts about the results or when information on which to base risk of bias judgments is missing or poorly reported.* | *Plausible bias that seriously weakens confidence in the results.* |  |
|  |  |  |  |  |
| **SUMMARY RISK-OF-BIAS ASSESSMENT FOR THE STUDY** | | | | |
| **RISK OF BIAS ASSESSMENT FOR THE STUDY** | | | | **Unclear** |
|  | *Low:* | *Unclear:* | *High:* |  |
|  | *Low risk of bias for all key domains.* | *Unclear risk of bias for one or more key domain.* | *High risk of bias for one or more key domains.* |  |

| **RISK OF BIAS ASSESSMENT CHECKLIST  FOR STUDIES INCLUDED IN SYSTEMATIC REVIEWS OF DRUG HARMS** | | | | |
| --- | --- | --- | --- | --- |
|  |  |  |  |  |
|  | **Study ID - Author** | Dimopoulos et al (2007), CC-5013-MM-010, NCT00424047 |  |  |
|  |  |  |  |  |
| **A. STUDY DESIGN AND OBJECTIVES** | | | | |
| **A1. Are study objectives clearly specified and appropriate?** | | | | **Yes** |
|  | *Yes:* | *No:* |  |  |
|  | *Study objectives clearly specified and appropriate.* | *Study objectives are not clearly specified or not appropriate.* |  |  |
| **A2. Is study design clearly specified and appropriate?** | | | | **Yes** |
|  | *Yes:* | *No:* |  |  |
|  | *Study design clearly specified and appropriate.* | *Study design not clearly specified or not appropriate.* |  |  |
| **A3. Is the study design free of run-in/lead-in period before inclusion/randomization of participants?** | | | | **Yes** |
|  | *Yes:* | *Unclear:* | *No:* |  |
|  | *No run-in/lead-in period.* | *Not clear information.* | *Presence of a run-in/lead-in period.* |  |
| **A4. Cross-over designs: Is the study designed to adequately address carry-over effect?** | | | | **N/A** |
| *N/A* | *Yes:* | *Unclear:* | *No:* |  |
|  | *Carry-over effect absent or adequately addressed (randomized order and sufficiently long wash-out period.* | *Not clear information.* | *Carry-over effect not adequately addressed and susceptible to bias the results.* |  |
| **A. RISK OF BIAS ASSESSMENT FOR STUDY DESIGN AND OBJECTIVES** | | | | **Low** |
|  | *Low:* | *Unclear:* | *High:* |  |
|  | *Plausible bias unlikely to seriously alter the results.* | *Plausible bias that raises some doubts about the results or when information on which to base risk of bias judgments is missing or poorly reported.* | *Plausible bias that seriously weakens confidence in the results.* |  |
| **Comments: Low risk of bias according to data provided by sources** | | | | |
|  |  |  |  |  |
| **B. BIAS IN SELECTION OF SUBJECTS AND CONSTITUTION OF STUDY GROUPS** | | | | |
| **B1. Was the method used to generate the allocation sequence adequate as to produce comparable groups?** | | | | **Yes** |
|  | *Yes:* | *Unclear:* | *No:* |  |
|  | *Allocation methods are adequate to produce comparable groups.* | *Allocation methods are not clearly reported.* | *Allocation methods are not adequate (e.g. assignment to treatment by birth date, week day, etc.), groups are not comparable.* |  |
| **B2. Was the method used to conceal the allocation sequence adequate as to produce comparable groups?** | | | | **Yes** |
|  | *Yes:* | *Unclear:* | *No:* |  |
|  | *Concealment is adequate.* | *Concealment methods are not clearly reported and groups may not be comparable.* | *Concealment methods are not adequate, groups are not comparable.* |  |
| **B3. Are all the subjects recruited from the same source population?** | | | | **Yes** |
|  | *Yes:* | *Unclear:* | *No:* |  |
|  | *All the subjects recruited from the same source population.* | *Unclear if all the subjects recruited from the same source population.* | *All the subjects are not recruited from the same source population.* |  |
| **B4. Were inclusion and exclusion criteria implemented uniformly across study groups?** | | | | **Yes** |
|  | *Yes:* | *Unclear:* | *No:* |  |
|  | *Selection criteria uniformly implemented.* | *Unclear if selection criteria are uniformly implemented.* | *Selection criteria not uniformly implemented.* |  |
| **B8. Are baseline characteristics and prognostic factors comparable between different groups?** | | | | **Yes** |
|  | *Yes:* | *Unclear:* | *No:* |  |
|  | *RCT: Groups are comparable at baseline.* | *No description of baseline characteristics or only significance tests.* | *The groups are unbalanced at baseline.* |  |
|  | *Cohort studies: Groups are comparable at baseline or matched for the main prognostic factors.* |  |  |  |
| **B. RISK OF BIAS ASSESSMENT FOR SELECTION OF PARTICIPANTS AND CONSTITUTION OF STUDY GROUPS** | | | | **Low** |
|  | *Low:* | *Unclear:* | *High:* |  |
|  | *Plausible bias unlikely to seriously alter the results.* | *Plausible bias that raises some doubts about the results or when information on which to base risk of bias judgments is missing or poorly reported.* | *Plausible bias that seriously weakens confidence in the results.* |  |
| **Comments: Low risk of bias according to data provided by sources** | | | | |
|  |  |  |  |  |
| **C. BIAS DUE TO WITHDRAWALS OR LOSS OF FOLLOW-UP (ATTRITION)** | | | | |
| **C1. Are the number of participants clearly reported throughout the study?** | | | | **Yes** |
|  | *Yes:* | *No:* |  |  |
|  | *Numbers of participants throughout the study are reported. Complete flow chart.* | *Numbers of patients at every stage is not clearly reported. Confusing information is reported regarding the number of participants. No or incomplete flow chart.* |  |  |
| **C2. Is the number of drop-outs/withdrawals due to harmful outcome clearly stated for each treatment arm?** | | | | **Yes** |
|  | *Yes:* | *No:* |  |  |
|  | *The number of drop-outs due to harmful outcome is specified.* | *The number of drop-outs due to harmful outcome is not specified, unclear or combined.* |  |  |
| **C3. Does the study adequately address biased loss to follow-up?** | | | | **yes** |
|  | *Yes:* | *Unclear:* | *No:* |  |
|  | *Complete follow-up or drop-outs unlikely to introduce bias or adequately controlled.* | *Drop-outs/withdraws due to harmful outcome are not clearly reported.* | *Loss to follow-up affects the safety outcome and is not adequately controlled.* |  |
| **C4. Are the results based on an intention-to-treat analysis?** | | | | **yes** |
|  | *Yes:* | *Unclear:* | *No:* |  |
|  | *Results are based on a strict intention-to-treat analysis.* | *Not clear if an intention-to-treat analysis is performed. No strict intention-to-treat analysis.* | *Results are not based on intention-to-treat analysis (not done or not possible).* |  |
| **C. RISK OF BIAS DUE TO WITHDRAWALS OR LOSS OF FOLLOW-UP (ATTRITION)** | | | | **Low** |
|  | *Low:* | *Unclear:* | *High:* |  |
|  | *Plausible bias unlikely to seriously alter the results.* | *Plausible bias that raises some doubts about the results or when information on which to base risk of bias judgments is missing or poorly reported.* | *Plausible bias that seriously weakens confidence in the results.* |  |
| **Comments: Low risk of bias according to data provided by study sources** | | | | |
|  |  |  |  |  |
| **D. INFORMATION BIAS REGARDING THE HARMFULL OUTCOME** | | | | |
| **D1. Is the definition of the harmful outcome clearly stated?** | | | | **No** |
|  | *Yes:* | *No:* |  |  |
|  | *RCT: clear / standardized definition of the harmful outcome (e.g. diagnostic codes, clinical and laboratory data). Cohort studies: clear definition of the outcome. Case-control studies: clear definition of cases.* | *Definition of the harmful outcome not reported or that leads to confusion. Terms not well-constructed, wrong definition.* |  |  |
| **D2. If applicable, is the severity of the harmful outcome clearly stated?** | | | | **No** |
|  | *N/A:* | *Yes:* | *No:* |  |
|  | *Self evident severity (e.g. death).* | *Detailed degree of severity or reference to a known scale of severity or a new scale developed for the study.* | *Unclear degrees of severity or without clear boundaries between them.* |  |
| **D3. Was the blinding methods of participants regarding the intervention appropriate considering the nature of the harmful outcome?** | | | | **Unclear** |
|  | *Yes:* | *Unclear:* | *No:* |  |
|  | *Blinding ensured (and unlikely broken) or outcome not likely to be influenced by lack of blinding.* | *There is no sufficient information regarding the process of blinding or the outcome assessment.* | *No blinding (or incomplete blinding or risk of broken blinding) and outcome likely to be influenced by lack of blinding.* |  |
| **D4. Was the blinding methods of harmful outcome assessment appropriate considering the nature of the harmful outcome?** | | | | **Unclear** |
|  | *Yes:* | *Unclear:* | *No:* |  |
|  | *Blinding ensured (and unlikely broken) or outcome assessment not likely to be influenced by lack of blinding.* | *There is no sufficient information regarding the process of blinding of outcome assessment.* | *No blinding (or incomplete blinding or risk of broken blinding) and outcome likely to be influenced by lack of blinding.* |  |
| **D5. Was the duration of follow-up adequate to assess the harmful outcome?** | | | | **Unclear** |
|  | *Yes:* | *Unclear:* | *No:* |  |
|  | *Sufficient duration of follow-up to assess the outcome.* | *It is unclear whether the duration of follow-up is adequate.* | *Too short duration of follow-up.* |  |
| **D6. Was the methods for ascertaining the harmful outcome adequately constructed and equal for all participants?** | | | | **Unclear** |
|  | *Yes:* | *Unclear:* | *No:* |  |
|  | *Adequate or validated methods of outcome measurement for all participants. Clinical reactions medically confirmed by a physician. Minimized risk of misclassification or differential assessment, reporting or detection.  RCT : Active harmful outcome surveillance (prospective/retrospective case-record review, questionnaires, patient’s diary/checklist…) .* | *There is no or not sufficient information to clearly determine how information on harmful outcome is collected or the process of minimizing misclassification.* | *Substantial risk of misclassification of outcome or differential assessment, reporting or detection. Clinical reactions not medically confirmed. RCT : Passive harmful outcome surveillance (patient’s volunteer reporting).* |  |
| **D7. Are the number of harmful outcome and the number of patients with a harmful outcome reported in both treatment arms?** | | | | **No** |
|  | *Yes:* | *Unclear:* | *No:* |  |
|  | *Numbers are reported. It is possible to calculate the rates of harmful outcome.* | *Confusion between the number of harmful outcomes or the number of patients with a harmful outcome, or general statements such as “5% of patients developed a harmful outcome”.* | *Neither the number of harmful outcomes nor the number of patients with a harmful outcome is reported. Or numbers are combining both treatment arms.* |  |
| **D8. Is the time frequency of harmful outcome assessment during the follow-up period appropriate?** | | | | **Unclear** |
|  | *Yes:* | *Unclear:* | *No:* |  |
|  | *For all study groups, the time frequency at which the harmful outcome is assessed is appropriate.* | *General statements such as “patients were routinely assessed for harmful outcomes”.* | *There is no regular collection of data on harmful outcomes during the study.* |  |
| **D9. Was the time between the exposure to a drug and the onset of the harmful outcome reported?** | | | | **No** |
|  | *Yes:* | *Unclear:* | *No:* |  |
|  | *The time between the drug exposure to the onset of harmful outcome is specified.* | *The authors do not report a clear time frame between drug exposure and harmful outcome.* | *The authors do not report the time between the drug exposure to the onset of harmful outcome.* |  |
| **D10. Was the process of determining that the harmful outcome is linked to the drug appropriate? Was the process blinded to the assigned treatment?** | | | | **No** |
|  | *Yes:* | *Unclear:* | *No:* |  |
|  | *Methods for causality assessment are appropriate and, if applicable, made by investigators blinded to the intervention.* | *Unclear how the causality attribution is made. It is not clear who make the assessment or whether it is blinded to the assigned treatment.* | *Causality assessment is made by investigators not blinded to the intervention, or by participants or sponsors, or unblinding of treatment assignment precedes the decision to withdraw.* |  |
| **D. RISK OF BIAS ASSESSMENT FOR INFORMATION BIAS REGARDING THE HARMFULL OUTCOME** | | | | **Unclear** |
|  | *Low:* | *Unclear:* | *High:* |  |
|  | *Plausible bias unlikely to seriously alter the results.* | *Plausible bias that raises some doubts about the results or when information on which to base risk of bias judgments is missing or poorly reported.* | *Plausible bias that seriously weakens confidence in the results.* |  |
| **Comments: Unclear risk of bias** | | | | |
|  |  |  |  |  |
| **E. OTHER INFORMATION BIAS** | | | | |
| **E1. Is blinding of care givers during follow-up adequately performed in order to avoid differential care between study groups (performance bias)?** | | | | **Yes** |
|  | *Yes:* | *Unclear:* | *No:* |  |
|  | *There is no risk of differential care or it is adequately addressed.* | *Unclear risk of bias due to differential care.* | *The bias due to differential care is not controlled.* |  |
| **E5. Does the study appear free of other information bias ?** | | | | **Yes** |
|  | *Yes:* | *Unclear:* | *No:* |  |
|  | *The study appears to be free of other information bias.* | *Unclear presence of other information bias.* | *Additional source of other information bias.* |  |
| **E. RISK OF BIAS ASSESSMENT FOR OTHER INFORMATION BIAS** | | | | **Low** |
|  | *Low:* | *Unclear:* | *High:* |  |
|  | *Plausible bias unlikely to seriously alter the results.* | *Plausible bias that raises some doubts about the results or when information on which to base risk of bias judgments is missing or poorly reported.* | *Plausible bias that seriously weakens confidence in the results.* |  |
| **Comments: Low of bias -> masking methods informations were well informative.** | | | | |
|  |  |  |  |  |
| **F. STATISTICAL METHODS TO CONTROL CONFOUNDING** | | | | |
| **F5. Does the study adequately address residual or unmeasured confounding?** | | | | **Yes** |
|  | *Yes:* | *Unclear:* | *No:* |  |
|  | *The study adequately addresses residual or unmeasured confounding.* | *Unclear presence of residual or unmeasured confounding.* | *Residual or unmeasured confounding is likely to be important.* |  |
| **F. RISK OF BIAS ASSESSMENT FOR STATISTICAL METHODS TO CONTROL CONFOUNDING:** | | | | **Low** |
|  | *Low:* | *Unclear:* | *High:* |  |
|  | *Plausible bias unlikely to seriously alter the results.* | *Plausible bias that raises some doubts about the results or when information on which to base risk of bias judgments is missing or poorly reported.* | *Plausible bias that seriously weakens confidence in the results.* |  |
| **Comments: Low risk of bias->Statistical Analysis section is well informative ( stratification model according to the baseline serum β2-microglobulin level (<2.5 mg per liter or ≥2.5 mg per liter), previous stem-cell transplantation (none or ≥1), and the number of previous antimyeloma regimens (1 or ≥2).** | | | | |
|  |  |  |  |  |
| **G. STATISTICAL METHODS EXCLUDING METHODS TO CONTROL CONFOUNDING** | | | | |
| **G1. Are the statistical methods used to analyze the harmful outcome appropriate?** | | | | **Unclear** |
|  | *Yes:* | *Unclear:* | *No:* |  |
|  | *Statistical techniques are appropriate to the data. If the distribution of the data (normal or not) is not described, it must be assumed that the estimates used were appropriate.* | *There is no description of the statistical techniques used, or the description is vague and not understandable.* | *The statistical techniques used are not appropriate.* |  |
| **G2. Is a survival analysis performed when there are individual differences in length of follow-up?** | | | | **Unclear** |
|  | *Yes:* | *Unclear:* | *No:* |  |
|  | *Follow-up is the same for all study patients, if not survival analysis is performed.* | *Unclear whether there are different lengths of follow-up or whether they are taken into account.* | *Differences of follow up were ignored.* |  |
| **G3. If applicable, is composite outcome of harms adequately constructed?** | | | | **N/A** |
| *N/A* | *Yes:* | *Unclear:* | *No:* |  |
|  | *Composite outcome appropriate.* | *Unclear whether composite outcome is appropriate.* | *Construction of composite not described or not appropriate.* |  |
| **G6.** **Are the results consistent in primary and secondary analyses? Are confounding effects consistent with known associations?** | | | | **Yes** |
|  | *Yes:* | *Unclear:* | *No:* |  |
|  | *Consistency of primary, secondary analyses and consistency of confounding effects with known associations.* | *Not sufficient information to determine consistency.* | *No consistency of primary, secondary analyses or no consistency of confounding effects with known associations.* |  |
| **G. RISK OF BIAS ASSESSMENT FOR STATISTICAL METHODS EXCLUDING METHODS TO CONTROL CONFOUNDING:** | | | | **Unclear** |
|  | *Low:* | *Unclear:* | *High:* |  |
|  | *Plausible bias unlikely to seriously alter the results.* | *Plausible bias that raises some doubts about the results or when information on which to base risk of bias judgments is missing or poorly reported.* | *Plausible bias that seriously weakens confidence in the results.* |  |
| **Comments: Unclear risk of bias** | | | | |
|  | | | |  |
| **H. CONFLICT OF INTEREST** | | | | |
| **H1. Were the conflict of interest or sources of funding clearly acknowledged?** | | | | **Yes** |
|  | *Yes:* | *No:* |  |  |
|  | *Potential sources of support are acknowledged.* | *No sources of funding reported or not sufficient information.* |  |  |
| **H2. Does the study appear free of conflicts of interest susceptible to have influenced design, analysis or reporting (selective reporting of outcome or analysis)?** | | | | **Yes** |
|  | *Yes:* | *Unclear:* | *No:* |  |
|  | *No conflicts of interest or not susceptible to have influenced design, analysis or reporting.* | *It is unclear if there are conflicts of interest or if they are susceptible to have influenced design, analysis or reporting.* | *Conflicts of interest susceptible to have influenced design, analysis or reporting.* |  |
| **H. RISK OF BIAS ASSESSMENT FOR CONFLICT OF INTEREST** | | | | **Low** |
|  | *Low:* | *Unclear:* | *High:* |  |
|  | *Plausible bias unlikely to seriously alter the results.* | *Plausible bias that raises some doubts about the results or when information on which to base risk of bias judgments is missing or poorly reported.* | *Plausible bias that seriously weakens confidence in the results.* |  |
|  |  |  |  |  |
| **SUMMARY RISK-OF-BIAS ASSESSMENT FOR THE STUDY** | | | | |
| **RISK OF BIAS ASSESSMENT FOR THE STUDY** | | | | **Unclear** |
|  | *Low:* | *Unclear:* | *High:* |  |
|  | *Low risk of bias for all key domains.* | *Unclear risk of bias for one or more key domain.* | *High risk of bias for one or more key domains.* |  |

| **RISK OF BIAS ASSESSMENT CHECKLIST  FOR STUDIES INCLUDED IN SYSTEMATIC REVIEWS OF DRUG HARMS** | | | | |
| --- | --- | --- | --- | --- |
|  |  |  |  |  |
|  | **Study ID - Author** | Dimopoulos et al (2013), MMY-2045/SEQUENTIAL, NCT00908232 |  |  |
|  |  |  |  |  |
| **A. STUDY DESIGN AND OBJECTIVES** | | | | |
| **A1. Are study objectives clearly specified and appropriate?** | | | | **Yes** |
|  | *Yes:* | *No:* |  |  |
|  | *Study objectives clearly specified and appropriate.* | *Study objectives are not clearly specified or not appropriate.* |  |  |
| **A2. Is study design clearly specified and appropriate?** | | | | **Yes** |
|  | *Yes:* | *No:* |  |  |
|  | *Study design clearly specified and appropriate.* | *Study design not clearly specified or not appropriate.* |  |  |
| **A3. Is the study design free of run-in/lead-in period before inclusion/randomization of participants?** | | | | **Yes** |
|  | *Yes:* | *Unclear:* | *No:* |  |
|  | *No run-in/lead-in period.* | *Not clear information.* | *Presence of a run-in/lead-in period.* |  |
| **A4. Cross-over designs: Is the study designed to adequately address carry-over effect?** | | | | **N/A** |
| *N/A* | *Yes:* | *Unclear:* | *No:* |  |
|  | *Carry-over effect absent or adequately addressed (randomized order and sufficiently long wash-out period.* | *Not clear information.* | *Carry-over effect not adequately addressed and susceptible to bias the results.* |  |
| **A. RISK OF BIAS ASSESSMENT FOR STUDY DESIGN AND OBJECTIVES** | | | | **Low** |
|  | *Low:* | *Unclear:* | *High:* |  |
|  | *Plausible bias unlikely to seriously alter the results.* | *Plausible bias that raises some doubts about the results or when information on which to base risk of bias judgments is missing or poorly reported.* | *Plausible bias that seriously weakens confidence in the results.* |  |
| **Comments: Low risk of bias according to data provided by sources** | | | | |
|  |  |  |  |  |
| **B. BIAS IN SELECTION OF SUBJECTS AND CONSTITUTION OF STUDY GROUPS** | | | | |
| **B1. Was the method used to generate the allocation sequence adequate as to produce comparable groups?** | | | | **Unclear** |
|  | *Yes:* | *Unclear:* | *No:* |  |
|  | *Allocation methods are adequate to produce comparable groups.* | *Allocation methods are not clearly reported.* | *Allocation methods are not adequate (e.g. assignment to treatment by birth date, week day, etc.), groups are not comparable.* |  |
| **B2. Was the method used to conceal the allocation sequence adequate as to produce comparable groups?** | | | | **Unclear** |
|  | *Yes:* | *Unclear:* | *No:* |  |
|  | *Concealment is adequate.* | *Concealment methods are not clearly reported and groups may not be comparable.* | *Concealment methods are not adequate, groups are not comparable.* |  |
| **B3. Are all the subjects recruited from the same source population?** | | | | **Yes** |
|  | *Yes:* | *Unclear:* | *No:* |  |
|  | *All the subjects recruited from the same source population.* | *Unclear if all the subjects recruited from the same source population.* | *All the subjects are not recruited from the same source population.* |  |
| **B4. Were inclusion and exclusion criteria implemented uniformly across study groups?** | | | | **Yes** |
|  | *Yes:* | *Unclear:* | *No:* |  |
|  | *Selection criteria uniformly implemented.* | *Unclear if selection criteria are uniformly implemented.* | *Selection criteria not uniformly implemented.* |  |
| **B8. Are baseline characteristics and prognostic factors comparable between different groups?** | | | | **Unclear** |
|  | *Yes:* | *Unclear:* | *No:* |  |
|  | *RCT: Groups are comparable at baseline.* | *No description of baseline characteristics or only significance tests.* | *The groups are unbalanced at baseline.* |  |
|  | *Cohort studies: Groups are comparable at baseline or matched for the main prognostic factors.* |  |  |  |
| **B. RISK OF BIAS ASSESSMENT FOR SELECTION OF PARTICIPANTS AND CONSTITUTION OF STUDY GROUPS** | | | | **Unclear** |
|  | *Low:* | *Unclear:* | *High:* |  |
|  | *Plausible bias unlikely to seriously alter the results.* | *Plausible bias that raises some doubts about the results or when information on which to base risk of bias judgments is missing or poorly reported.* | *Plausible bias that seriously weakens confidence in the results.* |  |
| **Comments: unclear risk of bias according to data provided by sources** | | | | |
|  |  |  |  |  |
| **C. BIAS DUE TO WITHDRAWALS OR LOSS OF FOLLOW-UP (ATTRITION)** | | | | |
| **C1. Are the number of participants clearly reported throughout the study?** | | | | **Yes** |
|  | *Yes:* | *No:* |  |  |
|  | *Numbers of participants throughout the study are reported. Complete flow chart.* | *Numbers of patients at every stage is not clearly reported. Confusing information is reported regarding the number of participants. No or incomplete flow chart.* |  |  |
| **C2. Is the number of drop-outs/withdrawals due to harmful outcome clearly stated for each treatment arm?** | | | | **Yes** |
|  | *Yes:* | *No:* |  |  |
|  | *The number of drop-outs due to harmful outcome is specified.* | *The number of drop-outs due to harmful outcome is not specified, unclear or combined.* |  |  |
| **C3. Does the study adequately address biased loss to follow-up?** | | | | **yes** |
|  | *Yes:* | *Unclear:* | *No:* |  |
|  | *Complete follow-up or drop-outs unlikely to introduce bias or adequately controlled.* | *Drop-outs/withdraws due to harmful outcome are not clearly reported.* | *Loss to follow-up affects the safety outcome and is not adequately controlled.* |  |
| **C4. Are the results based on an intention-to-treat analysis?** | | | | **yes** |
|  | *Yes:* | *Unclear:* | *No:* |  |
|  | *Results are based on a strict intention-to-treat analysis.* | *Not clear if an intention-to-treat analysis is performed. No strict intention-to-treat analysis.* | *Results are not based on intention-to-treat analysis (not done or not possible).* |  |
| **C. RISK OF BIAS DUE TO WITHDRAWALS OR LOSS OF FOLLOW-UP (ATTRITION)** | | | | **Low** |
|  | *Low:* | *Unclear:* | *High:* |  |
|  | *Plausible bias unlikely to seriously alter the results.* | *Plausible bias that raises some doubts about the results or when information on which to base risk of bias judgments is missing or poorly reported.* | *Plausible bias that seriously weakens confidence in the results.* |  |
| **Comments: Low risk of bias according to data provided by study sources** | | | | |
|  |  |  |  |  |
| **D. INFORMATION BIAS REGARDING THE HARMFULL OUTCOME** | | | | |
| **D1. Is the definition of the harmful outcome clearly stated?** | | | | **No** |
|  | *Yes:* | *No:* |  |  |
|  | *RCT: clear / standardized definition of the harmful outcome (e.g. diagnostic codes, clinical and laboratory data). Cohort studies: clear definition of the outcome. Case-control studies: clear definition of cases.* | *Definition of the harmful outcome not reported or that leads to confusion. Terms not well-constructed, wrong definition.* |  |  |
| **D2. If applicable, is the severity of the harmful outcome clearly stated?** | | | | **No** |
|  | *N/A:* | *Yes:* | *No:* |  |
|  | *Self evident severity (e.g. death).* | *Detailed degree of severity or reference to a known scale of severity or a new scale developed for the study.* | *Unclear degrees of severity or without clear boundaries between them.* |  |
| **D3. Was the blinding methods of participants regarding the intervention appropriate considering the nature of the harmful outcome?** | | | | **Unclear** |
|  | *Yes:* | *Unclear:* | *No:* |  |
|  | *Blinding ensured (and unlikely broken) or outcome not likely to be influenced by lack of blinding.* | *There is no sufficient information regarding the process of blinding or the outcome assessment.* | *No blinding (or incomplete blinding or risk of broken blinding) and outcome likely to be influenced by lack of blinding.* |  |
| **D4. Was the blinding methods of harmful outcome assessment appropriate considering the nature of the harmful outcome?** | | | | **Unclear** |
|  | *Yes:* | *Unclear:* | *No:* |  |
|  | *Blinding ensured (and unlikely broken) or outcome assessment not likely to be influenced by lack of blinding.* | *There is no sufficient information regarding the process of blinding of outcome assessment.* | *No blinding (or incomplete blinding or risk of broken blinding) and outcome likely to be influenced by lack of blinding.* |  |
| **D5. Was the duration of follow-up adequate to assess the harmful outcome?** | | | | **Unclear** |
|  | *Yes:* | *Unclear:* | *No:* |  |
|  | *Sufficient duration of follow-up to assess the outcome.* | *It is unclear whether the duration of follow-up is adequate.* | *Too short duration of follow-up.* |  |
| **D6. Was the methods for ascertaining the harmful outcome adequately constructed and equal for all participants?** | | | | **Unclear** |
|  | *Yes:* | *Unclear:* | *No:* |  |
|  | *Adequate or validated methods of outcome measurement for all participants. Clinical reactions medically confirmed by a physician. Minimized risk of misclassification or differential assessment, reporting or detection.  RCT : Active harmful outcome surveillance (prospective/retrospective case-record review, questionnaires, patient’s diary/checklist…) .* | *There is no or not sufficient information to clearly determine how information on harmful outcome is collected or the process of minimizing misclassification.* | *Substantial risk of misclassification of outcome or differential assessment, reporting or detection. Clinical reactions not medically confirmed. RCT : Passive harmful outcome surveillance (patient’s volunteer reporting).* |  |
| **D7. Are the number of harmful outcome and the number of patients with a harmful outcome reported in both treatment arms?** | | | | **No** |
|  | *Yes:* | *Unclear:* | *No:* |  |
|  | *Numbers are reported. It is possible to calculate the rates of harmful outcome.* | *Confusion between the number of harmful outcomes or the number of patients with a harmful outcome, or general statements such as “5% of patients developed a harmful outcome”.* | *Neither the number of harmful outcomes nor the number of patients with a harmful outcome is reported. Or numbers are combining both treatment arms.* |  |
| **D8. Is the time frequency of harmful outcome assessment during the follow-up period appropriate?** | | | | **Unclear** |
|  | *Yes:* | *Unclear:* | *No:* |  |
|  | *For all study groups, the time frequency at which the harmful outcome is assessed is appropriate.* | *General statements such as “patients were routinely assessed for harmful outcomes”.* | *There is no regular collection of data on harmful outcomes during the study.* |  |
| **D9. Was the time between the exposure to a drug and the onset of the harmful outcome reported?** | | | | **No** |
|  | *Yes:* | *Unclear:* | *No:* |  |
|  | *The time between the drug exposure to the onset of harmful outcome is specified.* | *The authors do not report a clear time frame between drug exposure and harmful outcome.* | *The authors do not report the time between the drug exposure to the onset of harmful outcome.* |  |
| **D10. Was the process of determining that the harmful outcome is linked to the drug appropriate? Was the process blinded to the assigned treatment?** | | | | **No** |
|  | *Yes:* | *Unclear:* | *No:* |  |
|  | *Methods for causality assessment are appropriate and, if applicable, made by investigators blinded to the intervention.* | *Unclear how the causality attribution is made. It is not clear who make the assessment or whether it is blinded to the assigned treatment.* | *Causality assessment is made by investigators not blinded to the intervention, or by participants or sponsors, or unblinding of treatment assignment precedes the decision to withdraw.* |  |
| **D. RISK OF BIAS ASSESSMENT FOR INFORMATION BIAS REGARDING THE HARMFULL OUTCOME** | | | | **Unclear** |
|  | *Low:* | *Unclear:* | *High:* |  |
|  | *Plausible bias unlikely to seriously alter the results.* | *Plausible bias that raises some doubts about the results or when information on which to base risk of bias judgments is missing or poorly reported.* | *Plausible bias that seriously weakens confidence in the results.* |  |
| **Comments: Unclear risk of bias** | | | | |
|  |  |  |  |  |
| **E. OTHER INFORMATION BIAS** | | | | |
| **E1. Is blinding of care givers during follow-up adequately performed in order to avoid differential care between study groups (performance bias)?** | | | | **Unclear** |
|  | *Yes:* | *Unclear:* | *No:* |  |
|  | *There is no risk of differential care or it is adequately addressed.* | *Unclear risk of bias due to differential care.* | *The bias due to differential care is not controlled.* |  |
| **E5. Does the study appear free of other information bias ?** | | | | **Yes** |
|  | *Yes:* | *Unclear:* | *No:* |  |
|  | *The study appears to be free of other information bias.* | *Unclear presence of other information bias.* | *Additional source of other information bias.* |  |
| **E. RISK OF BIAS ASSESSMENT FOR OTHER INFORMATION BIAS** | | | | **Unclear** |
|  | *Low:* | *Unclear:* | *High:* |  |
|  | *Plausible bias unlikely to seriously alter the results.* | *Plausible bias that raises some doubts about the results or when information on which to base risk of bias judgments is missing or poorly reported.* | *Plausible bias that seriously weakens confidence in the results.* |  |
| **Comments: unclear of bias -> open-label** | | | | |
|  |  |  |  |  |
| **F. STATISTICAL METHODS TO CONTROL CONFOUNDING** | | | | |
| **F5. Does the study adequately address residual or unmeasured confounding?** | | | | **Unclear** |
|  | *Yes:* | *Unclear:* | *No:* |  |
|  | *The study adequately addresses residual or unmeasured confounding.* | *Unclear presence of residual or unmeasured confounding.* | *Residual or unmeasured confounding is likely to be important.* |  |
| **F. RISK OF BIAS ASSESSMENT FOR STATISTICAL METHODS TO CONTROL CONFOUNDING:** | | | | **Unclear** |
|  | *Low:* | *Unclear:* | *High:* |  |
|  | *Plausible bias unlikely to seriously alter the results.* | *Plausible bias that raises some doubts about the results or when information on which to base risk of bias judgments is missing or poorly reported.* | *Plausible bias that seriously weakens confidence in the results.* |  |
| **Comments: unclear risk of bias** | | | | |
|  |  |  |  |  |
| **G. STATISTICAL METHODS EXCLUDING METHODS TO CONTROL CONFOUNDING** | | | | |
| **G1. Are the statistical methods used to analyze the harmful outcome appropriate?** | | | | **Unclear** |
|  | *Yes:* | *Unclear:* | *No:* |  |
|  | *Statistical techniques are appropriate to the data. If the distribution of the data (normal or not) is not described, it must be assumed that the estimates used were appropriate.* | *There is no description of the statistical techniques used, or the description is vague and not understandable.* | *The statistical techniques used are not appropriate.* |  |
| **G2. Is a survival analysis performed when there are individual differences in length of follow-up?** | | | | **Unclear** |
|  | *Yes:* | *Unclear:* | *No:* |  |
|  | *Follow-up is the same for all study patients, if not survival analysis is performed.* | *Unclear whether there are different lengths of follow-up or whether they are taken into account.* | *Differences of follow up were ignored.* |  |
| **G3. If applicable, is composite outcome of harms adequately constructed?** | | | | **N/A** |
| *N/A* | *Yes:* | *Unclear:* | *No:* |  |
|  | *Composite outcome appropriate.* | *Unclear whether composite outcome is appropriate.* | *Construction of composite not described or not appropriate.* |  |
| **G6.** **Are the results consistent in primary and secondary analyses? Are confounding effects consistent with known associations?** | | | | **Unclear** |
|  | *Yes:* | *Unclear:* | *No:* |  |
|  | *Consistency of primary, secondary analyses and consistency of confounding effects with known associations.* | *Not sufficient information to determine consistency.* | *No consistency of primary, secondary analyses or no consistency of confounding effects with known associations.* |  |
| **G. RISK OF BIAS ASSESSMENT FOR STATISTICAL METHODS EXCLUDING METHODS TO CONTROL CONFOUNDING:** | | | | **Unclear** |
|  | *Low:* | *Unclear:* | *High:* |  |
|  | *Plausible bias unlikely to seriously alter the results.* | *Plausible bias that raises some doubts about the results or when information on which to base risk of bias judgments is missing or poorly reported.* | *Plausible bias that seriously weakens confidence in the results.* |  |
| **Comments: Unclear risk of bias** | | | | |
|  | | | |  |
| **H. CONFLICT OF INTEREST** | | | | |
| **H1. Were the conflict of interest or sources of funding clearly acknowledged?** | | | | **Yes** |
|  | *Yes:* | *No:* |  |  |
|  | *Potential sources of support are acknowledged.* | *No sources of funding reported or not sufficient information.* |  |  |
| **H2. Does the study appear free of conflicts of interest susceptible to have influenced design, analysis or reporting (selective reporting of outcome or analysis)?** | | | | **Yes** |
|  | *Yes:* | *Unclear:* | *No:* |  |
|  | *No conflicts of interest or not susceptible to have influenced design, analysis or reporting.* | *It is unclear if there are conflicts of interest or if they are susceptible to have influenced design, analysis or reporting.* | *Conflicts of interest susceptible to have influenced design, analysis or reporting.* |  |
| **H. RISK OF BIAS ASSESSMENT FOR CONFLICT OF INTEREST** | | | | **Low** |
|  | *Low:* | *Unclear:* | *High:* |  |
|  | *Plausible bias unlikely to seriously alter the results.* | *Plausible bias that raises some doubts about the results or when information on which to base risk of bias judgments is missing or poorly reported.* | *Plausible bias that seriously weakens confidence in the results.* |  |
|  |  |  |  |  |
| **SUMMARY RISK-OF-BIAS ASSESSMENT FOR THE STUDY** | | | | |
| **RISK OF BIAS ASSESSMENT FOR THE STUDY** | | | | **Unclear** |
|  | *Low:* | *Unclear:* | *High:* |  |
|  | *Low risk of bias for all key domains.* | *Unclear risk of bias for one or more key domain.* | *High risk of bias for one or more key domains.* |  |

| **RISK OF BIAS ASSESSMENT CHECKLIST  FOR STUDIES INCLUDED IN SYSTEMATIC REVIEWS OF DRUG HARMS** | | | | |
| --- | --- | --- | --- | --- |
|  |  |  |  |  |
|  | **Study ID - Author** | Gay et al (2021), FORTE/UNITO-MM-01, NCT02203643 |  |  |
|  |  |  |  |  |
| **A. STUDY DESIGN AND OBJECTIVES** | | | | |
| **A1. Are study objectives clearly specified and appropriate?** | | | | **Yes** |
|  | *Yes:* | *No:* |  |  |
|  | *Study objectives clearly specified and appropriate.* | *Study objectives are not clearly specified or not appropriate.* |  |  |
| **A2. Is study design clearly specified and appropriate?** | | | | **Yes** |
|  | *Yes:* | *No:* |  |  |
|  | *Study design clearly specified and appropriate.* | *Study design not clearly specified or not appropriate.* |  |  |
| **A3. Is the study design free of run-in/lead-in period before inclusion/randomization of participants?** | | | | **Yes** |
|  | *Yes:* | *Unclear:* | *No:* |  |
|  | *No run-in/lead-in period.* | *Not clear information.* | *Presence of a run-in/lead-in period.* |  |
| **A4. Cross-over designs: Is the study designed to adequately address carry-over effect?** | | | | **yes** |
| *N/A* | *Yes:* | *Unclear:* | *No:* |  |
|  | *Carry-over effect absent or adequately addressed (randomized order and sufficiently long wash-out period.* | *Not clear information.* | *Carry-over effect not adequately addressed and susceptible to bias the results.* |  |
| **A. RISK OF BIAS ASSESSMENT FOR STUDY DESIGN AND OBJECTIVES** | | | | **Low** |
|  | *Low:* | *Unclear:* | *High:* |  |
|  | *Plausible bias unlikely to seriously alter the results.* | *Plausible bias that raises some doubts about the results or when information on which to base risk of bias judgments is missing or poorly reported.* | *Plausible bias that seriously weakens confidence in the results.* |  |
| **Comments: Low risk of bias according to data provided by sources** | | | | |
|  |  |  |  |  |
| **B. BIAS IN SELECTION OF SUBJECTS AND CONSTITUTION OF STUDY GROUPS** | | | | |
| **B1. Was the method used to generate the allocation sequence adequate as to produce comparable groups?** | | | | **Yes** |
|  | *Yes:* | *Unclear:* | *No:* |  |
|  | *Allocation methods are adequate to produce comparable groups.* | *Allocation methods are not clearly reported.* | *Allocation methods are not adequate (e.g. assignment to treatment by birth date, week day, etc.), groups are not comparable.* |  |
| **B2. Was the method used to conceal the allocation sequence adequate as to produce comparable groups?** | | | | **Yes** |
|  | *Yes:* | *Unclear:* | *No:* |  |
|  | *Concealment is adequate.* | *Concealment methods are not clearly reported and groups may not be comparable.* | *Concealment methods are not adequate, groups are not comparable.* |  |
| **B3. Are all the subjects recruited from the same source population?** | | | | **Yes** |
|  | *Yes:* | *Unclear:* | *No:* |  |
|  | *All the subjects recruited from the same source population.* | *Unclear if all the subjects recruited from the same source population.* | *All the subjects are not recruited from the same source population.* |  |
| **B4. Were inclusion and exclusion criteria implemented uniformly across study groups?** | | | | **Yes** |
|  | *Yes:* | *Unclear:* | *No:* |  |
|  | *Selection criteria uniformly implemented.* | *Unclear if selection criteria are uniformly implemented.* | *Selection criteria not uniformly implemented.* |  |
| **B8. Are baseline characteristics and prognostic factors comparable between different groups?** | | | | **Yes** |
|  | *Yes:* | *Unclear:* | *No:* |  |
|  | *RCT: Groups are comparable at baseline.* | *No description of baseline characteristics or only significance tests.* | *The groups are unbalanced at baseline.* |  |
|  | *Cohort studies: Groups are comparable at baseline or matched for the main prognostic factors.* |  |  |  |
| **B. RISK OF BIAS ASSESSMENT FOR SELECTION OF PARTICIPANTS AND CONSTITUTION OF STUDY GROUPS** | | | | **Low** |
|  | *Low:* | *Unclear:* | *High:* |  |
|  | *Plausible bias unlikely to seriously alter the results.* | *Plausible bias that raises some doubts about the results or when information on which to base risk of bias judgments is missing or poorly reported.* | *Plausible bias that seriously weakens confidence in the results.* |  |
| **Comments: low risk of bias according to data provided by sources** | | | | |
|  |  |  |  |  |
| **C. BIAS DUE TO WITHDRAWALS OR LOSS OF FOLLOW-UP (ATTRITION)** | | | | |
| **C1. Are the number of participants clearly reported throughout the study?** | | | | **Yes** |
|  | *Yes:* | *No:* |  |  |
|  | *Numbers of participants throughout the study are reported. Complete flow chart.* | *Numbers of patients at every stage is not clearly reported. Confusing information is reported regarding the number of participants. No or incomplete flow chart.* |  |  |
| **C2. Is the number of drop-outs/withdrawals due to harmful outcome clearly stated for each treatment arm?** | | | | **yes** |
|  | *Yes:* | *No:* |  |  |
|  | *The number of drop-outs due to harmful outcome is specified.* | *The number of drop-outs due to harmful outcome is not specified, unclear or combined.* |  |  |
| **C3. Does the study adequately address biased loss to follow-up?** | | | | **Yes** |
|  | *Yes:* | *Unclear:* | *No:* |  |
|  | *Complete follow-up or drop-outs unlikely to introduce bias or adequately controlled.* | *Drop-outs/withdraws due to harmful outcome are not clearly reported.* | *Loss to follow-up affects the safety outcome and is not adequately controlled.* |  |
| **C4. Are the results based on an intention-to-treat analysis?** | | | | **yes** |
|  | *Yes:* | *Unclear:* | *No:* |  |
|  | *Results are based on a strict intention-to-treat analysis.* | *Not clear if an intention-to-treat analysis is performed. No strict intention-to-treat analysis.* | *Results are not based on intention-to-treat analysis (not done or not possible).* |  |
| **C. RISK OF BIAS DUE TO WITHDRAWALS OR LOSS OF FOLLOW-UP (ATTRITION)** | | | | **Yes** |
|  | *Low:* | *Unclear:* | *High:* |  |
|  | *Plausible bias unlikely to seriously alter the results.* | *Plausible bias that raises some doubts about the results or when information on which to base risk of bias judgments is missing or poorly reported.* | *Plausible bias that seriously weakens confidence in the results.* |  |
| **Comments: low risk of bias according to data provided by study sources** | | | | |
|  |  |  |  |  |
| **D. INFORMATION BIAS REGARDING THE HARMFULL OUTCOME** | | | | |
| **D1. Is the definition of the harmful outcome clearly stated?** | | | | **No** |
|  | *Yes:* | *No:* |  |  |
|  | *RCT: clear / standardized definition of the harmful outcome (e.g. diagnostic codes, clinical and laboratory data). Cohort studies: clear definition of the outcome. Case-control studies: clear definition of cases.* | *Definition of the harmful outcome not reported or that leads to confusion. Terms not well-constructed, wrong definition.* |  |  |
| **D2. If applicable, is the severity of the harmful outcome clearly stated?** | | | | **No** |
|  | *N/A:* | *Yes:* | *No:* |  |
|  | *Self evident severity (e.g. death).* | *Detailed degree of severity or reference to a known scale of severity or a new scale developed for the study.* | *Unclear degrees of severity or without clear boundaries between them.* |  |
| **D3. Was the blinding methods of participants regarding the intervention appropriate considering the nature of the harmful outcome?** | | | | **Unclear** |
|  | *Yes:* | *Unclear:* | *No:* |  |
|  | *Blinding ensured (and unlikely broken) or outcome not likely to be influenced by lack of blinding.* | *There is no sufficient information regarding the process of blinding or the outcome assessment.* | *No blinding (or incomplete blinding or risk of broken blinding) and outcome likely to be influenced by lack of blinding.* |  |
| **D4. Was the blinding methods of harmful outcome assessment appropriate considering the nature of the harmful outcome?** | | | | **Unclear** |
|  | *Yes:* | *Unclear:* | *No:* |  |
|  | *Blinding ensured (and unlikely broken) or outcome assessment not likely to be influenced by lack of blinding.* | *There is no sufficient information regarding the process of blinding of outcome assessment.* | *No blinding (or incomplete blinding or risk of broken blinding) and outcome likely to be influenced by lack of blinding.* |  |
| **D5. Was the duration of follow-up adequate to assess the harmful outcome?** | | | | **Unclear** |
|  | *Yes:* | *Unclear:* | *No:* |  |
|  | *Sufficient duration of follow-up to assess the outcome.* | *It is unclear whether the duration of follow-up is adequate.* | *Too short duration of follow-up.* |  |
| **D6. Was the methods for ascertaining the harmful outcome adequately constructed and equal for all participants?** | | | | **Unclear** |
|  | *Yes:* | *Unclear:* | *No:* |  |
|  | *Adequate or validated methods of outcome measurement for all participants. Clinical reactions medically confirmed by a physician. Minimized risk of misclassification or differential assessment, reporting or detection.  RCT : Active harmful outcome surveillance (prospective/retrospective case-record review, questionnaires, patient’s diary/checklist…) .* | *There is no or not sufficient information to clearly determine how information on harmful outcome is collected or the process of minimizing misclassification.* | *Substantial risk of misclassification of outcome or differential assessment, reporting or detection. Clinical reactions not medically confirmed. RCT : Passive harmful outcome surveillance (patient’s volunteer reporting).* |  |
| **D7. Are the number of harmful outcome and the number of patients with a harmful outcome reported in both treatment arms?** | | | | **No** |
|  | *Yes:* | *Unclear:* | *No:* |  |
|  | *Numbers are reported. It is possible to calculate the rates of harmful outcome.* | *Confusion between the number of harmful outcomes or the number of patients with a harmful outcome, or general statements such as “5% of patients developed a harmful outcome”.* | *Neither the number of harmful outcomes nor the number of patients with a harmful outcome is reported. Or numbers are combining both treatment arms.* |  |
| **D8. Is the time frequency of harmful outcome assessment during the follow-up period appropriate?** | | | | **Unclear** |
|  | *Yes:* | *Unclear:* | *No:* |  |
|  | *For all study groups, the time frequency at which the harmful outcome is assessed is appropriate.* | *General statements such as “patients were routinely assessed for harmful outcomes”.* | *There is no regular collection of data on harmful outcomes during the study.* |  |
| **D9. Was the time between the exposure to a drug and the onset of the harmful outcome reported?** | | | | **No** |
|  | *Yes:* | *Unclear:* | *No:* |  |
|  | *The time between the drug exposure to the onset of harmful outcome is specified.* | *The authors do not report a clear time frame between drug exposure and harmful outcome.* | *The authors do not report the time between the drug exposure to the onset of harmful outcome.* |  |
| **D10. Was the process of determining that the harmful outcome is linked to the drug appropriate? Was the process blinded to the assigned treatment?** | | | | **No** |
|  | *Yes:* | *Unclear:* | *No:* |  |
|  | *Methods for causality assessment are appropriate and, if applicable, made by investigators blinded to the intervention.* | *Unclear how the causality attribution is made. It is not clear who make the assessment or whether it is blinded to the assigned treatment.* | *Causality assessment is made by investigators not blinded to the intervention, or by participants or sponsors, or unblinding of treatment assignment precedes the decision to withdraw.* |  |
| **D. RISK OF BIAS ASSESSMENT FOR INFORMATION BIAS REGARDING THE HARMFULL OUTCOME** | | | | **Unclear** |
|  | *Low:* | *Unclear:* | *High:* |  |
|  | *Plausible bias unlikely to seriously alter the results.* | *Plausible bias that raises some doubts about the results or when information on which to base risk of bias judgments is missing or poorly reported.* | *Plausible bias that seriously weakens confidence in the results.* |  |
| **Comments: Unclear risk of bias** | | | | |
|  |  |  |  |  |
| **E. OTHER INFORMATION BIAS** | | | | |
| **E1. Is blinding of care givers during follow-up adequately performed in order to avoid differential care between study groups (performance bias)?** | | | | **Unclear** |
|  | *Yes:* | *Unclear:* | *No:* |  |
|  | *There is no risk of differential care or it is adequately addressed.* | *Unclear risk of bias due to differential care.* | *The bias due to differential care is not controlled.* |  |
| **E5. Does the study appear free of other information bias ?** | | | | **Unclear** |
|  | *Yes:* | *Unclear:* | *No:* |  |
|  | *The study appears to be free of other information bias.* | *Unclear presence of other information bias.* | *Additional source of other information bias.* |  |
| **E. RISK OF BIAS ASSESSMENT FOR OTHER INFORMATION BIAS** | | | | **Unclear** |
|  | *Low:* | *Unclear:* | *High:* |  |
|  | *Plausible bias unlikely to seriously alter the results.* | *Plausible bias that raises some doubts about the results or when information on which to base risk of bias judgments is missing or poorly reported.* | *Plausible bias that seriously weakens confidence in the results.* |  |
| **Comments: unclear of bias -> open label** | | | | |
|  |  |  |  |  |
| **F. STATISTICAL METHODS TO CONTROL CONFOUNDING** | | | | |
| **F5. Does the study adequately address residual or unmeasured confounding?** | | | | **Yes** |
|  | *Yes:* | *Unclear:* | *No:* |  |
|  | *The study adequately addresses residual or unmeasured confounding.* | *Unclear presence of residual or unmeasured confounding.* | *Residual or unmeasured confounding is likely to be important.* |  |
| **F. RISK OF BIAS ASSESSMENT FOR STATISTICAL METHODS TO CONTROL CONFOUNDING:** | | | | **Low** |
|  | *Low:* | *Unclear:* | *High:* |  |
|  | *Plausible bias unlikely to seriously alter the results.* | *Plausible bias that raises some doubts about the results or when information on which to base risk of bias judgments is missing or poorly reported.* | *Plausible bias that seriously weakens confidence in the results.* |  |
| **Comments: Low risk of bias->Statistical Analysis section is well informative ( stratification according to International Staging System stage (I vs II/III), age (<60 years vs 60–65 years), induction–consolidation treatment)** | | | | |
|  |  |  |  |  |
| **G. STATISTICAL METHODS EXCLUDING METHODS TO CONTROL CONFOUNDING** | | | | |
| **G1. Are the statistical methods used to analyze the harmful outcome appropriate?** | | | | **Unclear** |
|  | *Yes:* | *Unclear:* | *No:* |  |
|  | *Statistical techniques are appropriate to the data. If the distribution of the data (normal or not) is not described, it must be assumed that the estimates used were appropriate.* | *There is no description of the statistical techniques used, or the description is vague and not understandable.* | *The statistical techniques used are not appropriate.* |  |
| **G2. Is a survival analysis performed when there are individual differences in length of follow-up?** | | | | **Unclear** |
|  | *Yes:* | *Unclear:* | *No:* |  |
|  | *Follow-up is the same for all study patients, if not survival analysis is performed.* | *Unclear whether there are different lengths of follow-up or whether they are taken into account.* | *Differences of follow up were ignored.* |  |
| **G3. If applicable, is composite outcome of harms adequately constructed?** | | | | **N/A** |
| *N/A* | *Yes:* | *Unclear:* | *No:* |  |
|  | *Composite outcome appropriate.* | *Unclear whether composite outcome is appropriate.* | *Construction of composite not described or not appropriate.* |  |
| **G6.** **Are the results consistent in primary and secondary analyses? Are confounding effects consistent with known associations?** | | | | **Yes** |
|  | *Yes:* | *Unclear:* | *No:* |  |
|  | *Consistency of primary, secondary analyses and consistency of confounding effects with known associations.* | *Not sufficient information to determine consistency.* | *No consistency of primary, secondary analyses or no consistency of confounding effects with known associations.* |  |
| **G. RISK OF BIAS ASSESSMENT FOR STATISTICAL METHODS EXCLUDING METHODS TO CONTROL CONFOUNDING:** | | | | **Unclear** |
|  | *Low:* | *Unclear:* | *High:* |  |
|  | *Plausible bias unlikely to seriously alter the results.* | *Plausible bias that raises some doubts about the results or when information on which to base risk of bias judgments is missing or poorly reported.* | *Plausible bias that seriously weakens confidence in the results.* |  |
| **Comments: Unclear risk of bias** | | | | |
|  | | | |  |
| **H. CONFLICT OF INTEREST** | | | | |
| **H1. Were the conflict of interest or sources of funding clearly acknowledged?** | | | | **Yes** |
|  | *Yes:* | *No:* |  |  |
|  | *Potential sources of support are acknowledged.* | *No sources of funding reported or not sufficient information.* |  |  |
| **H2. Does the study appear free of conflicts of interest susceptible to have influenced design, analysis or reporting (selective reporting of outcome or analysis)?** | | | | **Yes** |
|  | *Yes:* | *Unclear:* | *No:* |  |
|  | *No conflicts of interest or not susceptible to have influenced design, analysis or reporting.* | *It is unclear if there are conflicts of interest or if they are susceptible to have influenced design, analysis or reporting.* | *Conflicts of interest susceptible to have influenced design, analysis or reporting.* |  |
| **H. RISK OF BIAS ASSESSMENT FOR CONFLICT OF INTEREST** | | | | **Low** |
|  | *Low:* | *Unclear:* | *High:* |  |
|  | *Plausible bias unlikely to seriously alter the results.* | *Plausible bias that raises some doubts about the results or when information on which to base risk of bias judgments is missing or poorly reported.* | *Plausible bias that seriously weakens confidence in the results.* |  |
|  |  |  |  |  |
| **SUMMARY RISK-OF-BIAS ASSESSMENT FOR THE STUDY** | | | | |
| **RISK OF BIAS ASSESSMENT FOR THE STUDY** | | | | **Unclear** |
|  | *Low:* | *Unclear:* | *High:* |  |
|  | *Low risk of bias for all key domains.* | *Unclear risk of bias for one or more key domain.* | *High risk of bias for one or more key domains.* |  |

| **RISK OF BIAS ASSESSMENT CHECKLIST  FOR STUDIES INCLUDED IN SYSTEMATIC REVIEWS OF DRUG HARMS** | | | | |
| --- | --- | --- | --- | --- |
|  | **Study ID - Author** | Jones et al (2023), MYELOMA XI, NCT01554852 |  |  |
|  |  |  |  |  |
| **A. STUDY DESIGN AND OBJECTIVES** | | | | |
| **A1. Are study objectives clearly specified and appropriate?** | | | | **Yes** |
|  | *Yes:* | *No:* |  |  |
|  | *Study objectives clearly specified and appropriate.* | *Study objectives are not clearly specified or not appropriate.* |  |  |
| **A2. Is study design clearly specified and appropriate?** | | | | **Yes** |
|  | *Yes:* | *No:* |  |  |
|  | *Study design clearly specified and appropriate.* | *Study design not clearly specified or not appropriate.* |  |  |
| **A3. Is the study design free of run-in/lead-in period before inclusion/randomization of participants?** | | | | **Yes** |
|  | *Yes:* | *Unclear:* | *No:* |  |
|  | *No run-in/lead-in period.* | *Not clear information.* | *Presence of a run-in/lead-in period.* |  |
| **A4. Cross-over designs: Is the study designed to adequately address carry-over effect?** | | | | **Yes** |
| *N/A* | *Yes:* | *Unclear:* | *No:* |  |
|  | *Carry-over effect absent or adequately addressed (randomized order and sufficiently long wash-out period.* | *Not clear information.* | *Carry-over effect not adequately addressed and susceptible to bias the results.* |  |
| **A. RISK OF BIAS ASSESSMENT FOR STUDY DESIGN AND OBJECTIVES** | | | | **Low** |
|  | *Low:* | *Unclear:* | *High:* |  |
|  | *Plausible bias unlikely to seriously alter the results.* | *Plausible bias that raises some doubts about the results or when information on which to base risk of bias judgments is missing or poorly reported.* | *Plausible bias that seriously weakens confidence in the results.* |  |
| **Comments: Low risk of bias according to data provided by study protocol, SAP and results** | | | | |
|  |  |  |  |  |
| **B. BIAS IN SELECTION OF SUBJECTS AND CONSTITUTION OF STUDY GROUPS** | | | | |
| **B1. Was the method used to generate the allocation sequence adequate as to produce comparable groups?** | | | | **Yes** |
|  | *Yes:* | *Unclear:* | *No:* |  |
|  | *Allocation methods are adequate to produce comparable groups.* | *Allocation methods are not clearly reported.* | *Allocation methods are not adequate (e.g. assignment to treatment by birth date, week day, etc.), groups are not comparable.* |  |
| **B2. Was the method used to conceal the allocation sequence adequate as to produce comparable groups?** | | | | **Yes** |
|  | *Yes:* | *Unclear:* | *No:* |  |
|  | *Concealment is adequate.* | *Concealment methods are not clearly reported and groups may not be comparable.* | *Concealment methods are not adequate, groups are not comparable.* |  |
| **B3. Are all the subjects recruited from the same source population?** | | | | **Yes** |
|  | *Yes:* | *Unclear:* | *No:* |  |
|  | *All the subjects recruited from the same source population.* | *Unclear if all the subjects recruited from the same source population.* | *All the subjects are not recruited from the same source population.* |  |
| **B4. Were inclusion and exclusion criteria implemented uniformly across study groups?** | | | | **Yes** |
|  | *Yes:* | *Unclear:* | *No:* |  |
|  | *Selection criteria uniformly implemented.* | *Unclear if selection criteria are uniformly implemented.* | *Selection criteria not uniformly implemented.* |  |
| **B8. Are baseline characteristics and prognostic factors comparable between different groups?** | | | | **Yes** |
|  | *Yes:* | *Unclear:* | *No:* |  |
|  | *RCT: Groups are comparable at baseline.* | *No description of baseline characteristics or only significance tests.* | *The groups are unbalanced at baseline.* |  |
|  | *Cohort studies: Groups are comparable at baseline or matched for the main prognostic factors.* |  |  |  |
| **B. RISK OF BIAS ASSESSMENT FOR SELECTION OF PARTICIPANTS AND CONSTITUTION OF STUDY GROUPS** | | | | **Low** |
|  | *Low:* | *Unclear:* | *High:* |  |
|  | *Plausible bias unlikely to seriously alter the results.* | *Plausible bias that raises some doubts about the results or when information on which to base risk of bias judgments is missing or poorly reported.* | *Plausible bias that seriously weakens confidence in the results.* |  |
| **Comments: Low risk of bias according to data provided by study protocol, SAP and results** | | | | |
|  |  |  |  |  |
| **C. BIAS DUE TO WITHDRAWALS OR LOSS OF FOLLOW-UP (ATTRITION)** | | | | |
| **C1. Are the number of participants clearly reported throughout the study?** | | | | **Yes** |
|  | *Yes:* | *No:* |  |  |
|  | *Numbers of participants throughout the study are reported. Complete flow chart.* | *Numbers of patients at every stage is not clearly reported. Confusing information is reported regarding the number of participants. No or incomplete flow chart.* |  |  |
| **C2. Is the number of drop-outs/withdrawals due to harmful outcome clearly stated for each treatment arm?** | | | | **yes** |
|  | *Yes:* | *No:* |  |  |
|  | *The number of drop-outs due to harmful outcome is specified.* | *The number of drop-outs due to harmful outcome is not specified, unclear or combined.* |  |  |
| **C3. Does the study adequately address biased loss to follow-up?** | | | | **yes** |
|  | *Yes:* | *Unclear:* | *No:* |  |
|  | *Complete follow-up or drop-outs unlikely to introduce bias or adequately controlled.* | *Drop-outs/withdraws due to harmful outcome are not clearly reported.* | *Loss to follow-up affects the safety outcome and is not adequately controlled.* |  |
| **C4. Are the results based on an intention-to-treat analysis?** | | | | **yes** |
|  | *Yes:* | *Unclear:* | *No:* |  |
|  | *Results are based on a strict intention-to-treat analysis.* | *Not clear if an intention-to-treat analysis is performed. No strict intention-to-treat analysis.* | *Results are not based on intention-to-treat analysis (not done or not possible).* |  |
| **C. RISK OF BIAS DUE TO WITHDRAWALS OR LOSS OF FOLLOW-UP (ATTRITION)** | | | | **Low** |
|  | *Low:* | *Unclear:* | *High:* |  |
|  | *Plausible bias unlikely to seriously alter the results.* | *Plausible bias that raises some doubts about the results or when information on which to base risk of bias judgments is missing or poorly reported.* | *Plausible bias that seriously weakens confidence in the results.* |  |
| **Comments: Low risk of bias according to data provided by study protocol, SAP and results** | | | | |
|  |  |  |  |  |
| **D. INFORMATION BIAS REGARDING THE HARMFULL OUTCOME** | | | | |
| **D1. Is the definition of the harmful outcome clearly stated?** | | | | **Yes** |
|  | *Yes:* | *No:* |  |  |
|  | *RCT: clear / standardized definition of the harmful outcome (e.g. diagnostic codes, clinical and laboratory data). Cohort studies: clear definition of the outcome. Case-control studies: clear definition of cases.* | *Definition of the harmful outcome not reported or that leads to confusion. Terms not well-constructed, wrong definition.* |  |  |
| **D2. If applicable, is the severity of the harmful outcome clearly stated?** | | | | **Yes** |
|  | *N/A:* | *Yes:* | *No:* |  |
|  | *Self evident severity (e.g. death).* | *Detailed degree of severity or reference to a known scale of severity or a new scale developed for the study.* | *Unclear degrees of severity or without clear boundaries between them.* |  |
| **D3. Was the blinding methods of participants regarding the intervention appropriate considering the nature of the harmful outcome?** | | | | **Unclear** |
|  | *Yes:* | *Unclear:* | *No:* |  |
|  | *Blinding ensured (and unlikely broken) or outcome not likely to be influenced by lack of blinding.* | *There is no sufficient information regarding the process of blinding or the outcome assessment.* | *No blinding (or incomplete blinding or risk of broken blinding) and outcome likely to be influenced by lack of blinding.* |  |
| **D4. Was the blinding methods of harmful outcome assessment appropriate considering the nature of the harmful outcome?** | | | | **Unclear** |
|  | *Yes:* | *Unclear:* | *No:* |  |
|  | *Blinding ensured (and unlikely broken) or outcome assessment not likely to be influenced by lack of blinding.* | *There is no sufficient information regarding the process of blinding of outcome assessment.* | *No blinding (or incomplete blinding or risk of broken blinding) and outcome likely to be influenced by lack of blinding.* |  |
| **D5. Was the duration of follow-up adequate to assess the harmful outcome?** | | | | **Unclear** |
|  | *Yes:* | *Unclear:* | *No:* |  |
|  | *Sufficient duration of follow-up to assess the outcome.* | *It is unclear whether the duration of follow-up is adequate.* | *Too short duration of follow-up.* |  |
| **D6. Was the methods for ascertaining the harmful outcome adequately constructed and equal for all participants?** | | | | **Unclear** |
|  | *Yes:* | *Unclear:* | *No:* |  |
|  | *Adequate or validated methods of outcome measurement for all participants. Clinical reactions medically confirmed by a physician. Minimized risk of misclassification or differential assessment, reporting or detection.  RCT : Active harmful outcome surveillance (prospective/retrospective case-record review, questionnaires, patient’s diary/checklist…) .* | *There is no or not sufficient information to clearly determine how information on harmful outcome is collected or the process of minimizing misclassification.* | *Substantial risk of misclassification of outcome or differential assessment, reporting or detection. Clinical reactions not medically confirmed. RCT : Passive harmful outcome surveillance (patient’s volunteer reporting).* |  |
| **D7. Are the number of harmful outcome and the number of patients with a harmful outcome reported in both treatment arms?** | | | | **Yes** |
|  | *Yes:* | *Unclear:* | *No:* |  |
|  | *Numbers are reported. It is possible to calculate the rates of harmful outcome.* | *Confusion between the number of harmful outcomes or the number of patients with a harmful outcome, or general statements such as “5% of patients developed a harmful outcome”.* | *Neither the number of harmful outcomes nor the number of patients with a harmful outcome is reported. Or numbers are combining both treatment arms.* |  |
| **D8. Is the time frequency of harmful outcome assessment during the follow-up period appropriate?** | | | | **Unclear** |
|  | *Yes:* | *Unclear:* | *No:* |  |
|  | *For all study groups, the time frequency at which the harmful outcome is assessed is appropriate.* | *General statements such as “patients were routinely assessed for harmful outcomes”.* | *There is no regular collection of data on harmful outcomes during the study.* |  |
| **D9. Was the time between the exposure to a drug and the onset of the harmful outcome reported?** | | | | **Unclear** |
|  | *Yes:* | *Unclear:* | *No:* |  |
|  | *The time between the drug exposure to the onset of harmful outcome is specified.* | *The authors do not report a clear time frame between drug exposure and harmful outcome.* | *The authors do not report the time between the drug exposure to the onset of harmful outcome.* |  |
| **D10. Was the process of determining that the harmful outcome is linked to the drug appropriate? Was the process blinded to the assigned treatment?** | | | | **Unclear** |
|  | *Yes:* | *Unclear:* | *No:* |  |
|  | *Methods for causality assessment are appropriate and, if applicable, made by investigators blinded to the intervention.* | *Unclear how the causality attribution is made. It is not clear who make the assessment or whether it is blinded to the assigned treatment.* | *Causality assessment is made by investigators not blinded to the intervention, or by participants or sponsors, or unblinding of treatment assignment precedes the decision to withdraw.* |  |
| **D. RISK OF BIAS ASSESSMENT FOR INFORMATION BIAS REGARDING THE HARMFULL OUTCOME** | | | | **Unclear** |
|  | *Low:* | *Unclear:* | *High:* |  |
|  | *Plausible bias unlikely to seriously alter the results.* | *Plausible bias that raises some doubts about the results or when information on which to base risk of bias judgments is missing or poorly reported.* | *Plausible bias that seriously weakens confidence in the results.* |  |
| **Comments: unclear risk of bias according to data provided by study protocol, SAP and results** | | | | |
|  |  |  |  |  |
| **E. OTHER INFORMATION BIAS** | | | | |
| **E1. Is blinding of care givers during follow-up adequately performed in order to avoid differential care between study groups (performance bias)?** | | | | **Unclear** |
|  | *Yes:* | *Unclear:* | *No:* |  |
|  | *There is no risk of differential care or it is adequately addressed.* | *Unclear risk of bias due to differential care.* | *The bias due to differential care is not controlled.* |  |
| **E5. Does the study appear free of other information bias ?** | | | | **Unclear** |
|  | *Yes:* | *Unclear:* | *No:* |  |
|  | *The study appears to be free of other information bias.* | *Unclear presence of other information bias.* | *Additional source of other information bias.* |  |
| **E. RISK OF BIAS ASSESSMENT FOR OTHER INFORMATION BIAS** | | | | **Unclear** |
|  | *Low:* | *Unclear:* | *High:* |  |
|  | *Plausible bias unlikely to seriously alter the results.* | *Plausible bias that raises some doubts about the results or when information on which to base risk of bias judgments is missing or poorly reported.* | *Plausible bias that seriously weakens confidence in the results.* |  |
| **Comments: unclear risk of bias -> open label** | | | | |
|  |  |  |  |  |
| **F. STATISTICAL METHODS TO CONTROL CONFOUNDING** | | | | |
| **F5. Does the study adequately address residual or unmeasured confounding?** | | | | **Yes** |
|  | *Yes:* | *Unclear:* | *No:* |  |
|  | *The study adequately addresses residual or unmeasured confounding.* | *Unclear presence of residual or unmeasured confounding.* | *Residual or unmeasured confounding is likely to be important.* |  |
| **F. RISK OF BIAS ASSESSMENT FOR STATISTICAL METHODS TO CONTROL CONFOUNDING:** | | | | **Low** |
|  | *Low:* | *Unclear:* | *High:* |  |
|  | *Plausible bias unlikely to seriously alter the results.* | *Plausible bias that raises some doubts about the results or when information on which to base risk of bias judgments is missing or poorly reported.* | *Plausible bias that seriously weakens confidence in the results.* |  |
| **Comments: Low risk of bias->Statistical Analysis/results sections were informative (stratified by allocated induction and intensification treatment, and centre).** | | | | |
|  |  |  |  |  |
| **G. STATISTICAL METHODS EXCLUDING METHODS TO CONTROL CONFOUNDING** | | | | |
| **G1. Are the statistical methods used to analyze the harmful outcome appropriate?** | | | | **Unclear** |
|  | *Yes:* | *Unclear:* | *No:* |  |
|  | *Statistical techniques are appropriate to the data. If the distribution of the data (normal or not) is not described, it must be assumed that the estimates used were appropriate.* | *There is no description of the statistical techniques used, or the description is vague and not understandable.* | *The statistical techniques used are not appropriate.* |  |
| **G2. Is a survival analysis performed when there are individual differences in length of follow-up?** | | | | **Unclear** |
|  | *Yes:* | *Unclear:* | *No:* |  |
|  | *Follow-up is the same for all study patients, if not survival analysis is performed.* | *Unclear whether there are different lengths of follow-up or whether they are taken into account.* | *Differences of follow up were ignored.* |  |
| **G3. If applicable, is composite outcome of harms adequately constructed?** | | | | **N/A** |
| *N/A* | *Yes:* | *Unclear:* | *No:* |  |
|  | *Composite outcome appropriate.* | *Unclear whether composite outcome is appropriate.* | *Construction of composite not described or not appropriate.* |  |
| **G6.** **Are the results consistent in primary and secondary analyses? Are confounding effects consistent with known associations?** | | | | **Yes** |
|  | *Yes:* | *Unclear:* | *No:* |  |
|  | *Consistency of primary, secondary analyses and consistency of confounding effects with known associations.* | *Not sufficient information to determine consistency.* | *No consistency of primary, secondary analyses or no consistency of confounding effects with known associations.* |  |
| **G. RISK OF BIAS ASSESSMENT FOR STATISTICAL METHODS EXCLUDING METHODS TO CONTROL CONFOUNDING:** | | | | **Unclear** |
|  | *Low:* | *Unclear:* | *High:* |  |
|  | *Plausible bias unlikely to seriously alter the results.* | *Plausible bias that raises some doubts about the results or when information on which to base risk of bias judgments is missing or poorly reported.* | *Plausible bias that seriously weakens confidence in the results.* |  |
| **Comments: Unclear risk of bias** | | | | |
|  | | | |  |
| **H. CONFLICT OF INTEREST** | | | | |
| **H1. Were the conflict of interest or sources of funding clearly acknowledged?** | | | | **Yes** |
|  | *Yes:* | *No:* |  |  |
|  | *Potential sources of support are acknowledged.* | *No sources of funding reported or not sufficient information.* |  |  |
| **H2. Does the study appear free of conflicts of interest susceptible to have influenced design, analysis or reporting (selective reporting of outcome or analysis)?** | | | | **yes** |
|  | *Yes:* | *Unclear:* | *No:* |  |
|  | *No conflicts of interest or not susceptible to have influenced design, analysis or reporting.* | *It is unclear if there are conflicts of interest or if they are susceptible to have influenced design, analysis or reporting.* | *Conflicts of interest susceptible to have influenced design, analysis or reporting.* |  |
| **H. RISK OF BIAS ASSESSMENT FOR CONFLICT OF INTEREST** | | | | **low** |
|  | *Low:* | *Unclear:* | *High:* |  |
|  | *Plausible bias unlikely to seriously alter the results.* | *Plausible bias that raises some doubts about the results or when information on which to base risk of bias judgments is missing or poorly reported.* | *Plausible bias that seriously weakens confidence in the results.* |  |
|  |  |  |  |  |
| **SUMMARY RISK-OF-BIAS ASSESSMENT FOR THE STUDY** | | | | |
| **RISK OF BIAS ASSESSMENT FOR THE STUDY** | | | | **Unclear** |
|  | *Low:* | *Unclear:* | *High:* |  |
|  | *Low risk of bias for all key domains.* | *Unclear risk of bias for one or more key domain.* | *High risk of bias for one or more key domains.* |  |

| **RISK OF BIAS ASSESSMENT CHECKLIST  FOR STUDIES INCLUDED IN SYSTEMATIC REVIEWS OF DRUG HARMS** | | | | |
| --- | --- | --- | --- | --- |
|  |  |  |  |  |
|  | **Study ID - Author** | Jacobus et al (2016), ECOG-E1A05, NCT00522392 |  |  |
|  |  |  |  |  |
| **A. STUDY DESIGN AND OBJECTIVES** | | | | |
| **A1. Are study objectives clearly specified and appropriate?** | | | | **Yes** |
|  | *Yes:* | *No:* |  |  |
|  | *Study objectives clearly specified and appropriate.* | *Study objectives are not clearly specified or not appropriate.* |  |  |
| **A2. Is study design clearly specified and appropriate?** | | | | **Yes** |
|  | *Yes:* | *No:* |  |  |
|  | *Study design clearly specified and appropriate.* | *Study design not clearly specified or not appropriate.* |  |  |
| **A3. Is the study design free of run-in/lead-in period before inclusion/randomization of participants?** | | | | **Yes** |
|  | *Yes:* | *Unclear:* | *No:* |  |
|  | *No run-in/lead-in period.* | *Not clear information.* | *Presence of a run-in/lead-in period.* |  |
| **A4. Cross-over designs: Is the study designed to adequately address carry-over effect?** | | | | **N/A** |
| *N/A* | *Yes:* | *Unclear:* | *No:* |  |
|  | *Carry-over effect absent or adequately addressed (randomized order and sufficiently long wash-out period.* | *Not clear information.* | *Carry-over effect not adequately addressed and susceptible to bias the results.* |  |
| **A. RISK OF BIAS ASSESSMENT FOR STUDY DESIGN AND OBJECTIVES** | | | | **Low** |
|  | *Low:* | *Unclear:* | *High:* |  |
|  | *Plausible bias unlikely to seriously alter the results.* | *Plausible bias that raises some doubts about the results or when information on which to base risk of bias judgments is missing or poorly reported.* | *Plausible bias that seriously weakens confidence in the results.* |  |
| **Comments: Low risk of bias according to data provided by sources** | | | | |
|  |  |  |  |  |
| **B. BIAS IN SELECTION OF SUBJECTS AND CONSTITUTION OF STUDY GROUPS** | | | | |
| **B1. Was the method used to generate the allocation sequence adequate as to produce comparable groups?** | | | | **Unclear** |
|  | *Yes:* | *Unclear:* | *No:* |  |
|  | *Allocation methods are adequate to produce comparable groups.* | *Allocation methods are not clearly reported.* | *Allocation methods are not adequate (e.g. assignment to treatment by birth date, week day, etc.), groups are not comparable.* |  |
| **B2. Was the method used to conceal the allocation sequence adequate as to produce comparable groups?** | | | | **Yes** |
|  | *Yes:* | *Unclear:* | *No:* |  |
|  | *Concealment is adequate.* | *Concealment methods are not clearly reported and groups may not be comparable.* | *Concealment methods are not adequate, groups are not comparable.* |  |
| **B3. Are all the subjects recruited from the same source population?** | | | | **Yes** |
|  | *Yes:* | *Unclear:* | *No:* |  |
|  | *All the subjects recruited from the same source population.* | *Unclear if all the subjects recruited from the same source population.* | *All the subjects are not recruited from the same source population.* |  |
| **B4. Were inclusion and exclusion criteria implemented uniformly across study groups?** | | | | **Yes** |
|  | *Yes:* | *Unclear:* | *No:* |  |
|  | *Selection criteria uniformly implemented.* | *Unclear if selection criteria are uniformly implemented.* | *Selection criteria not uniformly implemented.* |  |
| **B8. Are baseline characteristics and prognostic factors comparable between different groups?** | | | | **Unclear** |
|  | *Yes:* | *Unclear:* | *No:* |  |
|  | *RCT: Groups are comparable at baseline.* | *No description of baseline characteristics or only significance tests.* | *The groups are unbalanced at baseline.* |  |
|  | *Cohort studies: Groups are comparable at baseline or matched for the main prognostic factors.* |  |  |  |
| **B. RISK OF BIAS ASSESSMENT FOR SELECTION OF PARTICIPANTS AND CONSTITUTION OF STUDY GROUPS** | | | | **Unclear** |
|  | *Low:* | *Unclear:* | *High:* |  |
|  | *Plausible bias unlikely to seriously alter the results.* | *Plausible bias that raises some doubts about the results or when information on which to base risk of bias judgments is missing or poorly reported.* | *Plausible bias that seriously weakens confidence in the results.* |  |
| **Comments: Unclear risk of bias according to data provided by sources** | | | | |
|  |  |  |  |  |
| **C. BIAS DUE TO WITHDRAWALS OR LOSS OF FOLLOW-UP (ATTRITION)** | | | | |
| **C1. Are the number of participants clearly reported throughout the study?** | | | | **Yes** |
|  | *Yes:* | *No:* |  |  |
|  | *Numbers of participants throughout the study are reported. Complete flow chart.* | *Numbers of patients at every stage is not clearly reported. Confusing information is reported regarding the number of participants. No or incomplete flow chart.* |  |  |
| **C2. Is the number of drop-outs/withdrawals due to harmful outcome clearly stated for each treatment arm?** | | | | **Yes** |
|  | *Yes:* | *No:* |  |  |
|  | *The number of drop-outs due to harmful outcome is specified.* | *The number of drop-outs due to harmful outcome is not specified, unclear or combined.* |  |  |
| **C3. Does the study adequately address biased loss to follow-up?** | | | | **Yes** |
|  | *Yes:* | *Unclear:* | *No:* |  |
|  | *Complete follow-up or drop-outs unlikely to introduce bias or adequately controlled.* | *Drop-outs/withdraws due to harmful outcome are not clearly reported.* | *Loss to follow-up affects the safety outcome and is not adequately controlled.* |  |
| **C4. Are the results based on an intention-to-treat analysis?** | | | | **Yes** |
|  | *Yes:* | *Unclear:* | *No:* |  |
|  | *Results are based on a strict intention-to-treat analysis.* | *Not clear if an intention-to-treat analysis is performed. No strict intention-to-treat analysis.* | *Results are not based on intention-to-treat analysis (not done or not possible).* |  |
| **C. RISK OF BIAS DUE TO WITHDRAWALS OR LOSS OF FOLLOW-UP (ATTRITION)** | | | | **Low** |
|  | *Low:* | *Unclear:* | *High:* |  |
|  | *Plausible bias unlikely to seriously alter the results.* | *Plausible bias that raises some doubts about the results or when information on which to base risk of bias judgments is missing or poorly reported.* | *Plausible bias that seriously weakens confidence in the results.* |  |
| **Comments: Low risk of bias according to data provided by sources** | | | | |
|  |  |  |  |  |
| **D. INFORMATION BIAS REGARDING THE HARMFULL OUTCOME** | | | | |
| **D1. Is the definition of the harmful outcome clearly stated?** | | | | **No** |
|  | *Yes:* | *No:* |  |  |
|  | *RCT: clear / standardized definition of the harmful outcome (e.g. diagnostic codes, clinical and laboratory data). Cohort studies: clear definition of the outcome. Case-control studies: clear definition of cases.* | *Definition of the harmful outcome not reported or that leads to confusion. Terms not well-constructed, wrong definition.* |  |  |
| **D2. If applicable, is the severity of the harmful outcome clearly stated?** | | | | **No** |
|  | *N/A:* | *Yes:* | *No:* |  |
|  | *Self evident severity (e.g. death).* | *Detailed degree of severity or reference to a known scale of severity or a new scale developed for the study.* | *Unclear degrees of severity or without clear boundaries between them.* |  |
| **D3. Was the blinding methods of participants regarding the intervention appropriate considering the nature of the harmful outcome?** | | | | **Unclear** |
|  | *Yes:* | *Unclear:* | *No:* |  |
|  | *Blinding ensured (and unlikely broken) or outcome not likely to be influenced by lack of blinding.* | *There is no sufficient information regarding the process of blinding or the outcome assessment.* | *No blinding (or incomplete blinding or risk of broken blinding) and outcome likely to be influenced by lack of blinding.* |  |
| **D4. Was the blinding methods of harmful outcome assessment appropriate considering the nature of the harmful outcome?** | | | | **Unclear** |
|  | *Yes:* | *Unclear:* | *No:* |  |
|  | *Blinding ensured (and unlikely broken) or outcome assessment not likely to be influenced by lack of blinding.* | *There is no sufficient information regarding the process of blinding of outcome assessment.* | *No blinding (or incomplete blinding or risk of broken blinding) and outcome likely to be influenced by lack of blinding.* |  |
| **D5. Was the duration of follow-up adequate to assess the harmful outcome?** | | | | **Unclear** |
|  | *Yes:* | *Unclear:* | *No:* |  |
|  | *Sufficient duration of follow-up to assess the outcome.* | *It is unclear whether the duration of follow-up is adequate.* | *Too short duration of follow-up.* |  |
| **D6. Was the methods for ascertaining the harmful outcome adequately constructed and equal for all participants?** | | | | **Unclear** |
|  | *Yes:* | *Unclear:* | *No:* |  |
|  | *Adequate or validated methods of outcome measurement for all participants. Clinical reactions medically confirmed by a physician. Minimized risk of misclassification or differential assessment, reporting or detection.  RCT : Active harmful outcome surveillance (prospective/retrospective case-record review, questionnaires, patient’s diary/checklist…) .* | *There is no or not sufficient information to clearly determine how information on harmful outcome is collected or the process of minimizing misclassification.* | *Substantial risk of misclassification of outcome or differential assessment, reporting or detection. Clinical reactions not medically confirmed. RCT : Passive harmful outcome surveillance (patient’s volunteer reporting).* |  |
| **D7. Are the number of harmful outcome and the number of patients with a harmful outcome reported in both treatment arms?** | | | | **Yes** |
|  | *Yes:* | *Unclear:* | *No:* |  |
|  | *Numbers are reported. It is possible to calculate the rates of harmful outcome.* | *Confusion between the number of harmful outcomes or the number of patients with a harmful outcome, or general statements such as “5% of patients developed a harmful outcome”.* | *Neither the number of harmful outcomes nor the number of patients with a harmful outcome is reported. Or numbers are combining both treatment arms.* |  |
| **D8. Is the time frequency of harmful outcome assessment during the follow-up period appropriate?** | | | | **Unclear** |
|  | *Yes:* | *Unclear:* | *No:* |  |
|  | *For all study groups, the time frequency at which the harmful outcome is assessed is appropriate.* | *General statements such as “patients were routinely assessed for harmful outcomes”.* | *There is no regular collection of data on harmful outcomes during the study.* |  |
| **D9. Was the time between the exposure to a drug and the onset of the harmful outcome reported?** | | | | **Unclear** |
|  | *Yes:* | *Unclear:* | *No:* |  |
|  | *The time between the drug exposure to the onset of harmful outcome is specified.* | *The authors do not report a clear time frame between drug exposure and harmful outcome.* | *The authors do not report the time between the drug exposure to the onset of harmful outcome.* |  |
| **D10. Was the process of determining that the harmful outcome is linked to the drug appropriate? Was the process blinded to the assigned treatment?** | | | | **Unclear** |
|  | *Yes:* | *Unclear:* | *No:* |  |
|  | *Methods for causality assessment are appropriate and, if applicable, made by investigators blinded to the intervention.* | *Unclear how the causality attribution is made. It is not clear who make the assessment or whether it is blinded to the assigned treatment.* | *Causality assessment is made by investigators not blinded to the intervention, or by participants or sponsors, or unblinding of treatment assignment precedes the decision to withdraw.* |  |
| **D. RISK OF BIAS ASSESSMENT FOR INFORMATION BIAS REGARDING THE HARMFULL OUTCOME** | | | | **Unclear** |
|  | *Low:* | *Unclear:* | *High:* |  |
|  | *Plausible bias unlikely to seriously alter the results.* | *Plausible bias that raises some doubts about the results or when information on which to base risk of bias judgments is missing or poorly reported.* | *Plausible bias that seriously weakens confidence in the results.* |  |
| **Comments: Unclear risk of bias** | | | | |
|  |  |  |  |  |
| **E. OTHER INFORMATION BIAS** | | | | |
| **E1. Is blinding of care givers during follow-up adequately performed in order to avoid differential care between study groups (performance bias)?** | | | | **Unclear** |
|  | *Yes:* | *Unclear:* | *No:* |  |
|  | *There is no risk of differential care or it is adequately addressed.* | *Unclear risk of bias due to differential care.* | *The bias due to differential care is not controlled.* |  |
| **E5. Does the study appear free of other information bias ?** | | | | **Unclear** |
|  | *Yes:* | *Unclear:* | *No:* |  |
|  | *The study appears to be free of other information bias.* | *Unclear presence of other information bias.* | *Additional source of other information bias.* |  |
| **E. RISK OF BIAS ASSESSMENT FOR OTHER INFORMATION BIAS** | | | |  |
|  | *Low:* | *Unclear:* | *High:* |  |
|  | *Plausible bias unlikely to seriously alter the results.* | *Plausible bias that raises some doubts about the results or when information on which to base risk of bias judgments is missing or poorly reported.* | *Plausible bias that seriously weakens confidence in the results.* | **Unclear** |
| **Comments: Unclear of bias -> open label** | | | | |
|  |  |  |  |  |
| **F. STATISTICAL METHODS TO CONTROL CONFOUNDING** | | | | |
| **F5. Does the study adequately address residual or unmeasured confounding?** | | | | **Yes** |
|  | *Yes:* | *Unclear:* | *No:* |  |
|  | *The study adequately addresses residual or unmeasured confounding.* | *Unclear presence of residual or unmeasured confounding.* | *Residual or unmeasured confounding is likely to be important.* |  |
| **F. RISK OF BIAS ASSESSMENT FOR STATISTICAL METHODS TO CONTROL CONFOUNDING:** | | | | **Low** |
|  | *Low:* | *Unclear:* | *High:* |  |
|  | *Plausible bias unlikely to seriously alter the results.* | *Plausible bias that raises some doubts about the results or when information on which to base risk of bias judgments is missing or poorly reported.* | *Plausible bias that seriously weakens confidence in the results.* |  |
| **Comments: Low risk of bias->Statistical Analysis section is well informative (Patients were stratified by prior lenalidomide-dexamethasone use during induction and by complete response (CR) status at registration).** | | | | |
|  |  |  |  |  |
| **G. STATISTICAL METHODS EXCLUDING METHODS TO CONTROL CONFOUNDING** | | | | |
| **G1. Are the statistical methods used to analyze the harmful outcome appropriate?** | | | | **Unclear** |
|  | *Yes:* | *Unclear:* | *No:* |  |
|  | *Statistical techniques are appropriate to the data. If the distribution of the data (normal or not) is not described, it must be assumed that the estimates used were appropriate.* | *There is no description of the statistical techniques used, or the description is vague and not understandable.* | *The statistical techniques used are not appropriate.* |  |
| **G2. Is a survival analysis performed when there are individual differences in length of follow-up?** | | | | **Yes** |
|  | *Yes:* | *Unclear:* | *No:* |  |
|  | *Follow-up is the same for all study patients, if not survival analysis is performed.* | *Unclear whether there are different lengths of follow-up or whether they are taken into account.* | *Differences of follow up were ignored.* |  |
| **G3. If applicable, is composite outcome of harms adequately constructed?** | | | | **N/A** |
| *N/A* | *Yes:* | *Unclear:* | *No:* |  |
|  | *Composite outcome appropriate.* | *Unclear whether composite outcome is appropriate.* | *Construction of composite not described or not appropriate.* |  |
| **G6.** **Are the results consistent in primary and secondary analyses? Are confounding effects consistent with known associations?** | | | | **Yes** |
|  | *Yes:* | *Unclear:* | *No:* |  |
|  | *Consistency of primary, secondary analyses and consistency of confounding effects with known associations.* | *Not sufficient information to determine consistency.* | *No consistency of primary, secondary analyses or no consistency of confounding effects with known associations.* |  |
| **G. RISK OF BIAS ASSESSMENT FOR STATISTICAL METHODS EXCLUDING METHODS TO CONTROL CONFOUNDING:** | | | | **Unclear** |
|  | *Low:* | *Unclear:* | *High:* |  |
|  | *Plausible bias unlikely to seriously alter the results.* | *Plausible bias that raises some doubts about the results or when information on which to base risk of bias judgments is missing or poorly reported.* | *Plausible bias that seriously weakens confidence in the results.* |  |
| **Comments: Unclear risk of bias** | | | | |
|  | | | |  |
| **H. CONFLICT OF INTEREST** | | | | |
| **H1. Were the conflict of interest or sources of funding clearly acknowledged?** | | | | **Yes** |
|  | *Yes:* | *No:* |  |  |
|  | *Potential sources of support are acknowledged.* | *No sources of funding reported or not sufficient information.* |  |  |
| **H2. Does the study appear free of conflicts of interest susceptible to have influenced design, analysis or reporting (selective reporting of outcome or analysis)?** | | | | **Yes** |
|  | *Yes:* | *Unclear:* | *No:* |  |
|  | *No conflicts of interest or not susceptible to have influenced design, analysis or reporting.* | *It is unclear if there are conflicts of interest or if they are susceptible to have influenced design, analysis or reporting.* | *Conflicts of interest susceptible to have influenced design, analysis or reporting.* |  |
| **H. RISK OF BIAS ASSESSMENT FOR CONFLICT OF INTEREST** | | | | **Low** |
|  | *Low:* | *Unclear:* | *High:* |  |
|  | *Plausible bias unlikely to seriously alter the results.* | *Plausible bias that raises some doubts about the results or when information on which to base risk of bias judgments is missing or poorly reported.* | *Plausible bias that seriously weakens confidence in the results.* |  |
|  |  |  |  |  |
| **SUMMARY RISK-OF-BIAS ASSESSMENT FOR THE STUDY** | | | | |
| **RISK OF BIAS ASSESSMENT FOR THE STUDY** | | | | **Unclear** |
|  | *Low:* | *Unclear:* | *High:* |  |
|  | *Low risk of bias for all key domains.* | *Unclear risk of bias for one or more key domain.* | *High risk of bias for one or more key domains.* |  |

| **RISK OF BIAS ASSESSMENT CHECKLIST  FOR STUDIES INCLUDED IN SYSTEMATIC REVIEWS OF DRUG HARMS** | | | | |
| --- | --- | --- | --- | --- |
|  |  |  |  |  |
|  | **Study ID - Author** | Kumar et al (2012), EVOLUTION, NCT00507442 |  |  |
|  |  |  |  |  |
| **A. STUDY DESIGN AND OBJECTIVES** | | | | |
| **A1. Are study objectives clearly specified and appropriate?** | | | | **Yes** |
|  | *Yes:* | *No:* |  |  |
|  | *Study objectives clearly specified and appropriate.* | *Study objectives are not clearly specified or not appropriate.* |  |  |
| **A2. Is study design clearly specified and appropriate?** | | | | **Yes** |
|  | *Yes:* | *No:* |  |  |
|  | *Study design clearly specified and appropriate.* | *Study design not clearly specified or not appropriate.* |  |  |
| **A3. Is the study design free of run-in/lead-in period before inclusion/randomization of participants?** | | | | **Yes** |
|  | *Yes:* | *Unclear:* | *No:* |  |
|  | *No run-in/lead-in period.* | *Not clear information.* | *Presence of a run-in/lead-in period.* |  |
| **A4. Cross-over designs: Is the study designed to adequately address carry-over effect?** | | | | **N/A** |
| *N/A* | *Yes:* | *Unclear:* | *No:* |  |
|  | *Carry-over effect absent or adequately addressed (randomized order and sufficiently long wash-out period.* | *Not clear information.* | *Carry-over effect not adequately addressed and susceptible to bias the results.* |  |
| **A. RISK OF BIAS ASSESSMENT FOR STUDY DESIGN AND OBJECTIVES** | | | | **Low** |
|  | *Low:* | *Unclear:* | *High:* |  |
|  | *Plausible bias unlikely to seriously alter the results.* | *Plausible bias that raises some doubts about the results or when information on which to base risk of bias judgments is missing or poorly reported.* | *Plausible bias that seriously weakens confidence in the results.* |  |
| **Comments: Low risk of bias according to data provided by sources** | | | | |
|  |  |  |  |  |
| **B. BIAS IN SELECTION OF SUBJECTS AND CONSTITUTION OF STUDY GROUPS** | | | | |
| **B1. Was the method used to generate the allocation sequence adequate as to produce comparable groups?** | | | | **Yes** |
|  | *Yes:* | *Unclear:* | *No:* |  |
|  | *Allocation methods are adequate to produce comparable groups.* | *Allocation methods are not clearly reported.* | *Allocation methods are not adequate (e.g. assignment to treatment by birth date, week day, etc.), groups are not comparable.* |  |
| **B2. Was the method used to conceal the allocation sequence adequate as to produce comparable groups?** | | | | **Yes** |
|  | *Yes:* | *Unclear:* | *No:* |  |
|  | *Concealment is adequate.* | *Concealment methods are not clearly reported and groups may not be comparable.* | *Concealment methods are not adequate, groups are not comparable.* |  |
| **B3. Are all the subjects recruited from the same source population?** | | | | **Yes** |
|  | *Yes:* | *Unclear:* | *No:* |  |
|  | *All the subjects recruited from the same source population.* | *Unclear if all the subjects recruited from the same source population.* | *All the subjects are not recruited from the same source population.* |  |
| **B4. Were inclusion and exclusion criteria implemented uniformly across study groups?** | | | | **Yes** |
|  | *Yes:* | *Unclear:* | *No:* |  |
|  | *Selection criteria uniformly implemented.* | *Unclear if selection criteria are uniformly implemented.* | *Selection criteria not uniformly implemented.* |  |
| **B8. Are baseline characteristics and prognostic factors comparable between different groups?** | | | | **Yes** |
|  | *Yes:* | *Unclear:* | *No:* |  |
|  | *RCT: Groups are comparable at baseline.* | *No description of baseline characteristics or only significance tests.* | *The groups are unbalanced at baseline.* |  |
|  | *Cohort studies: Groups are comparable at baseline or matched for the main prognostic factors.* |  |  |  |
| **B. RISK OF BIAS ASSESSMENT FOR SELECTION OF PARTICIPANTS AND CONSTITUTION OF STUDY GROUPS** | | | | **Low** |
|  | *Low:* | *Unclear:* | *High:* |  |
|  | *Plausible bias unlikely to seriously alter the results.* | *Plausible bias that raises some doubts about the results or when information on which to base risk of bias judgments is missing or poorly reported.* | *Plausible bias that seriously weakens confidence in the results.* |  |
| **Comments: Low risk of bias according to data provided by sources** | | | | |
|  |  |  |  |  |
| **C. BIAS DUE TO WITHDRAWALS OR LOSS OF FOLLOW-UP (ATTRITION)** | | | | |
| **C1. Are the number of participants clearly reported throughout the study?** | | | | **Yes** |
|  | *Yes:* | *No:* |  |  |
|  | *Numbers of participants throughout the study are reported. Complete flow chart.* | *Numbers of patients at every stage is not clearly reported. Confusing information is reported regarding the number of participants. No or incomplete flow chart.* |  |  |
| **C2. Is the number of drop-outs/withdrawals due to harmful outcome clearly stated for each treatment arm?** | | | | **Yes** |
|  | *Yes:* | *No:* |  |  |
|  | *The number of drop-outs due to harmful outcome is specified.* | *The number of drop-outs due to harmful outcome is not specified, unclear or combined.* |  |  |
| **C3. Does the study adequately address biased loss to follow-up?** | | | | **yes** |
|  | *Yes:* | *Unclear:* | *No:* |  |
|  | *Complete follow-up or drop-outs unlikely to introduce bias or adequately controlled.* | *Drop-outs/withdraws due to harmful outcome are not clearly reported.* | *Loss to follow-up affects the safety outcome and is not adequately controlled.* |  |
| **C4. Are the results based on an intention-to-treat analysis?** | | | | **yes** |
|  | *Yes:* | *Unclear:* | *No:* |  |
|  | *Results are based on a strict intention-to-treat analysis.* | *Not clear if an intention-to-treat analysis is performed. No strict intention-to-treat analysis.* | *Results are not based on intention-to-treat analysis (not done or not possible).* |  |
| **C. RISK OF BIAS DUE TO WITHDRAWALS OR LOSS OF FOLLOW-UP (ATTRITION)** | | | | **Low** |
|  | *Low:* | *Unclear:* | *High:* |  |
|  | *Plausible bias unlikely to seriously alter the results.* | *Plausible bias that raises some doubts about the results or when information on which to base risk of bias judgments is missing or poorly reported.* | *Plausible bias that seriously weakens confidence in the results.* |  |
| **Comments: Low risk of bias according to data provided by sources** | | | | |
|  |  |  |  |  |
| **D. INFORMATION BIAS REGARDING THE HARMFULL OUTCOME** | | | | |
| **D1. Is the definition of the harmful outcome clearly stated?** | | | | **no** |
|  | *Yes:* | *No:* |  |  |
|  | *RCT: clear / standardized definition of the harmful outcome (e.g. diagnostic codes, clinical and laboratory data). Cohort studies: clear definition of the outcome. Case-control studies: clear definition of cases.* | *Definition of the harmful outcome not reported or that leads to confusion. Terms not well-constructed, wrong definition.* |  |  |
| **D2. If applicable, is the severity of the harmful outcome clearly stated?** | | | | **no** |
|  | *N/A:* | *Yes:* | *No:* |  |
|  | *Self evident severity (e.g. death).* | *Detailed degree of severity or reference to a known scale of severity or a new scale developed for the study.* | *Unclear degrees of severity or without clear boundaries between them.* |  |
| **D3. Was the blinding methods of participants regarding the intervention appropriate considering the nature of the harmful outcome?** | | | | **Unclear** |
|  | *Yes:* | *Unclear:* | *No:* |  |
|  | *Blinding ensured (and unlikely broken) or outcome not likely to be influenced by lack of blinding.* | *There is no sufficient information regarding the process of blinding or the outcome assessment.* | *No blinding (or incomplete blinding or risk of broken blinding) and outcome likely to be influenced by lack of blinding.* |  |
| **D4. Was the blinding methods of harmful outcome assessment appropriate considering the nature of the harmful outcome?** | | | | **Unclear** |
|  | *Yes:* | *Unclear:* | *No:* |  |
|  | *Blinding ensured (and unlikely broken) or outcome assessment not likely to be influenced by lack of blinding.* | *There is no sufficient information regarding the process of blinding of outcome assessment.* | *No blinding (or incomplete blinding or risk of broken blinding) and outcome likely to be influenced by lack of blinding.* |  |
| **D5. Was the duration of follow-up adequate to assess the harmful outcome?** | | | | **Unclear** |
|  | *Yes:* | *Unclear:* | *No:* |  |
|  | *Sufficient duration of follow-up to assess the outcome.* | *It is unclear whether the duration of follow-up is adequate.* | *Too short duration of follow-up.* |  |
| **D6. Was the methods for ascertaining the harmful outcome adequately constructed and equal for all participants?** | | | | **Unclear** |
|  | *Yes:* | *Unclear:* | *No:* |  |
|  | *Adequate or validated methods of outcome measurement for all participants. Clinical reactions medically confirmed by a physician. Minimized risk of misclassification or differential assessment, reporting or detection.  RCT : Active harmful outcome surveillance (prospective/retrospective case-record review, questionnaires, patient’s diary/checklist…) .* | *There is no or not sufficient information to clearly determine how information on harmful outcome is collected or the process of minimizing misclassification.* | *Substantial risk of misclassification of outcome or differential assessment, reporting or detection. Clinical reactions not medically confirmed. RCT : Passive harmful outcome surveillance (patient’s volunteer reporting).* |  |
| **D7. Are the number of harmful outcome and the number of patients with a harmful outcome reported in both treatment arms?** | | | | **no** |
|  | *Yes:* | *Unclear:* | *No:* |  |
|  | *Numbers are reported. It is possible to calculate the rates of harmful outcome.* | *Confusion between the number of harmful outcomes or the number of patients with a harmful outcome, or general statements such as “5% of patients developed a harmful outcome”.* | *Neither the number of harmful outcomes nor the number of patients with a harmful outcome is reported. Or numbers are combining both treatment arms.* |  |
| **D8. Is the time frequency of harmful outcome assessment during the follow-up period appropriate?** | | | | **No** |
|  | *Yes:* | *Unclear:* | *No:* |  |
|  | *For all study groups, the time frequency at which the harmful outcome is assessed is appropriate.* | *General statements such as “patients were routinely assessed for harmful outcomes”.* | *There is no regular collection of data on harmful outcomes during the study.* |  |
| **D9. Was the time between the exposure to a drug and the onset of the harmful outcome reported?** | | | | **No** |
|  | *Yes:* | *Unclear:* | *No:* |  |
|  | *The time between the drug exposure to the onset of harmful outcome is specified.* | *The authors do not report a clear time frame between drug exposure and harmful outcome.* | *The authors do not report the time between the drug exposure to the onset of harmful outcome.* |  |
| **D10. Was the process of determining that the harmful outcome is linked to the drug appropriate? Was the process blinded to the assigned treatment?** | | | | **No** |
|  | *Yes:* | *Unclear:* | *No:* |  |
|  | *Methods for causality assessment are appropriate and, if applicable, made by investigators blinded to the intervention.* | *Unclear how the causality attribution is made. It is not clear who make the assessment or whether it is blinded to the assigned treatment.* | *Causality assessment is made by investigators not blinded to the intervention, or by participants or sponsors, or unblinding of treatment assignment precedes the decision to withdraw.* |  |
| **D. RISK OF BIAS ASSESSMENT FOR INFORMATION BIAS REGARDING THE HARMFULL OUTCOME** | | | | **Unclear** |
|  | *Low:* | *Unclear:* | *High:* |  |
|  | *Plausible bias unlikely to seriously alter the results.* | *Plausible bias that raises some doubts about the results or when information on which to base risk of bias judgments is missing or poorly reported.* | *Plausible bias that seriously weakens confidence in the results.* |  |
| **Comments: Unclear risk of bias** | | | | |
| **E. OTHER INFORMATION BIAS** | | | | |
| **E1. Is blinding of care givers during follow-up adequately performed in order to avoid differential care between study groups (performance bias)?** | | | | **Unclear** |
|  | *Yes:* | *Unclear:* | *No:* |  |
|  | *There is no risk of differential care or it is adequately addressed.* | *Unclear risk of bias due to differential care.* | *The bias due to differential care is not controlled.* |  |
| **E5. Does the study appear free of other information bias ?** | | | | **Unclear** |
|  | *Yes:* | *Unclear:* | *No:* |  |
|  | *The study appears to be free of other information bias.* | *Unclear presence of other information bias.* | *Additional source of other information bias.* |  |
| **E. RISK OF BIAS ASSESSMENT FOR OTHER INFORMATION BIAS** | | | | **Unclear** |
|  | *Low:* | *Unclear:* | *High:* |  |
|  | *Plausible bias unlikely to seriously alter the results.* | *Plausible bias that raises some doubts about the results or when information on which to base risk of bias judgments is missing or poorly reported.* | *Plausible bias that seriously weakens confidence in the results.* |  |
| **Comments: unclear of bias -> open label** | | | | |
|  |  |  |  |  |
| **F. STATISTICAL METHODS TO CONTROL CONFOUNDING** | | | | |
| **F5. Does the study adequately address residual or unmeasured confounding?** | | | | **Yes** |
|  | *Yes:* | *Unclear:* | *No:* |  |
|  | *The study adequately addresses residual or unmeasured confounding.* | *Unclear presence of residual or unmeasured confounding.* | *Residual or unmeasured confounding is likely to be important.* |  |
| **F. RISK OF BIAS ASSESSMENT FOR STATISTICAL METHODS TO CONTROL CONFOUNDING:** | | | | **Low** |
|  | *Low:* | *Unclear:* | *High:* |  |
|  | *Plausible bias unlikely to seriously alter the results.* | *Plausible bias that raises some doubts about the results or when information on which to base risk of bias judgments is missing or poorly reported.* | *Plausible bias that seriously weakens confidence in the results.* |  |
| **Comments: Low risk of bias->Statistical Analysis section is well informative ( stratification by International Staging System (ISS) and whether, in the opinion of the treating physician, the patient was eligible for ASCT)** | | | | |
|  |  |  |  |  |
| **G. STATISTICAL METHODS EXCLUDING METHODS TO CONTROL CONFOUNDING** | | | | |
| **G1. Are the statistical methods used to analyze the harmful outcome appropriate?** | | | | **Unclear** |
|  | *Yes:* | *Unclear:* | *No:* |  |
|  | *Statistical techniques are appropriate to the data. If the distribution of the data (normal or not) is not described, it must be assumed that the estimates used were appropriate.* | *There is no description of the statistical techniques used, or the description is vague and not understandable.* | *The statistical techniques used are not appropriate.* |  |
| **G2. Is a survival analysis performed when there are individual differences in length of follow-up?** | | | | **Unclear** |
|  | *Yes:* | *Unclear:* | *No:* |  |
|  | *Follow-up is the same for all study patients, if not survival analysis is performed.* | *Unclear whether there are different lengths of follow-up or whether they are taken into account.* | *Differences of follow up were ignored.* |  |
| **G3. If applicable, is composite outcome of harms adequately constructed?** | | | | **N/A** |
| *N/A* | *Yes:* | *Unclear:* | *No:* |  |
|  | *Composite outcome appropriate.* | *Unclear whether composite outcome is appropriate.* | *Construction of composite not described or not appropriate.* |  |
| **G6.** **Are the results consistent in primary and secondary analyses? Are confounding effects consistent with known associations?** | | | | **Yes** |
|  | *Yes:* | *Unclear:* | *No:* |  |
|  | *Consistency of primary, secondary analyses and consistency of confounding effects with known associations.* | *Not sufficient information to determine consistency.* | *No consistency of primary, secondary analyses or no consistency of confounding effects with known associations.* |  |
| **G. RISK OF BIAS ASSESSMENT FOR STATISTICAL METHODS EXCLUDING METHODS TO CONTROL CONFOUNDING:** | | | | **Unclear** |
|  | *Low:* | *Unclear:* | *High:* |  |
|  | *Plausible bias unlikely to seriously alter the results.* | *Plausible bias that raises some doubts about the results or when information on which to base risk of bias judgments is missing or poorly reported.* | *Plausible bias that seriously weakens confidence in the results.* |  |
| **Comments: Unclear risk of bias** | | | | |
|  | | | |  |
| **H. CONFLICT OF INTEREST** | | | | |
| **H1. Were the conflict of interest or sources of funding clearly acknowledged?** | | | | **Yes** |
|  | *Yes:* | *No:* |  |  |
|  | *Potential sources of support are acknowledged.* | *No sources of funding reported or not sufficient information.* |  |  |
| **H2. Does the study appear free of conflicts of interest susceptible to have influenced design, analysis or reporting (selective reporting of outcome or analysis)?** | | | | **Yes** |
|  | *Yes:* | *Unclear:* | *No:* |  |
|  | *No conflicts of interest or not susceptible to have influenced design, analysis or reporting.* | *It is unclear if there are conflicts of interest or if they are susceptible to have influenced design, analysis or reporting.* | *Conflicts of interest susceptible to have influenced design, analysis or reporting.* |  |
| **H. RISK OF BIAS ASSESSMENT FOR CONFLICT OF INTEREST** | | | | **Low** |
|  | *Low:* | *Unclear:* | *High:* |  |
|  | *Plausible bias unlikely to seriously alter the results.* | *Plausible bias that raises some doubts about the results or when information on which to base risk of bias judgments is missing or poorly reported.* | *Plausible bias that seriously weakens confidence in the results.* |  |
|  |  |  |  |  |
| **SUMMARY RISK-OF-BIAS ASSESSMENT FOR THE STUDY** | | | | |
| **RISK OF BIAS ASSESSMENT FOR THE STUDY** | | | | **Unclear** |
|  | *Low:* | *Unclear:* | *High:* |  |
|  | *Low risk of bias for all key domains.* | *Unclear risk of bias for one or more key domain.* | *High risk of bias for one or more key domains.* |  |

| **RISK OF BIAS ASSESSMENT CHECKLIST  FOR STUDIES INCLUDED IN SYSTEMATIC REVIEWS OF DRUG HARMS** | | | | |
| --- | --- | --- | --- | --- |
|  |  |  |  |  |
|  | **Study ID - Author** | Lonial et al (2019), ECOG-E3A06, NCT01169337 |  |  |
|  |  |  |  |  |
| **A. STUDY DESIGN AND OBJECTIVES** | | | | |
| **A1. Are study objectives clearly specified and appropriate?** | | | | **Yes** |
|  | *Yes:* | *No:* |  |  |
|  | *Study objectives clearly specified and appropriate.* | *Study objectives are not clearly specified or not appropriate.* |  |  |
| **A2. Is study design clearly specified and appropriate?** | | | | **Yes** |
|  | *Yes:* | *No:* |  |  |
|  | *Study design clearly specified and appropriate.* | *Study design not clearly specified or not appropriate.* |  |  |
| **A3. Is the study design free of run-in/lead-in period before inclusion/randomization of participants?** | | | | **Yes** |
|  | *Yes:* | *Unclear:* | *No:* |  |
|  | *No run-in/lead-in period.* | *Not clear information.* | *Presence of a run-in/lead-in period.* |  |
| **A4. Cross-over designs: Is the study designed to adequately address carry-over effect?** | | | | **Yes** |
| *N/A* | *Yes:* | *Unclear:* | *No:* |  |
|  | *Carry-over effect absent or adequately addressed (randomized order and sufficiently long wash-out period.* | *Not clear information.* | *Carry-over effect not adequately addressed and susceptible to bias the results.* |  |
| **A. RISK OF BIAS ASSESSMENT FOR STUDY DESIGN AND OBJECTIVES** | | | | **Low** |
|  | *Low:* | *Unclear:* | *High:* |  |
|  | *Plausible bias unlikely to seriously alter the results.* | *Plausible bias that raises some doubts about the results or when information on which to base risk of bias judgments is missing or poorly reported.* | *Plausible bias that seriously weakens confidence in the results.* |  |
| **Comments: Low risk of bias according to data provided by sources** | | | | |
|  |  |  |  |  |
| **B. BIAS IN SELECTION OF SUBJECTS AND CONSTITUTION OF STUDY GROUPS** | | | | |
| **B1. Was the method used to generate the allocation sequence adequate as to produce comparable groups?** | | | | **Yes** |
|  | *Yes:* | *Unclear:* | *No:* |  |
|  | *Allocation methods are adequate to produce comparable groups.* | *Allocation methods are not clearly reported.* | *Allocation methods are not adequate (e.g. assignment to treatment by birth date, week day, etc.), groups are not comparable.* |  |
| **B2. Was the method used to conceal the allocation sequence adequate as to produce comparable groups?** | | | | **Yes** |
|  | *Yes:* | *Unclear:* | *No:* |  |
|  | *Concealment is adequate.* | *Concealment methods are not clearly reported and groups may not be comparable.* | *Concealment methods are not adequate, groups are not comparable.* |  |
| **B3. Are all the subjects recruited from the same source population?** | | | | **Yes** |
|  | *Yes:* | *Unclear:* | *No:* |  |
|  | *All the subjects recruited from the same source population.* | *Unclear if all the subjects recruited from the same source population.* | *All the subjects are not recruited from the same source population.* |  |
| **B4. Were inclusion and exclusion criteria implemented uniformly across study groups?** | | | | **Yes** |
|  | *Yes:* | *Unclear:* | *No:* |  |
|  | *Selection criteria uniformly implemented.* | *Unclear if selection criteria are uniformly implemented.* | *Selection criteria not uniformly implemented.* |  |
| **B8. Are baseline characteristics and prognostic factors comparable between different groups?** | | | | **Yes** |
|  | *Yes:* | *Unclear:* | *No:* |  |
|  | *RCT: Groups are comparable at baseline.* | *No description of baseline characteristics or only significance tests.* | *The groups are unbalanced at baseline.* |  |
|  | *Cohort studies: Groups are comparable at baseline or matched for the main prognostic factors.* |  |  |  |
| **B. RISK OF BIAS ASSESSMENT FOR SELECTION OF PARTICIPANTS AND CONSTITUTION OF STUDY GROUPS** | | | | **Low** |
|  | *Low:* | *Unclear:* | *High:* |  |
|  | *Plausible bias unlikely to seriously alter the results.* | *Plausible bias that raises some doubts about the results or when information on which to base risk of bias judgments is missing or poorly reported.* | *Plausible bias that seriously weakens confidence in the results.* |  |
| **Comments: Low risk of bias according to data provided by sources** | | | | |
|  |  |  |  |  |
| **C. BIAS DUE TO WITHDRAWALS OR LOSS OF FOLLOW-UP (ATTRITION)** | | | | |
| **C1. Are the number of participants clearly reported throughout the study?** | | | | **Yes** |
|  | *Yes:* | *No:* |  |  |
|  | *Numbers of participants throughout the study are reported. Complete flow chart.* | *Numbers of patients at every stage is not clearly reported. Confusing information is reported regarding the number of participants. No or incomplete flow chart.* |  |  |
| **C2. Is the number of drop-outs/withdrawals due to harmful outcome clearly stated for each treatment arm?** | | | | **yes** |
|  | *Yes:* | *No:* |  |  |
|  | *The number of drop-outs due to harmful outcome is specified.* | *The number of drop-outs due to harmful outcome is not specified, unclear or combined.* |  |  |
| **C3. Does the study adequately address biased loss to follow-up?** | | | | **yes** |
|  | *Yes:* | *Unclear:* | *No:* |  |
|  | *Complete follow-up or drop-outs unlikely to introduce bias or adequately controlled.* | *Drop-outs/withdraws due to harmful outcome are not clearly reported.* | *Loss to follow-up affects the safety outcome and is not adequately controlled.* |  |
| **C4. Are the results based on an intention-to-treat analysis?** | | | | **yes** |
|  | *Yes:* | *Unclear:* | *No:* |  |
|  | *Results are based on a strict intention-to-treat analysis.* | *Not clear if an intention-to-treat analysis is performed. No strict intention-to-treat analysis.* | *Results are not based on intention-to-treat analysis (not done or not possible).* |  |
| **C. RISK OF BIAS DUE TO WITHDRAWALS OR LOSS OF FOLLOW-UP (ATTRITION)** | | | | **Low** |
|  | *Low:* | *Unclear:* | *High:* |  |
|  | *Plausible bias unlikely to seriously alter the results.* | *Plausible bias that raises some doubts about the results or when information on which to base risk of bias judgments is missing or poorly reported.* | *Plausible bias that seriously weakens confidence in the results.* |  |
| **Comments: Low risk of bias according to data provided by sources** | | | | |
|  |  |  |  |  |
| **D. INFORMATION BIAS REGARDING THE HARMFULL OUTCOME** | | | | |
| **D1. Is the definition of the harmful outcome clearly stated?** | | | | **No** |
|  | *Yes:* | *No:* |  |  |
|  | *RCT: clear / standardized definition of the harmful outcome (e.g. diagnostic codes, clinical and laboratory data). Cohort studies: clear definition of the outcome. Case-control studies: clear definition of cases.* | *Definition of the harmful outcome not reported or that leads to confusion. Terms not well-constructed, wrong definition.* |  |  |
| **D2. If applicable, is the severity of the harmful outcome clearly stated?** | | | | **No** |
|  | *N/A:* | *Yes:* | *No:* |  |
|  | *Self evident severity (e.g. death).* | *Detailed degree of severity or reference to a known scale of severity or a new scale developed for the study.* | *Unclear degrees of severity or without clear boundaries between them.* |  |
| **D3. Was the blinding methods of participants regarding the intervention appropriate considering the nature of the harmful outcome?** | | | | **Unclear** |
|  | *Yes:* | *Unclear:* | *No:* |  |
|  | *Blinding ensured (and unlikely broken) or outcome not likely to be influenced by lack of blinding.* | *There is no sufficient information regarding the process of blinding or the outcome assessment.* | *No blinding (or incomplete blinding or risk of broken blinding) and outcome likely to be influenced by lack of blinding.* |  |
| **D4. Was the blinding methods of harmful outcome assessment appropriate considering the nature of the harmful outcome?** | | | | **Unclear** |
|  | *Yes:* | *Unclear:* | *No:* |  |
|  | *Blinding ensured (and unlikely broken) or outcome assessment not likely to be influenced by lack of blinding.* | *There is no sufficient information regarding the process of blinding of outcome assessment.* | *No blinding (or incomplete blinding or risk of broken blinding) and outcome likely to be influenced by lack of blinding.* |  |
| **D5. Was the duration of follow-up adequate to assess the harmful outcome?** | | | | **Unclear** |
|  | *Yes:* | *Unclear:* | *No:* |  |
|  | *Sufficient duration of follow-up to assess the outcome.* | *It is unclear whether the duration of follow-up is adequate.* | *Too short duration of follow-up.* |  |
| **D6. Was the methods for ascertaining the harmful outcome adequately constructed and equal for all participants?** | | | | **Unclear** |
|  | *Yes:* | *Unclear:* | *No:* |  |
|  | *Adequate or validated methods of outcome measurement for all participants. Clinical reactions medically confirmed by a physician. Minimized risk of misclassification or differential assessment, reporting or detection.  RCT : Active harmful outcome surveillance (prospective/retrospective case-record review, questionnaires, patient’s diary/checklist…) .* | *There is no or not sufficient information to clearly determine how information on harmful outcome is collected or the process of minimizing misclassification.* | *Substantial risk of misclassification of outcome or differential assessment, reporting or detection. Clinical reactions not medically confirmed. RCT : Passive harmful outcome surveillance (patient’s volunteer reporting).* |  |
| **D7. Are the number of harmful outcome and the number of patients with a harmful outcome reported in both treatment arms?** | | | | **Unclear** |
|  | *Yes:* | *Unclear:* | *No:* |  |
|  | *Numbers are reported. It is possible to calculate the rates of harmful outcome.* | *Confusion between the number of harmful outcomes or the number of patients with a harmful outcome, or general statements such as “5% of patients developed a harmful outcome”.* | *Neither the number of harmful outcomes nor the number of patients with a harmful outcome is reported. Or numbers are combining both treatment arms.* |  |
| **D8. Is the time frequency of harmful outcome assessment during the follow-up period appropriate?** | | | | **Unclear** |
|  | *Yes:* | *Unclear:* | *No:* |  |
|  | *For all study groups, the time frequency at which the harmful outcome is assessed is appropriate.* | *General statements such as “patients were routinely assessed for harmful outcomes”.* | *There is no regular collection of data on harmful outcomes during the study.* |  |
| **D9. Was the time between the exposure to a drug and the onset of the harmful outcome reported?** | | | | **Unclear** |
|  | *Yes:* | *Unclear:* | *No:* |  |
|  | *The time between the drug exposure to the onset of harmful outcome is specified.* | *The authors do not report a clear time frame between drug exposure and harmful outcome.* | *The authors do not report the time between the drug exposure to the onset of harmful outcome.* |  |
| **D10. Was the process of determining that the harmful outcome is linked to the drug appropriate? Was the process blinded to the assigned treatment?** | | | | **Unclear** |
|  | *Yes:* | *Unclear:* | *No:* |  |
|  | *Methods for causality assessment are appropriate and, if applicable, made by investigators blinded to the intervention.* | *Unclear how the causality attribution is made. It is not clear who make the assessment or whether it is blinded to the assigned treatment.* | *Causality assessment is made by investigators not blinded to the intervention, or by participants or sponsors, or unblinding of treatment assignment precedes the decision to withdraw.* |  |
| **D. RISK OF BIAS ASSESSMENT FOR INFORMATION BIAS REGARDING THE HARMFULL OUTCOME** | | | | **Unclear** |
|  | *Low:* | *Unclear:* | *High:* |  |
|  | *Plausible bias unlikely to seriously alter the results.* | *Plausible bias that raises some doubts about the results or when information on which to base risk of bias judgments is missing or poorly reported.* | *Plausible bias that seriously weakens confidence in the results.* |  |
| **Comments: Unclear risk of bias** | | | | |
|  |  |  |  |  |
| **E. OTHER INFORMATION BIAS** | | | | |
| **E1. Is blinding of care givers during follow-up adequately performed in order to avoid differential care between study groups (performance bias)?** | | | | **Unclear** |
|  | *Yes:* | *Unclear:* | *No:* |  |
|  | *There is no risk of differential care or it is adequately addressed.* | *Unclear risk of bias due to differential care.* | *The bias due to differential care is not controlled.* |  |
| **E5. Does the study appear free of other information bias ?** | | | | **Unclear** |
|  | *Yes:* | *Unclear:* | *No:* |  |
|  | *The study appears to be free of other information bias.* | *Unclear presence of other information bias.* | *Additional source of other information bias.* |  |
| **E. RISK OF BIAS ASSESSMENT FOR OTHER INFORMATION BIAS** | | | | **Unclear** |
|  | *Low:* | *Unclear:* | *High:* |  |
|  | *Plausible bias unlikely to seriously alter the results.* | *Plausible bias that raises some doubts about the results or when information on which to base risk of bias judgments is missing or poorly reported.* | *Plausible bias that seriously weakens confidence in the results.* |  |
| **Comments: unclear of bias -> open label** | | | | |
|  |  |  |  |  |
| **F. STATISTICAL METHODS TO CONTROL CONFOUNDING** | | | | |
| **F5. Does the study adequately address residual or unmeasured confounding?** | | | | **Yes** |
|  | *Yes:* | *Unclear:* | *No:* |  |
|  | *The study adequately addresses residual or unmeasured confounding.* | *Unclear presence of residual or unmeasured confounding.* | *Residual or unmeasured confounding is likely to be important.* |  |
| **F. RISK OF BIAS ASSESSMENT FOR STATISTICAL METHODS TO CONTROL CONFOUNDING:** | | | | **Low** |
|  | *Low:* | *Unclear:* | *High:* |  |
|  | *Plausible bias unlikely to seriously alter the results.* | *Plausible bias that raises some doubts about the results or when information on which to base risk of bias judgments is missing or poorly reported.* | *Plausible bias that seriously weakens confidence in the results.* |  |
| **Comments: Low risk of bias->Statistical Analysis section is well informative (stratification by time since diagnosis ofSMM(<1 year v. >1 year)** | | | | |
|  |  |  |  |  |
| **G. STATISTICAL METHODS EXCLUDING METHODS TO CONTROL CONFOUNDING** | | | | |
| **G1. Are the statistical methods used to analyze the harmful outcome appropriate?** | | | | **Unclear** |
|  | *Yes:* | *Unclear:* | *No:* |  |
|  | *Statistical techniques are appropriate to the data. If the distribution of the data (normal or not) is not described, it must be assumed that the estimates used were appropriate.* | *There is no description of the statistical techniques used, or the description is vague and not understandable.* | *The statistical techniques used are not appropriate.* |  |
| **G2. Is a survival analysis performed when there are individual differences in length of follow-up?** | | | | **Yes** |
|  | *Yes:* | *Unclear:* | *No:* |  |
|  | *Follow-up is the same for all study patients, if not survival analysis is performed.* | *Unclear whether there are different lengths of follow-up or whether they are taken into account.* | *Differences of follow up were ignored.* |  |
| **G3. If applicable, is composite outcome of harms adequately constructed?** | | | | **Unclear** |
| *N/A* | *Yes:* | *Unclear:* | *No:* |  |
|  | *Composite outcome appropriate.* | *Unclear whether composite outcome is appropriate.* | *Construction of composite not described or not appropriate.* |  |
| **G6.** **Are the results consistent in primary and secondary analyses? Are confounding effects consistent with known associations?** | | | | **Yes** |
|  | *Yes:* | *Unclear:* | *No:* |  |
|  | *Consistency of primary, secondary analyses and consistency of confounding effects with known associations.* | *Not sufficient information to determine consistency.* | *No consistency of primary, secondary analyses or no consistency of confounding effects with known associations.* |  |
| **G. RISK OF BIAS ASSESSMENT FOR STATISTICAL METHODS EXCLUDING METHODS TO CONTROL CONFOUNDING:** | | | | **Unclear** |
|  | *Low:* | *Unclear:* | *High:* |  |
|  | *Plausible bias unlikely to seriously alter the results.* | *Plausible bias that raises some doubts about the results or when information on which to base risk of bias judgments is missing or poorly reported.* | *Plausible bias that seriously weakens confidence in the results.* |  |
| **Comments: Unclear risk of bias** | | | | |
|  | | | |  |
| **H. CONFLICT OF INTEREST** | | | | |
| **H1. Were the conflict of interest or sources of funding clearly acknowledged?** | | | | **Yes** |
|  | *Yes:* | *No:* |  |  |
|  | *Potential sources of support are acknowledged.* | *No sources of funding reported or not sufficient information.* |  |  |
| **H2. Does the study appear free of conflicts of interest susceptible to have influenced design, analysis or reporting (selective reporting of outcome or analysis)?** | | | | **Yes** |
|  | *Yes:* | *Unclear:* | *No:* |  |
|  | *No conflicts of interest or not susceptible to have influenced design, analysis or reporting.* | *It is unclear if there are conflicts of interest or if they are susceptible to have influenced design, analysis or reporting.* | *Conflicts of interest susceptible to have influenced design, analysis or reporting.* |  |
| **H. RISK OF BIAS ASSESSMENT FOR CONFLICT OF INTEREST** | | | | **Low** |
|  | *Low:* | *Unclear:* | *High:* |  |
|  | *Plausible bias unlikely to seriously alter the results.* | *Plausible bias that raises some doubts about the results or when information on which to base risk of bias judgments is missing or poorly reported.* | *Plausible bias that seriously weakens confidence in the results.* |  |
|  |  |  |  |  |
| **SUMMARY RISK-OF-BIAS ASSESSMENT FOR THE STUDY** | | | | |
| **RISK OF BIAS ASSESSMENT FOR THE STUDY** | | | | **Unclear** |
|  | *Low:* | *Unclear:* | *High:* |  |
|  | *Low risk of bias for all key domains.* | *Unclear risk of bias for one or more key domain.* | *High risk of bias for one or more key domains.* |  |

| **RISK OF BIAS ASSESSMENT CHECKLIST  FOR STUDIES INCLUDED IN SYSTEMATIC REVIEWS OF DRUG HARMS** | | | | |
| --- | --- | --- | --- | --- |
|  |  |  |  |  |
|  | **Study ID - Author** | Mateos et al (2022), QuiReDex, NCT00480363 |  |  |
|  |  |  |  |  |
| **A. STUDY DESIGN AND OBJECTIVES** | | | | |
| **A1. Are study objectives clearly specified and appropriate?** | | | | **Yes** |
|  | *Yes:* | *No:* |  |  |
|  | *Study objectives clearly specified and appropriate.* | *Study objectives are not clearly specified or not appropriate.* |  |  |
| **A2. Is study design clearly specified and appropriate?** | | | | **Yes** |
|  | *Yes:* | *No:* |  |  |
|  | *Study design clearly specified and appropriate.* | *Study design not clearly specified or not appropriate.* |  |  |
| **A3. Is the study design free of run-in/lead-in period before inclusion/randomization of participants?** | | | | **Yes** |
|  | *Yes:* | *Unclear:* | *No:* |  |
|  | *No run-in/lead-in period.* | *Not clear information.* | *Presence of a run-in/lead-in period.* |  |
| **A4. Cross-over designs: Is the study designed to adequately address carry-over effect?** | | | | **N/A** |
| *N/A* | *Yes:* | *Unclear:* | *No:* |  |
|  | *Carry-over effect absent or adequately addressed (randomized order and sufficiently long wash-out period.* | *Not clear information.* | *Carry-over effect not adequately addressed and susceptible to bias the results.* |  |
| **A. RISK OF BIAS ASSESSMENT FOR STUDY DESIGN AND OBJECTIVES** | | | | **Low** |
|  | *Low:* | *Unclear:* | *High:* |  |
|  | *Plausible bias unlikely to seriously alter the results.* | *Plausible bias that raises some doubts about the results or when information on which to base risk of bias judgments is missing or poorly reported.* | *Plausible bias that seriously weakens confidence in the results.* |  |
| **Comments: Low risk of bias according to data provided by sources** | | | | |
|  |  |  |  |  |
| **B. BIAS IN SELECTION OF SUBJECTS AND CONSTITUTION OF STUDY GROUPS** | | | | |
| **B1. Was the method used to generate the allocation sequence adequate as to produce comparable groups?** | | | | **Unclear** |
|  | *Yes:* | *Unclear:* | *No:* |  |
|  | *Allocation methods are adequate to produce comparable groups.* | *Allocation methods are not clearly reported.* | *Allocation methods are not adequate (e.g. assignment to treatment by birth date, week day, etc.), groups are not comparable.* |  |
| **B2. Was the method used to conceal the allocation sequence adequate as to produce comparable groups?** | | | | **Yes** |
|  | *Yes:* | *Unclear:* | *No:* |  |
|  | *Concealment is adequate.* | *Concealment methods are not clearly reported and groups may not be comparable.* | *Concealment methods are not adequate, groups are not comparable.* |  |
| **B3. Are all the subjects recruited from the same source population?** | | | | **Yes** |
|  | *Yes:* | *Unclear:* | *No:* |  |
|  | *All the subjects recruited from the same source population.* | *Unclear if all the subjects recruited from the same source population.* | *All the subjects are not recruited from the same source population.* |  |
| **B4. Were inclusion and exclusion criteria implemented uniformly across study groups?** | | | | **Yes** |
|  | *Yes:* | *Unclear:* | *No:* |  |
|  | *Selection criteria uniformly implemented.* | *Unclear if selection criteria are uniformly implemented.* | *Selection criteria not uniformly implemented.* |  |
| **B8. Are baseline characteristics and prognostic factors comparable between different groups?** | | | | **Yes** |
|  | *Yes:* | *Unclear:* | *No:* |  |
|  | *RCT: Groups are comparable at baseline.* | *No description of baseline characteristics or only significance tests.* | *The groups are unbalanced at baseline.* |  |
|  | *Cohort studies: Groups are comparable at baseline or matched for the main prognostic factors.* |  |  |  |
| **B. RISK OF BIAS ASSESSMENT FOR SELECTION OF PARTICIPANTS AND CONSTITUTION OF STUDY GROUPS** | | | | **Unclear** |
|  | *Low:* | *Unclear:* | *High:* |  |
|  | *Plausible bias unlikely to seriously alter the results.* | *Plausible bias that raises some doubts about the results or when information on which to base risk of bias judgments is missing or poorly reported.* | *Plausible bias that seriously weakens confidence in the results.* |  |
| **Comments: unclear risk of bias according to data provided by sources** | | | | |
|  |  |  |  |  |
| **C. BIAS DUE TO WITHDRAWALS OR LOSS OF FOLLOW-UP (ATTRITION)** | | | | |
| **C1. Are the number of participants clearly reported throughout the study?** | | | | **Yes** |
|  | *Yes:* | *No:* |  |  |
|  | *Numbers of participants throughout the study are reported. Complete flow chart.* | *Numbers of patients at every stage is not clearly reported. Confusing information is reported regarding the number of participants. No or incomplete flow chart.* |  |  |
| **C2. Is the number of drop-outs/withdrawals due to harmful outcome clearly stated for each treatment arm?** | | | | **Yes** |
|  | *Yes:* | *No:* |  |  |
|  | *The number of drop-outs due to harmful outcome is specified.* | *The number of drop-outs due to harmful outcome is not specified, unclear or combined.* |  |  |
| **C3. Does the study adequately address biased loss to follow-up?** | | | | **Yes** |
|  | *Yes:* | *Unclear:* | *No:* |  |
|  | *Complete follow-up or drop-outs unlikely to introduce bias or adequately controlled.* | *Drop-outs/withdraws due to harmful outcome are not clearly reported.* | *Loss to follow-up affects the safety outcome and is not adequately controlled.* |  |
| **C4. Are the results based on an intention-to-treat analysis?** | | | | **Yes** |
|  | *Yes:* | *Unclear:* | *No:* |  |
|  | *Results are based on a strict intention-to-treat analysis.* | *Not clear if an intention-to-treat analysis is performed. No strict intention-to-treat analysis.* | *Results are not based on intention-to-treat analysis (not done or not possible).* |  |
| **C. RISK OF BIAS DUE TO WITHDRAWALS OR LOSS OF FOLLOW-UP (ATTRITION)** | | | | **Yes** |
|  | *Low:* | *Unclear:* | *High:* |  |
|  | *Plausible bias unlikely to seriously alter the results.* | *Plausible bias that raises some doubts about the results or when information on which to base risk of bias judgments is missing or poorly reported.* | *Plausible bias that seriously weakens confidence in the results.* |  |
| **Comments: low risk of bias** | | | | |
|  |  |  |  |  |
| **D. INFORMATION BIAS REGARDING THE HARMFULL OUTCOME** | | | | |
| **D1. Is the definition of the harmful outcome clearly stated?** | | | | **No** |
|  | *Yes:* | *No:* |  |  |
|  | *RCT: clear / standardized definition of the harmful outcome (e.g. diagnostic codes, clinical and laboratory data). Cohort studies: clear definition of the outcome. Case-control studies: clear definition of cases.* | *Definition of the harmful outcome not reported or that leads to confusion. Terms not well-constructed, wrong definition.* |  |  |
| **D2. If applicable, is the severity of the harmful outcome clearly stated?** | | | | **N/A** |
|  | *N/A:* | *Yes:* | *No:* |  |
|  | *Self evident severity (e.g. death).* | *Detailed degree of severity or reference to a known scale of severity or a new scale developed for the study.* | *Unclear degrees of severity or without clear boundaries between them.* |  |
| **D3. Was the blinding methods of participants regarding the intervention appropriate considering the nature of the harmful outcome?** | | | | **Unclear** |
|  | *Yes:* | *Unclear:* | *No:* |  |
|  | *Blinding ensured (and unlikely broken) or outcome not likely to be influenced by lack of blinding.* | *There is no sufficient information regarding the process of blinding or the outcome assessment.* | *No blinding (or incomplete blinding or risk of broken blinding) and outcome likely to be influenced by lack of blinding.* |  |
| **D4. Was the blinding methods of harmful outcome assessment appropriate considering the nature of the harmful outcome?** | | | | **Unclear** |
|  | *Yes:* | *Unclear:* | *No:* |  |
|  | *Blinding ensured (and unlikely broken) or outcome assessment not likely to be influenced by lack of blinding.* | *There is no sufficient information regarding the process of blinding of outcome assessment.* | *No blinding (or incomplete blinding or risk of broken blinding) and outcome likely to be influenced by lack of blinding.* |  |
| **D5. Was the duration of follow-up adequate to assess the harmful outcome?** | | | | **Unclear** |
|  | *Yes:* | *Unclear:* | *No:* |  |
|  | *Sufficient duration of follow-up to assess the outcome.* | *It is unclear whether the duration of follow-up is adequate.* | *Too short duration of follow-up.* |  |
| **D6. Was the methods for ascertaining the harmful outcome adequately constructed and equal for all participants?** | | | | **Unclear** |
|  | *Yes:* | *Unclear:* | *No:* |  |
|  | *Adequate or validated methods of outcome measurement for all participants. Clinical reactions medically confirmed by a physician. Minimized risk of misclassification or differential assessment, reporting or detection.  RCT : Active harmful outcome surveillance (prospective/retrospective case-record review, questionnaires, patient’s diary/checklist…) .* | *There is no or not sufficient information to clearly determine how information on harmful outcome is collected or the process of minimizing misclassification.* | *Substantial risk of misclassification of outcome or differential assessment, reporting or detection. Clinical reactions not medically confirmed. RCT : Passive harmful outcome surveillance (patient’s volunteer reporting).* |  |
| **D7. Are the number of harmful outcome and the number of patients with a harmful outcome reported in both treatment arms?** | | | | **no** |
|  | *Yes:* | *Unclear:* | *No:* |  |
|  | *Numbers are reported. It is possible to calculate the rates of harmful outcome.* | *Confusion between the number of harmful outcomes or the number of patients with a harmful outcome, or general statements such as “5% of patients developed a harmful outcome”.* | *Neither the number of harmful outcomes nor the number of patients with a harmful outcome is reported. Or numbers are combining both treatment arms.* |  |
| **D8. Is the time frequency of harmful outcome assessment during the follow-up period appropriate?** | | | | **Unclear** |
|  | *Yes:* | *Unclear:* | *No:* |  |
|  | *For all study groups, the time frequency at which the harmful outcome is assessed is appropriate.* | *General statements such as “patients were routinely assessed for harmful outcomes”.* | *There is no regular collection of data on harmful outcomes during the study.* |  |
| **D9. Was the time between the exposure to a drug and the onset of the harmful outcome reported?** | | | | **No** |
|  | *Yes:* | *Unclear:* | *No:* |  |
|  | *The time between the drug exposure to the onset of harmful outcome is specified.* | *The authors do not report a clear time frame between drug exposure and harmful outcome.* | *The authors do not report the time between the drug exposure to the onset of harmful outcome.* |  |
| **D10. Was the process of determining that the harmful outcome is linked to the drug appropriate? Was the process blinded to the assigned treatment?** | | | | **Unclear** |
|  | *Yes:* | *Unclear:* | *No:* |  |
|  | *Methods for causality assessment are appropriate and, if applicable, made by investigators blinded to the intervention.* | *Unclear how the causality attribution is made. It is not clear who make the assessment or whether it is blinded to the assigned treatment.* | *Causality assessment is made by investigators not blinded to the intervention, or by participants or sponsors, or unblinding of treatment assignment precedes the decision to withdraw.* |  |
| **D. RISK OF BIAS ASSESSMENT FOR INFORMATION BIAS REGARDING THE HARMFULL OUTCOME** | | | | **Unclear** |
|  | *Low:* | *Unclear:* | *High:* |  |
|  | *Plausible bias unlikely to seriously alter the results.* | *Plausible bias that raises some doubts about the results or when information on which to base risk of bias judgments is missing or poorly reported.* | *Plausible bias that seriously weakens confidence in the results.* |  |
| **Comments: Unclear risk of bias** | | | | |
| **E. OTHER INFORMATION BIAS** | | | | |
| **E1. Is blinding of care givers during follow-up adequately performed in order to avoid differential care between study groups (performance bias)?** | | | | **Unclear** |
|  | *Yes:* | *Unclear:* | *No:* |  |
|  | *There is no risk of differential care or it is adequately addressed.* | *Unclear risk of bias due to differential care.* | *The bias due to differential care is not controlled.* |  |
| **E5. Does the study appear free of other information bias ?** | | | | **Unclear** |
|  | *Yes:* | *Unclear:* | *No:* |  |
|  | *The study appears to be free of other information bias.* | *Unclear presence of other information bias.* | *Additional source of other information bias.* |  |
| **E. RISK OF BIAS ASSESSMENT FOR OTHER INFORMATION BIAS** | | | | **Unclear** |
|  | *Low:* | *Unclear:* | *High:* |  |
|  | *Plausible bias unlikely to seriously alter the results.* | *Plausible bias that raises some doubts about the results or when information on which to base risk of bias judgments is missing or poorly reported.* | *Plausible bias that seriously weakens confidence in the results.* |  |
| **Comments: Unclear of bias -> open label** | | | | |
|  |  |  |  |  |
| **F. STATISTICAL METHODS TO CONTROL CONFOUNDING** | | | | |
| **F5. Does the study adequately address residual or unmeasured confounding?** | | | | **Yes** |
|  | *Yes:* | *Unclear:* | *No:* |  |
|  | *The study adequately addresses residual or unmeasured confounding.* | *Unclear presence of residual or unmeasured confounding.* | *Residual or unmeasured confounding is likely to be important.* |  |
| **F. RISK OF BIAS ASSESSMENT FOR STATISTICAL METHODS TO CONTROL CONFOUNDING:** | | | | **Low** |
|  | *Low:* | *Unclear:* | *High:* |  |
|  | *Plausible bias unlikely to seriously alter the results.* | *Plausible bias that raises some doubts about the results or when information on which to base risk of bias judgments is missing or poorly reported.* | *Plausible bias that seriously weakens confidence in the results.* |  |
| **Comments: Low risk of bias->Statistical Analysis section is well informative (stratification by to the time from the diagnosis of smoldering multiple myeloma to study enrollment (≤6 months vs. >6 months).** | | | | |
|  |  |  |  |  |
| **G. STATISTICAL METHODS EXCLUDING METHODS TO CONTROL CONFOUNDING** | | | | |
| **G1. Are the statistical methods used to analyze the harmful outcome appropriate?** | | | | **Unclear** |
|  | *Yes:* | *Unclear:* | *No:* |  |
|  | *Statistical techniques are appropriate to the data. If the distribution of the data (normal or not) is not described, it must be assumed that the estimates used were appropriate.* | *There is no description of the statistical techniques used, or the description is vague and not understandable.* | *The statistical techniques used are not appropriate.* |  |
| **G2. Is a survival analysis performed when there are individual differences in length of follow-up?** | | | | **Yes** |
|  | *Yes:* | *Unclear:* | *No:* |  |
|  | *Follow-up is the same for all study patients, if not survival analysis is performed.* | *Unclear whether there are different lengths of follow-up or whether they are taken into account.* | *Differences of follow up were ignored.* |  |
| **G3. If applicable, is composite outcome of harms adequately constructed?** | | | | **N/A** |
| *N/A* | *Yes:* | *Unclear:* | *No:* |  |
|  | *Composite outcome appropriate.* | *Unclear whether composite outcome is appropriate.* | *Construction of composite not described or not appropriate.* |  |
| **G6.** **Are the results consistent in primary and secondary analyses? Are confounding effects consistent with known associations?** | | | | **Yes** |
|  | *Yes:* | *Unclear:* | *No:* |  |
|  | *Consistency of primary, secondary analyses and consistency of confounding effects with known associations.* | *Not sufficient information to determine consistency.* | *No consistency of primary, secondary analyses or no consistency of confounding effects with known associations.* |  |
| **G. RISK OF BIAS ASSESSMENT FOR STATISTICAL METHODS EXCLUDING METHODS TO CONTROL CONFOUNDING:** | | | | **Unclear** |
|  | *Low:* | *Unclear:* | *High:* |  |
|  | *Plausible bias unlikely to seriously alter the results.* | *Plausible bias that raises some doubts about the results or when information on which to base risk of bias judgments is missing or poorly reported.* | *Plausible bias that seriously weakens confidence in the results.* |  |
| **Comments: Unclear risk of bias** | | | | |
|  | | | |  |
| **H. CONFLICT OF INTEREST** | | | | |
| **H1. Were the conflict of interest or sources of funding clearly acknowledged?** | | | | **Yes** |
|  | *Yes:* | *No:* |  |  |
|  | *Potential sources of support are acknowledged.* | *No sources of funding reported or not sufficient information.* |  |  |
| **H2. Does the study appear free of conflicts of interest susceptible to have influenced design, analysis or reporting (selective reporting of outcome or analysis)?** | | | | **Yes** |
|  | *Yes:* | *Unclear:* | *No:* |  |
|  | *No conflicts of interest or not susceptible to have influenced design, analysis or reporting.* | *It is unclear if there are conflicts of interest or if they are susceptible to have influenced design, analysis or reporting.* | *Conflicts of interest susceptible to have influenced design, analysis or reporting.* |  |
| **H. RISK OF BIAS ASSESSMENT FOR CONFLICT OF INTEREST** | | | | **Low** |
|  | *Low:* | *Unclear:* | *High:* |  |
|  | *Plausible bias unlikely to seriously alter the results.* | *Plausible bias that raises some doubts about the results or when information on which to base risk of bias judgments is missing or poorly reported.* | *Plausible bias that seriously weakens confidence in the results.* |  |
|  |  |  |  |  |
| **SUMMARY RISK-OF-BIAS ASSESSMENT FOR THE STUDY** | | | | |
| **RISK OF BIAS ASSESSMENT FOR THE STUDY** | | | | **Unclear** |
|  | *Low:* | *Unclear:* | *High:* |  |
|  | *Low risk of bias for all key domains.* | *Unclear risk of bias for one or more key domain.* | *High risk of bias for one or more key domains.* |  |

| **RISK OF BIAS ASSESSMENT CHECKLIST  FOR STUDIES INCLUDED IN SYSTEMATIC REVIEWS OF DRUG HARMS** | | | | |
| --- | --- | --- | --- | --- |
|  |  |  |  |  |
|  | **Study ID - Author** | McCarthy et al (2017), CALGB 100104, NCT00114101 |  |  |
|  |  |  |  |  |
| **A. STUDY DESIGN AND OBJECTIVES** | | | | |
| **A1. Are study objectives clearly specified and appropriate?** | | | | **Yes** |
|  | *Yes:* | *No:* |  |  |
|  | *Study objectives clearly specified and appropriate.* | *Study objectives are not clearly specified or not appropriate.* |  |  |
| **A2. Is study design clearly specified and appropriate?** | | | | **Yes** |
|  | *Yes:* | *No:* |  |  |
|  | *Study design clearly specified and appropriate.* | *Study design not clearly specified or not appropriate.* |  |  |
| **A3. Is the study design free of run-in/lead-in period before inclusion/randomization of participants?** | | | | **Yes** |
|  | *Yes:* | *Unclear:* | *No:* |  |
|  | *No run-in/lead-in period.* | *Not clear information.* | *Presence of a run-in/lead-in period.* |  |
| **A4. Cross-over designs: Is the study designed to adequately address carry-over effect?** | | | | **Yes** |
| *N/A* | *Yes:* | *Unclear:* | *No:* |  |
|  | *Carry-over effect absent or adequately addressed (randomized order and sufficiently long wash-out period.* | *Not clear information.* | *Carry-over effect not adequately addressed and susceptible to bias the results.* |  |
| **A. RISK OF BIAS ASSESSMENT FOR STUDY DESIGN AND OBJECTIVES** | | | | **Low** |
|  | *Low:* | *Unclear:* | *High:* |  |
|  | *Plausible bias unlikely to seriously alter the results.* | *Plausible bias that raises some doubts about the results or when information on which to base risk of bias judgments is missing or poorly reported.* | *Plausible bias that seriously weakens confidence in the results.* |  |
| **Comments: Low risk of bias according to data provided by sources** | | | | |
|  |  |  |  |  |
| **B. BIAS IN SELECTION OF SUBJECTS AND CONSTITUTION OF STUDY GROUPS** | | | | |
| **B1. Was the method used to generate the allocation sequence adequate as to produce comparable groups?** | | | | **Yes** |
|  | *Yes:* | *Unclear:* | *No:* |  |
|  | *Allocation methods are adequate to produce comparable groups.* | *Allocation methods are not clearly reported.* | *Allocation methods are not adequate (e.g. assignment to treatment by birth date, week day, etc.), groups are not comparable.* |  |
| **B2. Was the method used to conceal the allocation sequence adequate as to produce comparable groups?** | | | | **Yes** |
|  | *Yes:* | *Unclear:* | *No:* |  |
|  | *Concealment is adequate.* | *Concealment methods are not clearly reported and groups may not be comparable.* | *Concealment methods are not adequate, groups are not comparable.* |  |
| **B3. Are all the subjects recruited from the same source population?** | | | | **Yes** |
|  | *Yes:* | *Unclear:* | *No:* |  |
|  | *All the subjects recruited from the same source population.* | *Unclear if all the subjects recruited from the same source population.* | *All the subjects are not recruited from the same source population.* |  |
| **B4. Were inclusion and exclusion criteria implemented uniformly across study groups?** | | | | **Yes** |
|  | *Yes:* | *Unclear:* | *No:* |  |
|  | *Selection criteria uniformly implemented.* | *Unclear if selection criteria are uniformly implemented.* | *Selection criteria not uniformly implemented.* |  |
| **B8. Are baseline characteristics and prognostic factors comparable between different groups?** | | | | **Yes** |
|  | *Yes:* | *Unclear:* | *No:* |  |
|  | *RCT: Groups are comparable at baseline.* | *No description of baseline characteristics or only significance tests.* | *The groups are unbalanced at baseline.* |  |
|  | *Cohort studies: Groups are comparable at baseline or matched for the main prognostic factors.* |  |  |  |
| **B. RISK OF BIAS ASSESSMENT FOR SELECTION OF PARTICIPANTS AND CONSTITUTION OF STUDY GROUPS** | | | | **Low** |
|  | *Low:* | *Unclear:* | *High:* |  |
|  | *Plausible bias unlikely to seriously alter the results.* | *Plausible bias that raises some doubts about the results or when information on which to base risk of bias judgments is missing or poorly reported.* | *Plausible bias that seriously weakens confidence in the results.* |  |
| **Comments: low risk of bias according to data provided by sources** | | | | |
|  |  |  |  |  |
| **C. BIAS DUE TO WITHDRAWALS OR LOSS OF FOLLOW-UP (ATTRITION)** | | | | |
| **C1. Are the number of participants clearly reported throughout the study?** | | | | **Yes** |
|  | *Yes:* | *No:* |  |  |
|  | *Numbers of participants throughout the study are reported. Complete flow chart.* | *Numbers of patients at every stage is not clearly reported. Confusing information is reported regarding the number of participants. No or incomplete flow chart.* |  |  |
| **C2. Is the number of drop-outs/withdrawals due to harmful outcome clearly stated for each treatment arm?** | | | | **Yes** |
|  | *Yes:* | *No:* |  |  |
|  | *The number of drop-outs due to harmful outcome is specified.* | *The number of drop-outs due to harmful outcome is not specified, unclear or combined.* |  |  |
| **C3. Does the study adequately address biased loss to follow-up?** | | | | **Yes** |
|  | *Yes:* | *Unclear:* | *No:* |  |
|  | *Complete follow-up or drop-outs unlikely to introduce bias or adequately controlled.* | *Drop-outs/withdraws due to harmful outcome are not clearly reported.* | *Loss to follow-up affects the safety outcome and is not adequately controlled.* |  |
| **C4. Are the results based on an intention-to-treat analysis?** | | | | **Yes** |
|  | *Yes:* | *Unclear:* | *No:* |  |
|  | *Results are based on a strict intention-to-treat analysis.* | *Not clear if an intention-to-treat analysis is performed. No strict intention-to-treat analysis.* | *Results are not based on intention-to-treat analysis (not done or not possible).* |  |
| **C. RISK OF BIAS DUE TO WITHDRAWALS OR LOSS OF FOLLOW-UP (ATTRITION)** | | | | **Yes** |
|  | *Low:* | *Unclear:* | *High:* |  |
|  | *Plausible bias unlikely to seriously alter the results.* | *Plausible bias that raises some doubts about the results or when information on which to base risk of bias judgments is missing or poorly reported.* | *Plausible bias that seriously weakens confidence in the results.* |  |
| **Comments: Low risk of bias according to data provided by sources** | | | | |
|  |  |  |  |  |
| **D. INFORMATION BIAS REGARDING THE HARMFULL OUTCOME** | | | | |
| **D1. Is the definition of the harmful outcome clearly stated?** | | | | **Yes** |
|  | *Yes:* | *No:* |  |  |
|  | *RCT: clear / standardized definition of the harmful outcome (e.g. diagnostic codes, clinical and laboratory data). Cohort studies: clear definition of the outcome. Case-control studies: clear definition of cases.* | *Definition of the harmful outcome not reported or that leads to confusion. Terms not well-constructed, wrong definition.* |  |  |
| **D2. If applicable, is the severity of the harmful outcome clearly stated?** | | | | **Yes** |
|  | *N/A:* | *Yes:* | *No:* |  |
|  | *Self evident severity (e.g. death).* | *Detailed degree of severity or reference to a known scale of severity or a new scale developed for the study.* | *Unclear degrees of severity or without clear boundaries between them.* |  |
| **D3. Was the blinding methods of participants regarding the intervention appropriate considering the nature of the harmful outcome?** | | | | **Yes** |
|  | *Yes:* | *Unclear:* | *No:* |  |
|  | *Blinding ensured (and unlikely broken) or outcome not likely to be influenced by lack of blinding.* | *There is no sufficient information regarding the process of blinding or the outcome assessment.* | *No blinding (or incomplete blinding or risk of broken blinding) and outcome likely to be influenced by lack of blinding.* |  |
| **D4. Was the blinding methods of harmful outcome assessment appropriate considering the nature of the harmful outcome?** | | | | **Unclear** |
|  | *Yes:* | *Unclear:* | *No:* |  |
|  | *Blinding ensured (and unlikely broken) or outcome assessment not likely to be influenced by lack of blinding.* | *There is no sufficient information regarding the process of blinding of outcome assessment.* | *No blinding (or incomplete blinding or risk of broken blinding) and outcome likely to be influenced by lack of blinding.* |  |
| **D5. Was the duration of follow-up adequate to assess the harmful outcome?** | | | | **Unclear** |
|  | *Yes:* | *Unclear:* | *No:* |  |
|  | *Sufficient duration of follow-up to assess the outcome.* | *It is unclear whether the duration of follow-up is adequate.* | *Too short duration of follow-up.* |  |
| **D6. Was the methods for ascertaining the harmful outcome adequately constructed and equal for all participants?** | | | | **Unclear** |
|  | *Yes:* | *Unclear:* | *No:* |  |
|  | *Adequate or validated methods of outcome measurement for all participants. Clinical reactions medically confirmed by a physician. Minimized risk of misclassification or differential assessment, reporting or detection.  RCT : Active harmful outcome surveillance (prospective/retrospective case-record review, questionnaires, patient’s diary/checklist…) .* | *There is no or not sufficient information to clearly determine how information on harmful outcome is collected or the process of minimizing misclassification.* | *Substantial risk of misclassification of outcome or differential assessment, reporting or detection. Clinical reactions not medically confirmed. RCT : Passive harmful outcome surveillance (patient’s volunteer reporting).* |  |
| **D7. Are the number of harmful outcome and the number of patients with a harmful outcome reported in both treatment arms?** | | | | **Yes** |
|  | *Yes:* | *Unclear:* | *No:* |  |
|  | *Numbers are reported. It is possible to calculate the rates of harmful outcome.* | *Confusion between the number of harmful outcomes or the number of patients with a harmful outcome, or general statements such as “5% of patients developed a harmful outcome”.* | *Neither the number of harmful outcomes nor the number of patients with a harmful outcome is reported. Or numbers are combining both treatment arms.* |  |
| **D8. Is the time frequency of harmful outcome assessment during the follow-up period appropriate?** | | | | **Yes** |
|  | *Yes:* | *Unclear:* | *No:* |  |
|  | *For all study groups, the time frequency at which the harmful outcome is assessed is appropriate.* | *General statements such as “patients were routinely assessed for harmful outcomes”.* | *There is no regular collection of data on harmful outcomes during the study.* |  |
| **D9. Was the time between the exposure to a drug and the onset of the harmful outcome reported?** | | | | **Yes** |
|  | *Yes:* | *Unclear:* | *No:* |  |
|  | *The time between the drug exposure to the onset of harmful outcome is specified.* | *The authors do not report a clear time frame between drug exposure and harmful outcome.* | *The authors do not report the time between the drug exposure to the onset of harmful outcome.* |  |
| **D10. Was the process of determining that the harmful outcome is linked to the drug appropriate? Was the process blinded to the assigned treatment?** | | | | **Unclear** |
|  | *Yes:* | *Unclear:* | *No:* |  |
|  | *Methods for causality assessment are appropriate and, if applicable, made by investigators blinded to the intervention.* | *Unclear how the causality attribution is made. It is not clear who make the assessment or whether it is blinded to the assigned treatment.* | *Causality assessment is made by investigators not blinded to the intervention, or by participants or sponsors, or unblinding of treatment assignment precedes the decision to withdraw.* |  |
| **D. RISK OF BIAS ASSESSMENT FOR INFORMATION BIAS REGARDING THE HARMFULL OUTCOME** | | | | **Unclear** |
|  | *Low:* | *Unclear:* | *High:* |  |
|  | *Plausible bias unlikely to seriously alter the results.* | *Plausible bias that raises some doubts about the results or when information on which to base risk of bias judgments is missing or poorly reported.* | *Plausible bias that seriously weakens confidence in the results.* |  |
| **Comments: Unclear risk of bias** | | | | |
|  |  |  |  |  |
| **E. OTHER INFORMATION BIAS** | | | | |
| **E1. Is blinding of care givers during follow-up adequately performed in order to avoid differential care between study groups (performance bias)?** | | | | **Yes** |
|  | *Yes:* | *Unclear:* | *No:* |  |
|  | *There is no risk of differential care or it is adequately addressed.* | *Unclear risk of bias due to differential care.* | *The bias due to differential care is not controlled.* |  |
| **E5. Does the study appear free of other information bias ?** | | | | **Yes** |
|  | *Yes:* | *Unclear:* | *No:* |  |
|  | *The study appears to be free of other information bias.* | *Unclear presence of other information bias.* | *Additional source of other information bias.* |  |
| **E. RISK OF BIAS ASSESSMENT FOR OTHER INFORMATION BIAS** | | | | **Low** |
|  | *Low:* | *Unclear:* | *High:* |  |
|  | *Plausible bias unlikely to seriously alter the results.* | *Plausible bias that raises some doubts about the results or when information on which to base risk of bias judgments is missing or poorly reported.* | *Plausible bias that seriously weakens confidence in the results.* |  |
| **Comments: Low of bias -> masking methods informations were well informative (pivotal +supp data).** | | | | |
|  |  |  |  |  |
| **F. STATISTICAL METHODS TO CONTROL CONFOUNDING** | | | | |
| **F5. Does the study adequately address residual or unmeasured confounding?** | | | | **Yes** |
|  | *Yes:* | *Unclear:* | *No:* |  |
|  | *The study adequately addresses residual or unmeasured confounding.* | *Unclear presence of residual or unmeasured confounding.* | *Residual or unmeasured confounding is likely to be important.* |  |
| **F. RISK OF BIAS ASSESSMENT FOR STATISTICAL METHODS TO CONTROL CONFOUNDING:** | | | | **Low** |
|  | *Low:* | *Unclear:* | *High:* |  |
|  | *Plausible bias unlikely to seriously alter the results.* | *Plausible bias that raises some doubts about the results or when information on which to base risk of bias judgments is missing or poorly reported.* | *Plausible bias that seriously weakens confidence in the results.* |  |
| **Comments: Low risk of bias->Statistical Analysis section is well informative (stratification by normal or elevated serum β2-microglobulin level at registration (≤2.5 mg per liter vs. >2.5 mg per liter [≤211.9 nmol per liter vs. >211.9 nmol per liter]), prior use or nonuse of thalidomide during induction therapy; and prior use or nonuse of lenalidomide during induction therapy).** | | | | |
|  |  |  |  |  |
| **G. STATISTICAL METHODS EXCLUDING METHODS TO CONTROL CONFOUNDING** | | | | |
| **G1. Are the statistical methods used to analyze the harmful outcome appropriate?** | | | | **Unclear** |
|  | *Yes:* | *Unclear:* | *No:* |  |
|  | *Statistical techniques are appropriate to the data. If the distribution of the data (normal or not) is not described, it must be assumed that the estimates used were appropriate.* | *There is no description of the statistical techniques used, or the description is vague and not understandable.* | *The statistical techniques used are not appropriate.* |  |
| **G2. Is a survival analysis performed when there are individual differences in length of follow-up?** | | | | **Yes** |
|  | *Yes:* | *Unclear:* | *No:* |  |
|  | *Follow-up is the same for all study patients, if not survival analysis is performed.* | *Unclear whether there are different lengths of follow-up or whether they are taken into account.* | *Differences of follow up were ignored.* |  |
| **G3. If applicable, is composite outcome of harms adequately constructed?** | | | | **Unclear** |
| *N/A* | *Yes:* | *Unclear:* | *No:* |  |
|  | *Composite outcome appropriate.* | *Unclear whether composite outcome is appropriate.* | *Construction of composite not described or not appropriate.* |  |
| **G6.** **Are the results consistent in primary and secondary analyses? Are confounding effects consistent with known associations?** | | | | **Yes** |
|  | *Yes:* | *Unclear:* | *No:* |  |
|  | *Consistency of primary, secondary analyses and consistency of confounding effects with known associations.* | *Not sufficient information to determine consistency.* | *No consistency of primary, secondary analyses or no consistency of confounding effects with known associations.* |  |
| **G. RISK OF BIAS ASSESSMENT FOR STATISTICAL METHODS EXCLUDING METHODS TO CONTROL CONFOUNDING:** | | | | **Unclear** |
|  | *Low:* | *Unclear:* | *High:* |  |
|  | *Plausible bias unlikely to seriously alter the results.* | *Plausible bias that raises some doubts about the results or when information on which to base risk of bias judgments is missing or poorly reported.* | *Plausible bias that seriously weakens confidence in the results.* |  |
| **Comments: Unclear risk of bias** | | | | |
|  | | | |  |
| **H. CONFLICT OF INTEREST** | | | | |
| **H1. Were the conflict of interest or sources of funding clearly acknowledged?** | | | | **Yes** |
|  | *Yes:* | *No:* |  |  |
|  | *Potential sources of support are acknowledged.* | *No sources of funding reported or not sufficient information.* |  |  |
| **H2. Does the study appear free of conflicts of interest susceptible to have influenced design, analysis or reporting (selective reporting of outcome or analysis)?** | | | | **Yes** |
|  | *Yes:* | *Unclear:* | *No:* |  |
|  | *No conflicts of interest or not susceptible to have influenced design, analysis or reporting.* | *It is unclear if there are conflicts of interest or if they are susceptible to have influenced design, analysis or reporting.* | *Conflicts of interest susceptible to have influenced design, analysis or reporting.* |  |
| **H. RISK OF BIAS ASSESSMENT FOR CONFLICT OF INTEREST** | | | | **Low** |
|  | *Low:* | *Unclear:* | *High:* |  |
|  | *Plausible bias unlikely to seriously alter the results.* | *Plausible bias that raises some doubts about the results or when information on which to base risk of bias judgments is missing or poorly reported.* | *Plausible bias that seriously weakens confidence in the results.* |  |
|  |  |  |  |  |
| **SUMMARY RISK-OF-BIAS ASSESSMENT FOR THE STUDY** | | | | |
| **RISK OF BIAS ASSESSMENT FOR THE STUDY** | | | | **Unclear** |
|  | *Low:* | *Unclear:* | *High:* |  |
|  | *Low risk of bias for all key domains.* | *Unclear risk of bias for one or more key domain.* | *High risk of bias for one or more key domains.* |  |

| **RISK OF BIAS ASSESSMENT CHECKLIST  FOR STUDIES INCLUDED IN SYSTEMATIC REVIEWS OF DRUG HARMS** | | | | |
| --- | --- | --- | --- | --- |
|  |  |  |  |  |
|  | **Study ID - Author** | Palumbo et al (2012), CC-5013-MM-015, NCT00405756 |  |  |
|  |  |  |  |  |
| **A. STUDY DESIGN AND OBJECTIVES** | | | | |
| **A1. Are study objectives clearly specified and appropriate?** | | | | **Yes** |
|  | *Yes:* | *No:* |  |  |
|  | *Study objectives clearly specified and appropriate.* | *Study objectives are not clearly specified or not appropriate.* |  |  |
| **A2. Is study design clearly specified and appropriate?** | | | | **Yes** |
|  | *Yes:* | *No:* |  |  |
|  | *Study design clearly specified and appropriate.* | *Study design not clearly specified or not appropriate.* |  |  |
| **A3. Is the study design free of run-in/lead-in period before inclusion/randomization of participants?** | | | | **Yes** |
|  | *Yes:* | *Unclear:* | *No:* |  |
|  | *No run-in/lead-in period.* | *Not clear information.* | *Presence of a run-in/lead-in period.* |  |
| **A4. Cross-over designs: Is the study designed to adequately address carry-over effect?** | | | | **Yes** |
| *N/A* | *Yes:* | *Unclear:* | *No:* |  |
|  | *Carry-over effect absent or adequately addressed (randomized order and sufficiently long wash-out period.* | *Not clear information.* | *Carry-over effect not adequately addressed and susceptible to bias the results.* |  |
| **A. RISK OF BIAS ASSESSMENT FOR STUDY DESIGN AND OBJECTIVES** | | | | **Low** |
|  | *Low:* | *Unclear:* | *High:* |  |
|  | *Plausible bias unlikely to seriously alter the results.* | *Plausible bias that raises some doubts about the results or when information on which to base risk of bias judgments is missing or poorly reported.* | *Plausible bias that seriously weakens confidence in the results.* |  |
| **Comments: Low risk of bias according to data provided by sources** | | | | |
|  |  |  |  |  |
| **B. BIAS IN SELECTION OF SUBJECTS AND CONSTITUTION OF STUDY GROUPS** | | | | |
| **B1. Was the method used to generate the allocation sequence adequate as to produce comparable groups?** | | | | **Unclear** |
|  | *Yes:* | *Unclear:* | *No:* |  |
|  | *Allocation methods are adequate to produce comparable groups.* | *Allocation methods are not clearly reported.* | *Allocation methods are not adequate (e.g. assignment to treatment by birth date, week day, etc.), groups are not comparable.* |  |
| **B2. Was the method used to conceal the allocation sequence adequate as to produce comparable groups?** | | | | **Yes** |
|  | *Yes:* | *Unclear:* | *No:* |  |
|  | *Concealment is adequate.* | *Concealment methods are not clearly reported and groups may not be comparable.* | *Concealment methods are not adequate, groups are not comparable.* |  |
| **B3. Are all the subjects recruited from the same source population?** | | | | **Yes** |
|  | *Yes:* | *Unclear:* | *No:* |  |
|  | *All the subjects recruited from the same source population.* | *Unclear if all the subjects recruited from the same source population.* | *All the subjects are not recruited from the same source population.* |  |
| **B4. Were inclusion and exclusion criteria implemented uniformly across study groups?** | | | | **Yes** |
|  | *Yes:* | *Unclear:* | *No:* |  |
|  | *Selection criteria uniformly implemented.* | *Unclear if selection criteria are uniformly implemented.* | *Selection criteria not uniformly implemented.* |  |
| **B8. Are baseline characteristics and prognostic factors comparable between different groups?** | | | | **Yes** |
|  | *Yes:* | *Unclear:* | *No:* |  |
|  | *RCT: Groups are comparable at baseline.* | *No description of baseline characteristics or only significance tests.* | *The groups are unbalanced at baseline.* |  |
|  | *Cohort studies: Groups are comparable at baseline or matched for the main prognostic factors.* |  |  |  |
| **B. RISK OF BIAS ASSESSMENT FOR SELECTION OF PARTICIPANTS AND CONSTITUTION OF STUDY GROUPS** | | | | **Unclear** |
|  | *Low:* | *Unclear:* | *High:* |  |
|  | *Plausible bias unlikely to seriously alter the results.* | *Plausible bias that raises some doubts about the results or when information on which to base risk of bias judgments is missing or poorly reported.* | *Plausible bias that seriously weakens confidence in the results.* |  |
| **Comments: unclear risk of bias according to data provided by sources** | | | | |
|  |  |  |  |  |
| **C. BIAS DUE TO WITHDRAWALS OR LOSS OF FOLLOW-UP (ATTRITION)** | | | | |
| **C1. Are the number of participants clearly reported throughout the study?** | | | | **Yes** |
|  | *Yes:* | *No:* |  |  |
|  | *Numbers of participants throughout the study are reported. Complete flow chart.* | *Numbers of patients at every stage is not clearly reported. Confusing information is reported regarding the number of participants. No or incomplete flow chart.* |  |  |
| **C2. Is the number of drop-outs/withdrawals due to harmful outcome clearly stated for each treatment arm?** | | | | **yes** |
|  | *Yes:* | *No:* |  |  |
|  | *The number of drop-outs due to harmful outcome is specified.* | *The number of drop-outs due to harmful outcome is not specified, unclear or combined.* |  |  |
| **C3. Does the study adequately address biased loss to follow-up?** | | | | **yes** |
|  | *Yes:* | *Unclear:* | *No:* |  |
|  | *Complete follow-up or drop-outs unlikely to introduce bias or adequately controlled.* | *Drop-outs/withdraws due to harmful outcome are not clearly reported.* | *Loss to follow-up affects the safety outcome and is not adequately controlled.* |  |
| **C4. Are the results based on an intention-to-treat analysis?** | | | | **yes** |
|  | *Yes:* | *Unclear:* | *No:* |  |
|  | *Results are based on a strict intention-to-treat analysis.* | *Not clear if an intention-to-treat analysis is performed. No strict intention-to-treat analysis.* | *Results are not based on intention-to-treat analysis (not done or not possible).* |  |
| **C. RISK OF BIAS DUE TO WITHDRAWALS OR LOSS OF FOLLOW-UP (ATTRITION)** | | | | **Low** |
|  | *Low:* | *Unclear:* | *High:* |  |
|  | *Plausible bias unlikely to seriously alter the results.* | *Plausible bias that raises some doubts about the results or when information on which to base risk of bias judgments is missing or poorly reported.* | *Plausible bias that seriously weakens confidence in the results.* |  |
| **Comments: Low risk of bias according to data provided by sources** | | | | |
|  |  |  |  |  |
| **D. INFORMATION BIAS REGARDING THE HARMFULL OUTCOME** | | | | |
| **D1. Is the definition of the harmful outcome clearly stated?** | | | | **Yes** |
|  | *Yes:* | *No:* |  |  |
|  | *RCT: clear / standardized definition of the harmful outcome (e.g. diagnostic codes, clinical and laboratory data). Cohort studies: clear definition of the outcome. Case-control studies: clear definition of cases.* | *Definition of the harmful outcome not reported or that leads to confusion. Terms not well-constructed, wrong definition.* |  |  |
| **D2. If applicable, is the severity of the harmful outcome clearly stated?** | | | | **Yes** |
|  | *N/A:* | *Yes:* | *No:* |  |
|  | *Self evident severity (e.g. death).* | *Detailed degree of severity or reference to a known scale of severity or a new scale developed for the study.* | *Unclear degrees of severity or without clear boundaries between them.* |  |
| **D3. Was the blinding methods of participants regarding the intervention appropriate considering the nature of the harmful outcome?** | | | | **Yes** |
|  | *Yes:* | *Unclear:* | *No:* |  |
|  | *Blinding ensured (and unlikely broken) or outcome not likely to be influenced by lack of blinding.* | *There is no sufficient information regarding the process of blinding or the outcome assessment.* | *No blinding (or incomplete blinding or risk of broken blinding) and outcome likely to be influenced by lack of blinding.* |  |
| **D4. Was the blinding methods of harmful outcome assessment appropriate considering the nature of the harmful outcome?** | | | | **Unclear** |
|  | *Yes:* | *Unclear:* | *No:* |  |
|  | *Blinding ensured (and unlikely broken) or outcome assessment not likely to be influenced by lack of blinding.* | *There is no sufficient information regarding the process of blinding of outcome assessment.* | *No blinding (or incomplete blinding or risk of broken blinding) and outcome likely to be influenced by lack of blinding.* |  |
| **D5. Was the duration of follow-up adequate to assess the harmful outcome?** | | | | **Unclear** |
|  | *Yes:* | *Unclear:* | *No:* |  |
|  | *Sufficient duration of follow-up to assess the outcome.* | *It is unclear whether the duration of follow-up is adequate.* | *Too short duration of follow-up.* |  |
| **D6. Was the methods for ascertaining the harmful outcome adequately constructed and equal for all participants?** | | | | **Unclear** |
|  | *Yes:* | *Unclear:* | *No:* |  |
|  | *Adequate or validated methods of outcome measurement for all participants. Clinical reactions medically confirmed by a physician. Minimized risk of misclassification or differential assessment, reporting or detection.  RCT : Active harmful outcome surveillance (prospective/retrospective case-record review, questionnaires, patient’s diary/checklist…) .* | *There is no or not sufficient information to clearly determine how information on harmful outcome is collected or the process of minimizing misclassification.* | *Substantial risk of misclassification of outcome or differential assessment, reporting or detection. Clinical reactions not medically confirmed. RCT : Passive harmful outcome surveillance (patient’s volunteer reporting).* |  |
| **D7. Are the number of harmful outcome and the number of patients with a harmful outcome reported in both treatment arms?** | | | | **Yes** |
|  | *Yes:* | *Unclear:* | *No:* |  |
|  | *Numbers are reported. It is possible to calculate the rates of harmful outcome.* | *Confusion between the number of harmful outcomes or the number of patients with a harmful outcome, or general statements such as “5% of patients developed a harmful outcome”.* | *Neither the number of harmful outcomes nor the number of patients with a harmful outcome is reported. Or numbers are combining both treatment arms.* |  |
| **D8. Is the time frequency of harmful outcome assessment during the follow-up period appropriate?** | | | | **Unclear** |
|  | *Yes:* | *Unclear:* | *No:* |  |
|  | *For all study groups, the time frequency at which the harmful outcome is assessed is appropriate.* | *General statements such as “patients were routinely assessed for harmful outcomes”.* | *There is no regular collection of data on harmful outcomes during the study.* |  |
| **D9. Was the time between the exposure to a drug and the onset of the harmful outcome reported?** | | | | **Unclear** |
|  | *Yes:* | *Unclear:* | *No:* |  |
|  | *The time between the drug exposure to the onset of harmful outcome is specified.* | *The authors do not report a clear time frame between drug exposure and harmful outcome.* | *The authors do not report the time between the drug exposure to the onset of harmful outcome.* |  |
| **D10. Was the process of determining that the harmful outcome is linked to the drug appropriate? Was the process blinded to the assigned treatment?** | | | | **Unclear** |
|  | *Yes:* | *Unclear:* | *No:* |  |
|  | *Methods for causality assessment are appropriate and, if applicable, made by investigators blinded to the intervention.* | *Unclear how the causality attribution is made. It is not clear who make the assessment or whether it is blinded to the assigned treatment.* | *Causality assessment is made by investigators not blinded to the intervention, or by participants or sponsors, or unblinding of treatment assignment precedes the decision to withdraw.* |  |
| **D. RISK OF BIAS ASSESSMENT FOR INFORMATION BIAS REGARDING THE HARMFULL OUTCOME** | | | | **Unclear** |
|  | *Low:* | *Unclear:* | *High:* |  |
|  | *Plausible bias unlikely to seriously alter the results.* | *Plausible bias that raises some doubts about the results or when information on which to base risk of bias judgments is missing or poorly reported.* | *Plausible bias that seriously weakens confidence in the results.* |  |
| **Comments: Unclear risk of bias** | | | | |
|  |  |  |  |  |
| **E. OTHER INFORMATION BIAS** | | | | |
| **E1. Is blinding of care givers during follow-up adequately performed in order to avoid differential care between study groups (performance bias)?** | | | | **Yes** |
|  | *Yes:* | *Unclear:* | *No:* |  |
|  | *There is no risk of differential care or it is adequately addressed.* | *Unclear risk of bias due to differential care.* | *The bias due to differential care is not controlled.* |  |
| **E5. Does the study appear free of other information bias ?** | | | | **Yes** |
|  | *Yes:* | *Unclear:* | *No:* |  |
|  | *The study appears to be free of other information bias.* | *Unclear presence of other information bias.* | *Additional source of other information bias.* |  |
| **E. RISK OF BIAS ASSESSMENT FOR OTHER INFORMATION BIAS** | | | | **Low** |
|  | *Low:* | *Unclear:* | *High:* |  |
|  | *Plausible bias unlikely to seriously alter the results.* | *Plausible bias that raises some doubts about the results or when information on which to base risk of bias judgments is missing or poorly reported.* | *Plausible bias that seriously weakens confidence in the results.* |  |
| **Comments: Low of bias -> masking methods informations were well informative (pivotal +supp data).** | | | | |
|  |  |  |  |  |
| **F. STATISTICAL METHODS TO CONTROL CONFOUNDING** | | | | |
| **F5. Does the study adequately address residual or unmeasured confounding?** | | | | **Yes** |
|  | *Yes:* | *Unclear:* | *No:* |  |
|  | *The study adequately addresses residual or unmeasured confounding.* | *Unclear presence of residual or unmeasured confounding.* | *Residual or unmeasured confounding is likely to be important.* |  |
| **F. RISK OF BIAS ASSESSMENT FOR STATISTICAL METHODS TO CONTROL CONFOUNDING:** | | | | **Low** |
|  | *Low:* | *Unclear:* | *High:* |  |
|  | *Plausible bias unlikely to seriously alter the results.* | *Plausible bias that raises some doubts about the results or when information on which to base risk of bias judgments is missing or poorly reported.* | *Plausible bias that seriously weakens confidence in the results.* |  |
| **Comments: Low risk of bias->Statistical Analysis section is well informative (stratified by age (65 to 75 years vs. >75 years) and International Staging System stage (stage I or II vs. stage III, with higher stages indicating more severe disease).** | | | | |
|  |  |  |  |  |
| **G. STATISTICAL METHODS EXCLUDING METHODS TO CONTROL CONFOUNDING** | | | | |
| **G1. Are the statistical methods used to analyze the harmful outcome appropriate?** | | | | **Unclear** |
|  | *Yes:* | *Unclear:* | *No:* |  |
|  | *Statistical techniques are appropriate to the data. If the distribution of the data (normal or not) is not described, it must be assumed that the estimates used were appropriate.* | *There is no description of the statistical techniques used, or the description is vague and not understandable.* | *The statistical techniques used are not appropriate.* |  |
| **G2. Is a survival analysis performed when there are individual differences in length of follow-up?** | | | | **Yes** |
|  | *Yes:* | *Unclear:* | *No:* |  |
|  | *Follow-up is the same for all study patients, if not survival analysis is performed.* | *Unclear whether there are different lengths of follow-up or whether they are taken into account.* | *Differences of follow up were ignored.* |  |
| **G3. If applicable, is composite outcome of harms adequately constructed?** | | | | **Unclear** |
| *N/A* | *Yes:* | *Unclear:* | *No:* |  |
|  | *Composite outcome appropriate.* | *Unclear whether composite outcome is appropriate.* | *Construction of composite not described or not appropriate.* |  |
| **G6.** **Are the results consistent in primary and secondary analyses? Are confounding effects consistent with known associations?** | | | | **Yes** |
|  | *Yes:* | *Unclear:* | *No:* |  |
|  | *Consistency of primary, secondary analyses and consistency of confounding effects with known associations.* | *Not sufficient information to determine consistency.* | *No consistency of primary, secondary analyses or no consistency of confounding effects with known associations.* |  |
| **G. RISK OF BIAS ASSESSMENT FOR STATISTICAL METHODS EXCLUDING METHODS TO CONTROL CONFOUNDING:** | | | | **Unclear** |
|  | *Low:* | *Unclear:* | *High:* |  |
|  | *Plausible bias unlikely to seriously alter the results.* | *Plausible bias that raises some doubts about the results or when information on which to base risk of bias judgments is missing or poorly reported.* | *Plausible bias that seriously weakens confidence in the results.* |  |
| **Comments: Unclear risk of bias** | | | | |
|  | | | |  |
| **H. CONFLICT OF INTEREST** | | | | |
| **H1. Were the conflict of interest or sources of funding clearly acknowledged?** | | | | **Yes** |
|  | *Yes:* | *No:* |  |  |
|  | *Potential sources of support are acknowledged.* | *No sources of funding reported or not sufficient information.* |  |  |
| **H2. Does the study appear free of conflicts of interest susceptible to have influenced design, analysis or reporting (selective reporting of outcome or analysis)?** | | | | **Yes** |
|  | *Yes:* | *Unclear:* | *No:* |  |
|  | *No conflicts of interest or not susceptible to have influenced design, analysis or reporting.* | *It is unclear if there are conflicts of interest or if they are susceptible to have influenced design, analysis or reporting.* | *Conflicts of interest susceptible to have influenced design, analysis or reporting.* |  |
| **H. RISK OF BIAS ASSESSMENT FOR CONFLICT OF INTEREST** | | | | **Low** |
|  | *Low:* | *Unclear:* | *High:* |  |
|  | *Plausible bias unlikely to seriously alter the results.* | *Plausible bias that raises some doubts about the results or when information on which to base risk of bias judgments is missing or poorly reported.* | *Plausible bias that seriously weakens confidence in the results.* |  |
|  |  |  |  |  |
| **SUMMARY RISK-OF-BIAS ASSESSMENT FOR THE STUDY** | | | | |
| **RISK OF BIAS ASSESSMENT FOR THE STUDY** | | | | **Unclear** |
|  | *Low:* | *Unclear:* | *High:* |  |
|  | *Low risk of bias for all key domains.* | *Unclear risk of bias for one or more key domain.* | *High risk of bias for one or more key domains.* |  |

| **RISK OF BIAS ASSESSMENT CHECKLIST  FOR STUDIES INCLUDED IN SYSTEMATIC REVIEWS OF DRUG HARMS** | | | | |
| --- | --- | --- | --- | --- |
|  |  |  |  |  |
|  | **Study ID - Author** | Palumbo et al (2014), RV-MM-PI-209/GIMEMA, NCT00551928 |  |  |
|  |  |  |  |  |
| **A. STUDY DESIGN AND OBJECTIVES** | | | | |
| **A1. Are study objectives clearly specified and appropriate?** | | | | **Yes** |
|  | *Yes:* | *No:* |  |  |
|  | *Study objectives clearly specified and appropriate.* | *Study objectives are not clearly specified or not appropriate.* |  |  |
| **A2. Is study design clearly specified and appropriate?** | | | | **Yes** |
|  | *Yes:* | *No:* |  |  |
|  | *Study design clearly specified and appropriate.* | *Study design not clearly specified or not appropriate.* |  |  |
| **A3. Is the study design free of run-in/lead-in period before inclusion/randomization of participants?** | | | | **Yes** |
|  | *Yes:* | *Unclear:* | *No:* |  |
|  | *No run-in/lead-in period.* | *Not clear information.* | *Presence of a run-in/lead-in period.* |  |
| **A4. Cross-over designs: Is the study designed to adequately address carry-over effect?** | | | | **N/A** |
| *N/A* | *Yes:* | *Unclear:* | *No:* |  |
|  | *Carry-over effect absent or adequately addressed (randomized order and sufficiently long wash-out period.* | *Not clear information.* | *Carry-over effect not adequately addressed and susceptible to bias the results.* |  |
| **A. RISK OF BIAS ASSESSMENT FOR STUDY DESIGN AND OBJECTIVES** | | | | **Low** |
|  | *Low:* | *Unclear:* | *High:* |  |
|  | *Plausible bias unlikely to seriously alter the results.* | *Plausible bias that raises some doubts about the results or when information on which to base risk of bias judgments is missing or poorly reported.* | *Plausible bias that seriously weakens confidence in the results.* |  |
| **Comments: Low risk of bias according to data provided by sources** | | | | |
|  |  |  |  |  |
| **B. BIAS IN SELECTION OF SUBJECTS AND CONSTITUTION OF STUDY GROUPS** | | | | |
| **B1. Was the method used to generate the allocation sequence adequate as to produce comparable groups?** | | | | **Yes** |
|  | *Yes:* | *Unclear:* | *No:* |  |
|  | *Allocation methods are adequate to produce comparable groups.* | *Allocation methods are not clearly reported.* | *Allocation methods are not adequate (e.g. assignment to treatment by birth date, week day, etc.), groups are not comparable.* |  |
| **B2. Was the method used to conceal the allocation sequence adequate as to produce comparable groups?** | | | | **Yes** |
|  | *Yes:* | *Unclear:* | *No:* |  |
|  | *Concealment is adequate.* | *Concealment methods are not clearly reported and groups may not be comparable.* | *Concealment methods are not adequate, groups are not comparable.* |  |
| **B3. Are all the subjects recruited from the same source population?** | | | | **Yes** |
|  | *Yes:* | *Unclear:* | *No:* |  |
|  | *All the subjects recruited from the same source population.* | *Unclear if all the subjects recruited from the same source population.* | *All the subjects are not recruited from the same source population.* |  |
| **B4. Were inclusion and exclusion criteria implemented uniformly across study groups?** | | | | **Yes** |
|  | *Yes:* | *Unclear:* | *No:* |  |
|  | *Selection criteria uniformly implemented.* | *Unclear if selection criteria are uniformly implemented.* | *Selection criteria not uniformly implemented.* |  |
| **B8. Are baseline characteristics and prognostic factors comparable between different groups?** | | | | **Yes** |
|  | *Yes:* | *Unclear:* | *No:* |  |
|  | *RCT: Groups are comparable at baseline.* | *No description of baseline characteristics or only significance tests.* | *The groups are unbalanced at baseline.* |  |
|  | *Cohort studies: Groups are comparable at baseline or matched for the main prognostic factors.* |  |  |  |
| **B. RISK OF BIAS ASSESSMENT FOR SELECTION OF PARTICIPANTS AND CONSTITUTION OF STUDY GROUPS** | | | | **Low** |
|  | *Low:* | *Unclear:* | *High:* |  |
|  | *Plausible bias unlikely to seriously alter the results.* | *Plausible bias that raises some doubts about the results or when information on which to base risk of bias judgments is missing or poorly reported.* | *Plausible bias that seriously weakens confidence in the results.* |  |
| **Comments: Low risk of bias according to data provided by sources** | | | | |
|  |  |  |  |  |
| **C. BIAS DUE TO WITHDRAWALS OR LOSS OF FOLLOW-UP (ATTRITION)** | | | | |
| **C1. Are the number of participants clearly reported throughout the study?** | | | | **Yes** |
|  | *Yes:* | *No:* |  |  |
|  | *Numbers of participants throughout the study are reported. Complete flow chart.* | *Numbers of patients at every stage is not clearly reported. Confusing information is reported regarding the number of participants. No or incomplete flow chart.* |  |  |
| **C2. Is the number of drop-outs/withdrawals due to harmful outcome clearly stated for each treatment arm?** | | | | **Yes** |
|  | *Yes:* | *No:* |  |  |
|  | *The number of drop-outs due to harmful outcome is specified.* | *The number of drop-outs due to harmful outcome is not specified, unclear or combined.* |  |  |
| **C3. Does the study adequately address biased loss to follow-up?** | | | | **Yes** |
|  | *Yes:* | *Unclear:* | *No:* |  |
|  | *Complete follow-up or drop-outs unlikely to introduce bias or adequately controlled.* | *Drop-outs/withdraws due to harmful outcome are not clearly reported.* | *Loss to follow-up affects the safety outcome and is not adequately controlled.* |  |
| **C4. Are the results based on an intention-to-treat analysis?** | | | | **Yes** |
|  | *Yes:* | *Unclear:* | *No:* |  |
|  | *Results are based on a strict intention-to-treat analysis.* | *Not clear if an intention-to-treat analysis is performed. No strict intention-to-treat analysis.* | *Results are not based on intention-to-treat analysis (not done or not possible).* |  |
| **C. RISK OF BIAS DUE TO WITHDRAWALS OR LOSS OF FOLLOW-UP (ATTRITION)** | | | | **Low** |
|  | *Low:* | *Unclear:* | *High:* |  |
|  | *Plausible bias unlikely to seriously alter the results.* | *Plausible bias that raises some doubts about the results or when information on which to base risk of bias judgments is missing or poorly reported.* | *Plausible bias that seriously weakens confidence in the results.* |  |
| **Comments: Low risk of bias according to data provided by sources** | | | | |
|  |  |  |  |  |
| **D. INFORMATION BIAS REGARDING THE HARMFULL OUTCOME** | | | | |
| **D1. Is the definition of the harmful outcome clearly stated?** | | | | **No** |
|  | *Yes:* | *No:* |  |  |
|  | *RCT: clear / standardized definition of the harmful outcome (e.g. diagnostic codes, clinical and laboratory data). Cohort studies: clear definition of the outcome. Case-control studies: clear definition of cases.* | *Definition of the harmful outcome not reported or that leads to confusion. Terms not well-constructed, wrong definition.* |  |  |
| **D2. If applicable, is the severity of the harmful outcome clearly stated?** | | | | **No** |
|  | *N/A:* | *Yes:* | *No:* |  |
|  | *Self evident severity (e.g. death).* | *Detailed degree of severity or reference to a known scale of severity or a new scale developed for the study.* | *Unclear degrees of severity or without clear boundaries between them.* |  |
| **D3. Was the blinding methods of participants regarding the intervention appropriate considering the nature of the harmful outcome?** | | | | **Unclear** |
|  | *Yes:* | *Unclear:* | *No:* |  |
|  | *Blinding ensured (and unlikely broken) or outcome not likely to be influenced by lack of blinding.* | *There is no sufficient information regarding the process of blinding or the outcome assessment.* | *No blinding (or incomplete blinding or risk of broken blinding) and outcome likely to be influenced by lack of blinding.* |  |
| **D4. Was the blinding methods of harmful outcome assessment appropriate considering the nature of the harmful outcome?** | | | | **Unclear** |
|  | *Yes:* | *Unclear:* | *No:* |  |
|  | *Blinding ensured (and unlikely broken) or outcome assessment not likely to be influenced by lack of blinding.* | *There is no sufficient information regarding the process of blinding of outcome assessment.* | *No blinding (or incomplete blinding or risk of broken blinding) and outcome likely to be influenced by lack of blinding.* |  |
| **D5. Was the duration of follow-up adequate to assess the harmful outcome?** | | | | **Unclear** |
|  | *Yes:* | *Unclear:* | *No:* |  |
|  | *Sufficient duration of follow-up to assess the outcome.* | *It is unclear whether the duration of follow-up is adequate.* | *Too short duration of follow-up.* |  |
| **D6. Was the methods for ascertaining the harmful outcome adequately constructed and equal for all participants?** | | | | **Unclear** |
|  | *Yes:* | *Unclear:* | *No:* |  |
|  | *Adequate or validated methods of outcome measurement for all participants. Clinical reactions medically confirmed by a physician. Minimized risk of misclassification or differential assessment, reporting or detection.  RCT : Active harmful outcome surveillance (prospective/retrospective case-record review, questionnaires, patient’s diary/checklist…) .* | *There is no or not sufficient information to clearly determine how information on harmful outcome is collected or the process of minimizing misclassification.* | *Substantial risk of misclassification of outcome or differential assessment, reporting or detection. Clinical reactions not medically confirmed. RCT : Passive harmful outcome surveillance (patient’s volunteer reporting).* |  |
| **D7. Are the number of harmful outcome and the number of patients with a harmful outcome reported in both treatment arms?** | | | | **No** |
|  | *Yes:* | *Unclear:* | *No:* |  |
|  | *Numbers are reported. It is possible to calculate the rates of harmful outcome.* | *Confusion between the number of harmful outcomes or the number of patients with a harmful outcome, or general statements such as “5% of patients developed a harmful outcome”.* | *Neither the number of harmful outcomes nor the number of patients with a harmful outcome is reported. Or numbers are combining both treatment arms.* |  |
| **D8. Is the time frequency of harmful outcome assessment during the follow-up period appropriate?** | | | | **Unclear** |
|  | *Yes:* | *Unclear:* | *No:* |  |
|  | *For all study groups, the time frequency at which the harmful outcome is assessed is appropriate.* | *General statements such as “patients were routinely assessed for harmful outcomes”.* | *There is no regular collection of data on harmful outcomes during the study.* |  |
| **D9. Was the time between the exposure to a drug and the onset of the harmful outcome reported?** | | | | **No** |
|  | *Yes:* | *Unclear:* | *No:* |  |
|  | *The time between the drug exposure to the onset of harmful outcome is specified.* | *The authors do not report a clear time frame between drug exposure and harmful outcome.* | *The authors do not report the time between the drug exposure to the onset of harmful outcome.* |  |
| **D10. Was the process of determining that the harmful outcome is linked to the drug appropriate? Was the process blinded to the assigned treatment?** | | | | **No** |
|  | *Yes:* | *Unclear:* | *No:* |  |
|  | *Methods for causality assessment are appropriate and, if applicable, made by investigators blinded to the intervention.* | *Unclear how the causality attribution is made. It is not clear who make the assessment or whether it is blinded to the assigned treatment.* | *Causality assessment is made by investigators not blinded to the intervention, or by participants or sponsors, or unblinding of treatment assignment precedes the decision to withdraw.* |  |
| **D. RISK OF BIAS ASSESSMENT FOR INFORMATION BIAS REGARDING THE HARMFULL OUTCOME** | | | | **Unclear** |
|  | *Low:* | *Unclear:* | *High:* |  |
|  | *Plausible bias unlikely to seriously alter the results.* | *Plausible bias that raises some doubts about the results or when information on which to base risk of bias judgments is missing or poorly reported.* | *Plausible bias that seriously weakens confidence in the results.* |  |
| **Comments: Unclear risk of bias** | | | | |
| **E. OTHER INFORMATION BIAS** | | | | |
| **E1. Is blinding of care givers during follow-up adequately performed in order to avoid differential care between study groups (performance bias)?** | | | | **Unclear** |
|  | *Yes:* | *Unclear:* | *No:* |  |
|  | *There is no risk of differential care or it is adequately addressed.* | *Unclear risk of bias due to differential care.* | *The bias due to differential care is not controlled.* |  |
| **E5. Does the study appear free of other information bias ?** | | | | **Unclear** |
|  | *Yes:* | *Unclear:* | *No:* |  |
|  | *The study appears to be free of other information bias.* | *Unclear presence of other information bias.* | *Additional source of other information bias.* |  |
| **E. RISK OF BIAS ASSESSMENT FOR OTHER INFORMATION BIAS** | | | | **Unclear** |
|  | *Low:* | *Unclear:* | *High:* |  |
|  | *Plausible bias unlikely to seriously alter the results.* | *Plausible bias that raises some doubts about the results or when information on which to base risk of bias judgments is missing or poorly reported.* | *Plausible bias that seriously weakens confidence in the results.* |  |
| **Comments: Unclear of bias > open label** | | | | |
|  |  |  |  |  |
| **F. STATISTICAL METHODS TO CONTROL CONFOUNDING** | | | | |
| **F5. Does the study adequately address residual or unmeasured confounding?** | | | | **Yes** |
|  | *Yes:* | *Unclear:* | *No:* |  |
|  | *The study adequately addresses residual or unmeasured confounding.* | *Unclear presence of residual or unmeasured confounding.* | *Residual or unmeasured confounding is likely to be important.* |  |
| **F. RISK OF BIAS ASSESSMENT FOR STATISTICAL METHODS TO CONTROL CONFOUNDING:** | | | | **Low** |
|  | *Low:* | *Unclear:* | *High:* |  |
|  | *Plausible bias unlikely to seriously alter the results.* | *Plausible bias that raises some doubts about the results or when information on which to base risk of bias judgments is missing or poorly reported.* | *Plausible bias that seriously weakens confidence in the results.* |  |
| **Comments: Low risk of bias->Statistical Analysis section is well informative ( stratification by according to International Staging System disease stage20 (stage I or II vs. stage III, with higher stages indicating more severe disease) and age (≤60 years vs. 61 to 65 years)).** | | | | |
|  |  |  |  |  |
| **G. STATISTICAL METHODS EXCLUDING METHODS TO CONTROL CONFOUNDING** | | | | |
| **G1. Are the statistical methods used to analyze the harmful outcome appropriate?** | | | | **Unclear** |
|  | *Yes:* | *Unclear:* | *No:* |  |
|  | *Statistical techniques are appropriate to the data. If the distribution of the data (normal or not) is not described, it must be assumed that the estimates used were appropriate.* | *There is no description of the statistical techniques used, or the description is vague and not understandable.* | *The statistical techniques used are not appropriate.* |  |
| **G2. Is a survival analysis performed when there are individual differences in length of follow-up?** | | | | **Yes** |
|  | *Yes:* | *Unclear:* | *No:* |  |
|  | *Follow-up is the same for all study patients, if not survival analysis is performed.* | *Unclear whether there are different lengths of follow-up or whether they are taken into account.* | *Differences of follow up were ignored.* |  |
| **G3. If applicable, is composite outcome of harms adequately constructed?** | | | | **N/A** |
| *N/A* | *Yes:* | *Unclear:* | *No:* |  |
|  | *Composite outcome appropriate.* | *Unclear whether composite outcome is appropriate.* | *Construction of composite not described or not appropriate.* |  |
| **G6.** **Are the results consistent in primary and secondary analyses? Are confounding effects consistent with known associations?** | | | | **Yes** |
|  | *Yes:* | *Unclear:* | *No:* |  |
|  | *Consistency of primary, secondary analyses and consistency of confounding effects with known associations.* | *Not sufficient information to determine consistency.* | *No consistency of primary, secondary analyses or no consistency of confounding effects with known associations.* |  |
| **G. RISK OF BIAS ASSESSMENT FOR STATISTICAL METHODS EXCLUDING METHODS TO CONTROL CONFOUNDING:** | | | | **Unclear** |
|  | *Low:* | *Unclear:* | *High:* |  |
|  | *Plausible bias unlikely to seriously alter the results.* | *Plausible bias that raises some doubts about the results or when information on which to base risk of bias judgments is missing or poorly reported.* | *Plausible bias that seriously weakens confidence in the results.* |  |
| **Comments: Unclear risk of bias** | | | | |
|  | | | |  |
| **H. CONFLICT OF INTEREST** | | | | |
| **H1. Were the conflict of interest or sources of funding clearly acknowledged?** | | | | **Yes** |
|  | *Yes:* | *No:* |  |  |
|  | *Potential sources of support are acknowledged.* | *No sources of funding reported or not sufficient information.* |  |  |
| **H2. Does the study appear free of conflicts of interest susceptible to have influenced design, analysis or reporting (selective reporting of outcome or analysis)?** | | | | **Yes** |
|  | *Yes:* | *Unclear:* | *No:* |  |
|  | *No conflicts of interest or not susceptible to have influenced design, analysis or reporting.* | *It is unclear if there are conflicts of interest or if they are susceptible to have influenced design, analysis or reporting.* | *Conflicts of interest susceptible to have influenced design, analysis or reporting.* |  |
| **H. RISK OF BIAS ASSESSMENT FOR CONFLICT OF INTEREST** | | | | **Low** |
|  | *Low:* | *Unclear:* | *High:* |  |
|  | *Plausible bias unlikely to seriously alter the results.* | *Plausible bias that raises some doubts about the results or when information on which to base risk of bias judgments is missing or poorly reported.* | *Plausible bias that seriously weakens confidence in the results.* |  |
|  |  |  |  |  |
| **SUMMARY RISK-OF-BIAS ASSESSMENT FOR THE STUDY** | | | | |
| **RISK OF BIAS ASSESSMENT FOR THE STUDY** | | | | **Unclear** |
|  | *Low:* | *Unclear:* | *High:* |  |
|  | *Low risk of bias for all key domains.* | *Unclear risk of bias for one or more key domain.* | *High risk of bias for one or more key domains.* |  |

| **RISK OF BIAS ASSESSMENT CHECKLIST  FOR STUDIES INCLUDED IN SYSTEMATIC REVIEWS OF DRUG HARMS** | | | | |
| --- | --- | --- | --- | --- |
|  |  |  |  |  |
|  | **Study ID - Author** | Slade et al (2022), MMRC-066/201411060, NCT02253316 |  |  |
|  |  |  |  |  |
| **A. STUDY DESIGN AND OBJECTIVES** | | | | |
| **A1. Are study objectives clearly specified and appropriate?** | | | | **Yes** |
|  | *Yes:* | *No:* |  |  |
|  | *Study objectives clearly specified and appropriate.* | *Study objectives are not clearly specified or not appropriate.* |  |  |
| **A2. Is study design clearly specified and appropriate?** | | | | **Yes** |
|  | *Yes:* | *No:* |  |  |
|  | *Study design clearly specified and appropriate.* | *Study design not clearly specified or not appropriate.* |  |  |
| **A3. Is the study design free of run-in/lead-in period before inclusion/randomization of participants?** | | | | **Yes** |
|  | *Yes:* | *Unclear:* | *No:* |  |
|  | *No run-in/lead-in period.* | *Not clear information.* | *Presence of a run-in/lead-in period.* |  |
| **A4. Cross-over designs: Is the study designed to adequately address carry-over effect?** | | | | **N/A** |
| *N/A* | *Yes:* | *Unclear:* | *No:* |  |
|  | *Carry-over effect absent or adequately addressed (randomized order and sufficiently long wash-out period.* | *Not clear information.* | *Carry-over effect not adequately addressed and susceptible to bias the results.* |  |
| **A. RISK OF BIAS ASSESSMENT FOR STUDY DESIGN AND OBJECTIVES** | | | | **Low** |
|  | *Low:* | *Unclear:* | *High:* |  |
|  | *Plausible bias unlikely to seriously alter the results.* | *Plausible bias that raises some doubts about the results or when information on which to base risk of bias judgments is missing or poorly reported.* | *Plausible bias that seriously weakens confidence in the results.* |  |
| **Comments: Low risk of bias according to data provided by sources** | | | | |
|  |  |  |  |  |
| **B. BIAS IN SELECTION OF SUBJECTS AND CONSTITUTION OF STUDY GROUPS** | | | | |
| **B1. Was the method used to generate the allocation sequence adequate as to produce comparable groups?** | | | | **Unclear** |
|  | *Yes:* | *Unclear:* | *No:* |  |
|  | *Allocation methods are adequate to produce comparable groups.* | *Allocation methods are not clearly reported.* | *Allocation methods are not adequate (e.g. assignment to treatment by birth date, week day, etc.), groups are not comparable.* |  |
| **B2. Was the method used to conceal the allocation sequence adequate as to produce comparable groups?** | | | | **Yes** |
|  | *Yes:* | *Unclear:* | *No:* |  |
|  | *Concealment is adequate.* | *Concealment methods are not clearly reported and groups may not be comparable.* | *Concealment methods are not adequate, groups are not comparable.* |  |
| **B3. Are all the subjects recruited from the same source population?** | | | | **Yes** |
|  | *Yes:* | *Unclear:* | *No:* |  |
|  | *All the subjects recruited from the same source population.* | *Unclear if all the subjects recruited from the same source population.* | *All the subjects are not recruited from the same source population.* |  |
| **B4. Were inclusion and exclusion criteria implemented uniformly across study groups?** | | | | **Yes** |
|  | *Yes:* | *Unclear:* | *No:* |  |
|  | *Selection criteria uniformly implemented.* | *Unclear if selection criteria are uniformly implemented.* | *Selection criteria not uniformly implemented.* |  |
| **B8. Are baseline characteristics and prognostic factors comparable between different groups?** | | | | **Yes** |
|  | *Yes:* | *Unclear:* | *No:* |  |
|  | *RCT: Groups are comparable at baseline.* | *No description of baseline characteristics or only significance tests.* | *The groups are unbalanced at baseline.* |  |
|  | *Cohort studies: Groups are comparable at baseline or matched for the main prognostic factors.* |  |  |  |
| **B. RISK OF BIAS ASSESSMENT FOR SELECTION OF PARTICIPANTS AND CONSTITUTION OF STUDY GROUPS** | | | | **Unclear** |
|  | *Low:* | *Unclear:* | *High:* |  |
|  | *Plausible bias unlikely to seriously alter the results.* | *Plausible bias that raises some doubts about the results or when information on which to base risk of bias judgments is missing or poorly reported.* | *Plausible bias that seriously weakens confidence in the results.* |  |
| **Comments: unclear risk of bias according to data provided by sources** | | | | |
|  |  |  |  |  |
| **C. BIAS DUE TO WITHDRAWALS OR LOSS OF FOLLOW-UP (ATTRITION)** | | | | |
| **C1. Are the number of participants clearly reported throughout the study?** | | | | **No** |
|  | *Yes:* | *No:* |  |  |
|  | *Numbers of participants throughout the study are reported. Complete flow chart.* | *Numbers of patients at every stage is not clearly reported. Confusing information is reported regarding the number of participants. No or incomplete flow chart.* |  |  |
| **C2. Is the number of drop-outs/withdrawals due to harmful outcome clearly stated for each treatment arm?** | | | | **No** |
|  | *Yes:* | *No:* |  |  |
|  | *The number of drop-outs due to harmful outcome is specified.* | *The number of drop-outs due to harmful outcome is not specified, unclear or combined.* |  |  |
| **C3. Does the study adequately address biased loss to follow-up?** | | | | **Unclear** |
|  | *Yes:* | *Unclear:* | *No:* |  |
|  | *Complete follow-up or drop-outs unlikely to introduce bias or adequately controlled.* | *Drop-outs/withdraws due to harmful outcome are not clearly reported.* | *Loss to follow-up affects the safety outcome and is not adequately controlled.* |  |
| **C4. Are the results based on an intention-to-treat analysis?** | | | | **Yes** |
|  | *Yes:* | *Unclear:* | *No:* |  |
|  | *Results are based on a strict intention-to-treat analysis.* | *Not clear if an intention-to-treat analysis is performed. No strict intention-to-treat analysis.* | *Results are not based on intention-to-treat analysis (not done or not possible).* |  |
| **C. RISK OF BIAS DUE TO WITHDRAWALS OR LOSS OF FOLLOW-UP (ATTRITION)** | | | | **Unclear** |
|  | *Low:* | *Unclear:* | *High:* |  |
|  | *Plausible bias unlikely to seriously alter the results.* | *Plausible bias that raises some doubts about the results or when information on which to base risk of bias judgments is missing or poorly reported.* | *Plausible bias that seriously weakens confidence in the results.* |  |
| **Comments: unclear risk of bias according to data provided by sources** | | | | |
|  |  |  |  |  |
| **D. INFORMATION BIAS REGARDING THE HARMFULL OUTCOME** | | | | |
| **D1. Is the definition of the harmful outcome clearly stated?** | | | | **Yes** |
|  | *Yes:* | *No:* |  |  |
|  | *RCT: clear / standardized definition of the harmful outcome (e.g. diagnostic codes, clinical and laboratory data). Cohort studies: clear definition of the outcome. Case-control studies: clear definition of cases.* | *Definition of the harmful outcome not reported or that leads to confusion. Terms not well-constructed, wrong definition.* |  |  |
| **D2. If applicable, is the severity of the harmful outcome clearly stated?** | | | | **Yes** |
|  | *N/A:* | *Yes:* | *No:* |  |
|  | *Self evident severity (e.g. death).* | *Detailed degree of severity or reference to a known scale of severity or a new scale developed for the study.* | *Unclear degrees of severity or without clear boundaries between them.* |  |
| **D3. Was the blinding methods of participants regarding the intervention appropriate considering the nature of the harmful outcome?** | | | | **Unclear** |
|  | *Yes:* | *Unclear:* | *No:* |  |
|  | *Blinding ensured (and unlikely broken) or outcome not likely to be influenced by lack of blinding.* | *There is no sufficient information regarding the process of blinding or the outcome assessment.* | *No blinding (or incomplete blinding or risk of broken blinding) and outcome likely to be influenced by lack of blinding.* |  |
| **D4. Was the blinding methods of harmful outcome assessment appropriate considering the nature of the harmful outcome?** | | | | **Unclear** |
|  | *Yes:* | *Unclear:* | *No:* |  |
|  | *Blinding ensured (and unlikely broken) or outcome assessment not likely to be influenced by lack of blinding.* | *There is no sufficient information regarding the process of blinding of outcome assessment.* | *No blinding (or incomplete blinding or risk of broken blinding) and outcome likely to be influenced by lack of blinding.* |  |
| **D5. Was the duration of follow-up adequate to assess the harmful outcome?** | | | | **Unclear** |
|  | *Yes:* | *Unclear:* | *No:* |  |
|  | *Sufficient duration of follow-up to assess the outcome.* | *It is unclear whether the duration of follow-up is adequate.* | *Too short duration of follow-up.* |  |
| **D6. Was the methods for ascertaining the harmful outcome adequately constructed and equal for all participants?** | | | | **Unclear** |
|  | *Yes:* | *Unclear:* | *No:* |  |
|  | *Adequate or validated methods of outcome measurement for all participants. Clinical reactions medically confirmed by a physician. Minimized risk of misclassification or differential assessment, reporting or detection.  RCT : Active harmful outcome surveillance (prospective/retrospective case-record review, questionnaires, patient’s diary/checklist…) .* | *There is no or not sufficient information to clearly determine how information on harmful outcome is collected or the process of minimizing misclassification.* | *Substantial risk of misclassification of outcome or differential assessment, reporting or detection. Clinical reactions not medically confirmed. RCT : Passive harmful outcome surveillance (patient’s volunteer reporting).* |  |
| **D7. Are the number of harmful outcome and the number of patients with a harmful outcome reported in both treatment arms?** | | | | **Yes** |
|  | *Yes:* | *Unclear:* | *No:* |  |
|  | *Numbers are reported. It is possible to calculate the rates of harmful outcome.* | *Confusion between the number of harmful outcomes or the number of patients with a harmful outcome, or general statements such as “5% of patients developed a harmful outcome”.* | *Neither the number of harmful outcomes nor the number of patients with a harmful outcome is reported. Or numbers are combining both treatment arms.* |  |
| **D8. Is the time frequency of harmful outcome assessment during the follow-up period appropriate?** | | | | **Unclear** |
|  | *Yes:* | *Unclear:* | *No:* |  |
|  | *For all study groups, the time frequency at which the harmful outcome is assessed is appropriate.* | *General statements such as “patients were routinely assessed for harmful outcomes”.* | *There is no regular collection of data on harmful outcomes during the study.* |  |
| **D9. Was the time between the exposure to a drug and the onset of the harmful outcome reported?** | | | | **No** |
|  | *Yes:* | *Unclear:* | *No:* |  |
|  | *The time between the drug exposure to the onset of harmful outcome is specified.* | *The authors do not report a clear time frame between drug exposure and harmful outcome.* | *The authors do not report the time between the drug exposure to the onset of harmful outcome.* |  |
| **D10. Was the process of determining that the harmful outcome is linked to the drug appropriate? Was the process blinded to the assigned treatment?** | | | | **No** |
|  | *Yes:* | *Unclear:* | *No:* |  |
|  | *Methods for causality assessment are appropriate and, if applicable, made by investigators blinded to the intervention.* | *Unclear how the causality attribution is made. It is not clear who make the assessment or whether it is blinded to the assigned treatment.* | *Causality assessment is made by investigators not blinded to the intervention, or by participants or sponsors, or unblinding of treatment assignment precedes the decision to withdraw.* |  |
| **D. RISK OF BIAS ASSESSMENT FOR INFORMATION BIAS REGARDING THE HARMFULL OUTCOME** | | | | **Unclear** |
|  | *Low:* | *Unclear:* | *High:* |  |
|  | *Plausible bias unlikely to seriously alter the results.* | *Plausible bias that raises some doubts about the results or when information on which to base risk of bias judgments is missing or poorly reported.* | *Plausible bias that seriously weakens confidence in the results.* |  |
| **Comments: Unclear risk of bias** | | | | |
|  |  |  |  |  |
| **E. OTHER INFORMATION BIAS** | | | | |
| **E1. Is blinding of care givers during follow-up adequately performed in order to avoid differential care between study groups (performance bias)?** | | | | **Unclear** |
|  | *Yes:* | *Unclear:* | *No:* |  |
|  | *There is no risk of differential care or it is adequately addressed.* | *Unclear risk of bias due to differential care.* | *The bias due to differential care is not controlled.* |  |
| **E5. Does the study appear free of other information bias ?** | | | | **Unclear** |
|  | *Yes:* | *Unclear:* | *No:* |  |
|  | *The study appears to be free of other information bias.* | *Unclear presence of other information bias.* | *Additional source of other information bias.* |  |
| **E. RISK OF BIAS ASSESSMENT FOR OTHER INFORMATION BIAS** | | | | **Unclear** |
|  | *Low:* | *Unclear:* | *High:* |  |
|  | *Plausible bias unlikely to seriously alter the results.* | *Plausible bias that raises some doubts about the results or when information on which to base risk of bias judgments is missing or poorly reported.* | *Plausible bias that seriously weakens confidence in the results.* |  |
| **Comments: unclear risk of bias -> open label** | | | | |
|  |  |  |  |  |
| **F. STATISTICAL METHODS TO CONTROL CONFOUNDING** | | | | |
| **F5. Does the study adequately address residual or unmeasured confounding?** | | | | **Yes** |
|  | *Yes:* | *Unclear:* | *No:* |  |
|  | *The study adequately addresses residual or unmeasured confounding.* | *Unclear presence of residual or unmeasured confounding.* | *Residual or unmeasured confounding is likely to be important.* |  |
| **F. RISK OF BIAS ASSESSMENT FOR STATISTICAL METHODS TO CONTROL CONFOUNDING:** | | | | **Low** |
|  | *Low:* | *Unclear:* | *High:* |  |
|  | *Plausible bias unlikely to seriously alter the results.* | *Plausible bias that raises some doubts about the results or when information on which to base risk of bias judgments is missing or poorly reported.* | *Plausible bias that seriously weakens confidence in the results.* |  |
| **Comments: Low risk of bias->Statistical Analysis section is well informative ( stratification by mrd status).** | | | | |
|  |  |  |  |  |
| **G. STATISTICAL METHODS EXCLUDING METHODS TO CONTROL CONFOUNDING** | | | | |
| **G1. Are the statistical methods used to analyze the harmful outcome appropriate?** | | | | **Unclear** |
|  | *Yes:* | *Unclear:* | *No:* |  |
|  | *Statistical techniques are appropriate to the data. If the distribution of the data (normal or not) is not described, it must be assumed that the estimates used were appropriate.* | *There is no description of the statistical techniques used, or the description is vague and not understandable.* | *The statistical techniques used are not appropriate.* |  |
| **G2. Is a survival analysis performed when there are individual differences in length of follow-up?** | | | | **Yes** |
|  | *Yes:* | *Unclear:* | *No:* |  |
|  | *Follow-up is the same for all study patients, if not survival analysis is performed.* | *Unclear whether there are different lengths of follow-up or whether they are taken into account.* | *Differences of follow up were ignored.* |  |
| **G3. If applicable, is composite outcome of harms adequately constructed?** | | | | **N/A** |
| *N/A* | *Yes:* | *Unclear:* | *No:* |  |
|  | *Composite outcome appropriate.* | *Unclear whether composite outcome is appropriate.* | *Construction of composite not described or not appropriate.* |  |
| **G6.** **Are the results consistent in primary and secondary analyses? Are confounding effects consistent with known associations?** | | | | **Yes** |
|  | *Yes:* | *Unclear:* | *No:* |  |
|  | *Consistency of primary, secondary analyses and consistency of confounding effects with known associations.* | *Not sufficient information to determine consistency.* | *No consistency of primary, secondary analyses or no consistency of confounding effects with known associations.* |  |
| **G. RISK OF BIAS ASSESSMENT FOR STATISTICAL METHODS EXCLUDING METHODS TO CONTROL CONFOUNDING:** | | | | **Unclear** |
|  | *Low:* | *Unclear:* | *High:* |  |
|  | *Plausible bias unlikely to seriously alter the results.* | *Plausible bias that raises some doubts about the results or when information on which to base risk of bias judgments is missing or poorly reported.* | *Plausible bias that seriously weakens confidence in the results.* |  |
| **Comments: Unclear risk of bias** | | | | |
|  | | | |  |
| **H. CONFLICT OF INTEREST** | | | | |
| **H1. Were the conflict of interest or sources of funding clearly acknowledged?** | | | | **Yes** |
|  | *Yes:* | *No:* |  |  |
|  | *Potential sources of support are acknowledged.* | *No sources of funding reported or not sufficient information.* |  |  |
| **H2. Does the study appear free of conflicts of interest susceptible to have influenced design, analysis or reporting (selective reporting of outcome or analysis)?** | | | | **Yes** |
|  | *Yes:* | *Unclear:* | *No:* |  |
|  | *No conflicts of interest or not susceptible to have influenced design, analysis or reporting.* | *It is unclear if there are conflicts of interest or if they are susceptible to have influenced design, analysis or reporting.* | *Conflicts of interest susceptible to have influenced design, analysis or reporting.* |  |
| **H. RISK OF BIAS ASSESSMENT FOR CONFLICT OF INTEREST** | | | | **Low** |
|  | *Low:* | *Unclear:* | *High:* |  |
|  | *Plausible bias unlikely to seriously alter the results.* | *Plausible bias that raises some doubts about the results or when information on which to base risk of bias judgments is missing or poorly reported.* | *Plausible bias that seriously weakens confidence in the results.* |  |
|  |  |  |  |  |
| **SUMMARY RISK-OF-BIAS ASSESSMENT FOR THE STUDY** | | | | |
| **RISK OF BIAS ASSESSMENT FOR THE STUDY** | | | | **Unclear** |
|  | *Low:* | *Unclear:* | *High:* |  |
|  | *Low risk of bias for all key domains.* | *Unclear risk of bias for one or more key domain.* | *High risk of bias for one or more key domains.* |  |

| **RISK OF BIAS ASSESSMENT CHECKLIST  FOR STUDIES INCLUDED IN SYSTEMATIC REVIEWS OF DRUG HARMS** | | | | |
| --- | --- | --- | --- | --- |
|  |  |  |  |  |
|  | **Study ID - Author** | Weber et al (2007), CC-5013-MM-009, NCT00056160 |  |  |
|  |  |  |  |  |
| **A. STUDY DESIGN AND OBJECTIVES** | | | | |
| **A1. Are study objectives clearly specified and appropriate?** | | | | **Yes** |
|  | *Yes:* | *No:* |  |  |
|  | *Study objectives clearly specified and appropriate.* | *Study objectives are not clearly specified or not appropriate.* |  |  |
| **A2. Is study design clearly specified and appropriate?** | | | | **Yes** |
|  | *Yes:* | *No:* |  |  |
|  | *Study design clearly specified and appropriate.* | *Study design not clearly specified or not appropriate.* |  |  |
| **A3. Is the study design free of run-in/lead-in period before inclusion/randomization of participants?** | | | | **Yes** |
|  | *Yes:* | *Unclear:* | *No:* |  |
|  | *No run-in/lead-in period.* | *Not clear information.* | *Presence of a run-in/lead-in period.* |  |
| **A4. Cross-over designs: Is the study designed to adequately address carry-over effect?** | | | | **N/A** |
| *N/A* | *Yes:* | *Unclear:* | *No:* |  |
|  | *Carry-over effect absent or adequately addressed (randomized order and sufficiently long wash-out period.* | *Not clear information.* | *Carry-over effect not adequately addressed and susceptible to bias the results.* |  |
| **A. RISK OF BIAS ASSESSMENT FOR STUDY DESIGN AND OBJECTIVES** | | | | **Low** |
|  | *Low:* | *Unclear:* | *High:* |  |
|  | *Plausible bias unlikely to seriously alter the results.* | *Plausible bias that raises some doubts about the results or when information on which to base risk of bias judgments is missing or poorly reported.* | *Plausible bias that seriously weakens confidence in the results.* |  |
| **Comments: Low risk of bias according to data provided by sources** | | | | |
|  |  |  |  |  |
| **B. BIAS IN SELECTION OF SUBJECTS AND CONSTITUTION OF STUDY GROUPS** | | | | |
| **B1. Was the method used to generate the allocation sequence adequate as to produce comparable groups?** | | | | **Yes** |
|  | *Yes:* | *Unclear:* | *No:* |  |
|  | *Allocation methods are adequate to produce comparable groups.* | *Allocation methods are not clearly reported.* | *Allocation methods are not adequate (e.g. assignment to treatment by birth date, week day, etc.), groups are not comparable.* |  |
| **B2. Was the method used to conceal the allocation sequence adequate as to produce comparable groups?** | | | | **Yes** |
|  | *Yes:* | *Unclear:* | *No:* |  |
|  | *Concealment is adequate.* | *Concealment methods are not clearly reported and groups may not be comparable.* | *Concealment methods are not adequate, groups are not comparable.* |  |
| **B3. Are all the subjects recruited from the same source population?** | | | | **Yes** |
|  | *Yes:* | *Unclear:* | *No:* |  |
|  | *All the subjects recruited from the same source population.* | *Unclear if all the subjects recruited from the same source population.* | *All the subjects are not recruited from the same source population.* |  |
| **B4. Were inclusion and exclusion criteria implemented uniformly across study groups?** | | | | **Yes** |
|  | *Yes:* | *Unclear:* | *No:* |  |
|  | *Selection criteria uniformly implemented.* | *Unclear if selection criteria are uniformly implemented.* | *Selection criteria not uniformly implemented.* |  |
| **B8. Are baseline characteristics and prognostic factors comparable between different groups?** | | | | **Yes** |
|  | *Yes:* | *Unclear:* | *No:* |  |
|  | *RCT: Groups are comparable at baseline.* | *No description of baseline characteristics or only significance tests.* | *The groups are unbalanced at baseline.* |  |
|  | *Cohort studies: Groups are comparable at baseline or matched for the main prognostic factors.* |  |  |  |
| **B. RISK OF BIAS ASSESSMENT FOR SELECTION OF PARTICIPANTS AND CONSTITUTION OF STUDY GROUPS** | | | | **Low** |
|  | *Low:* | *Unclear:* | *High:* |  |
|  | *Plausible bias unlikely to seriously alter the results.* | *Plausible bias that raises some doubts about the results or when information on which to base risk of bias judgments is missing or poorly reported.* | *Plausible bias that seriously weakens confidence in the results.* |  |
| **Comments: Low risk of bias according to data provided by sources** | | | | |
|  |  |  |  |  |
| **C. BIAS DUE TO WITHDRAWALS OR LOSS OF FOLLOW-UP (ATTRITION)** | | | | |
| **C1. Are the number of participants clearly reported throughout the study?** | | | | **Yes** |
|  | *Yes:* | *No:* |  |  |
|  | *Numbers of participants throughout the study are reported. Complete flow chart.* | *Numbers of patients at every stage is not clearly reported. Confusing information is reported regarding the number of participants. No or incomplete flow chart.* |  |  |
| **C2. Is the number of drop-outs/withdrawals due to harmful outcome clearly stated for each treatment arm?** | | | | **Yes** |
|  | *Yes:* | *No:* |  |  |
|  | *The number of drop-outs due to harmful outcome is specified.* | *The number of drop-outs due to harmful outcome is not specified, unclear or combined.* |  |  |
| **C3. Does the study adequately address biased loss to follow-up?** | | | | **Yes** |
|  | *Yes:* | *Unclear:* | *No:* |  |
|  | *Complete follow-up or drop-outs unlikely to introduce bias or adequately controlled.* | *Drop-outs/withdraws due to harmful outcome are not clearly reported.* | *Loss to follow-up affects the safety outcome and is not adequately controlled.* |  |
| **C4. Are the results based on an intention-to-treat analysis?** | | | | **Yes** |
|  | *Yes:* | *Unclear:* | *No:* |  |
|  | *Results are based on a strict intention-to-treat analysis.* | *Not clear if an intention-to-treat analysis is performed. No strict intention-to-treat analysis.* | *Results are not based on intention-to-treat analysis (not done or not possible).* |  |
| **C. RISK OF BIAS DUE TO WITHDRAWALS OR LOSS OF FOLLOW-UP (ATTRITION)** | | | | **Low** |
|  | *Low:* | *Unclear:* | *High:* |  |
|  | *Plausible bias unlikely to seriously alter the results.* | *Plausible bias that raises some doubts about the results or when information on which to base risk of bias judgments is missing or poorly reported.* | *Plausible bias that seriously weakens confidence in the results.* |  |
| **Comments: Low risk of bias according to data provided by sources** | | | | |
|  |  |  |  |  |
| **D. INFORMATION BIAS REGARDING THE HARMFULL OUTCOME** | | | | |
| **D1. Is the definition of the harmful outcome clearly stated?** | | | | **No** |
|  | *Yes:* | *No:* |  |  |
|  | *RCT: clear / standardized definition of the harmful outcome (e.g. diagnostic codes, clinical and laboratory data). Cohort studies: clear definition of the outcome. Case-control studies: clear definition of cases.* | *Definition of the harmful outcome not reported or that leads to confusion. Terms not well-constructed, wrong definition.* |  |  |
| **D2. If applicable, is the severity of the harmful outcome clearly stated?** | | | | **N/A** |
|  | *N/A:* | *Yes:* | *No:* |  |
|  | *Self evident severity (e.g. death).* | *Detailed degree of severity or reference to a known scale of severity or a new scale developed for the study.* | *Unclear degrees of severity or without clear boundaries between them.* |  |
| **D3. Was the blinding methods of participants regarding the intervention appropriate considering the nature of the harmful outcome?** | | | | **Unclear** |
|  | *Yes:* | *Unclear:* | *No:* |  |
|  | *Blinding ensured (and unlikely broken) or outcome not likely to be influenced by lack of blinding.* | *There is no sufficient information regarding the process of blinding or the outcome assessment.* | *No blinding (or incomplete blinding or risk of broken blinding) and outcome likely to be influenced by lack of blinding.* |  |
| **D4. Was the blinding methods of harmful outcome assessment appropriate considering the nature of the harmful outcome?** | | | | **Unclear** |
|  | *Yes:* | *Unclear:* | *No:* |  |
|  | *Blinding ensured (and unlikely broken) or outcome assessment not likely to be influenced by lack of blinding.* | *There is no sufficient information regarding the process of blinding of outcome assessment.* | *No blinding (or incomplete blinding or risk of broken blinding) and outcome likely to be influenced by lack of blinding.* |  |
| **D5. Was the duration of follow-up adequate to assess the harmful outcome?** | | | | **Unclear** |
|  | *Yes:* | *Unclear:* | *No:* |  |
|  | *Sufficient duration of follow-up to assess the outcome.* | *It is unclear whether the duration of follow-up is adequate.* | *Too short duration of follow-up.* |  |
| **D6. Was the methods for ascertaining the harmful outcome adequately constructed and equal for all participants?** | | | | **Unclear** |
|  | *Yes:* | *Unclear:* | *No:* |  |
|  | *Adequate or validated methods of outcome measurement for all participants. Clinical reactions medically confirmed by a physician. Minimized risk of misclassification or differential assessment, reporting or detection.  RCT : Active harmful outcome surveillance (prospective/retrospective case-record review, questionnaires, patient’s diary/checklist…) .* | *There is no or not sufficient information to clearly determine how information on harmful outcome is collected or the process of minimizing misclassification.* | *Substantial risk of misclassification of outcome or differential assessment, reporting or detection. Clinical reactions not medically confirmed. RCT : Passive harmful outcome surveillance (patient’s volunteer reporting).* |  |
| **D7. Are the number of harmful outcome and the number of patients with a harmful outcome reported in both treatment arms?** | | | | **No** |
|  | *Yes:* | *Unclear:* | *No:* |  |
|  | *Numbers are reported. It is possible to calculate the rates of harmful outcome.* | *Confusion between the number of harmful outcomes or the number of patients with a harmful outcome, or general statements such as “5% of patients developed a harmful outcome”.* | *Neither the number of harmful outcomes nor the number of patients with a harmful outcome is reported. Or numbers are combining both treatment arms.* |  |
| **D8. Is the time frequency of harmful outcome assessment during the follow-up period appropriate?** | | | | **Unclear** |
|  | *Yes:* | *Unclear:* | *No:* |  |
|  | *For all study groups, the time frequency at which the harmful outcome is assessed is appropriate.* | *General statements such as “patients were routinely assessed for harmful outcomes”.* | *There is no regular collection of data on harmful outcomes during the study.* |  |
| **D9. Was the time between the exposure to a drug and the onset of the harmful outcome reported?** | | | | **No** |
|  | *Yes:* | *Unclear:* | *No:* |  |
|  | *The time between the drug exposure to the onset of harmful outcome is specified.* | *The authors do not report a clear time frame between drug exposure and harmful outcome.* | *The authors do not report the time between the drug exposure to the onset of harmful outcome.* |  |
| **D10. Was the process of determining that the harmful outcome is linked to the drug appropriate? Was the process blinded to the assigned treatment?** | | | | **No** |
|  | *Yes:* | *Unclear:* | *No:* |  |
|  | *Methods for causality assessment are appropriate and, if applicable, made by investigators blinded to the intervention.* | *Unclear how the causality attribution is made. It is not clear who make the assessment or whether it is blinded to the assigned treatment.* | *Causality assessment is made by investigators not blinded to the intervention, or by participants or sponsors, or unblinding of treatment assignment precedes the decision to withdraw.* |  |
| **D. RISK OF BIAS ASSESSMENT FOR INFORMATION BIAS REGARDING THE HARMFULL OUTCOME** | | | | **Unclear** |
|  | *Low:* | *Unclear:* | *High:* |  |
|  | *Plausible bias unlikely to seriously alter the results.* | *Plausible bias that raises some doubts about the results or when information on which to base risk of bias judgments is missing or poorly reported.* | *Plausible bias that seriously weakens confidence in the results.* |  |
| **Comments: Unclear risk of bias** | | | | |
| **E. OTHER INFORMATION BIAS** | | | | |
| **E1. Is blinding of care givers during follow-up adequately performed in order to avoid differential care between study groups (performance bias)?** | | | | **Yes** |
|  | *Yes:* | *Unclear:* | *No:* |  |
|  | *There is no risk of differential care or it is adequately addressed.* | *Unclear risk of bias due to differential care.* | *The bias due to differential care is not controlled.* |  |
| **E5. Does the study appear free of other information bias ?** | | | | **Yes** |
|  | *Yes:* | *Unclear:* | *No:* |  |
|  | *The study appears to be free of other information bias.* | *Unclear presence of other information bias.* | *Additional source of other information bias.* |  |
| **E. RISK OF BIAS ASSESSMENT FOR OTHER INFORMATION BIAS** | | | | **Low** |
|  | *Low:* | *Unclear:* | *High:* |  |
|  | *Plausible bias unlikely to seriously alter the results.* | *Plausible bias that raises some doubts about the results or when information on which to base risk of bias judgments is missing or poorly reported.* | *Plausible bias that seriously weakens confidence in the results.* |  |
| **Comments: Low of bias -> masking methods informations were well informative.** | | | | |
| **!!** |  |  |  |  |
| **F. STATISTICAL METHODS TO CONTROL CONFOUNDING** | | | | |
| **F5. Does the study adequately address residual or unmeasured confounding?** | | | | **Yes** |
|  | *Yes:* | *Unclear:* | *No:* |  |
|  | *The study adequately addresses residual or unmeasured confounding.* | *Unclear presence of residual or unmeasured confounding.* | *Residual or unmeasured confounding is likely to be important.* |  |
| **F. RISK OF BIAS ASSESSMENT FOR STATISTICAL METHODS TO CONTROL CONFOUNDING:** | | | | **Low** |
|  | *Low:* | *Unclear:* | *High:* |  |
|  | *Plausible bias unlikely to seriously alter the results.* | *Plausible bias that raises some doubts about the results or when information on which to base risk of bias judgments is missing or poorly reported.* | *Plausible bias that seriously weakens confidence in the results.* |  |
| **Comments: Low risk of bias->Statistical Analysis section is well informative ( stratification by the level of serum β2-microglobulin (<2.5 mg per liter vs. ≥2.5 mg per liter), previous stemcell transplantation (none vs. ≥1), and the number of previous antimyeloma therapies (1 vs. ≥2)).** | | | | |
|  |  |  |  |  |
| **G. STATISTICAL METHODS EXCLUDING METHODS TO CONTROL CONFOUNDING** | | | | |
| **G1. Are the statistical methods used to analyze the harmful outcome appropriate?** | | | | **Unclear** |
|  | *Yes:* | *Unclear:* | *No:* |  |
|  | *Statistical techniques are appropriate to the data. If the distribution of the data (normal or not) is not described, it must be assumed that the estimates used were appropriate.* | *There is no description of the statistical techniques used, or the description is vague and not understandable.* | *The statistical techniques used are not appropriate.* |  |
| **G2. Is a survival analysis performed when there are individual differences in length of follow-up?** | | | | **Yes** |
|  | *Yes:* | *Unclear:* | *No:* |  |
|  | *Follow-up is the same for all study patients, if not survival analysis is performed.* | *Unclear whether there are different lengths of follow-up or whether they are taken into account.* | *Differences of follow up were ignored.* |  |
| **G3. If applicable, is composite outcome of harms adequately constructed?** | | | | **N/A** |
| *N/A* | *Yes:* | *Unclear:* | *No:* |  |
|  | *Composite outcome appropriate.* | *Unclear whether composite outcome is appropriate.* | *Construction of composite not described or not appropriate.* |  |
| **G6.** **Are the results consistent in primary and secondary analyses? Are confounding effects consistent with known associations?** | | | | **Yes** |
|  | *Yes:* | *Unclear:* | *No:* |  |
|  | *Consistency of primary, secondary analyses and consistency of confounding effects with known associations.* | *Not sufficient information to determine consistency.* | *No consistency of primary, secondary analyses or no consistency of confounding effects with known associations.* |  |
| **G. RISK OF BIAS ASSESSMENT FOR STATISTICAL METHODS EXCLUDING METHODS TO CONTROL CONFOUNDING:** | | | | **Unclear** |
|  | *Low:* | *Unclear:* | *High:* |  |
|  | *Plausible bias unlikely to seriously alter the results.* | *Plausible bias that raises some doubts about the results or when information on which to base risk of bias judgments is missing or poorly reported.* | *Plausible bias that seriously weakens confidence in the results.* |  |
| **Comments: Unclear risk of bias** | | | | |
|  | | | |  |
| **H. CONFLICT OF INTEREST** | | | | |
| **H1. Were the conflict of interest or sources of funding clearly acknowledged?** | | | | **Yes** |
|  | *Yes:* | *No:* |  |  |
|  | *Potential sources of support are acknowledged.* | *No sources of funding reported or not sufficient information.* |  |  |
| **H2. Does the study appear free of conflicts of interest susceptible to have influenced design, analysis or reporting (selective reporting of outcome or analysis)?** | | | | **Yes** |
|  | *Yes:* | *Unclear:* | *No:* |  |
|  | *No conflicts of interest or not susceptible to have influenced design, analysis or reporting.* | *It is unclear if there are conflicts of interest or if they are susceptible to have influenced design, analysis or reporting.* | *Conflicts of interest susceptible to have influenced design, analysis or reporting.* |  |
| **H. RISK OF BIAS ASSESSMENT FOR CONFLICT OF INTEREST** | | | | **Low** |
|  | *Low:* | *Unclear:* | *High:* |  |
|  | *Plausible bias unlikely to seriously alter the results.* | *Plausible bias that raises some doubts about the results or when information on which to base risk of bias judgments is missing or poorly reported.* | *Plausible bias that seriously weakens confidence in the results.* |  |
|  |  |  |  |  |
| **SUMMARY RISK-OF-BIAS ASSESSMENT FOR THE STUDY** | | | | |
| **RISK OF BIAS ASSESSMENT FOR THE STUDY** | | | | **Unclear** |
|  | *Low:* | *Unclear:* | *High:* |  |
|  | *Low risk of bias for all key domains.* | *Unclear risk of bias for one or more key domain.* | *High risk of bias for one or more key domains.* |  |

| **RISK OF BIAS ASSESSMENT CHECKLIST  FOR STUDIES INCLUDED IN SYSTEMATIC REVIEWS OF DRUG HARMS** | | | | |
| --- | --- | --- | --- | --- |
|  |  |  |  |  |
|  | **Study ID - Author** | Zonder et al (2011), SWOG 0232, NCT00064038 |  |  |
|  |  |  |  |  |
| **A. STUDY DESIGN AND OBJECTIVES** | | | | |
| **A1. Are study objectives clearly specified and appropriate?** | | | | **Yes** |
|  | *Yes:* | *No:* |  |  |
|  | *Study objectives clearly specified and appropriate.* | *Study objectives are not clearly specified or not appropriate.* |  |  |
| **A2. Is study design clearly specified and appropriate?** | | | | **Yes** |
|  | *Yes:* | *No:* |  |  |
|  | *Study design clearly specified and appropriate.* | *Study design not clearly specified or not appropriate.* |  |  |
| **A3. Is the study design free of run-in/lead-in period before inclusion/randomization of participants?** | | | | **Yes** |
|  | *Yes:* | *Unclear:* | *No:* |  |
|  | *No run-in/lead-in period.* | *Not clear information.* | *Presence of a run-in/lead-in period.* |  |
| **A4. Cross-over designs: Is the study designed to adequately address carry-over effect?** | | | | **Yes** |
| *N/A* | *Yes:* | *Unclear:* | *No:* |  |
|  | *Carry-over effect absent or adequately addressed (randomized order and sufficiently long wash-out period.* | *Not clear information.* | *Carry-over effect not adequately addressed and susceptible to bias the results.* |  |
| **A. RISK OF BIAS ASSESSMENT FOR STUDY DESIGN AND OBJECTIVES** | | | | **Low** |
|  | *Low:* | *Unclear:* | *High:* |  |
|  | *Plausible bias unlikely to seriously alter the results.* | *Plausible bias that raises some doubts about the results or when information on which to base risk of bias judgments is missing or poorly reported.* | *Plausible bias that seriously weakens confidence in the results.* |  |
| **Comments: Low risk of bias according to data provided by sources** | | | | |
|  |  |  |  |  |
| **B. BIAS IN SELECTION OF SUBJECTS AND CONSTITUTION OF STUDY GROUPS** | | | | |
| **B1. Was the method used to generate the allocation sequence adequate as to produce comparable groups?** | | | | **Unclear** |
|  | *Yes:* | *Unclear:* | *No:* |  |
|  | *Allocation methods are adequate to produce comparable groups.* | *Allocation methods are not clearly reported.* | *Allocation methods are not adequate (e.g. assignment to treatment by birth date, week day, etc.), groups are not comparable.* |  |
| **B2. Was the method used to conceal the allocation sequence adequate as to produce comparable groups?** | | | | **Yes** |
|  | *Yes:* | *Unclear:* | *No:* |  |
|  | *Concealment is adequate.* | *Concealment methods are not clearly reported and groups may not be comparable.* | *Concealment methods are not adequate, groups are not comparable.* |  |
| **B3. Are all the subjects recruited from the same source population?** | | | | **Yes** |
|  | *Yes:* | *Unclear:* | *No:* |  |
|  | *All the subjects recruited from the same source population.* | *Unclear if all the subjects recruited from the same source population.* | *All the subjects are not recruited from the same source population.* |  |
| **B4. Were inclusion and exclusion criteria implemented uniformly across study groups?** | | | | **Yes** |
|  | *Yes:* | *Unclear:* | *No:* |  |
|  | *Selection criteria uniformly implemented.* | *Unclear if selection criteria are uniformly implemented.* | *Selection criteria not uniformly implemented.* |  |
| **B8. Are baseline characteristics and prognostic factors comparable between different groups?** | | | | **Yes** |
|  | *Yes:* | *Unclear:* | *No:* |  |
|  | *RCT: Groups are comparable at baseline.* | *No description of baseline characteristics or only significance tests.* | *The groups are unbalanced at baseline.* |  |
|  | *Cohort studies: Groups are comparable at baseline or matched for the main prognostic factors.* |  |  |  |
| **B. RISK OF BIAS ASSESSMENT FOR SELECTION OF PARTICIPANTS AND CONSTITUTION OF STUDY GROUPS** | | | | **Unclear** |
|  | *Low:* | *Unclear:* | *High:* |  |
|  | *Plausible bias unlikely to seriously alter the results.* | *Plausible bias that raises some doubts about the results or when information on which to base risk of bias judgments is missing or poorly reported.* | *Plausible bias that seriously weakens confidence in the results.* |  |
| **Comments: unclear risk of bias according to data provided by sources** | | | | |
|  |  |  |  |  |
| **C. BIAS DUE TO WITHDRAWALS OR LOSS OF FOLLOW-UP (ATTRITION)** | | | | |
| **C1. Are the number of participants clearly reported throughout the study?** | | | | **Yes** |
|  | *Yes:* | *No:* |  |  |
|  | *Numbers of participants throughout the study are reported. Complete flow chart.* | *Numbers of patients at every stage is not clearly reported. Confusing information is reported regarding the number of participants. No or incomplete flow chart.* |  |  |
| **C2. Is the number of drop-outs/withdrawals due to harmful outcome clearly stated for each treatment arm?** | | | | **yes** |
|  | *Yes:* | *No:* |  |  |
|  | *The number of drop-outs due to harmful outcome is specified.* | *The number of drop-outs due to harmful outcome is not specified, unclear or combined.* |  |  |
| **C3. Does the study adequately address biased loss to follow-up?** | | | | **Yes** |
|  | *Yes:* | *Unclear:* | *No:* |  |
|  | *Complete follow-up or drop-outs unlikely to introduce bias or adequately controlled.* | *Drop-outs/withdraws due to harmful outcome are not clearly reported.* | *Loss to follow-up affects the safety outcome and is not adequately controlled.* |  |
| **C4. Are the results based on an intention-to-treat analysis?** | | | | **Yes** |
|  | *Yes:* | *Unclear:* | *No:* |  |
|  | *Results are based on a strict intention-to-treat analysis.* | *Not clear if an intention-to-treat analysis is performed. No strict intention-to-treat analysis.* | *Results are not based on intention-to-treat analysis (not done or not possible).* |  |
| **C. RISK OF BIAS DUE TO WITHDRAWALS OR LOSS OF FOLLOW-UP (ATTRITION)** | | | | **Low** |
|  | *Low:* | *Unclear:* | *High:* |  |
|  | *Plausible bias unlikely to seriously alter the results.* | *Plausible bias that raises some doubts about the results or when information on which to base risk of bias judgments is missing or poorly reported.* | *Plausible bias that seriously weakens confidence in the results.* |  |
| **Comments: Low risk of bias according to data provided by sources** | | | | |
|  |  |  |  |  |
| **D. INFORMATION BIAS REGARDING THE HARMFULL OUTCOME** | | | | |
| **D1. Is the definition of the harmful outcome clearly stated?** | | | | **No** |
|  | *Yes:* | *No:* |  |  |
|  | *RCT: clear / standardized definition of the harmful outcome (e.g. diagnostic codes, clinical and laboratory data). Cohort studies: clear definition of the outcome. Case-control studies: clear definition of cases.* | *Definition of the harmful outcome not reported or that leads to confusion. Terms not well-constructed, wrong definition.* |  |  |
| **D2. If applicable, is the severity of the harmful outcome clearly stated?** | | | | **No** |
|  | *N/A:* | *Yes:* | *No:* |  |
|  | *Self evident severity (e.g. death).* | *Detailed degree of severity or reference to a known scale of severity or a new scale developed for the study.* | *Unclear degrees of severity or without clear boundaries between them.* |  |
| **D3. Was the blinding methods of participants regarding the intervention appropriate considering the nature of the harmful outcome?** | | | | **Unclear** |
|  | *Yes:* | *Unclear:* | *No:* |  |
|  | *Blinding ensured (and unlikely broken) or outcome not likely to be influenced by lack of blinding.* | *There is no sufficient information regarding the process of blinding or the outcome assessment.* | *No blinding (or incomplete blinding or risk of broken blinding) and outcome likely to be influenced by lack of blinding.* |  |
| **D4. Was the blinding methods of harmful outcome assessment appropriate considering the nature of the harmful outcome?** | | | | **Unclear** |
|  | *Yes:* | *Unclear:* | *No:* |  |
|  | *Blinding ensured (and unlikely broken) or outcome assessment not likely to be influenced by lack of blinding.* | *There is no sufficient information regarding the process of blinding of outcome assessment.* | *No blinding (or incomplete blinding or risk of broken blinding) and outcome likely to be influenced by lack of blinding.* |  |
| **D5. Was the duration of follow-up adequate to assess the harmful outcome?** | | | | **Unclear** |
|  | *Yes:* | *Unclear:* | *No:* |  |
|  | *Sufficient duration of follow-up to assess the outcome.* | *It is unclear whether the duration of follow-up is adequate.* | *Too short duration of follow-up.* |  |
| **D6. Was the methods for ascertaining the harmful outcome adequately constructed and equal for all participants?** | | | | **Unclear** |
|  | *Yes:* | *Unclear:* | *No:* |  |
|  | *Adequate or validated methods of outcome measurement for all participants. Clinical reactions medically confirmed by a physician. Minimized risk of misclassification or differential assessment, reporting or detection.  RCT : Active harmful outcome surveillance (prospective/retrospective case-record review, questionnaires, patient’s diary/checklist…) .* | *There is no or not sufficient information to clearly determine how information on harmful outcome is collected or the process of minimizing misclassification.* | *Substantial risk of misclassification of outcome or differential assessment, reporting or detection. Clinical reactions not medically confirmed. RCT : Passive harmful outcome surveillance (patient’s volunteer reporting).* |  |
| **D7. Are the number of harmful outcome and the number of patients with a harmful outcome reported in both treatment arms?** | | | | **No** |
|  | *Yes:* | *Unclear:* | *No:* |  |
|  | *Numbers are reported. It is possible to calculate the rates of harmful outcome.* | *Confusion between the number of harmful outcomes or the number of patients with a harmful outcome, or general statements such as “5% of patients developed a harmful outcome”.* | *Neither the number of harmful outcomes nor the number of patients with a harmful outcome is reported. Or numbers are combining both treatment arms.* |  |
| **D8. Is the time frequency of harmful outcome assessment during the follow-up period appropriate?** | | | | **Unclear** |
|  | *Yes:* | *Unclear:* | *No:* |  |
|  | *For all study groups, the time frequency at which the harmful outcome is assessed is appropriate.* | *General statements such as “patients were routinely assessed for harmful outcomes”.* | *There is no regular collection of data on harmful outcomes during the study.* |  |
| **D9. Was the time between the exposure to a drug and the onset of the harmful outcome reported?** | | | | **Unclear** |
|  | *Yes:* | *Unclear:* | *No:* |  |
|  | *The time between the drug exposure to the onset of harmful outcome is specified.* | *The authors do not report a clear time frame between drug exposure and harmful outcome.* | *The authors do not report the time between the drug exposure to the onset of harmful outcome.* |  |
| **D10. Was the process of determining that the harmful outcome is linked to the drug appropriate? Was the process blinded to the assigned treatment?** | | | | **Unclear** |
|  | *Yes:* | *Unclear:* | *No:* |  |
|  | *Methods for causality assessment are appropriate and, if applicable, made by investigators blinded to the intervention.* | *Unclear how the causality attribution is made. It is not clear who make the assessment or whether it is blinded to the assigned treatment.* | *Causality assessment is made by investigators not blinded to the intervention, or by participants or sponsors, or unblinding of treatment assignment precedes the decision to withdraw.* |  |
| **D. RISK OF BIAS ASSESSMENT FOR INFORMATION BIAS REGARDING THE HARMFULL OUTCOME** | | | | **Unclear** |
|  | *Low:* | *Unclear:* | *High:* |  |
|  | *Plausible bias unlikely to seriously alter the results.* | *Plausible bias that raises some doubts about the results or when information on which to base risk of bias judgments is missing or poorly reported.* | *Plausible bias that seriously weakens confidence in the results.* |  |
| **Comments: unclear risk of bias** | | | | |
|  |  |  |  |  |
| **E. OTHER INFORMATION BIAS** | | | | |
| **E1. Is blinding of care givers during follow-up adequately performed in order to avoid differential care between study groups (performance bias)?** | | | | **Yes** |
|  | *Yes:* | *Unclear:* | *No:* |  |
|  | *There is no risk of differential care or it is adequately addressed.* | *Unclear risk of bias due to differential care.* | *The bias due to differential care is not controlled.* |  |
| **E5. Does the study appear free of other information bias ?** | | | | **Yes** |
|  | *Yes:* | *Unclear:* | *No:* |  |
|  | *The study appears to be free of other information bias.* | *Unclear presence of other information bias.* | *Additional source of other information bias.* |  |
| **E. RISK OF BIAS ASSESSMENT FOR OTHER INFORMATION BIAS** | | | | **Low** |
|  | *Low:* | *Unclear:* | *High:* |  |
|  | *Plausible bias unlikely to seriously alter the results.* | *Plausible bias that raises some doubts about the results or when information on which to base risk of bias judgments is missing or poorly reported.* | *Plausible bias that seriously weakens confidence in the results.* |  |
| **Comments: Low risk of bias according to data provided by sources** | | | | |
|  |  |  |  |  |
| **F. STATISTICAL METHODS TO CONTROL CONFOUNDING** | | | | |
| **F5. Does the study adequately address residual or unmeasured confounding?** | | | | **Yes** |
|  | *Yes:* | *Unclear:* | *No:* |  |
|  | *The study adequately addresses residual or unmeasured confounding.* | *Unclear presence of residual or unmeasured confounding.* | *Residual or unmeasured confounding is likely to be important.* |  |
| **F. RISK OF BIAS ASSESSMENT FOR STATISTICAL METHODS TO CONTROL CONFOUNDING:** | | | | **Low** |
|  | *Low:* | *Unclear:* | *High:* |  |
|  | *Plausible bias unlikely to seriously alter the results.* | *Plausible bias that raises some doubts about the results or when information on which to base risk of bias judgments is missing or poorly reported.* | *Plausible bias that seriously weakens confidence in the results.* |  |
| **Comments: Low risk of bias according to data provided by sources. The stratification factors were International Staging System stage4 (1 vs 2 vs 3) and Zubrod performance status (0 or 1 vs 2 or 3).** | | | | |
|  |  |  |  |  |
| **G. STATISTICAL METHODS EXCLUDING METHODS TO CONTROL CONFOUNDING** | | | | |
| **G1. Are the statistical methods used to analyze the harmful outcome appropriate?** | | | | **Unclear** |
|  | *Yes:* | *Unclear:* | *No:* |  |
|  | *Statistical techniques are appropriate to the data. If the distribution of the data (normal or not) is not described, it must be assumed that the estimates used were appropriate.* | *There is no description of the statistical techniques used, or the description is vague and not understandable.* | *The statistical techniques used are not appropriate.* |  |
| **G2. Is a survival analysis performed when there are individual differences in length of follow-up?** | | | | **Yes** |
|  | *Yes:* | *Unclear:* | *No:* |  |
|  | *Follow-up is the same for all study patients, if not survival analysis is performed.* | *Unclear whether there are different lengths of follow-up or whether they are taken into account.* | *Differences of follow up were ignored.* |  |
| **G3. If applicable, is composite outcome of harms adequately constructed?** | | | | **N/A** |
| *N/A* | *Yes:* | *Unclear:* | *No:* |  |
|  | *Composite outcome appropriate.* | *Unclear whether composite outcome is appropriate.* | *Construction of composite not described or not appropriate.* |  |
| **G6.** **Are the results consistent in primary and secondary analyses? Are confounding effects consistent with known associations?** | | | | **Yes** |
|  | *Yes:* | *Unclear:* | *No:* |  |
|  | *Consistency of primary, secondary analyses and consistency of confounding effects with known associations.* | *Not sufficient information to determine consistency.* | *No consistency of primary, secondary analyses or no consistency of confounding effects with known associations.* |  |
| **G. RISK OF BIAS ASSESSMENT FOR STATISTICAL METHODS EXCLUDING METHODS TO CONTROL CONFOUNDING:** | | | | **Unclear** |
|  | *Low:* | *Unclear:* | *High:* |  |
|  | *Plausible bias unlikely to seriously alter the results.* | *Plausible bias that raises some doubts about the results or when information on which to base risk of bias judgments is missing or poorly reported.* | *Plausible bias that seriously weakens confidence in the results.* |  |
| **Comments: unclear risk of bias according to data provided by sources** | | | | |
|  | | | |  |
| **H. CONFLICT OF INTEREST** | | | | |
| **H1. Were the conflict of interest or sources of funding clearly acknowledged?** | | | | **Yes** |
|  | *Yes:* | *No:* |  |  |
|  | *Potential sources of support are acknowledged.* | *No sources of funding reported or not sufficient information.* |  |  |
| **H2. Does the study appear free of conflicts of interest susceptible to have influenced design, analysis or reporting (selective reporting of outcome or analysis)?** | | | | **Yes** |
|  | *Yes:* | *Unclear:* | *No:* |  |
|  | *No conflicts of interest or not susceptible to have influenced design, analysis or reporting.* | *It is unclear if there are conflicts of interest or if they are susceptible to have influenced design, analysis or reporting.* | *Conflicts of interest susceptible to have influenced design, analysis or reporting.* |  |
| **H. RISK OF BIAS ASSESSMENT FOR CONFLICT OF INTEREST** | | | | **Low** |
|  | *Low:* | *Unclear:* | *High:* |  |
|  | *Plausible bias unlikely to seriously alter the results.* | *Plausible bias that raises some doubts about the results or when information on which to base risk of bias judgments is missing or poorly reported.* | *Plausible bias that seriously weakens confidence in the results.* |  |
|  |  |  |  |  |
| **SUMMARY RISK-OF-BIAS ASSESSMENT FOR THE STUDY** | | | | |
| **RISK OF BIAS ASSESSMENT FOR THE STUDY** | | | | **Unclear** |
|  | *Low:* | *Unclear:* | *High:* |  |
|  | *Low risk of bias for all key domains.* | *Unclear risk of bias for one or more key domain.* | *High risk of bias for one or more key domains.* |  |

# Supplementary Table 7. Quality of evidence with Grading of Recommendations Assessment, Development and Evaluation system (GRADE).

| **Certainty assessment** | | | | | | | **Summary of findings** | | | | |
| --- | --- | --- | --- | --- | --- | --- | --- | --- | --- | --- | --- |
| **Participants (studies) Follow-up** | **Risk of bias** | **Inconsistency** | **Indirectness** | **Imprecision** | **Publication bias** | **Overall certainty of evidence** | **Study event rates (%)** | | **Relative effect (95% CI)** | **Anticipated absolute effects** | |
|  |  |  |  |  |  |  | **With control** | **With lenalidomide** |  | **Risk with control** | **Risk difference with lenalidomide** |
| **Placebo RCTs** | | | | | | | | | | | |
| 2362 (8 RCTs) | serious | not serious | serious | not serious | strong association | ⨁⨁⨁◯ Moderate | 2/1172 (0.2%) | 10/1190 (0.8%) | **OR 3.82** (1.22 to 11.92) | 2 per 1 000 | **5 more per 1 000** (from 0 fewer to 18 more) |
| **Open-label RCTs** | | | | | | | | | | | |
| 1001 (6 RCTs) | serious | not serious | serious | not serious | very strong association | ⨁⨁⨁⨁ High | 0/466 (0.0%) | 3/535 (0.6%) | **OR 6.49** (0.66 to 63.55) | 0 per 1 000 | **0 fewer per 1 000** (from 0 fewer to 0 fewer) |
| **Observation RCTs** | | | | | | | | | | | |
| 2617 (4 RCTs) | serious | not serious | serious | not serious | very strong association | ⨁⨁⨁⨁ High | 0/1077 (0.0%) | 5/1540 (0.3%) | **OR 5.29** (0.88 to 31.73) | 0 per 1 000 | **0 fewer per 1 000** (from 0 fewer to 0 fewer) |
| **All RCTs** | | | | | | | | | | | |
| 5980 (18 RCTs) | serious | not serious | serious | not serious | strong association | ⨁⨁⨁◯ Moderate | 2/2715 (0.1%) | 18/3265 (0.6%) | **OR 4.48** (1.85 to 10.86) | 1 per 1 000 | **3 more per 1 000** (from 1 more to 7 more) |

***Legends**: **CI:** confidence interval; **OR:** Peto Odds Ratio

***Reference**: (from GRADEpro GDT: GRADEpro Guideline Development Tool [Software]. McMaster University, 2015 (developed by Evidence Prime, Inc.). Available from [gradepro.org](https://gradepro.org/cite/gradepro.org).

***Interpretation**:

| **Grade** | **Definition** |
| --- | --- |
| High | Further research is very unlikely to change our confidence in the estimate of effect. |
| Moderate | Further research is likely to have an important impact on our confidence in the estimate of effect and may change the estimate. |
| Low | Further research is very likely to have an important impact on our confidence in the estimate of effect and is likely to change the estimate. |
| Very low | Any estimate of effect is very uncertain |

# Supplementary Table 8. Subgroup analyses on the risk of therapy-associated acute lymphoblastic leukaemia with lenalidomide versus placebo (A) or all controls (B).

ALL=Acute Lymphoblastic Leukaemia. NA=Not applicable. RCT=randomized controlled trial.

**A Lenalidomide versus placebo (n=8 RCTs)**

| **Subgroup analyses** | **Peto odds ratio**  **(95% CI)** | ***I²* (%); χ²**  **p value** | **χ² p value**  **for subgroup differences** |
| --- | --- | --- | --- |
| **Age at baseline, years** |  |  |  |
| <60 | 3·59 [1·09-11·79] | 0%; 0·47 | 0·73 |
| 60-70 | No event reported | NA | ·· |
| >70 | Not estimable | NA | ·· |
| unavailable | No event reported | NA | ·· |
| **Multiple myeloma disease** |  |  |  |
| Newly diagnosed | 3·82 [1·22-11·92] | 0%; 0·72 | NA |
| Relapsed or refractory | No event reported | NA | ·· |
| **Previous exposure to lenalidomide** |  |  |  |
| Yes | Not estimable | NA | 0·42 |
| No | 7·38 [1·04-52·40] | 0%; 0·99 | ·· |
| **Previous exposure to drug inducing ALL** |  |  |  |
| Yes | Not estimable | NA | 0·51 |
| No | 3·06 [0·82-11·42] | 0%; 0·64 | ·· |
| **Previous stem-cell transplantation** |  |  |  |
| Yes | 3·59 [1·09-11·79] | 0%; 0·47 | 0·73 |
| No | Not estimable | NA | ·· |
| **Lenalidomide starting dose** |  |  |  |
| 25mg/day | Not estimable | NA | 0·51 |
| 10mg/day | 3·06 [0·82-11·42] | 0%; 0·64 | ·· |
| **Lenalidomide regimen** |  |  |  |
| Alone | 3·59 [1·09-11·79] | 0%; 0·47 | 0·73 |
| Combination | Not estimable | NA | ·· |
| **Lenalidomide setting use** |  |  |  |
| Induction and maintenance therapy | Not estimable | NA | 0·72 |
| Consolidation and maintenance therapy | Not estimable | NA | ·· |
| Maintenance therapy | Not estimable | NA | ·· |
| Relapse or refractory | No event reported | NA | ·· |
| **Lenalidomide duration, months** |  |  |  |
| 0-12 | No event reported | NA | 0·73 |
| 12-24 | Not estimable | NA | ·· |
| 24-36 | 3·59 [1·09-11·79] | 0%; 0·47 | ·· |
| unavailable | No event reported | NA | ·· |
| **Follow-up length, years** |  |  |  |
| ≥3 | 3·59 [1·09-11·79] | 0%; 0·47 | 0·73 |
| <3 | Not estimable | NA | ·· |
| unavailable | No event reported | NA | ·· |
| **Study stopped early** |  |  |  |
| Yes | No event reported | NA | NA |
| No | 3·82 [1·22-11·92] | 0%; 0·72 | ·· |

**B Lenalidomide versus any control (n=18 RCTs)**

| **Subgroup analyses** | **Peto odds ratio**  **(95% CI)** | ***I²* (%); χ²**  **p value** | **χ² p value**  **for subgroup differences** |
| --- | --- | --- | --- |
| **Age at baseline, years** |  |  |  |
| <60 | 4·07 [1·42-11·70] | 0%; 0·69 | 0·94 |
| 60-70 | No event reported | NA | ·· |
| >70 | Not estimable | NA | ·· |
| unavailable | No event reported | NA | ·· |
| **Multiple myeloma disease** |  |  |  |
| Smoldering | No event reported | NA | NA |
| Newly diagnosed | 4·48 [1·85-10·86] | 0%; 0·93 | ·· |
| Relapsed or refractory | No event reported | NA | ·· |
| **Previous exposure to lenalidomide** |  |  |  |
| Yes | 3·94 [1·46-10·63] | 0%; 0·76 | 0·58 |
| No | 7·38 [1·04-52·50] | 0%; 0·99 | ·· |
| **Previous exposure to drug inducing ALL** |  |  |  |
| Yes | 6·13 [1·85-20·29] | 0%; 0·97 | 0·44 |
| No | 3·06 [0·82-11·42] | 0%; 0·64 | ·· |
| **Previous stem-cell transplantation** |  |  |  |
| Yes | 4·36 [1·76-10·81] | 0%; 0·85 | 0·79 |
| No | Not estimable | NA | ·· |
| unavailable | No event reported | NA | ·· |
| **Lenalidomide starting dose** |  |  |  |
| 25mg/day | 6·00 [1·47-24·47] | 0%; 0·82 | 0·60 |
| 15mg/day or 25mg/day | No event reported | NA | ·· |
| 15mg/day | No event reported | NA | ·· |
| 10mg/day | 3·70 [1·18-11·55] | 0%; 0·76 | ·· |
| **Lenalidomide regimen** |  |  |  |
| Alone | 4·36 [1·76-10·81] | 0%; 0·85 | 0·79 |
| Combination | Not estimable | NA | ·· |
| **Lenalidomide setting use** |  |  |  |
| Prevention of end organ damage | No event reported | NA | 0·86 |
| Induction therapy | No event reported | NA | ·· |
| Induction, intensification, consolidation and maintenance therapy | No event reported | NA | ·· |
| Induction and maintenance therapy | Not estimable | NA | ·· |
| Consolidation therapy | No event reported | NA | ·· |
| Consolidation and maintenance therapy | Not estimable | NA | ·· |
| Maintenance therapy | 3·94 [1·46-10·63] | 0%; 0·76 | ·· |
| Relapse or refractory | No event reported | NA | ·· |
| **Lenalidomide duration, months** |  |  |  |
| 0-12 | No event reported | NA | 0·86 |
| 12-24 | 5·61 [1·10-28·65] | 0%; 0·87 | ·· |
| 24-36 | 3·59 [1·09-11·79] | 0%; 0·47 | ·· |
| unavailable | Not estimable | NA | ·· |
| **Follow-up length, years** |  |  |  |
| ≥3 | 4·04 [1·50-10·88] | 0%; 0·72 | 0·65 |
| <3 | 6·73 [0·94-48·35] | 0%; 0·95 | ·· |
| unavailable | No event reported | NA | ·· |
| **Study stopped early** |  |  |  |
| Yes | No event reported | NA | NA |
| No | 4·48 [1·85-10·86] | 0%; 0·93 | ·· |
| unavailable | No event reported | NA |  |

# Supplementary Figure 4. Sensitivity analysis on the risk of therapy-associated acute lymphoblastic leukaemia with lenalidomide versus placebo in RCTs (n=8).

Sensitivity analyses to evaluate the contribution of the number of acute lymphoblastic leukaemia reported in supplementary appendix of placebo CALGB 100104 trial. ALL=Acute Lymphoblastic Leukaemia. NA=Not applicable. RCT=randomized controlled trial.


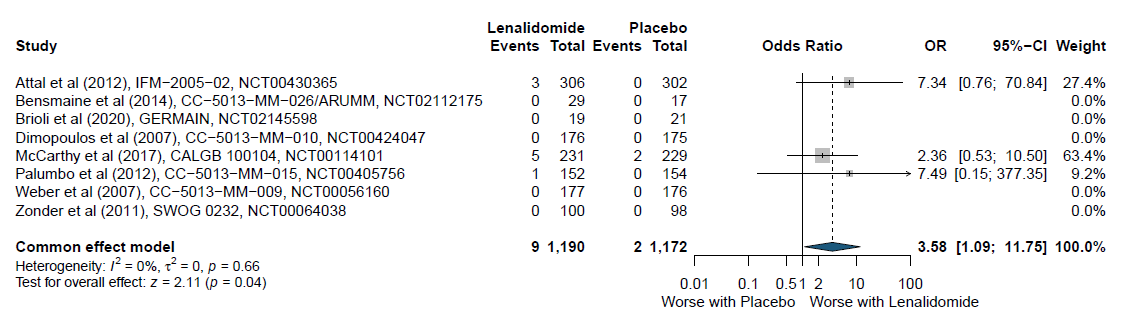


# **Supplementary Table 9. Characteristics of patients diagnosed with acute lymphoblastic leukaemia following lenalidomide use from the WHO’s pharmacovigilance database**. Data collection was on March 1, 2024 according to VigiBase’s Extract Case Level function.

|  | All cases (n=269) | ·· |
| --- | --- | --- |
| **Year of report** | ·· | ·· |
| Total data | 269 | ·· |
| 2023 | 86 | 32% |
| 2022 | 29 | 11% |
| 2021 | 11 | 4% |
| 2020 | 13 | 5% |
| 2019 | 23 | 9% |
| 2018 | 25 | 9% |
| 2017 | 16 | 6% |
| 2016 | 5 | 2% |
| 2015 | 25 | 9% |
| 2014 | 17 | 6% |
| 2013 | 1 | 0% |
| 2012 | 9 | 3% |
| 2011 | 3 | 1% |
| 2010 | 2 | 1% |
| 2009 | 1 | 0% |
| 2008 | 3 | 1% |
| **Type of report** | ·· | ·· |
| Total data | 268 | ·· |
| Spontaneous | 209 | 78% |
| Report from study | 59 | 22% |
| **Acute lymphoblastic leukaemia types** | ·· | ·· |
| Total data | 269 | ·· |
| Acute lymphoblastic leukaemia | 187 | 69% |
| B-cell type acute leukaemia | 81 | 30% |
| Philadelphia positive acute lymphocytic leukaemia | 1 | <1% |
| **Age at onset, years** | ·· | ·· |
| Total data | 218 |  |
| <18 | 1 | <1% |
| 18-45 | 4 | 2% |
| 45-64 | 90 | 41% |
| 65-74 | 102 | 47% |
| 75+ | 21 | 10% |
| **Sex** | ·· | ·· |
| Total data | 251 |  |
| Male | 151 | 60% |
| Female | 100 | 40% |
| **Geographical location** | ·· | ·· |
| Total data | 269 | ·· |
| Americas | 242 | 90% |
| European | 19 | 7% |
| Western Pacific | 8 | 3% |
| **Reporter** | ·· | ·· |
| Total data | 265 | ·· |
| Physician | 103 | 39% |
| Pharmacist | 18 | 7% |
| Other Health Professional | 137 | 52% |
| Consumer or other non health professional | 7 | 3% |
| **Lenalidomide indications** | ·· | ·· |
| Total data | 260 | ·· |
| Multiple myeloma | 241 | 93% |
| Non-ALL leukaemias | 7 | 3% |
| Lymphomas | 3 | 1% |
| Other | 9 | 3% |
| **Serious cases** | ·· | ·· |
| Total data | 269 | ·· |
| Yes | 267 | 99% |
| No | 2 | 1% |
| **Outcomes following ALL onset** | ·· | ·· |
| Total data | 100 | ·· |
| Not recovered/not resolved | 42 | 42% |
| Death | 24 | 24% |
| Recovered/resolved | 22 | 22% |
| Recovering/resolving | 12 | 12% |
| **Treatments modifications folllowing ALL** | ·· | ·· |
| Total data | 89 | ·· |
| Drug withdrawn | 83 | 93% |
| Dose not changed | 3 | 3% |
| Dose increased | 2 | 2% |
| Dose reduced | 1 | 1% |
| **Co-suspected anticancer drugs** | ·· | ·· |
| Total data | 269 | ·· |
| dexamethasone | 90 | 33% |
| bortezomib | 57 | 21% |
| cyclophosphamide | 42 | 16% |
| melphalan | 36 | 13% |
| carfilzomib | 19 | 7% |
| thalidomide | 13 | 5% |
| pomalidomide | 10 | 4% |
| vincristine | 7 | 3% |
| doxorubicin | 5 | 2% |
| rituximab | 5 | 2% |
| methotrexate | 4 | 1% |
| cytarabine | 3 | 1% |
| **Co-reported adverse events ≥5%** | ·· | ·· |
| Total data | 67 | ·· |
| pancytopenia | 10 | 15% |
| cytopenia | 6 | 9% |
| infection | 6 | 9% |
| febrile neutropenia | 4 | 6% |
| plasma cell myeloma recurrent | 4 | 6% |
| pneumonia | 4 | 6% |
| sepsis | 4 | 6% |
| thrombocytopenia | 4 | 6% |

# Supplementary Table 10. Review of litterature associated to ALL post-lenalidomide in patients with multiple myeloma. *All patients received IMiDs including 6 (46,5%) lenalidomide, data available for the entire cohort.

| **Study reference** | **n** | **Median Age** | **Time from MM to ALL, years** | **ASCT with melphalan conditionning n, (%)** | **lenalidomide exposure duration, months** | **Low hypodiploidy ALL (%)** | ***TP53* mutation** | **Overall survival** |
| --- | --- | --- | --- | --- | --- | --- | --- | --- |
| **Geyer et al, *Blood Adv***^31^**, 2023** | 32 | 65 (50-86) | 5.5 (0.6-11.7) | 22/32 (69%) | 41.8 (1.8-114.1) | 1/4 (25%) | 9/19 (47%) | 35.8 months |
| **Barnell et al, *Blood Advances***^32^**, 2023** | 17 | 60 (45-74) | NA | 15/17 (88%) | 45.6 (12-156) | NA | 14/17 (82%) | NA |
| **Crosby et al, *J Investig Med High Impact Case Rep***^33^**, 2022** | 1 | 71 | ~6 years | 1 (100%) | ~6 years | 1 (100%) | NA | NA (soon after treatment  was discontinued) |
| **Germans et al, *Am J Clin Pathol***^34^**, 2020** | 2 | 53.5 (43-64) | NA | 2/2 (100%) | 48 (20.5-75.5) | NA | 2/2 (100%) | > 1 year |
| **Aldos et al, *Leukemia***^35^**, 2019** | 13* | 60 (43–67) | 5.4 (3.3-10) | 10/13 (77%) | NA | NA | 3/13 (23%) | 1 year OS: 77% (95% CI: 44%–92%) |
| **Tan et al, *Hematol Oncol***^36^**, 2017** | 3 | 53 (34-59) | 6 (2.5-7) | 2/3 (66%) | 36 (30-84) | 0 | NA | from 1 month to > 1 year |
| **Gustave Roussy database (not published)** | 3 | 74 (65-75) | 7.4 (4.7-15.5) | 2/3 (66%) | 28 (6-32) | 0 | NA | from 9 months to > 2 year |

# Supplementary Limitations of systematic review and pharmacovigilance study.

We aknowledge that our systematic review and meta-analysis exhibit some limitations. The first of them is related to the follow-up duration, which varies across trials. The 7·5 years median follow-up of CALGB 100104 trial^7^ is the longest of all placebo RCTs, followed by the ~3·75 years of IFM-2005-02 and SWOG-0232 studies^14,20^, and finally less than 2·5 years for the remaining trials. Based on the CALGB 100104 data, lenalidomide-associated ALL occurred with a median latency period of 5·1 [2·1-9·4] years (n=7). These lengths deal with the studies’ endpoints or early closure of the trials, but they are not designed to capture ALL events. Secondly, SPM monitoring practices differ between trials, and to date there is no consensus guideline to address it. This could be explained by the lack of studies assessing the risk or incidence of specific type of SPM (e.g., lung, prostate) occurring on case-by-case basis in patients with MM exposed to lenalidomide. We also noted that FDA Prescribing Information reports an increased incidence of haematologic and solid SPM in clinical trials in patients with MM.^37^ By excluding non-melanoma skin SPM, only AML and MDS hSPM are precisely listed. Overall, these differences might affect the estimation of risk and incidence of lenalidomide-associated ALL. Finally, we acknowledge that your work was focused on MM setting only ant therefore, we are not aware of the risk and incidence of t-ALL in chronic lymphocytic leukemia, lymphomas (diffuse large B-cell, follicular, mantle cell or marginal zone) or myelodysplastic syndromes.

Since 1968, VigiBase has aggregate over than 35 million reports of suspected AEs due to drug administration, provided by ~150 countries. Inherently to pharmacovigilance studies conducted in VigiBase, we do not have access to the initial workup that led to the diagnosis of lenalidomide-associated ALL, such as clinical evaluation (e.g., hepatosplenomegaly, lymphadenopathy) and laboratory tests (e.g., complete blood count, morphology or flow cytometry to characterise blast cells from peripheral blood, bone marrow or lymph node). Furthermore, cytogenetic and molecular features including *TP53* mutational status are also missing. Thus, we acknowledge that a definitive causal relationship between lenalidomide use and ALL onset cannot be formally ascertained.

# Supplementary references

1 Zorzela L, Loke YK, Ioannidis JP, Golder S, Santaguida P, Altman DG *et al.* PRISMA harms checklist: improving harms reporting in systematic reviews. *BMJ* 2016; **352**: i157.

2 WHOCC - ATC/DDD Index. https://www.whocc.no/atc_ddd_index/?code=L04AX04 (accessed 24 Jan2024).

3 Morice P-M, Leary A, Dolladille C, Chrétien B, Poulain L, González-Martín A *et al.* Myelodysplastic syndrome and acute myeloid leukaemia in patients treated with PARP inhibitors: a safety meta-analysis of randomised controlled trials and a retrospective study of the WHO pharmacovigilance database. *Lancet Haematol* 2021; **8**: e122–e134.

4 L01A ALKYLATING AGENTS WHOCC - ATC/DDD Index. https://www.whocc.no/atc_ddd_index/?code=L01A&showdescription=no (accessed 24 Jan2024).

5 L01DB Anthracyclines and related substances WHOCC - ATC/DDD Index. https://www.whocc.no/atc_ddd_index/?code=L01DB&showdescription=no (accessed 24 Jan2024).

6 Saleem K, Franz J, Klem ML, Yabes JG, Boyiadzis M, Jones JR *et al.* Second primary malignancies in patients with haematological cancers treated with lenalidomide: a systematic review and meta-analysis. *The Lancet Haematology* 2022; **9**: e906–e918.

7 Holstein SA, Jung S-H, Richardson PG, Hofmeister CC, Hurd DD, Hassoun H *et al.* Updated analysis of CALGB (Alliance) 100104 assessing lenalidomide versus placebo maintenance after single autologous stem-cell transplantation for multiple myeloma: a randomised, double-blind, phase 3 trial. *The Lancet Haematology* 2017; **4**: e431–e442.

8 Faillie J-L, Ferrer P, Gouverneur A, Driot D, Berkemeyer S, Vidal X *et al.* A new risk of bias checklist applicable to randomized trials, observational studies, and systematic reviews was developed and validated to be used for systematic reviews focusing on drug adverse events. *Journal of Clinical Epidemiology* 2017; **86**: 168–175.

9 Morton S, Murad M, O’Connor E, Lee C, Booth M, Vandermeer B *et al.* Quantitative Synthesis—An Update - Methods Guide for Effectiveness and Comparative Effectiveness Reviews - NCBI Bookshelf. 2018.https://www.ncbi.nlm.nih.gov/books/NBK519365/ (accessed 18 Mar2020).

10 Sedgwick P. Relative risks versus odds ratios. *BMJ* 2014; **348**. doi:10.1136/bmj.g1407.

11 Aldoss I, Stiller T, Tsai N-C, Song JY, Cao T, Bandara NA *et al.* Therapy-related acute lymphoblastic leukemia has distinct clinical and cytogenetic features compared to de novo acute lymphoblastic leukemia, but outcomes are comparable in transplanted patients. *Haematologica* 2018; **103**: 1662–1668.

12 Jonathan J Deeks, Julian PT Higgins, Douglas G Altman; on behalf of the Cochrane Statistical Methods Group. Chapter 10: Analysing data and undertaking meta-analyses. https://training.cochrane.org/handbook/current/chapter-10 (accessed 25 Jan2024).

13 Cancer Therapy Evaluation Program Adverse Event Reporting System (CTEP-AERS) | Protocol Development | CTEP. https://ctep.cancer.gov/protocoldevelopment/electronic_applications/adverse_events.htm (accessed 12 Apr2021).

14 Attal M, Lauwers-Cances V, Marit G, Caillot D, Moreau P, Facon T *et al.* Lenalidomide Maintenance after Stem-Cell Transplantation for Multiple Myeloma. *New England Journal of Medicine* 2012; **366**: 1782–1791.

15 EudraCT Number 2013-001729-26 - Clinical trial results - EU Clinical Trials Register. https://www.clinicaltrialsregister.eu/ctr-search/trial/2013-001729-26/results (accessed 30 Jan2024).

16 Brioli A, Manz K, Pfirrmann M, Hänel M, Schwarzer AC, Prange-Krex G *et al.* Frailty impairs the feasibility of induction therapy but not of maintenance therapy in elderly myeloma patients: final results of the German Maintenance Study (GERMAIN). *J Cancer Res Clin Oncol* 2020; **146**: 749–759.

17 A Multi-center, Randomized, Parallel-group, Double-blind, Placebo Controlled Study of CC-5013 Plus Dexamethasone Versus Dexamethasone Alone in Previously Treated Subjects With Multiple Myeloma. clinicaltrials.gov, 2017https://clinicaltrials.gov/study/NCT00424047 (accessed 1 Jan2024).

18 Palumbo A, Hajek R, Delforge M, Kropff M, Petrucci MT, Catalano J *et al.* Continuous lenalidomide treatment for newly diagnosed multiple myeloma. *N Engl J Med* 2012; **366**: 1759–1769.

19 Weber DM, Chen C, Niesvizky R, Wang M, Belch A, Stadtmauer EA *et al.* Lenalidomide plus dexamethasone for relapsed multiple myeloma in North America. *N Engl J Med* 2007; **357**: 2133–2142.

20 13th International Myeloma Workshop, Paris, France, May 3–6, 2011. *Haematologica* 2011; **96**: S1–S177.

21 Dimopoulos MA, Beksac M, Benboubker L, Roddie H, Allietta N, Broer E *et al.* Phase II study of bortezomib-dexamethasone alone or with added cyclophosphamide or lenalidomide for sub-optimal response as second-line treatment for patients with multiple myeloma. *Haematologica* 2013; **98**: 1264–1272.

22 Gay F, Musto P, Rota-Scalabrini D, Bertamini L, Belotti A, Galli M *et al.* Carfilzomib with cyclophosphamide and dexamethasone or lenalidomide and dexamethasone plus autologous transplantation or carfilzomib plus lenalidomide and dexamethasone, followed by maintenance with carfilzomib plus lenalidomide or lenalidomide alone for patients with newly diagnosed multiple myeloma (FORTE): a randomised, open-label, phase 2 trial. *Lancet Oncol* 2021; **22**: 1705–1720.

23 Jacobus SJ, Rajkumar SV, Weiss M, Stewart AK, Stadtmauer EA, Callander NS *et al.* Randomized phase III trial of consolidation therapy with bortezomib-lenalidomide-Dexamethasone (VRd) vs bortezomib-dexamethasone (Vd) for patients with multiple myeloma who have completed a dexamethasone based induction regimen. *Blood Cancer J* 2016; **6**: e448.

24 Kumar S, Flinn I, Richardson PG, Hari P, Callander N, Noga SJ *et al.* Randomized, multicenter, phase 2 study (EVOLUTION) of combinations of bortezomib, dexamethasone, cyclophosphamide, and lenalidomide in previously untreated multiple myeloma. *Blood* 2012; **119**: 4375–4382.

25 Palumbo A, Cavallo F, Gay F, Di Raimondo F, Ben Yehuda D, Petrucci MT *et al.* Autologous transplantation and maintenance therapy in multiple myeloma. *N Engl J Med* 2014; **371**: 895–905.

26 Slade M, Martin TG, Nathwani N, Fiala MA, Rettig MP, Gao F *et al.* Ixazomib, lenalidomide and dexamethasone consolidation with randomized ixazomib or lenalidomide maintenance after autologous transplant in newly diagnosed multiple myeloma. *Leukemia* 2022; **36**: 2917–2921.
[truncated: 2,678 more chars]
